# Combined small RNA and degradome sequencing to identify miRNAs and their targets in response to drought in foxtail millet

**DOI:** 10.1186/s12863-016-0364-7

**Published:** 2016-04-12

**Authors:** Yongqiang Wang, Lin Li, Sha Tang, Jianguang Liu, Hanshuang Zhang, Hui Zhi, Guanqing Jia, Xianmin Diao

**Affiliations:** College of Life Science, Hebei Normal University, Shijiazhuang, 050012 People’s Republic of China; Institute of Crop Sciences, Chinese Academy of Agricultural Sciences, Beijing, 100081 People’s Republic of China; Institute of cotton, Hebei Academy of Agricultural and Forestry Sciences, Shijiazhuang, 05003 People’s Republic of China

**Keywords:** miRNA, High-throughput sequencing, Drought, Foxtail millet, Degradome

## Abstract

**Background:**

Foxtail millet (*Setaria italica*) is a diploid C_4_ panicoid species. Because of its prominent drought resistance, small genome size, self-pollination, and short life cycle, foxtail millet has become an ideal model system for studying drought tolerance of crops. MicroRNAs (miRNAs) are endogenous, small RNAs that play important regulatory roles in the development and stress response in plants.

**Results:**

In this study, we applied Illumina sequencing to systematically investigate the drought-responsive miRNAs derived from *S. italica* inbred An04-4783 seedlings grown under control and drought conditions. Degradome sequencing was applied to confirm the targets of these miRNAs at a global level. A total of 81 known miRNAs belonging to 28 families were identified, among which 14 miRNAs were upregulated and four were downregulated in response to drought. In addition, 72 potential novel miRNAs were identified, three of which were differentially expressed under drought conditions. Degradome sequencing analysis showed that 56 and 26 genes were identified as targets of known and novel miRNAs, respectively.

**Conclusions:**

Our analysis revealed post-transcriptional remodeling of cell development, transcription factors, ABA signaling, and cellar homeostasis in *S.italica* in response to drought. This preliminary characterization provided useful information for further studies on the regulatory networks of drought-responsive miRNAs in foxtail millet.

**Electronic supplementary material:**

The online version of this article (doi:10.1186/s12863-016-0364-7) contains supplementary material, which is available to authorized users.

## Background

Foxtail millet (*Setaria italica*), a diploid C_4_ panicoid species with a small genome of ~ 510 Mb [1], is an important food and fodder grain crop in arid and semi-arid regions of Asia, especially in China and India. The majority of foxtail millet varieties are abiotic stress tolerant, particularly to drought. The water use efficiency (WUE) of foxtail millet is higher than that of maize, wheat, and sorghum given that maize requires 470 g of water, wheat requires 510 g, and foxtail millet requires only 257 g for 1 g of dry biomass [2]. Because of its drought tolerance, foxtail millet was described as the “Oasis of Arid Agriculture” [3]. The prominent drought resistance, small genome size, self-pollination, and short life cycle has made foxtail millet an ideal model system for studying drought tolerance in plants. Many drought-inducible genes with various functions have been identified by molecular and genomic analyses in foxtail millet [4–7]. Recently, with the discovery of small RNAs, post-transcriptional regulation of drought response by miRNAs has been examined [8–10].

MicroRNAs (miRNAs) are endogenous, 20–24 nt noncoding RNAs that play important regulatory roles in eukaryotes by targeting mRNAs for cleavage or translational repression [11]. In plants, primary miRNAs are transcribed by RNA polymerase II [12, 13] and then processed by Dicer-like (DCL) into precursors (pre-miRNA) with stem-loop structures. Subsequently, these pre-miRNAs are cleaved into miRNA::miRNA* duplexes and exported to the cytoplasm by the HASTY protein [14, 15]. The mature miRNAs are incorporated into the RNA-induced silencing complex (RISC) to target specific mRNAs and downregulate the expression of target mRNAs [16]. Increasing evidence indicates that miRNAs have an influential role in numerous processes in plants, including development, abiotic stress tolerance, nutrient starvation, and metabolism [17–20].

Drought is a major abiotic stress factor that limits crop productivity. Plants respond to drought stress through complex mechanisms that allow them to adapt to water-deficit conditions by synthesizing or suppressing specific drought-related proteins. Recent studies have shown that miRNAs play important roles in drought tolerance by regulating the expression of drought-responsive genes [21, 22]. Many miRNAs associated with abiotic stress responses have been identified in plant species, including *Arabidopsis thaliana* [23, 24], *Oryza sativa* [25–28], *Zea mays* [29, 30], *Populus* [31], and *Medicago truncatula* [32]. Li et al. (2008) reported that miR169 was downregulated by drought stress through an ABA-dependent pathway, and miR169-overexpressing plants showed enhanced leaf water loss and were more sensitive to drought stress than wild-type plants [33]. Zhou et al. (2013) found that transgenic Creeping Bentgrass overexpressing *Osa-miR319a* showed morphological changes and enhanced drought and salt tolerance [34].

Next-generation sequencing and bioinformatics prediction provide effective methods for plant miRNA discovery and analysis. In foxtail millet, Yi et al. (2013) characterized the miRNA repertoire by deep sequencing and identified 43 known miRNAs and 172 novel miRNAs [35]. Khan et al. (2014) identified 355 mature miRNAs through computer analysis [36], and Han et al (2014) identified 271 foxtail millet miRNAs belonging to 44 families using a bioinformatics approach [37]. These results are useful for miRNA studies in foxtail millet. However, there has been no study on the differential expression of miRNAs in foxtail millet under drought stress, and most microRNA targets in previous studies were predicted by bioinformatics, which require confirmation.

Various studies have indicated that different genotypes of plant showed different gene-expression profiles in response to drought, and more genes were significantly drought regulated in the sensitive compared with the tolerant cultivars [38]. Thus, in this study a drought-sensitive cultivar was used to study potential drought-responsive miRNAs and their targets in foxtail millet. We constructed two libraries of sRNAs from foxtail millet under control and water-deficit conditions, which were sequenced using the Illumina sequencing platform. Degradome sequencing was applied to directly detect cleaved miRNA targets at a global level in foxtail millet.

## Methods

### Plant materials and stress treatment

To evaluate drought resistance at the seedling stage, 10 varieties of foxtail millet were subjected to repeated drought treatments [39], and the results are shown in Additional file 1. Among them, An04-4783 was identified to be more sensitive to drought stress. An04-4783 is a mordern cultivar of *S. italica*, which was developed a decade ago in Anyang academy of agriculture sciences, Henan, China, and is publicly available as germplasm resource in Chinese Crop Germplasm Information System (CGRIS). The An04-4783 seedlings were grown in the greenhouse (28 °C day/20 °C night and 16 h day/8 h night) with compound media, including vermiculite and sand. Plants were well irrigated according to evaporation demand and watered with 1 × Hoagland nutrient solution. After 14 days, deficit irrigation treatments were applied by withholding watering on the stressed pots while control pots were well-watered. Leaf water potential (LWP) was measured using the psypro WP data logger (Wesco) as an indicator of stress level. Fresh leaves were sampled from control plants (CL, well watered, Ψ_wp_ = -0.5 MPa) and moderately drought-stressed plants (DT, Ψ_wp_ = -1.4 MPa), and frozen in liquid nitrogen for RNA extraction.

### Small RNA library and degradome library preparation and sequencing

Total RNAs was extracted from mixed leaf tissues using the Tri-Reagent kit (Sigma, USA) according to the manufacturer’s instructions. The 5’ and 3’ adaptors were ligated sequentially to the RNAs and amplified by RT-PCR. Small RNAs from 140 bp to 160 bp were selected by polyacrylamide gel electrophoresis and sequenced on an Illumina HiSeq 2000 sequencer.

Degradome libraries were constructed as described previously [40] with some modification. Total RNAs from control plant leaves was sent to BGI (Beijing, China) to prepare the degradome sequencing libraries. The protocol is as below: 1. Approximately 150 ng of mRNA was used to anneal with biotinylated random primers (BPRs). 2. Streptavidin capture of RNA fragments through BPRs. 3. 5’ adaptor was ligated to only those RNAs containing 5’-monophosphates, followed with reverse transcription and PCR. 4. Libraries were sequenced using the 5’ adapter only, resulting in the sequencing of the first 50 nucleotides of the inserts that represented the 5’ ends of the original RNAs. Single-end sequencing (50 bp) were then performed on an Illumina Hiseq 2000 [41].

### Small RNA data analysis

Raw sequences were processed by removing adapters and discarding low-quality sequences, and clean small reads were obtained and aligned against the *S. italica* genome (Phytozome v10.0) using bowtie software v1.01 [42] with perfect match. The matched reads were then used as queries to search against the Rfam database [43] to remove rRNA, tRNA, snRNA, and snoRNA, and the remaining reads were search against the miRBase database (Release 21) [44] and evaluated using miRcheck [45]. Only miRNAs matched to known miRNAs with no more than two mismatches in the miRBase database and whose precursors could fold into stem-loop structures were considered to be known miRNAs of *S. italica*. The unannotated sRNAs were subsequently analyzed for potential novel miRNAs using miRCat software with default plant parameters [46] and psRobot software [47]. Secondary structures of potential miRNAs were checked using RNAfold software [48]. The criteria we used to identify miRNAs were referred to previous research Meyer et al (2008) [49] and Li et al (2011) [50] and listed as follows: (1) precursor sequence could form a marked stem-loop hairpin secondary structure, (2) there are no more than four mismatches between miRNA and miRNA*, (3) asymmetric bulges are minimal in size no more than 2 bases in mature sequence, and (4) the maximum free energy allowed for a miRNA precursor was -18 kcal mol^-1^. To exclude the contaminative effect of small interfering RNA (siRNA) on miRNA identification, we mainly considered two main factors: 1. Structural feature of siRNA. siRNA is a class of short double-stranded RNA molecules, each strand of which is 2 nt longer than the other at the 3’end [50]. 2. Limit the number of loci of identified miRNAs. The number of loci of known miRNAs in plant genome is mostly less than 24 [51]. Higher number of loci often occurs in repeat-rich regions from which siRNAs are produced.

### Degradome analysis

After adapter trimming and removing low quality reads, clean reads perfectly matching the *S. italica* genome were collected for further analysis using PAREsnip software [52]. The cleaved target transcripts were categorized into five classes based on the abundance of degradome tags indicative of miRNA-mediated cleavage. Category 0 comprised the sequences whose abundance at the cleavage site was the only maximum on the transcript; in category 1, the reads abundance at the cleavage site was the maximum but not unique; category 2 consisted of sequences whose abundance at the cleavage site was higher than the median but not the maximum; category 3 included sequences whose abundance at the cleavage site was equal to or below the median; the remaining sequences, which were the only raw reads at the cleavage site, were classified as category 4.

### Differential expression analysis of miRNAs

The reads of each library were normalized by TPM (Transcript per million), normalized expression = (actual miRNA count/total count of clean reads) × 1,000,000 [49, 50]. Differential expression between drought and control conditions was calculated using IDEG6 software [53] (http://telethon.bio.unipd.it/bioinfo/IDEG6_form/). Audic and Claverie, Fisher’s exact test, and general chi-square statistical methods were applied. miRNAs with absolute value log_2_ A fold-change (DT/CK) ≥ 1 and a significance threshold ≤ 0.01 were considered to reflect significantly differentially expressed (DE) miRNAs.

### miRNA validation

To validate the results of miRNAs from high-throughput sequencing, qRT-PCR was performed using SYBR Green PCR Master Mix on qTOWER 2.2 (Analytik Jena AG). The small RNAs were extracted from leaves of foxtail millet using the miRNA pure Mini kit (Beijing ComWin Biotech). The miRNA cDNA kit (Beijing ComWin Biotech) was used in the reverse transcription reaction. Quantitative real time PCR was performed using the miRNA Real-Time PCR Assay kit (Beijing ComWin Biotech). Each PCR reaction consisted of 2 μl of product from the diluted reverse transcription reaction, 0.5 μl sequence-specific forward primer, 0.5 μl universal reverse primer, 12.5 μl of 2 × miRNA qPCR premix (with SYBR and ROX), and 9.5 μl of nuclease-free water. The U6 gene was used as an internal control. The reaction conditions were set as follows: 95 °C for 10 min followed by 40 cycles of 95 °C for 15 seconds and 60 °C for 1 minute, with a final dissociation curve analysis. All reactions were performed with three biological replicates for each sample (primer list in Additional file 2). Real-time PCR data analysis was performed by qPCR soft 3.0 (Analytik Jena AG).

### Drought-related miRNA-mRNA network construction

Based on the analysis of small RNA data and degradome sequencing, drought-related miRNAs and Arabidopsis genes homologous to drought-related miRNA targets were used to construct the miRNA–mRNA interaction network. The functional relationship between two genes was retrieved from STRING database v10 [54]. If two genes were annotated to be related, we added an edge between them in the network drawn using the Cytoscape software [55].

## Results

### Physiological response of foxtail millet to drought stress

**Fig. 1:**
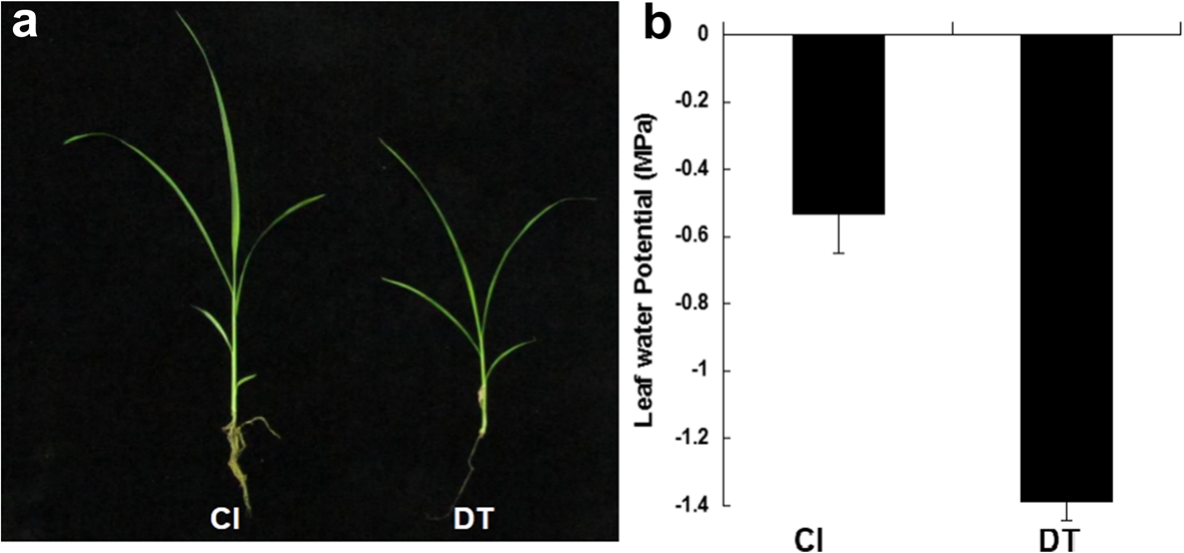
Effects of drought stress on phenotypic alterations and changes in leaf water potential (WP) in foxtail millet seedlings. **a** After drought treatment for 3 days, the plants were smaller compared with control plants, and the leaves changed color. **b** Leaf water potential (LWP) of control and drought treatment plants. After drought treatment, LWP decreased from -0.5 Mp (CL) to -1.4 Mp (DT)

Seedlings of the drought-treated group (DT) were subjected to a natural soil drying process, whereas the control group (CL) was irrigated to field water capacity daily. Pre-dawn leaf water potential (Ψ_wp_) was measured throughout the process to monitor the intensity of drought stress. On the third day of drought treatment, Ψ_wp_ decreased by about one-half in the DT compared with the CL group (Fig. 1a). The drought-stressed seedlings showed yellow and rolling leaves as well as reduced plant growth (Fig. 1b), which indicated that drought stress significantly affected plants in the DT group during this time.

### High-throughput sequencing of small RNAs in foxtail millet

**Fig. 2:**
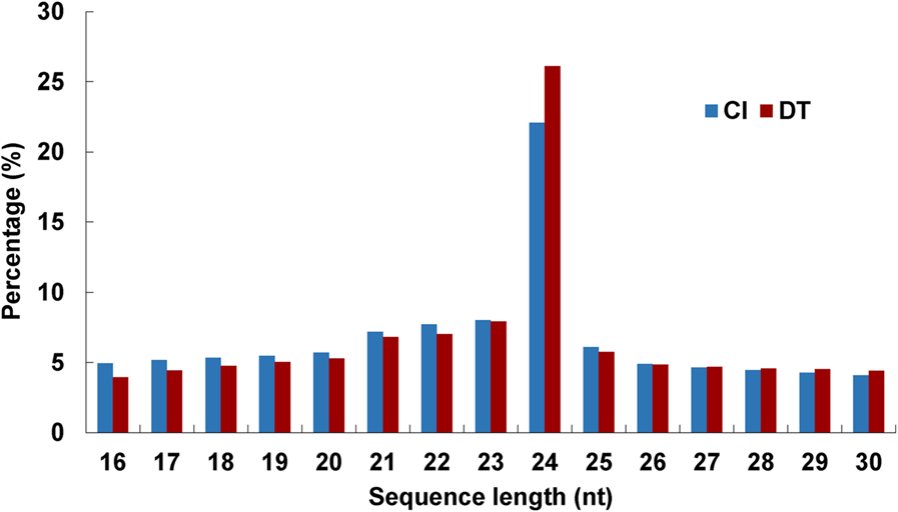
Sequence length distribution of sRNA in the library of control and drought versions of foxtail millet

To identify miRNAs from foxtail millet under water-deficit conditions, two small RNA libraries were constructed based on sequencing data from CL and DT groups of foxtail millet leaves. Raw sRNA sequencing reads have been deposited at EMBL (https://www.ebi.ac.uk/) under accession number ERP014347. After removing the low-quality sequences, adapter, and sequences smaller than 16 nt and larger than 30 nt, 14,124,084 clean reads (1,487,858 unique sequences) in CL and 10,374,842 clean reads (1,579,854 unique sequences) in DT were obtained. Analysis of the length distribution of unique sRNAs reads showed that the two libraries contain similar data, with the 24 nt sRNAs being the most abundant (Fig. 2) and this result was consistent with previous studies in foxtail millet [35] and maize [56, 57]. The common and different sequences in the CL and DT libraries were analyzed for unique and total sRNAs (Table 1), and 52.41 % of total sequences appeared in two libraries. However, only 11.85 % percent of unique sequences overlapped between the two libraries. This limited overlap indicated that there was diversity in the sRNAs of foxtail millet in response to drought.

**Table 1:** Statistical analysis of common and specific sRNAs between control (CL) and drought-treatment (DT) libraries

Type	Unique sRNAs	Percent (%)	Total sRNAs	Percent (%)
Total_sRNA	3067712	100.00 %	24498926	100.00 %
CL & DT	363399	11.85 %	12839242	52.41 %
CL specific	1124459	36.65 %	1284842	5.24 %
DT specific	1579854	51.50 %	10374842	42.35 %

To obtain a comprehensive view of the sequence distribution of all sRNA reads, clean reads were divided into different categories of exon-sense, exon-antisense, intron antisense, intron sense, and sRNAs, including non-coding RNAs (tRNA, rRNA, snRNA, snoRNA and miRNA) through mapping against the Rfam database and miRBase (Table 2). Our results showed that the proportion of miRNA sequences represented a very small fraction (< 1 %) of the total and unique sequences. The majority of unique sequences (> 90 %) were classified as other, which could not be mapped to any known reference database. The largest fraction of unannotated sequences may represent novel miRNAs and other classes of small ncRNAs. Similar results have been found in other plant species [58].

**Table 2:** Statistical analysis of sRNAs for control (CL) and drought-treatment (DT) libraries

CL (control)					DT (drought-treatment)			
Type	Uniq sRNAs	Percent	Total sRNA	Percent	Uniq sRNAs	Percent	Total sRNA	Percent
Exon antisense	137	0.01	141	0.00	94	0.01	95	0.00
Exon sense	394	0.03	491	0.00	180	0.01	191	0.00
Intron antisense	34	0.00	35	0.00	29	0.00	29	0.00
Intron sense	225	0.02	252	0.00	91	0.01	93	0.00
miRNA	8698	0.58	117589	0.83	7814	0.49	104080	1.00
rRNA	98782	6.64	2310754	16.36	118155	7.48	2026243	19.53
repeat	10278	0.69	184428	1.31	10425	0.66	197797	1.91
tRNA	7782	0.52	217892	1.54	9434	0.60	152753	1.47
others	1361528	91.51	11292502	79.95	1433632	90.74	7893561	76.08
Total	1487858	100.00	14124084	100.00	1579854	100.00	10374842	100.00

### Identification of known miRNAs

**Fig. 3:**
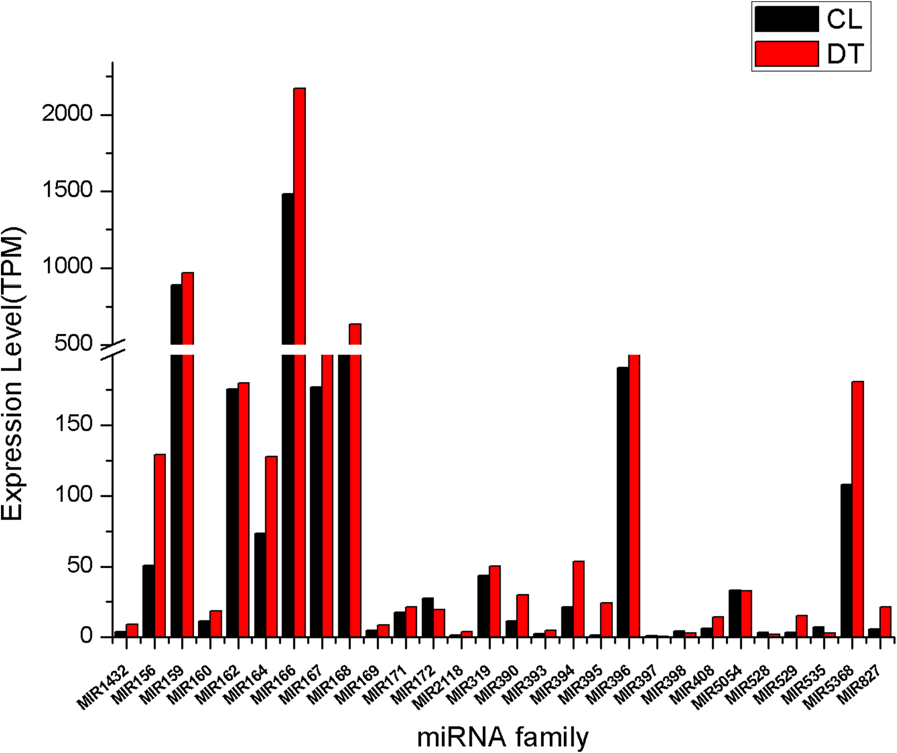
Expression levels of known miRNA families in CL and DT libraries

To identify the known miRNAs of foxtail millet (conserved and species-specific), clean reads of two libraries were searched against mature plant miRNAs from the miRNA database. After filtering miRNAs whose pre-miRNA could not form hairpin secondary structures, 81 miRNAs were identified in the CL and DT libraries, which were clustered into 28 families based on the similarity of the mature miRNA sequence. Among them, 29 miRNA* were identified based on sequence alignment. The length of pre-miRNA ranged from 66 to 222 nt and negative MFEs (minimum free energies) ranged from -32.1 to -98.9 kcal/mol (Additional file 3). Compared with the 48 foxtail millet miRNA families from a previous report by Bennetzen et al. [59], the results showed that these miRNA families are common. Analysis of all known miRNA family reads of two libraries showed that the number of reads varied significantly, ranging from 14 to 20,970 (1484.7 TPM) in the CL library and from 4 to 22,500 (2168.7 TPM) in the DT library. MIR166 was the most abundant miRNA family in both the CL and DT libraries. In contrast, MIR397 and MIR2118 showed low expression levels (Fig. 3). Based on analysis of location of precursor, we found that in foxtail millet, more than 87 % of known miRNAs are derived from intergenic regions, and others originate from coding sequence regions (Additional file 3). This result was consistent with previous studies [60].

### Identification of potential novel miRNAs in foxtail millet

After identifying exons, rRNA, tRNA, snoRNA, snRNA, and known miRNAs, we pooled the remaining unannotated sRNA sequences of two libraries and predicted novel miRNAs using miRcat software with default plant parameters and psRobot software. A total of 72 novel miRNA candidates were obtained. The length of precursor miRNA sequences varied from 61 to 208 nt, and the negative MFEs of the identified foxtail millet miRNA precursors varied from -18.0 to -111.8 kcal/mol (Additional file 4). The secondary structures of novel miRNA precursors shown in Additional file 5. Among these potential miRNAs, eight miRNAs with complementary miRNA* were identified, which supported their role as novel miRNAs of foxtail millet (Table 3). The majority of these miRNAs had relatively low expression, which was consistent with previous studies in other plants [58, 59]. The low abundance of novel miRNAs suggests that the majority of foxtail millet-specific miRNAs are expressed at low levels. Characteristics of novel miRNA precursor location were similar to known miRNA, about 72 % miRNAs were from intergenic regions, 20 % miRNAs were derived from intronic and 8 % originated from coding sequences.

**Table 3:** Potential novel miRNAs with miRNA* found in *S. italica*

miRNA	Mature Sequence	Arm	Length (nt)	miRNA* sequence	percursor location	MFE
sit_novel_miR10	GTATGGAAGAACTGCTGCGCCA	3p	22	ATGGTGTACCGGTTGTTATGC	scaffold_7:35210708..35210784:-	-30.2
sit_novel_miR15	CACTATAGGAGCTGGCCAGGT	5p	21	AGGCTAGGCTTGCGACTGGAG	scaffold_14:67096..67179:-	-31.4
sit_novel_miR30	TTAGGCTCGGGGACTATGGTG	5p	21	CCGTAGCCCCTGCTCCTGATG	scaffold_5:4967704..4967886:-	-101.4
sit_novel_miR41	GTGCTCCCTCCCGTTGTCACC	3p	21	TGACAACGAGAGAGAGCA	scaffold_8:21627028..21627140:+	-71.2
sit_novel_miR42	TGAGCCGAACCAATATCACTC	3p	21	CGTGGTGTTGTTTCGGCTCATG	scaffold_1:34236041..34236153:+	-53.1
sit_novel_miR45	GGATATTGGTGCGGTTCAATC	5p	21	TTGAGCCGTGCCAATATCACG	scaffold_7:30396911..30397013:-	-50.3
sit_novel_miR48	TGGTAGGCATTCTGGTTAAGT	3p	21	TTAGCCAAGAATGACTTGCCTATC	scaffold_3:6158117..6158229:+	-49.2
sit_novel_miR56	TTGACAGAAGAGAGCGAGCAC	5p	21	GCTCGCTCCTCTTTCTGTCAGC	scaffold_4:31435223..31435323:+	-66.6

### Differential expression analysis of known and novel miRNAs of foxtail millet under drought stress

To identify drought-associated miRNAs of foxtail millet, we removed miRNAs whose expression levels were too low to be analyzed for differential expression (sequencing frequency < 10 in CL and DT libraries) and compared the normalized expression of miRNAs between the CL and DT libraries. A total of 18 known miRNAs belonging to 16 families were significantly expressed with more than one log2 fold change (Additional file 6). Among these DE miRNAs, 14 miRNAs (sit-miR1432-3p, sit-miR156a-5p, sit-miR156b-5p, sit-miR164a-5p, sit-miR167b-5p, sit-miR171c-3p, sit-miR2118-3p, sit-miR390-5p, sit-miR394-5p, sit-miR395-3p, sit-miR408-3p, sit-miR529a-3p, sit-miR529b-3p, and sit-miR827) were upregulated and 4 miRNAs (sit-miR159b-3p, sit-miR319c-5p, sit-miR528-5p and sit-miR535-5p) were downregulated; some of these miRNA families have been associated with drought stress in previous studies: miR156 [31, 61], miR159 [23], miR167 [23, 61], miR395 [62], and miR408 [63]. We also identified three potential novel miRNAs considered to be drought-response miRNAs based on the differential expression between the CL and DT libraries. Of these miRNAs, two (sit-novel-miR10, sit-novel-miR56) were upregulated, and one (sit-novel-miR18) was downregulated (Additional file 7).

**Fig. 4:**
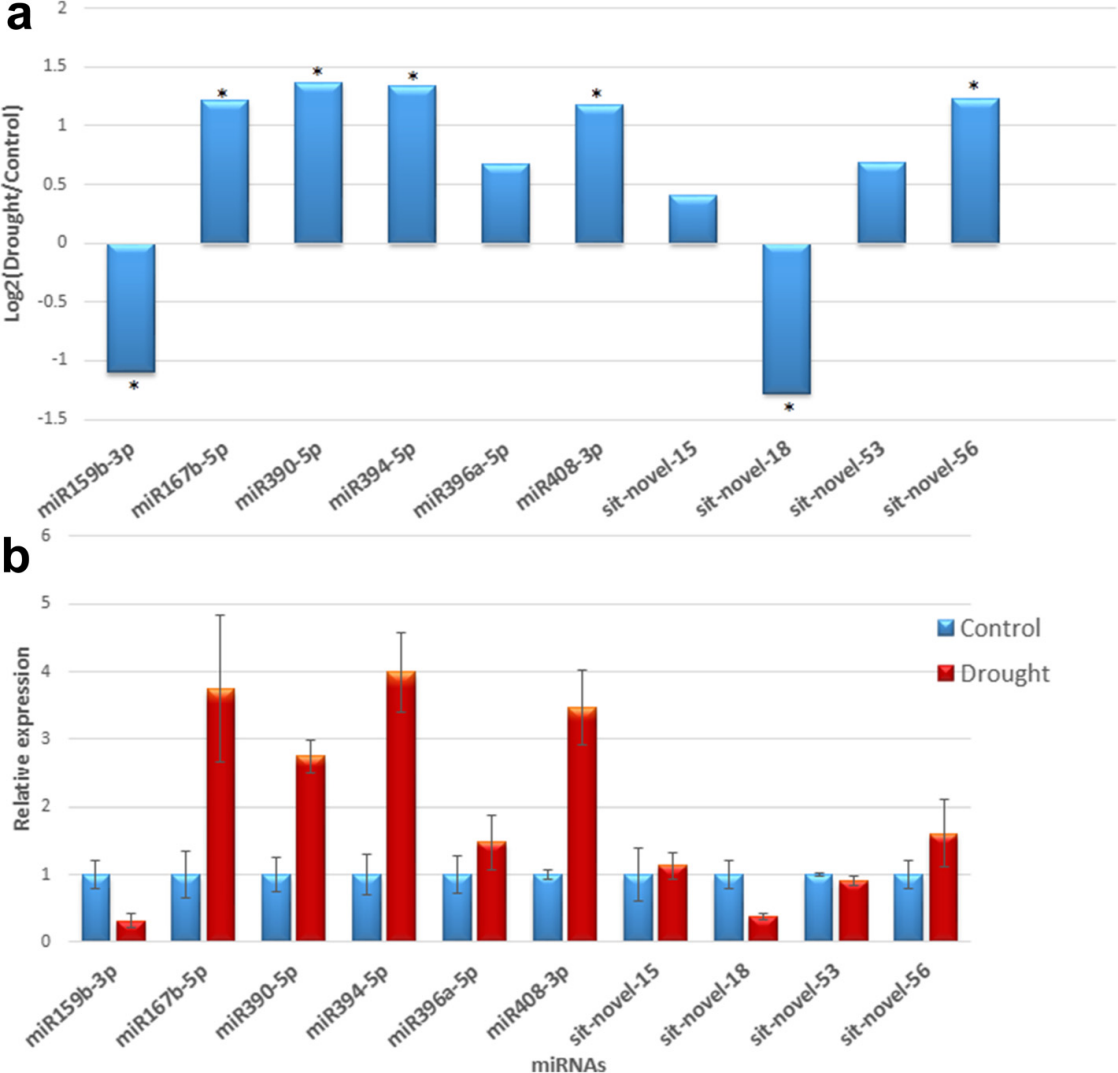
Differential expression analysis of conserved and novel drought-responsive miRNAs. **a** Fold change (log_2_) in control library relative to drought library detected by solexa small RNA sequencing. **b** The relative expression level of miRNAs measured by RT-qPCR. * means significant difference between control and drought stress at *P* ≤ 0.01

**Fig. 5:**
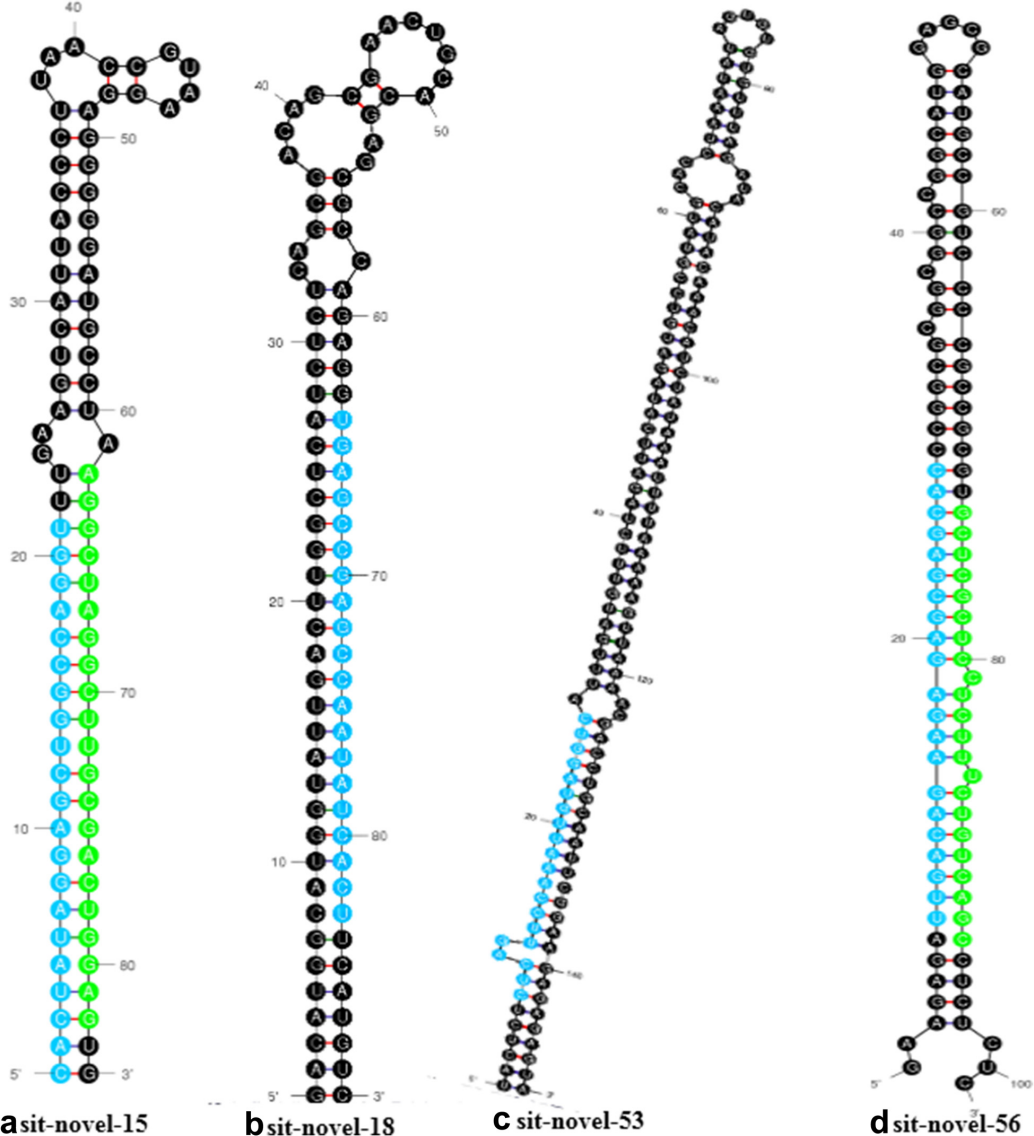
Secondary structure prediction of novel foxtail millet miRNA precursors. The blue colored sequences represent mature miRNA, and the green colored sequences represent the miRNA* (**a**, sit-novel-15; **b**, sit-novel-18; **c**, sit-novel-53; **d**, sit-novel-56).

To verify the results of miRNA sequencing and bioinformatics analysis, six known miRNAs (sit-miR159b, sit-miR167b, sit-miR390, sit-miR394, sit-miR396a, and miR408) and four novel miRNAs (sit-novel-miR15, sit-novel-miR18, sit-novel-miR53, and sit-novel-miR56) were selected randomly for validation by qRT-PCR. The results showed that the fold change of expression obtained by qRT-PCR was not completely consistent with bioinformatics analysis results, but the expression trend was similar (Fig. 4). The stem-loop secondary structure of four novel miRNAs is shown in Fig. 5. These results suggested that Solexa sequencing was successfully applied to identify drought-related miRNAs in foxtail millet.

### Target prediction of miRNAs and validation by degradome sequencing

In foxtail millet, numerous miRNA targets have been predicted previously [35, 36], but few miRNA targets have been validated experimentally. To identify miRNA targets in foxtail millet at the global level, we employed the degradome sequencing approach to identify target genes for known miRNAs and candidate novel miRNAs. Raw sequencing data generated by degradome sequencing are available at EMBL with the accession number ERP014368. After removing adapter sequences and low-quality tags, we obtained a total of 11,762,879 clean reads (3,528,168 unique reads) representing the 5’ uncapped ends, of which 7,239,426 (2,433,599 unique reads) were perfectly matched to the *S. italica* genome. The reads that perfectly mapped to the genome were subjected to further analysis using PAREsnip software [52].

In this study, 56 target genes for 12 known miRNA families were identified. Based on the abundance of degradome tags at the target sites, these cleaved targets were classified into five categories; 42 target genes were classified into category 0, 4 target genes into category 1, 6 target genes into category 2, 2 target genes into category 3, and 2 target genes into category 4 (Table 4). The detailed information is provided in Additional file 8, and the t-plots for targets are illustrated in Additional file 9. The majority of known miRNAs regulated multiple target genes (ranging from 1 to 11). Among them, the sit-miR156 family, with 11 unique target genes, had the largest number of target genes; the sit-miR172 and sit-miR393 families had only one target gene, and the others had two to eight targets. Functional analysis of these target genes showed that they were enriched in transcription factors, such as SBP-box transcription factor (sit-miR156), MYB (sit-miR159), ARF (sit-miR160), NAC (sit-miR164), HD-zip transcription factor (sit-miR166), GRAS (sit-miR171), and GRF (sit-miR396). These results were consistent with a previous study in *S. italica* and other species [8, 35].

**Table 4:** Targets of known miRNAs identified by degradome sequencing.

Family	Target gene	Cleave position	category	Alignment score	Target gene annotation
miR156	Si013870m	837	0	1	SBP-box gene family member
	Si006471m	1311	0	1	SBP-box gene family member
	Si031154m	736	2	2	SBP-box gene family member
	Si013747m	981	0	1	SBP-box gene family member
	Si006472m	1210	0	1	SBP-box gene family member
	Si017749m	758	0	1	SBP-box gene family member
	Si030195m	924	2	1	SBP-box gene family member
	Si030892m	577	0	1	SBP-box gene family member
	Si026656m	904	0	2	SBP-box gene family member
	Si013308m	134	2	2.5	lectin-like protein kinase
	Si001804m	1444	0	1	SBP-box gene family member
miR159	Si000907m	970	0	1	myb domain protein 33 (MYB 33)
	Si002023m	493	1	3	unknown
	Si039629m	895	1	2	myb domain protein 101 (MYB101)
miR160	Si021833m	1181	0	1	auxin response factor 17 (ARF17)
	Si005991m	1767	0	2	auxin response factor 16 (ARF16)
	Si009541m	1349	0	2	auxin response factor 16 (ARF16)
	Si016559m	1481	0	3	auxin response factor 16 (ARF16)
	Si034525m	1328	0	2	auxin response factor 16 (ARF16)
	Si016509m	1364	0	3	auxin response factor 16 (ARF16)
miR162	Si010280m	2108	2	3	Adenosylmethionine decarboxylase family protein
	Si033853m	3138	0	1	dicer-like 1
	Si010282m	1537	2	3	Adenosylmethionine decarboxylase family protein
	Si002017m	941	2	2.5	NADP-dependent oxidoreductase
miR164	Si006975m	855	0	1/2	NAC domain containing protein
	Si017567m	864	0	3	NAC domain containing protein
	Si010553m	724	0	3	NAC domain containing protein
	Si017570m	840	0	3	NAC domain containing protein
	Si022747m	791	0	2	NAC domain containing protein
	Si017931m	968	0	3	NAC domain containing protein
	Si011317m	399	3	4	photosystem I subunit
	Si011389m	399	3	4	photosystem I subunit
miR166	Si034228m	851	0	2.5/3	Homeobox-leucine zipper family protein (HD-Zip)
	Si021201m	750	1	2.5/3	Homeobox-leucine zipper family protein (HD-Zip)
	Si034254m	799	0	2.5/3	Homeobox-leucine zipper family protein (HD-Zip)
	Si034251m	963	0	2.5/3	Homeobox-leucine zipper family protein (HD-Zip)
	Si000283m	577	4	2.5	Homeobox-leucine zipper family protein (HD-Zip)
miR167	Si021157m	3055	0	4	auxin response factor 8 (ARF8)
	Si000404m	2361	2	3.5	ARF
miR171	Si016508m	1082/1085	0/2	0.5/1.5	GRAS family transcription factor
	Si006258m	506/503	0/2	0.5/1.5	GRAS family transcription factor
	Si034686m	1001	1	2.5	Microtubule associated protein (MAP65/ASE1) family protein
	Si039098m	923	0	1.5	GRAS family transcription factor
miR172	Si018249m	597	0	3.5	cis-trans isomerase family protein
miR393	Si008122m	462	0	4.5	tesmin/TSO1-like CXC domain containing protein
miR396	Si035794m	679	0	2	growth-regulating factor 2(GRF2)
	Si008818m	662	0	2	growth-regulating factor 5(GRF5)
	Si026680m	329	0	2.5	growth-regulating factor 2(GRF2)
	Si011853m	935	0	2	growth-regulating factor 5(GRF5)
	Si034822m	896	0	2.5	growth-regulating factor 1(GRF1)
miR827	Si009522m	197	0	1	Major Facilitator Superfamily with SPX (SYG1/Pho81/XPR1) domain-containing protein
	Si009706m	197	0	1	Major Facilitator Superfamily with SPX (SYG1/Pho81/XPR1) domain-containing protein
	Si016510m	313	0	3	Major Facilitator Superfamily with SPX (SYG1/Pho81/XPR1) domain-containing protein
	Si009851m	197	0	1	Major Facilitator Superfamily with SPX (SYG1/Pho81/XPR1) domain-containing protein
	Si016511m	313	0	3	Major Facilitator Superfamily with SPX (SYG1/Pho81/XPR1) domain-containing protein
	Si009523m	197	0	1	Major Facilitator Superfamily with SPX (SYG1/Pho81/XPR1) domain-containing protein

Furthermore, we identified a total of 26 target genes for 9 novel miRNAs (Additional file 8, Additional file 10). Unlike the targets of known miRNAs, most targets of novel miRNAs fell into category 2. Of these 26 target genes, 10 were in category 2, 6 were in category 3, four were in category 4, three were in category 0 and 1. Descriptions of the target gene showed that the target genes of novel miRNAs had more diverse functions, including hydroxyproline-rich glycoprotein, dirigent-like protein, ubiquitin conjugating enzyme protein, and some unknown genes.

Pervious researches suggested that most miRNA targets are located in protein coding regions in plants, which is different from animals [64]. Our results also support this point of view. In this study, among 56 target genes for known miRNAs, 51 target sites located in coding region, and only 5 target sites located in UTR region. Similar results were found in targets for novel miRNAs, 20 out of 26 target sites located in coding region, and others located in UTR region (Additional file 8).

At the same time, we predicted targets of known and novel miRNAs using the psRNA Target program with default parameters [65]. The *S. italica* (foxtail millet) CDS library (provide by the JGI Genomic Project) was selected as the transcript/genomic library for the target search (Additional file 11 and Additional file 12). Compared with the results of degradome sequencing, 28 known miRNAs targets and 6 novel miRNAs targets could be detected using two methods. Additionally, some targets that were not detected by degradome sequencing, such as the targets of miR2118 and miR408, were predicted using psRNATarget.

**Fig. 6:**
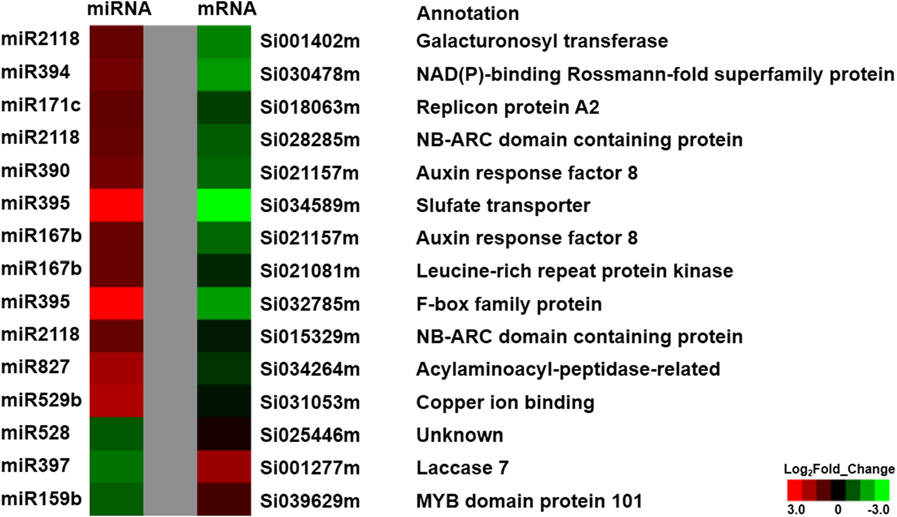
A combined heat map of the negative correlation between a miRNA and its target in foxtail millet under drought stress. The red represents upregulated expression, and the green represents downregulated expression

Combined with the results of transcriptome sequencing (data not published), we analyzed the relationship between DE miRNAs and their target’s expression. A total of 15 known miRNA–target pairs showed negative correlations (Fig. 6) and were composed of 10 miRNAs and 14 target genes. Functional annotation showed that some target genes, such as auxin response factor (ARF), NB-ARC domain-containing disease-resistance protein (NB-ARC), leucine-rich repeat receptor-like protein kinase (LRPK), F-box family protein, MYB transcript factor, and laccase, respond to stress. The sulfate transporter showed a strong negative association with miR395 and is known to play an important role in the response to abiotic stress [66]. In addition, Si030478m is homologous to NAD(P)-binding Rossmann-fold superfamily protein and involved in dehydrogenases of metabolic pathways, and Si031053m is homologous to copper ion-binding protein and involved in photorespiration.

### Drought-related miRNAs network

**Fig. 7:**
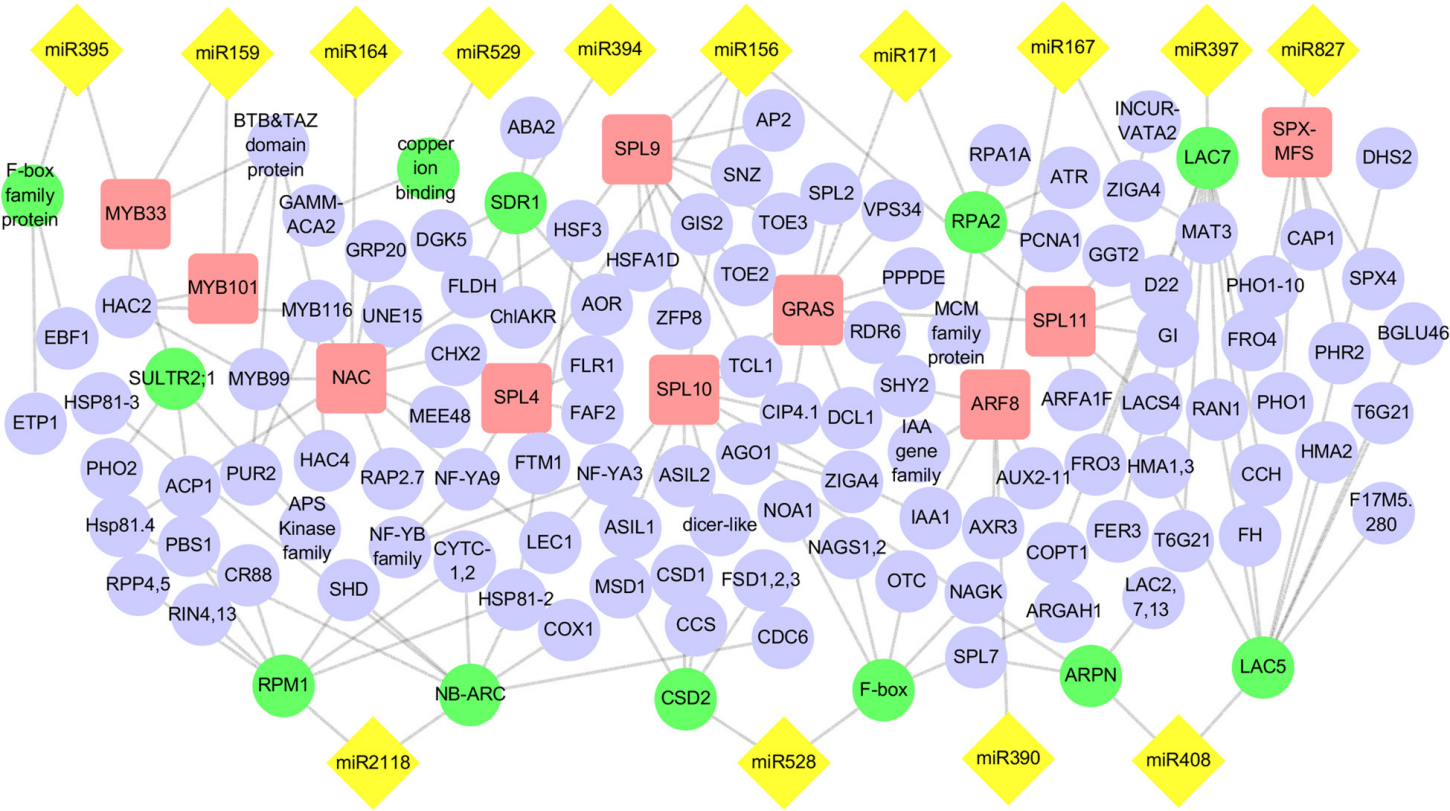
microRNA-mediated regulatory networks. Targets of DE miRNAs homologous to Arabidopsis and the constructed network based on the Protein–Protein Interaction data from the STRING database. Pink round rectangle represents the target identified by degradome sequencing, green ellipse represents the predicted target by psRNA Target, and other proteins are shown as a gray circle

To increase our understanding of the regulatory role of drought-related miRNAs, we constructed a miRNA-mediated interaction network based on targets and protein interaction data from the STRING database (Fig. 7). This network contains 14 DE miRNAs and 129 genes. The yellow diamond represents the miRNAs of foxtail millet that were DE in response to drought, the pink rectangle represents the target identified by degradome sequencing, the predicted target was labelled with a green ellipse, and other proteins are shown as a gray circle. The network revealed that many nodes were connected through protein–protein interaction data from the STRING database and form a complex network. In this network, most of these targets identified by degradome sequencing were transcription factors, including MYB, NAC, SPL, ARF, and GRAS. Targets predicted by *in silico* analysis included diverse important enzymes, such as NB-ARC, F-box, LAC, and copper ion-binding protein, which are believed to play an important role in the stress response.

## Discussion

As an important drought-tolerant crop, foxtail millet provides an ideal system to study drought tolerance. Increasing evidence has indicated that miRNAs play an important role in plant in response to drought. Considering the importance of miRNAs, many miRNAs of foxtail millet have been identified by high-throughput sequencing and bioinformatics approaches [35–37]. However, these studies focused on whole genome scales, which cannot reveal regulatory roles at the transcriptional level. Moreover, compared with identified miRNAs from other species, such as *Arabidopsis*, maize, and rice, there were fewer miRNAs in foxtail millet. The majority of foxtail millet-specific miRNAs, especially drought-related miRNAs, remain unidentified. In the present study, we constructed two sRNA libraries (control and drought treatment) and identified conserved, novel miRNAs, as well as drought-related miRNAs in foxtail millet.

### Drought-responsive miRNA

Comparisons of the expression levels of miRNAs in the control and drought libraries revealed that 18 miRNAs belonging to 16 miRNA families changed significantly. Of these miRNA families, some are thought to be associated with drought in other species, such as miR159, miR167, and miR390. During the response to drought, miR167 was upregulated in *Arabidopsis* [23] and *P. euphratica* [50]. In this study, sit-miR167b was significantly upregulated under drought stress, and two target genes (*Si021157m* and *Si000404m*) encoding ARF genes were identified based on degradome sequencing. Recently, a study in soybeans showed that miR167 positively regulates nodules and lateral roots by repressing the target genes *GmARF8a* and *GmARF8b* (homologous genes of Arabidopsis *AtARF8*) [67], which indicated that miR167 modulates root adaptation to drought stress. miR390 is another miRNA known to be involved in drought stress. In the present study, miR390 was upregulated, which was consistent with the results in cowpeas [68] and *Brachypodium distachyon* [69]. It was reported that miR390 targets the TAS genes, which generates ta-siRNAs (trans-acting small interfering RNA) and regulates Auxin Response Factor (ARF) to modulate lateral root emergence and organ polarity establishment. These results indicated that some miRNAs are conserved in response to drought across plants.

However, as reported in previous studies, some drought-related miRNAs show different expression patterns in response to drought stress. For example, miR156 was upregulated in cowpeas and barley in response to drought stress [68, 70], but it was downregulated in rice under conditions of drought. [27] Our results showed that two members of miR156 (sit-miR156a and sit-miR156b) were significantly upregulated, with more than one log2 fold change. Furthermore, several studies have shown that the expression of miR398 was induced by drought stress. It was reported that miR398 was downregulated under drought stress in *M. truncatula* [32], maize, and peach [71]. However, upregulation was observed in *Triticum dicoccoides* [62] and *M. truncatula* [63]. In this study, miR398 showed no significant change in response to drought. This is another example of a discrepancy in the miRNA expression patterns across different studies. Previous studies indicated that some miRNAs respond differently in different tissues under conditions of drought; for example, in barley, miR166 was upregulated in leaves but downregulated in roots, whereas miR156a, miR171, and miR408 were induced in leaves but unchanged in roots [61]. In addition, some studies have suggested that the pattern of miRNA expression differ in different genotypes within the same species; for example, the majority of miRNAs were upregulated during water-deficit stress in the sensitive soybean. However, for the tolerant genotype, the majority of miRNAs were downregulated [72]. The similar results were found in foxtail millet [60], after drought treated with PEG6000, majority of drought-related miRNAs in tolerant cultivar were up-regulated, whereas in sensitive cultivar showed down-regulated. These conflicting results require more detailed research to characterize drought-responsive miRNAs in plants.

In addition to the known miRNAs, we also identified 72 novel miRNAs; 3 of these miRNAs changed significantly after drought stress. Previous reports have suggested that highly conserved miRNAs are widespread with high expression, whereas less conserved miRNAs are often species-specific with weak expression [71]. Our results were also consistent with previous reports. In this study, most predicted novel miRNAs had very low counts compared with known miRNAs, and only eight novel miRNAs had more than 100 TPM. This result may be due to the evolutionary conservation of plant miRNAs, and these conserved miRNAs may be involved in key metabolic processes; thus, their expression may be higher than non-conserved miRNAs [71]. It is possible that these miRNAs play a species-specific role in drought responses in foxtail millet. Although these novel miRNAs have low expression, they may have an effect similar to that of miRNAs with high expression. For all 153 miRNAs we identified, 55 of them were also found in Yadav’s study [60]. As we expected, majority of these common miRNAs (92.7 %) show the similar expression patterns in response to stresses in both studies (Additional file 13).

### Target genes of foxtail millet miRNAs

In foxtail millet, the majority of miRNA targets were predicted using bioinformatics, and very few miRNA targets were identified experimentally. Degradome sequencing technology provides a powerful tool with which to study miRNA–target interactions at the transcriptome level. In this study, we identified 56 targets for 12 known miRNA families using degradome sequencing. Based on our analysis, the majority of these targets have a conserved function with miRNA targets in other species. Most of the identified targets of the known miRNAs belong to transcription factors, such as miR156 targeting the squamosal promoter-binding family (SPB), miR159 targeting MYB, miR160 targeting several auxin response factors (ARF), and miR164 targeting no apical meristem protein (NAC). It has been indicated that conserved miRNAs play a crucial role in post-transcriptional regulation in plant species [64]. These results suggest that degradome sequencing can be successfully applied to identify miRNA targets in foxtail millet with high accuracy and efficiency.

Gene function annotation of conserved miRNAs targets showed that most of them were classified into transcription factors. For example, in *Arabidopsis* and *Populus*, 95 conserved miRNAs targets were identified, 68 % of those encode transcription factor. In soybean, 82 % of miRNA targets were transcription factors [73]. Same results were also reported in rice [74], maize [75], and grapevine [76]. There are also a few miRNAs target to genes encoding enzymes of basic biochemical pathways. For example, miR397 targets laccase and miR398 targets copper superoxide dismutases. However, those were only constitute a minor portion of all identified target genes in plants [64].

In the present study, 26 targets for 9 novel miRNAs were identified using degradome sequencing. Compared with the targets of known miRNAs, the targets of novel miRNA had a wide variety of functions, including those involving glycoprotein, dehydrogenase, oxidoreductase, transcription factor, and unknown proteins. Another difference between the targets of conserved and novel miRNAs is that the majority of novel miRNA targets belonged to categories 2 and 3, and similar results were also found in *Brassica juncea* [77] and grapevine [76], which suggest these novel miRNAs are young and not fully stabilized evolutionarily.

Three potential novel miRNAs were considered drought-response miRNAs based on the DE between the CL and DT libraries. Only target of sit-novel-miR10 was identified based on degradome sequencing, and others were not identified based on degradome sequencing, possibly because these miRNAs regulate target genes by repressing translation. Further studies are required to increase our understanding of the regulatory mechanism of these miRNAs.

### miRNA role in the drought-stress responses of foxtail millet

Our study identified 16 known miRNA families and 3 novel miRNAs that were DE in foxtail millet under drought conditions (Additional file 6 and Additional file 7). Among known miRNAs, 34 unique targets of 6 DE miRNA families were validated using degradome sequencing (Table 4 and Fig. 7). As expected, the majority of these DE miRNAs were related to drought-stress responses in previous studies [78, 79]. For example, our study found that miR167b-5p was enriched and significantly upregulated in response to drought stress in foxtail millet. Similar results were shown in Arabidopsis [23], rice, and maize [80, 81], which indicates that miR167 can respond to ABA and control stomatal movement. miR390 was reported to be upregulated under drought stress in *Brachypodium distachyon* and *Vigna unguiculata* [69]. In *Arabidopsis*, miR390 mediated the miR390–tasiRNA–ARF regulatory pathway and regulated lateral root growth. In foxtail millet, a similar expression pattern and the same target gene of miR390 was identified via SL-qPCR and degradome sequencing, respectively. These results suggest that numerous miRNAs have conserved functions in regulating abiotic stress responses in various plant species. Combined with the miRNA expression patterns, target prediction, and degradome sequencing results (Figs. 6 and 7), our study increases our understanding about how foxtail millet An04 responded to water deficiency:

i. *Growth repression under drought conditions*. Morphology and physiology experiments have shown that foxtail millet An04 has the lowest germination rates and significant growth repression when lacking a water supply. Several DE miRNAs and their targets involved in cell growth and development regulation were identified in drought-treated plants. miR2118 were induced up to ~ two-fold in An04 under water-deficient conditions, whereas its predicted target genes, *Si001402m*, were repressed markedly. *Si001402m* encoded a galacturonosyl transferase, and the homologous gene in *Arabidopsis* plays an important role in glucuronoxylan accumulation and secondary cell wall development [82]. Another putative mechanism crucial for cell growth remodeling in foxtail millet in response to drought is the miRNA397–laccase regulatory pathway. We found that miRNA397 and its target laccase 7 show an inverse correlation in gene expression, indicative of classical miRNA-mediated mRNA degradation. It has been shown that modulation of laccase by miR397 can affect plant biomass production in both *Arabidopsis* and *Populus* [83, 84].
ii. *Drought-responsive transcription factors regulated by miRNAs*. In our study, several miRNA families were shown to regulate transcription factors and control the plant response to drought stress. The NAC transcription factor (TFs) family is one of the TFs that regulate drought tolerance in plants. In *Arabidopsis*, miR164 mediates the cleavage of NAC1, which further downregulates auxin signals and reduces lateral root growth [19]. In rice, the miR164-targeted NAC genes were shown to be negative regulators of drought tolerance [85]. Our study showed that miR164a was induced by water treatment; six target genes (*Si006975m*, *Si017567m*, *Si010553m*, *Si017570m*, *Si022747m*, and *Si017931m*), all of which belong to the NAC transcription factor family, were identified by degradome sequencing. It is possible that the enhanced expression of miR164a represses NAC gene expression and regulates drought responses in foxtail millet. Another example of the miRNA–TFs co-regulatory pathway found in our study is miR156-targeted squamosal-promoter binding protein-like (SPL) networks. Stief et al. (2014) demonstrated that miR156 can target SPL transcription factor genes and regulate tolerance to recurring environmental stress [86]. Our results showed that miR156 was induced by drought, and SPL4, 9, 10, and 11 were identified as its targets, indicating that miR156-SPLs play a role in response to drought in foxtail millets.
iii. *miRNAs mediate Abscisic acid (ABA) signaling*. ABA is a phytohormone critical for drought-stress signaling in plants. miRNA159 is known to be a negative regulator of ABA responses [87]. In *Arabidopsis*, ABA increases miR159 accumulation through an ABI3-dependent pathway and suppresses MYB33 and MYB101 expression, which eventually induced plants stress responses. The same genes and their expression patterns were identified in foxtail millet, suggesting that the mechanism by which miR159 regulated ABA signaling through MYB33 and MYB101 transcript was conserved in foxtail millet An04. miR394 is involved in ABA-dependent Arabidopsis salt and drought stress responses [88], and upregulation of miR394 in drought-treated An04 plants may initiate ABA- and stress-responsive gene expression to allow for plant adaption to water-deficient conditions.
iv. *miRNAs involved in cellar homeostasis*. It is important for plants to sustain cellar homeostasis under environmental stress. Two DE miRNAs (miR528 and miR395) were shown to be involved in cellar homeostasis maintenance. In our study, miR528 was significantly downregulated by drought, and the same result was reported in maize. The target gene of miR528 encoded a peroxidase (POD), which is an important component of the antioxidative enzyme system in plants. The downregulation of miR528 may promote the removal of excessive reactive oxygen species (ROS) and help maintain cellar homeostasis in foxtail millet under drought conditions. miR395 was enriched in the drought-treated group, and its putative target was predicted to be a sulfate transporter, SULTR2;1. Regulation of the sulfate assimilation process through the miR395–SULTR2;1 interaction had been confirmed in Arabidopsis [89]. Moreover, sulfate transporters play a role in re-equilibrating the flux of sulfate between and within different tissues and improving drought tolerance in plants [66].

## Conclusions

In the present study, we detected 18 miRNA members in 16 families and predicted 3 novel miRNAs in response to drought in foxtail millet. Furthermore, 56 targets for 12 known miRNA families and 26 target genes for 9 novel miRNAs were identified by degradome sequencing at the global level. These results provide useful information for the study of drought-responsive miRNAs in foxtail millet. Further studies are required to identify these miRNAs and their targets using techniques such as RLM-RACE. On the other hand, it is important to determine whether these genes enhance drought tolerance in transgenic plants.

## Ethics (and consent to participate)

Not applicable.

## Consent to publish

Not applicable.

## Availability of data and materials

Raw data supporting our findings can be found at EMBL-EBI European Nucleotide Archive (https://www.ebi.ac.uk/) under accession numbers ERP014347 and ERP014368.

## Additional files

Additional file 1: The survival rate of 10 varieties of foxtail millet under repeated drought treatments. (TIF 166 kb) Additional file 2: Primers used for RT-PCR in this study. (DOC 35 kb) Additional file 3: Detailed information of known miRNAs in foxtail millet. (XLS 72 kb) Additional file 4: Detailed information of novel miRNAs in foxtail millet. (XLS 58 kb) Additional file 5: The secondary structures of novel miRNA precursors (PDF 324 kb) Additional file 6: Differential expression analysis of known miRNAs under drought stress. (XLS 21 kb) Additional file 7: Differential expression analysis of novel miRNAs under drought stress. (XLS 38 kb) Additional file 8: Targets of miRNAs identified by degradome sequence. (XLS 46 kb) Additional file 9: Target plots of identified miRNA targets using degradome sequencing. (PDF 762 kb) Additional file 10: Targets of novel miRNAs identified by degradome sequencing. (DOC 57 kb) Additional file 11: Targets of known miRNAs predicted by the web tool psRNATarget. (XLS 69 kb) Additional file 12: Targets of novel miRNAs predicted by the web tool psRNATarget. (XLS 187 kb) Additional file 13: miRNA comparison data among different foxtail millet varieties. (XLSX 17 kb)

## Abbreviations

miRNA: microRNA
sRNA: small RNA
siRNA: small interfering RNA
ta-siRNAs: trans-acting small interfering RNA
WUE: water use efficiency
pre-miRNA: precursors of microRNA
LWP: leaf water potential
TPM: Transcript per million
DT: drought-treated group
CL: control group
MFEs: minimum free energies
DE: differentially expressed
