# Supplementary figures and images for: Combined small RNA and degradome sequencing to identify miRNAs and their targets in response to drought in foxtail millet
Source: BMC Genet. 2016 Apr 12;17:57. doi: 10.1186/s12863-016-0364-7 (PMC4828802; doi:10.1186/s12863-016-0364-7)

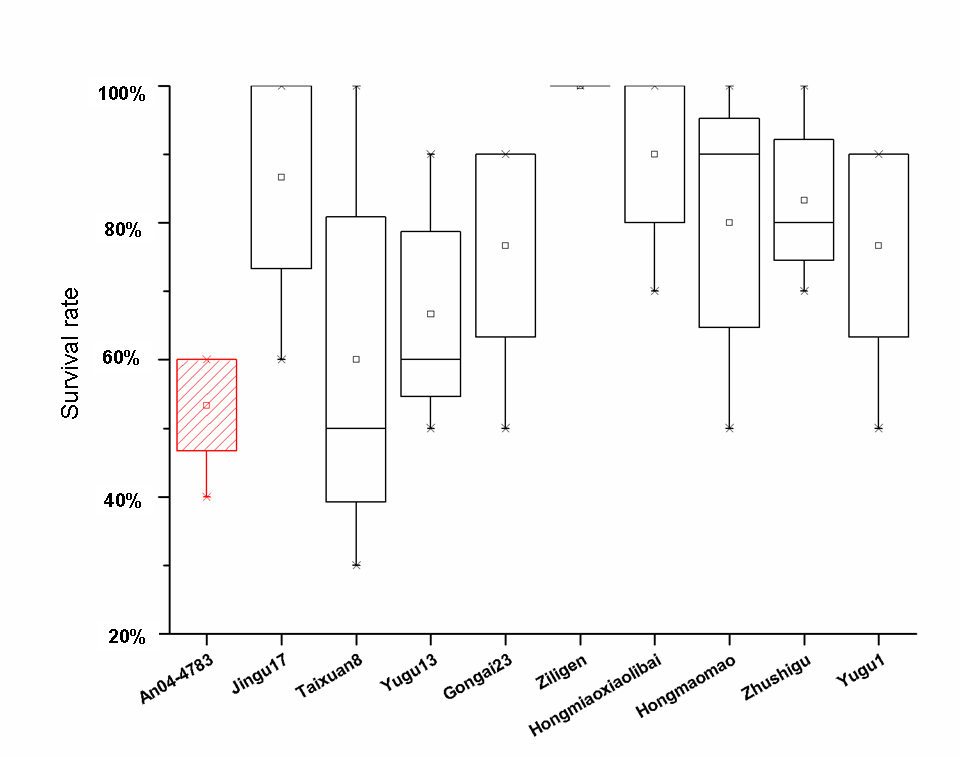

Supplement: Additional file 1: — The survival rate of 10 varieties of foxtail millet under repeated drought treatments. (TIF 166 kb) [file 12863_2016_364_MOESM1_ESM.tif]
