# Supplementary material for: Combined small RNA and degradome sequencing to identify miRNAs and their targets in response to drought in foxtail millet
Source: BMC Genet. 2016 Apr 12;17:57. doi: 10.1186/s12863-016-0364-7 (PMC4828802; doi:10.1186/s12863-016-0364-7)
Supplement: Additional file 2: — Primers used for RT-PCR in this study. (DOC 35 kb) [file 12863_2016_364_MOESM2_ESM.doc]

**Additional file 2:** Primers used in this study

| **miRNA** | **RT primer** | **Forward primer** |
| --- | --- | --- |
| **sit-miR159b** | GTCGTATCCAGTGCAGGGTCCGAGGTATTCGCACTGGATACGACGGAGCT | CGGGGTTGGATTGAAGGG |
| **sit-miR167b** | GTCGTATCCAGTGCAGGGTCCGAGGTATTCGCACTGGATACGACTCAGAT | CGGGCTGAAGCTGCCAG |
| **sit-miR390** | GTCGTATCCAGTGCAGGGTCCGAGGTATTCGCACTGGATACGACGGCGCT | GGGCAAGCTCAGGAGGGATA |
| **sit-miR394** | GTCGTATCCAGTGCAGGGTCCGAGGTATTCGCACTGGATACGACGGAGGT | AAGGTGGTTGGCATTCTGTCC |
| **sit-miR396a** | GTCGTATCCAGTGCAGGGTCCGAGGTATTCGCACTGGATACGACCAGTTC | CGGCTCCACAGGCTTTCT |
| **sit-miR408** | GTCGTATCCAGTGCAGGGTCCGAGGTATTCGCACTGGATACGACGCCAGG | CGCAGGGTCCGAGGTATTC |
| **sit-novel-15** | GTCGTATCCAGTGCAGGGTCCGAGGTATTCGCACTGGATACGACACCTGG | CGGGCACACTATAGGAGCTGG |
| **sit-novel-18** | GTCGTATCCAGTGCAGGGTCCGAGGTATTCGCACTGGATACGACAGTGAT | TGCCCGTGAGCCGAGCC |
| **sit-novel-53** | GTCGTATCCAGTGCAGGGTCCGAGGTATTCGCACTGGATACGACGACCTA | TGCCCGCTCAGTTCCAAA |
| **sit-novel-56** | GTCGTATCCAGTGCAGGGTCCGAGGTATTCGCACTGGATACGACGTGCTC | CGGGTTGACAGAAGAGAGCG |
| **U6** | CGCAGGGTCCGAGGTATTCTTTGGACCATTTCTCGAT | GGAACGATACAGAGAAGATTAGCA |
| **Universal Reverse** | CGCAGGGTCCGAGGTATTC | |
