# Supplementary material for: Combined small RNA and degradome sequencing to identify miRNAs and their targets in response to drought in foxtail millet
Source: BMC Genet. 2016 Apr 12;17:57. doi: 10.1186/s12863-016-0364-7 (PMC4828802; doi:10.1186/s12863-016-0364-7)
Supplement: Additional file 5: — The secondary structures of novel miRNA precursors (PDF 324 kb) [file 12863_2016_364_MOESM5_ESM.pdf]

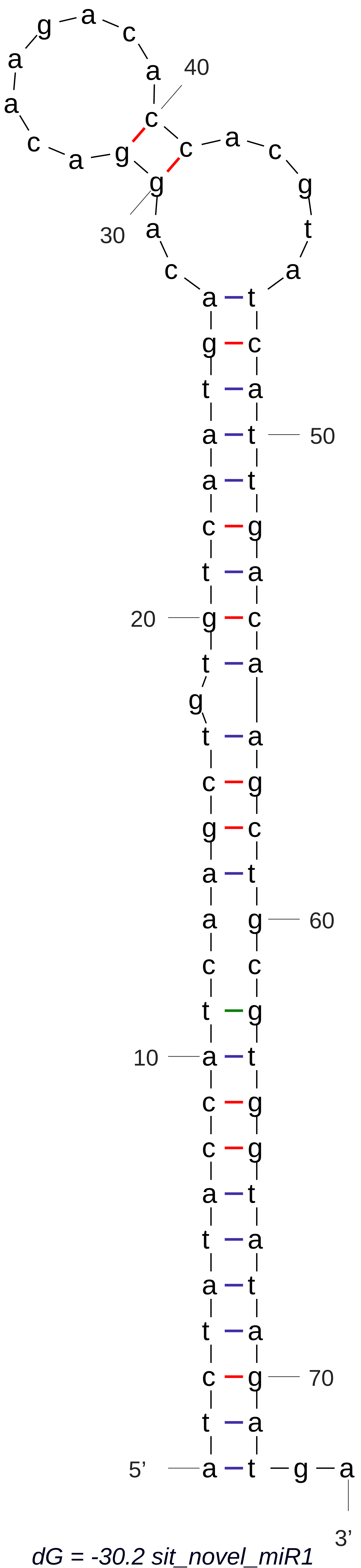

dG = -30.2 sit_novel_miR1

Output of sir_graph (©)
mfold_util 4.7

Created Tue Feb 23 05:29:48 2016

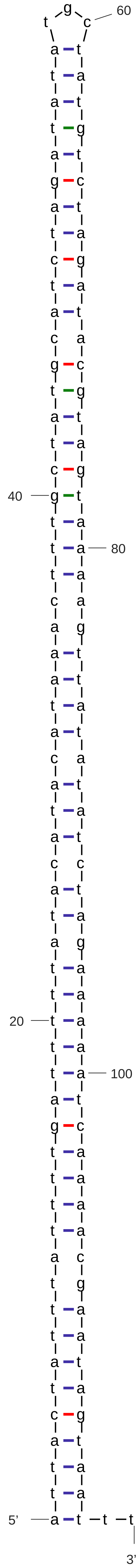

dG = -43.6 sit_novel_miR2

Output of sir_graph (©)
mfold_util 4.7

Created Tue Feb 23 05:30:02 2016

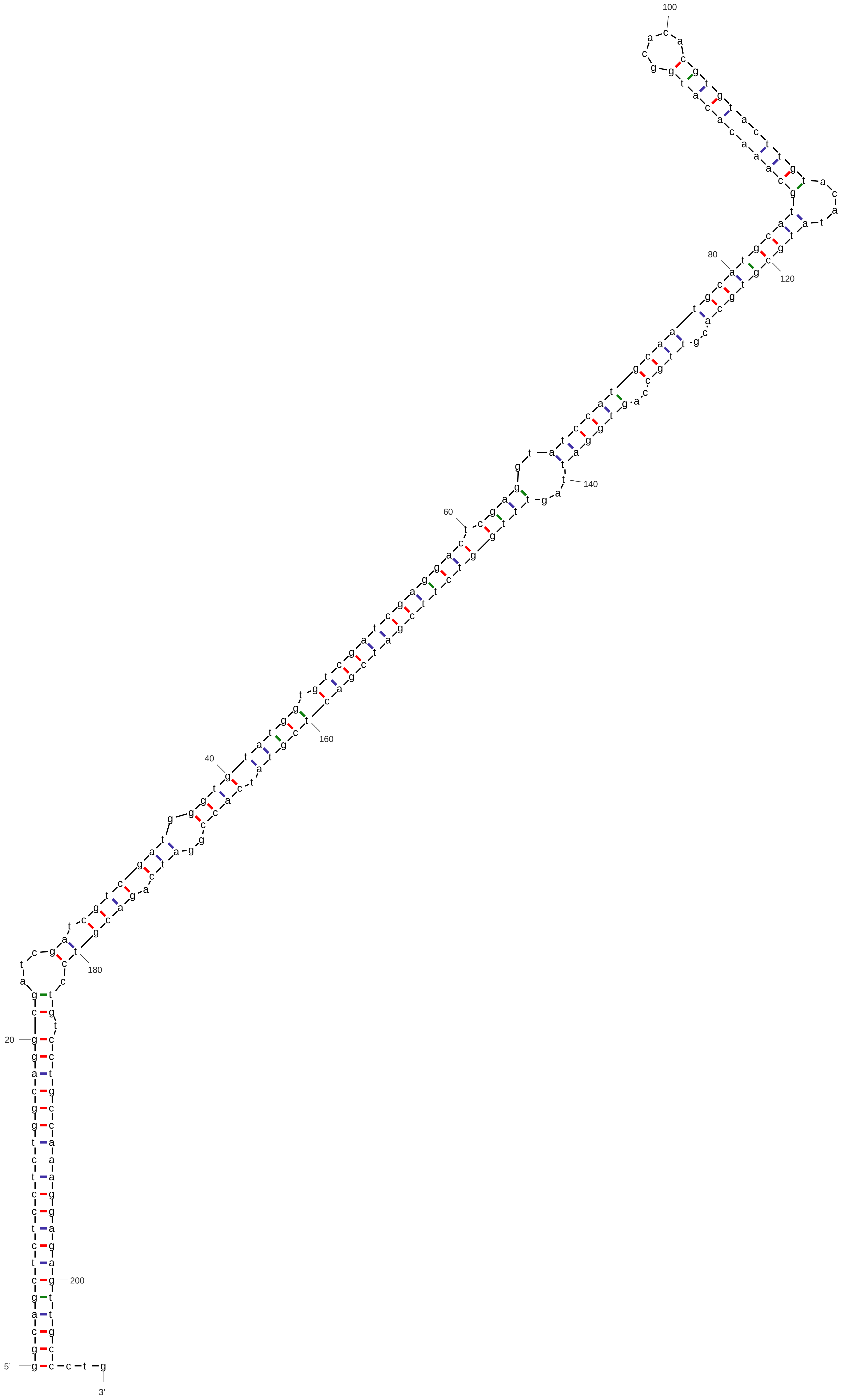

dG = -109.7 sit_novel_miR3

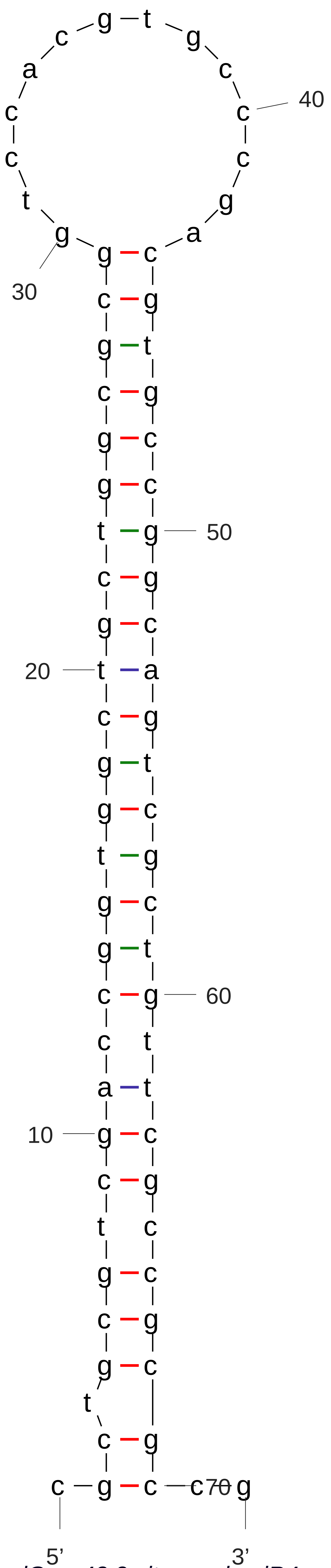

dG = -43.2 sit_novel_miR4

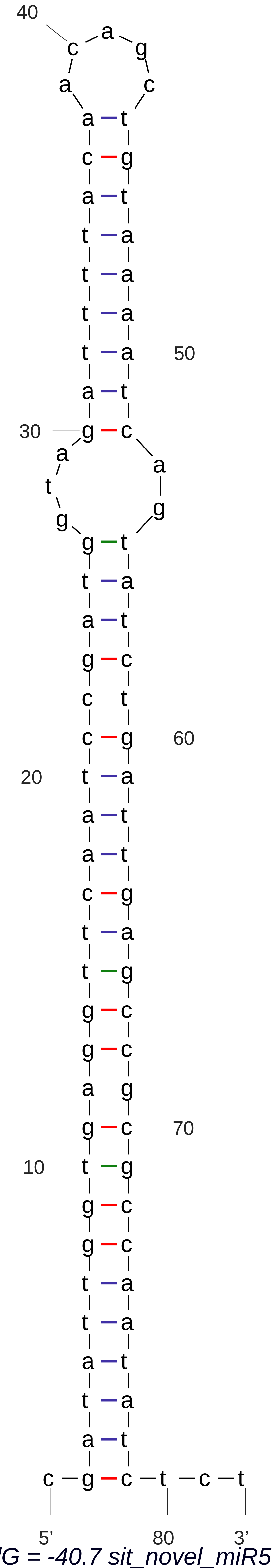

dG = -40.7 sit_novel_miR5

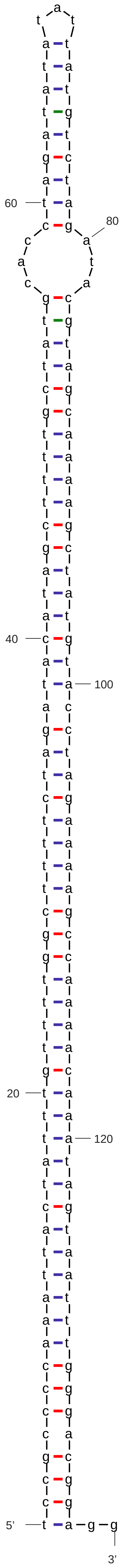

dG = -92.2 sit_novel_miR6

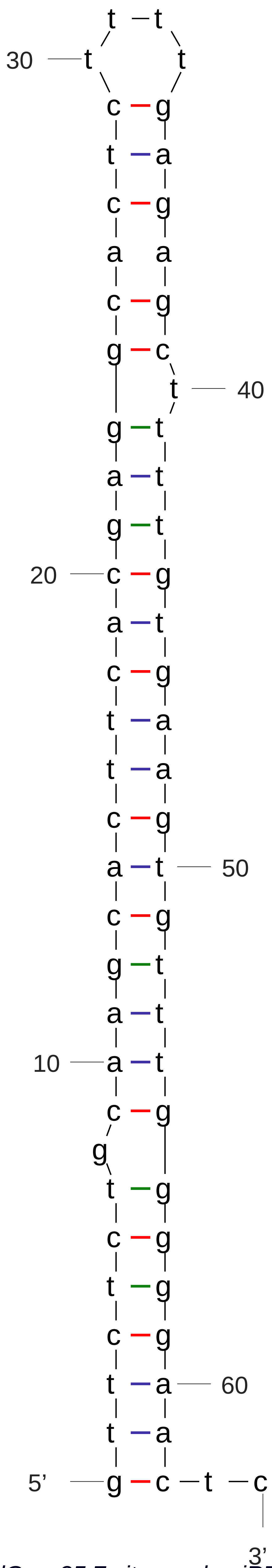

dG = -35.7 sit_novel_miR7

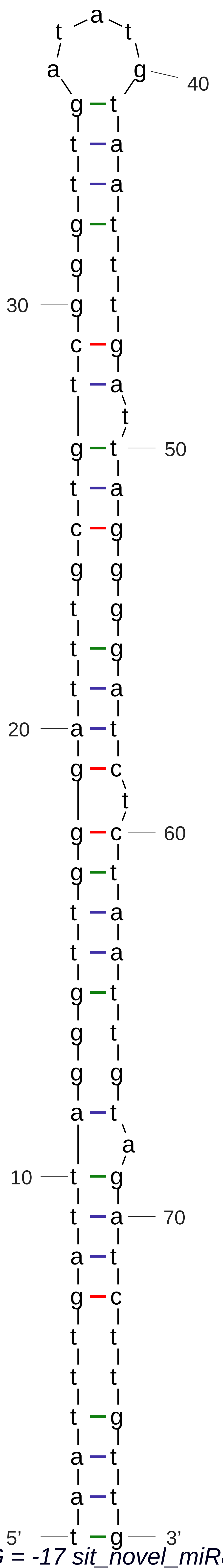

dG = -17 sit_novel_miR8

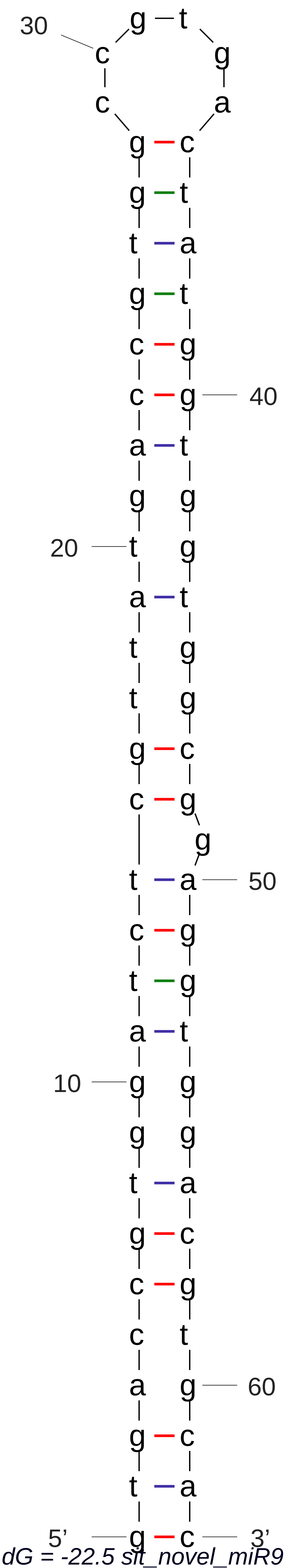

dG = -22.5 sit_novel_miR9

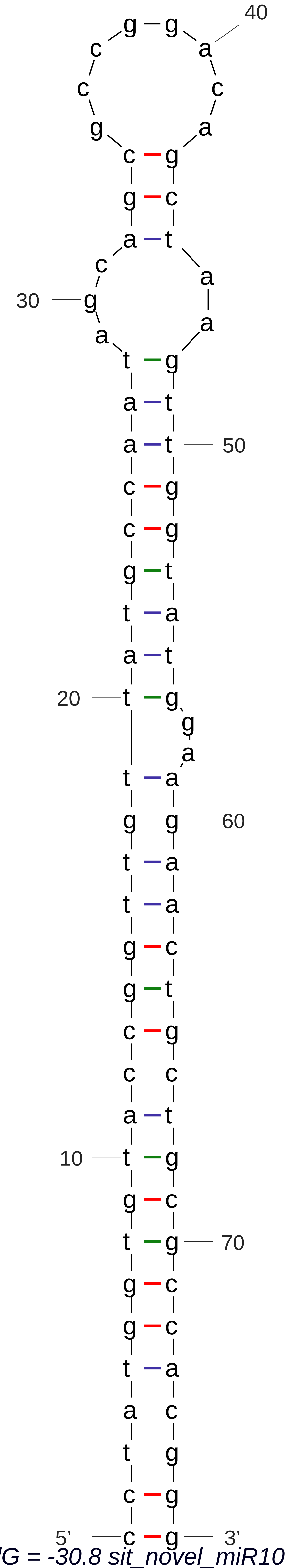

dG = -30.8 sit_novel_miR10

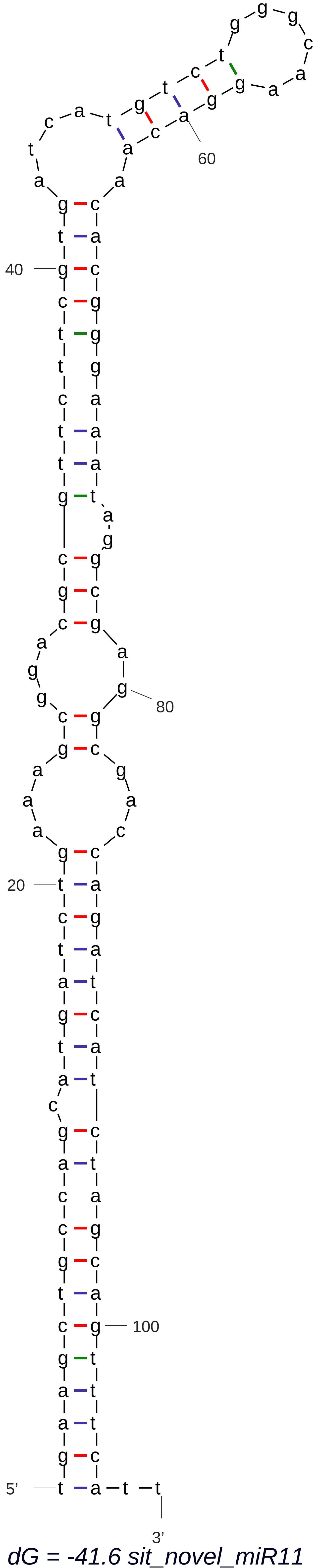

dG = -41.6 sit_novel_miR11

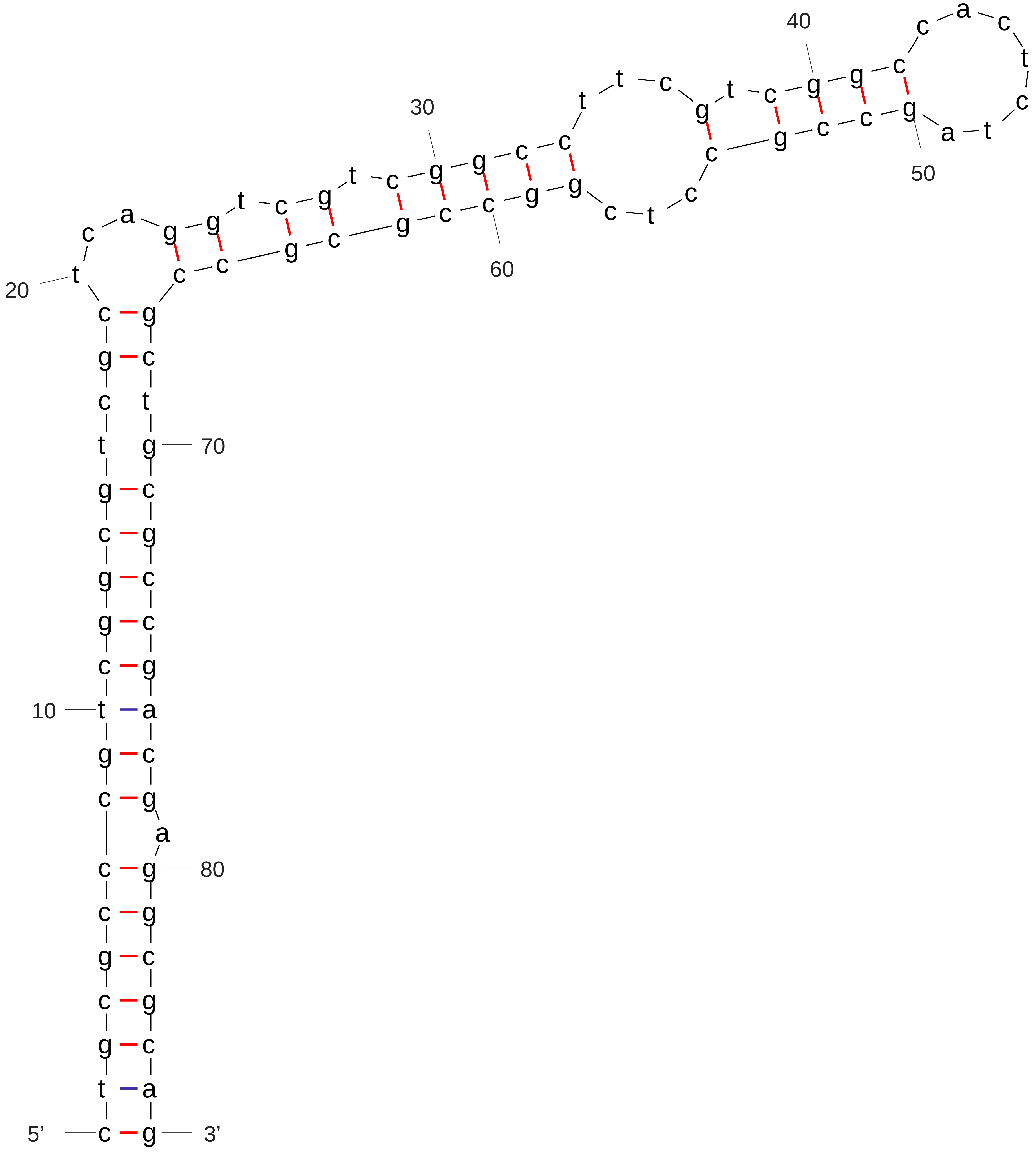

dG = -54.7 sit_novel_miR12

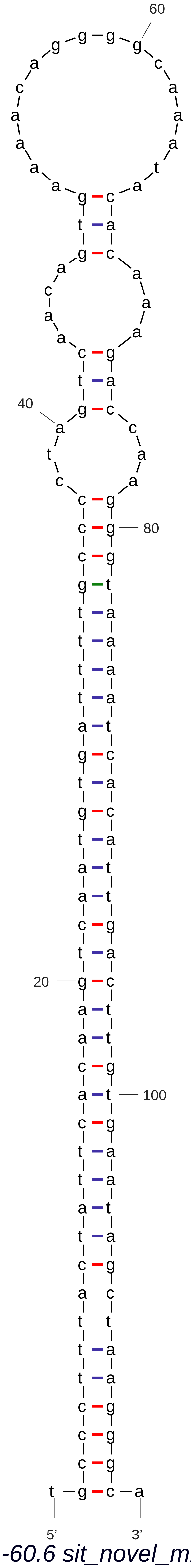

dG = -60.6 sit_novel_miR13

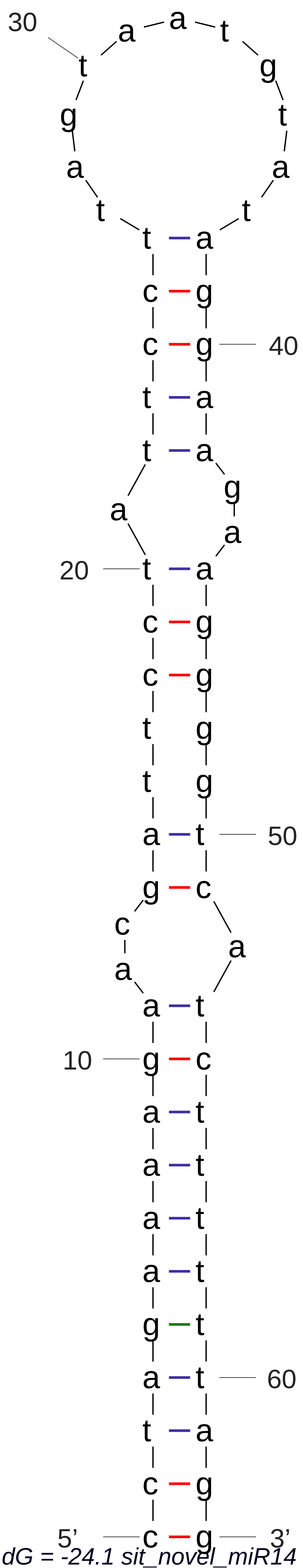

dG = -24.1 sit_novel_miR14

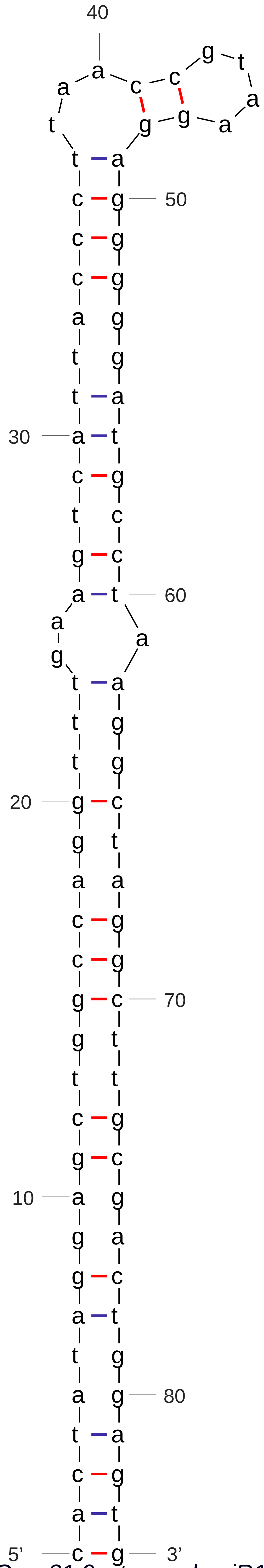

dG = -31.9 sit_novel_miR15

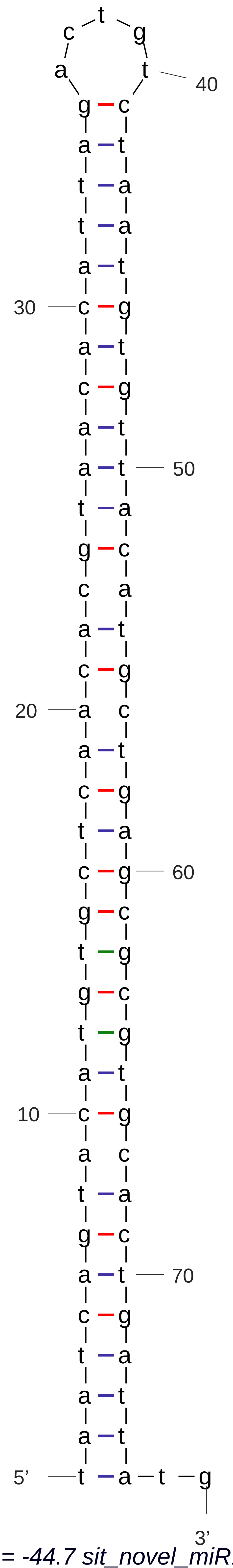

dG = -44.7 sit_novel_miR16

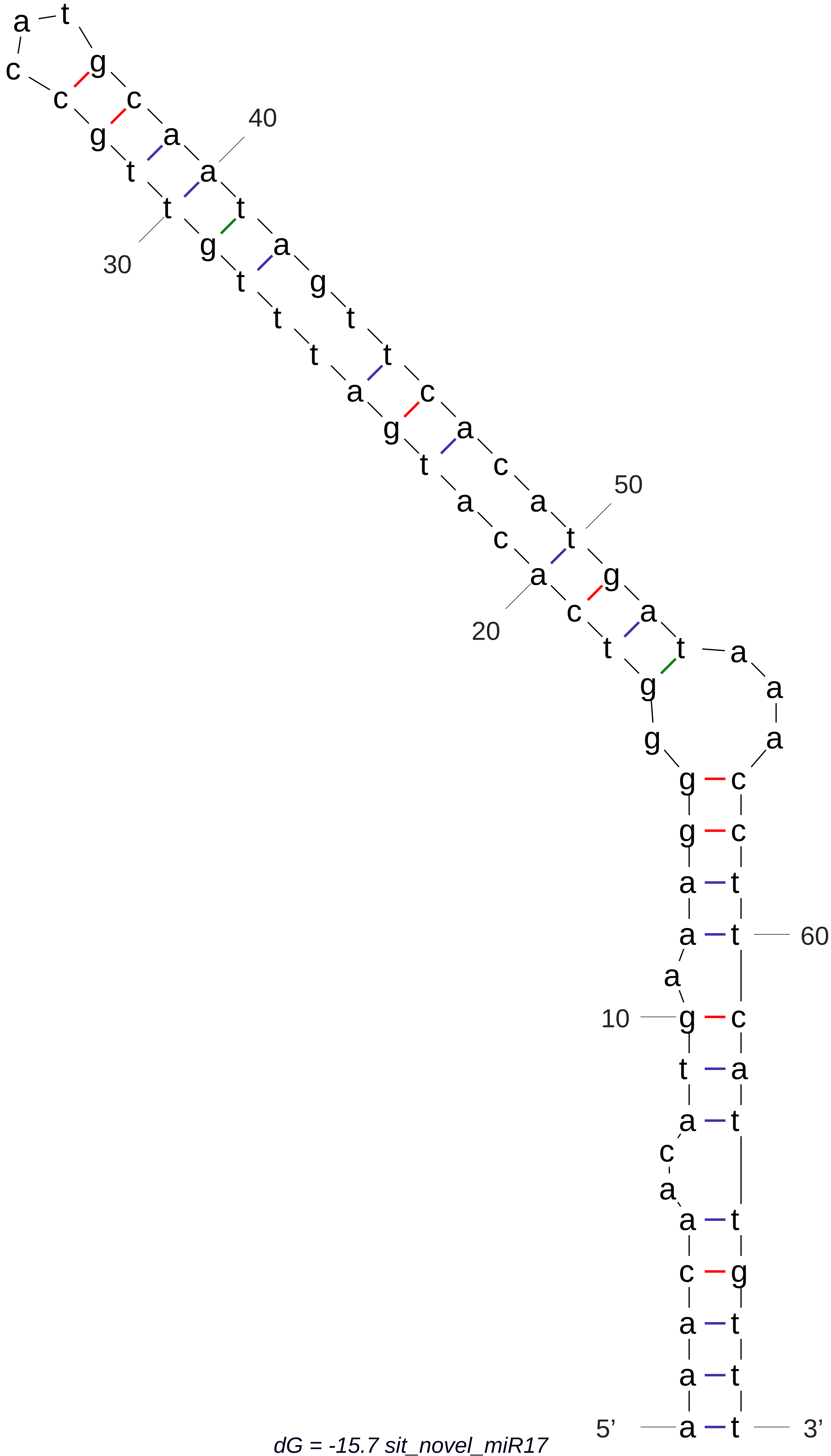

dG = -15.7 sit_novel_miR17

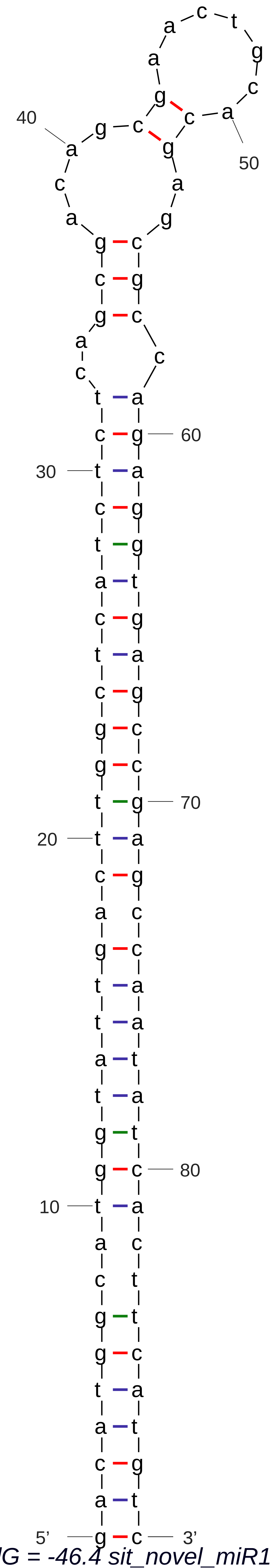

dG = -46.4 sit_novel_miR18

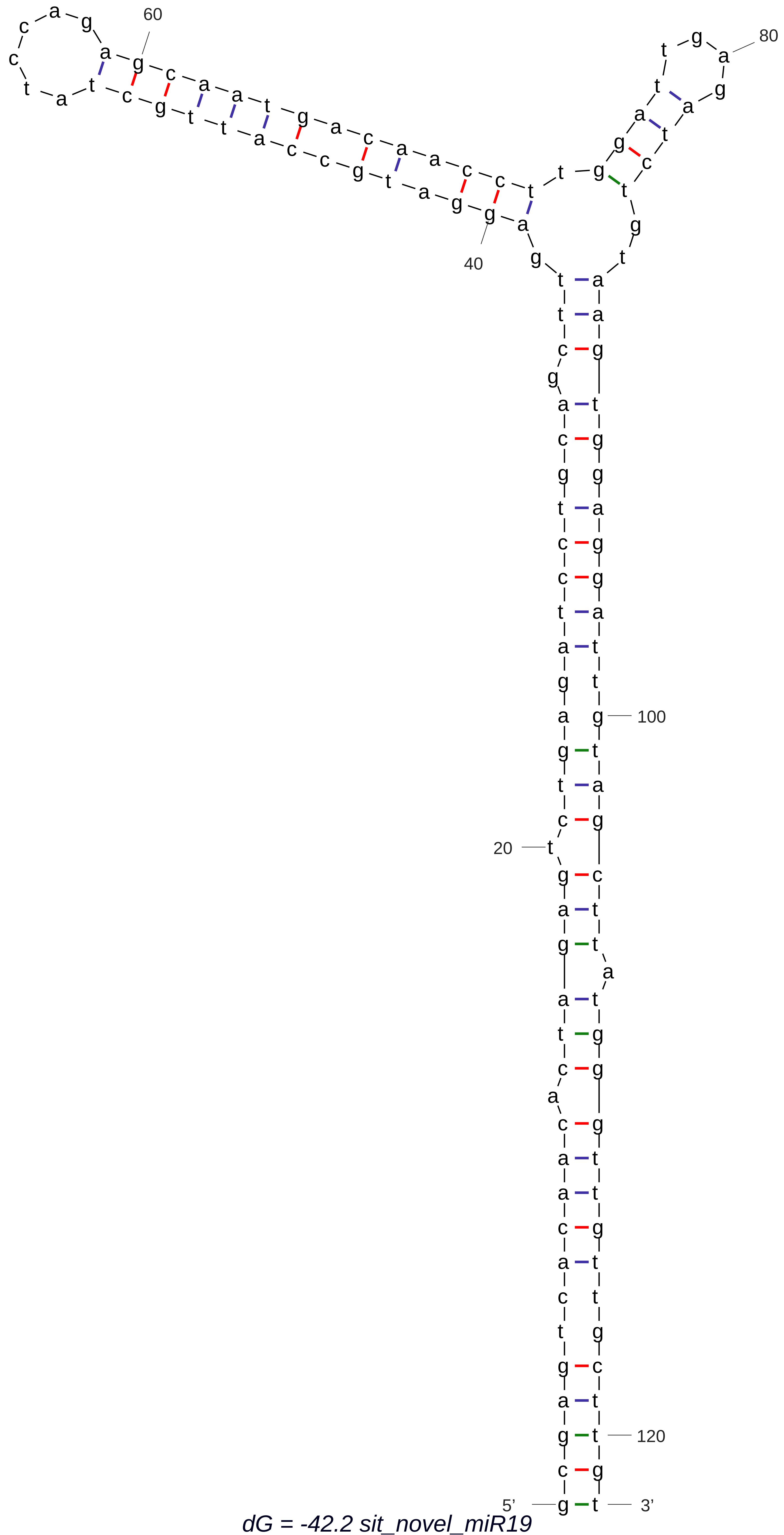

dG = -42.2 sit_novel_miR19

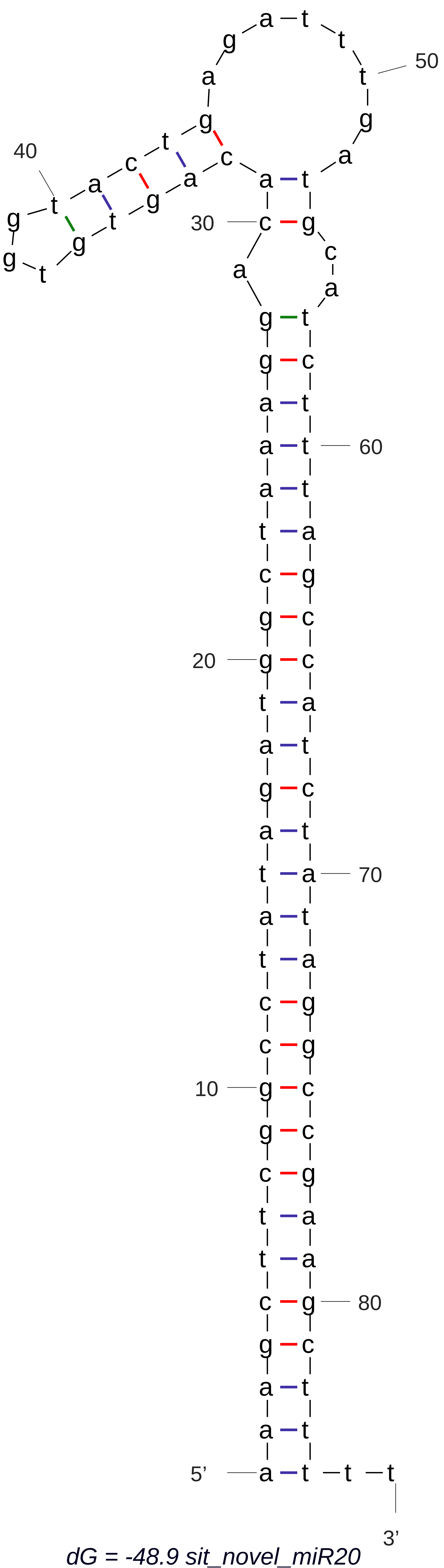

dG = -48.9 sit_novel_miR20

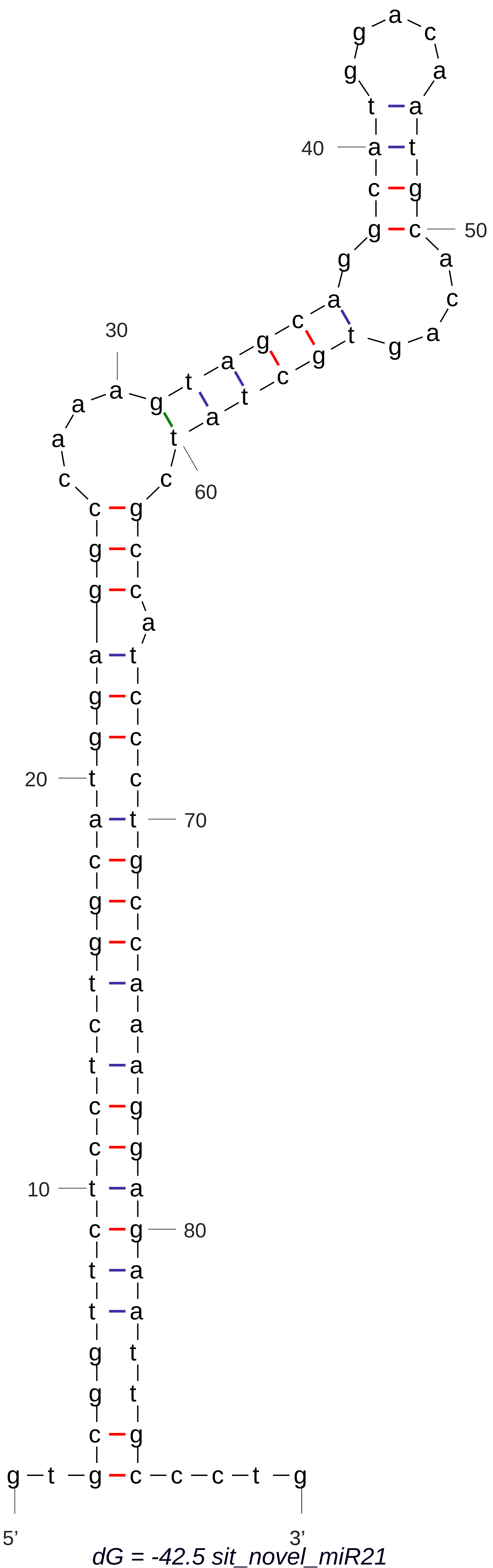

dG = -42.5 sit_novel_miR21

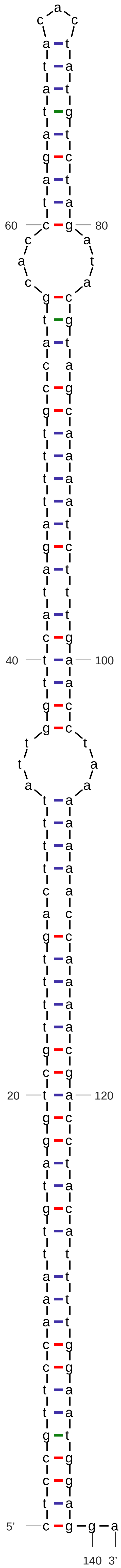

dG = -74.6 sit_novel_miR22

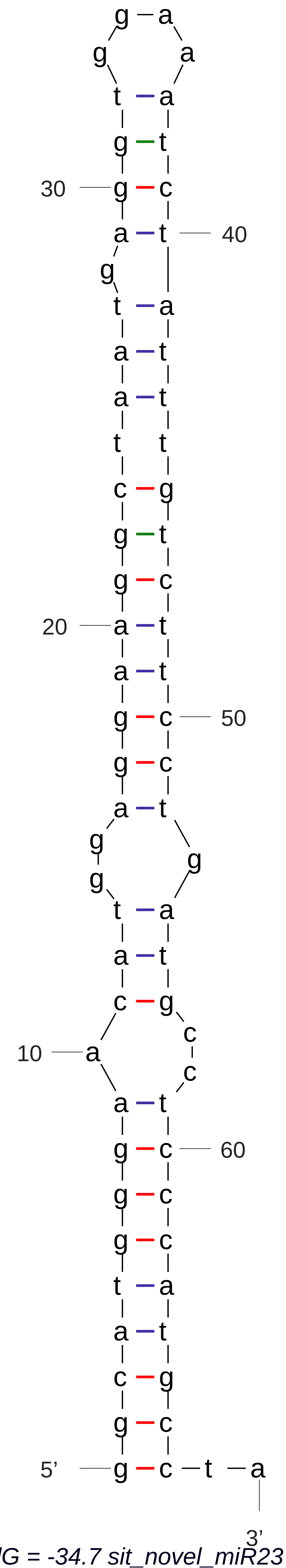

dG = -34.7 sit_novel_miR23

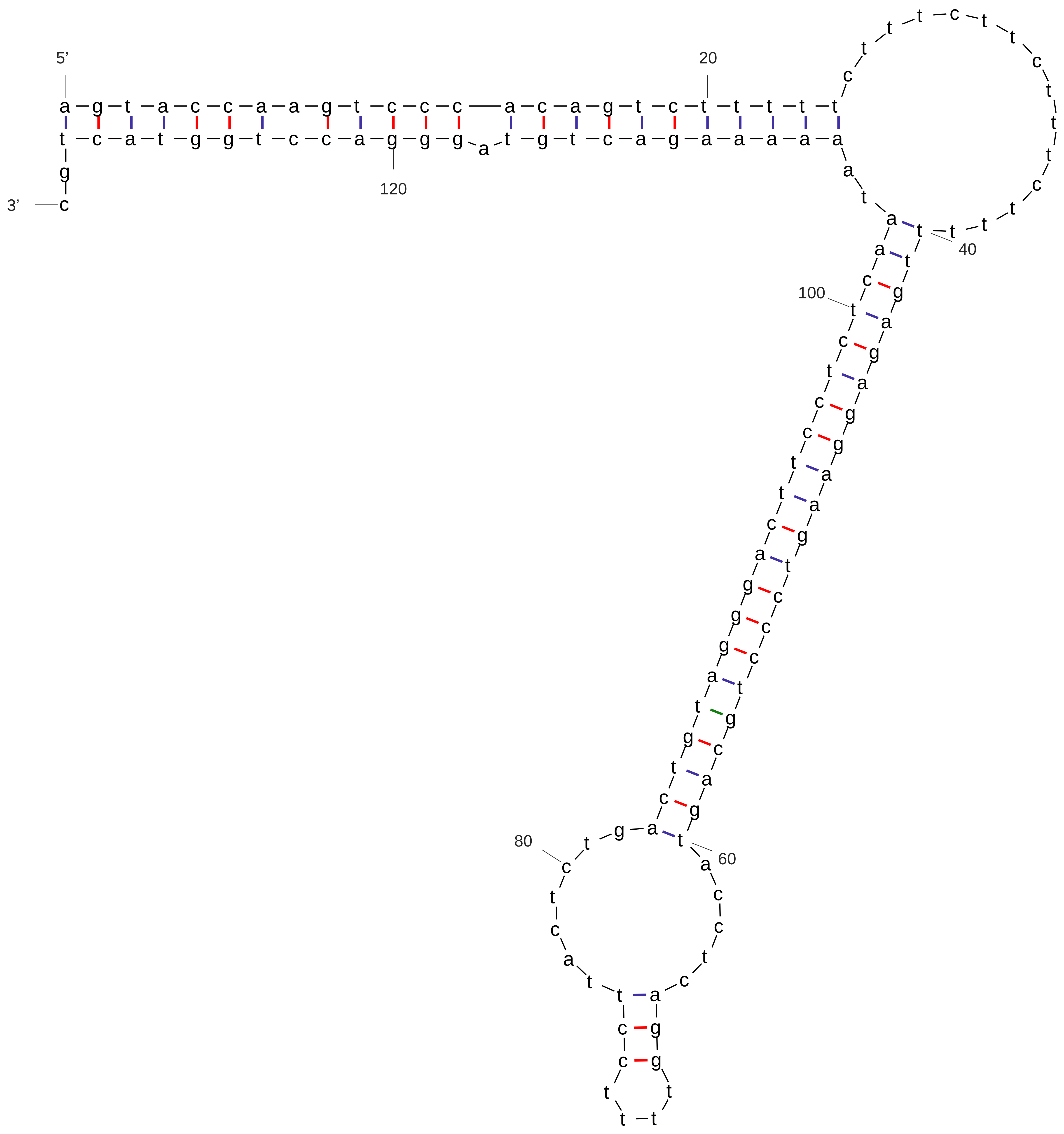

dG = -72.9 sit_novel_miR24

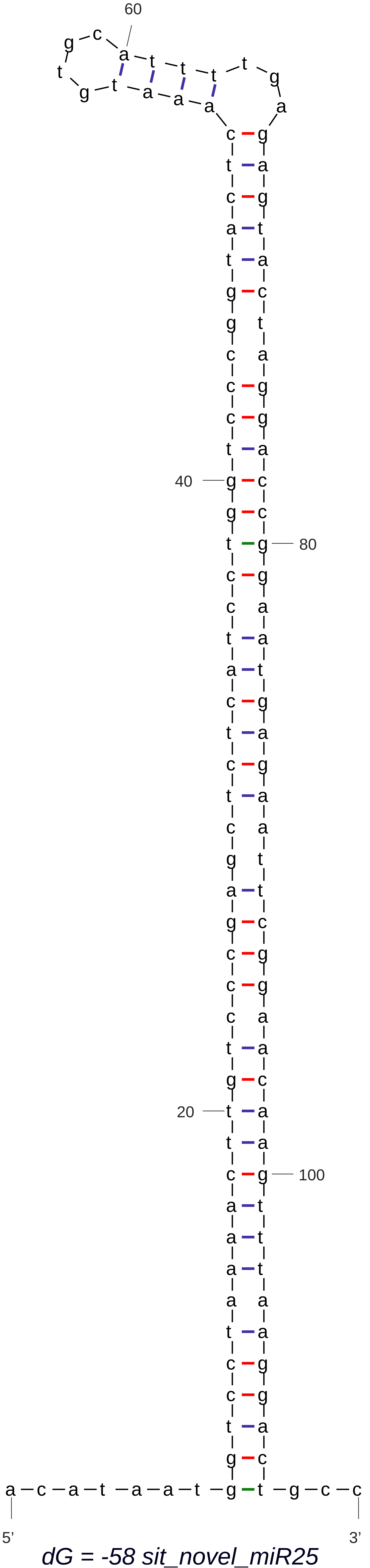

dG = -58 sit_novel_miR25

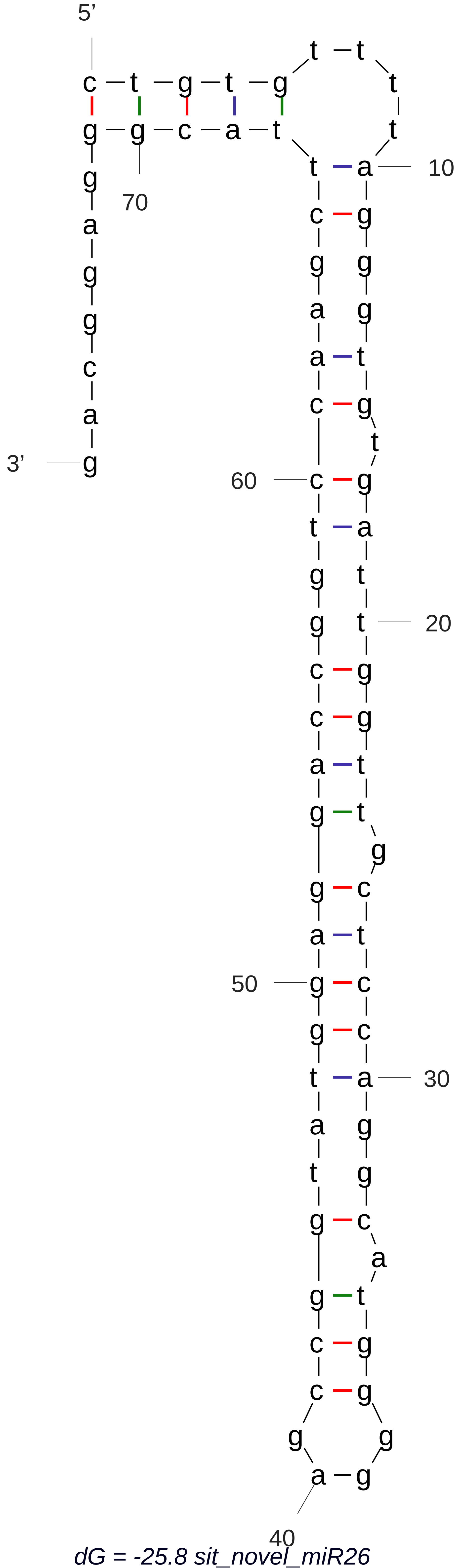

dG = -25.8 sit_novel_miR26

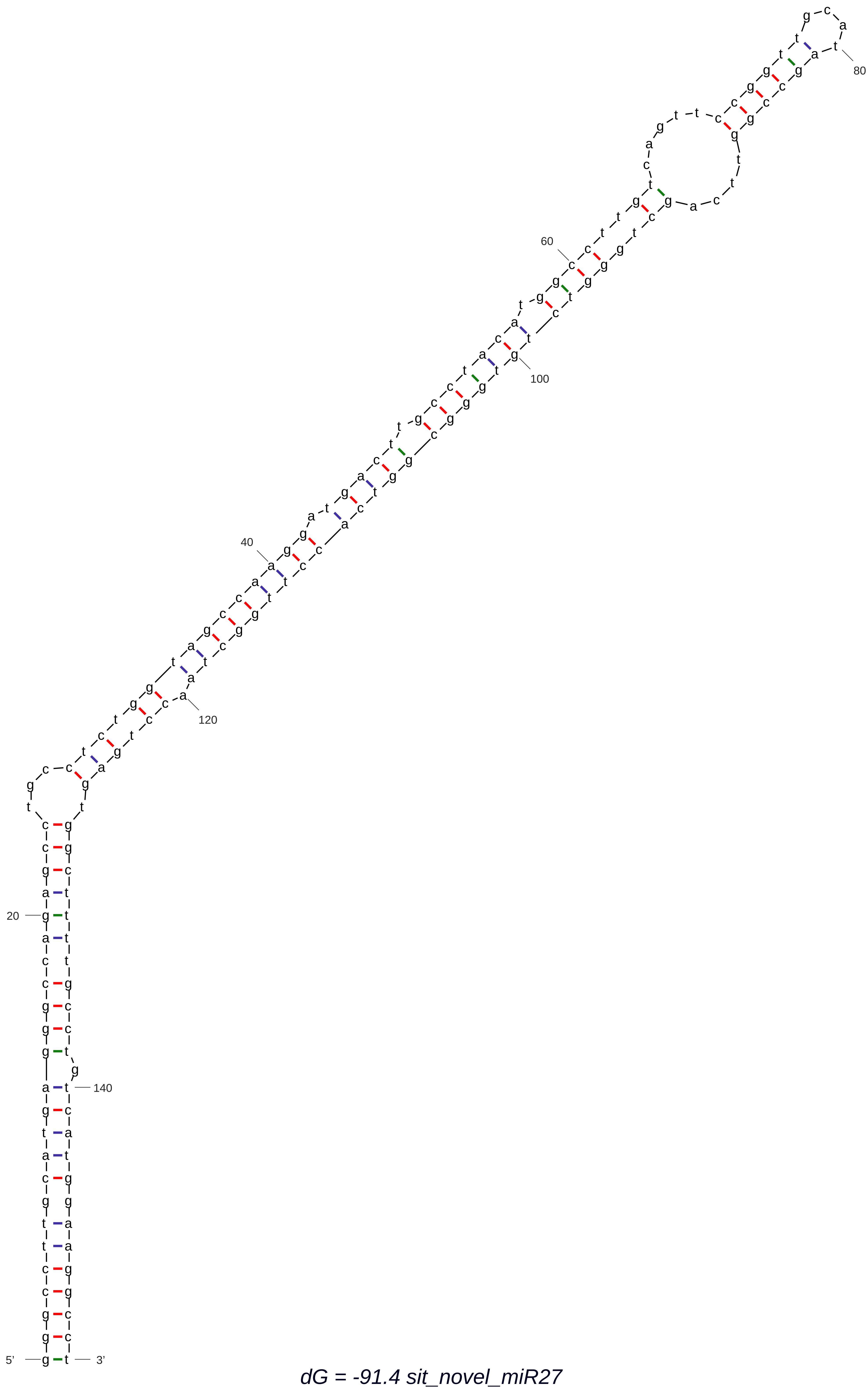

dG = -91.4 sit_novel_miR27

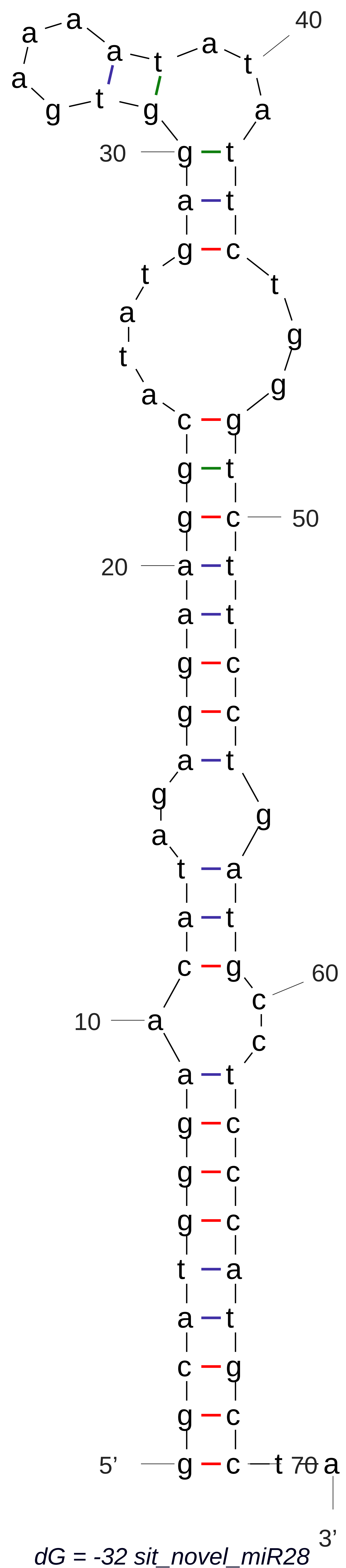

dG = -32 sit_novel_miR28

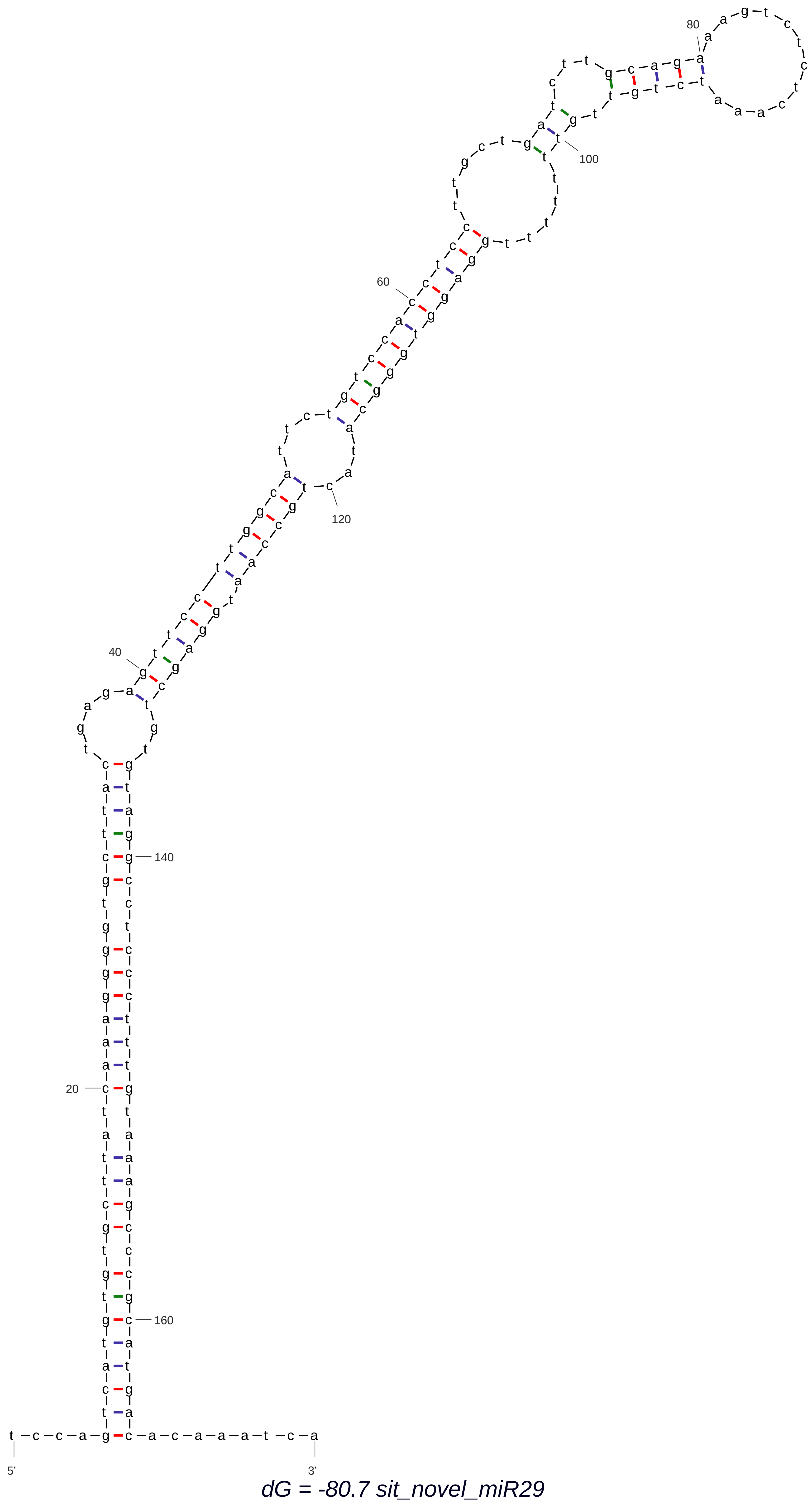

dG = -80.7 sit_novel_miR29

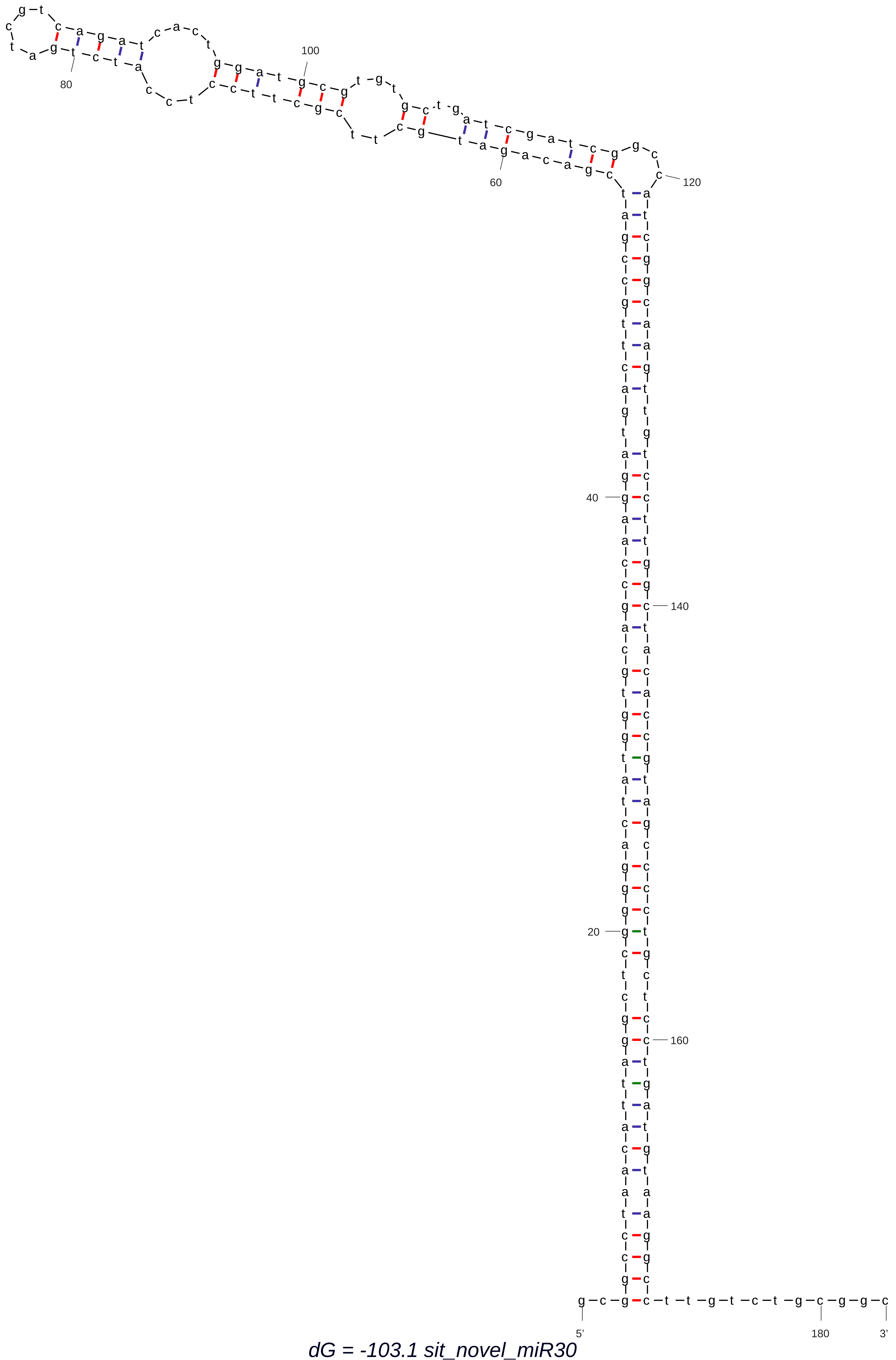

dG = -103.1 sit_novel_miR30

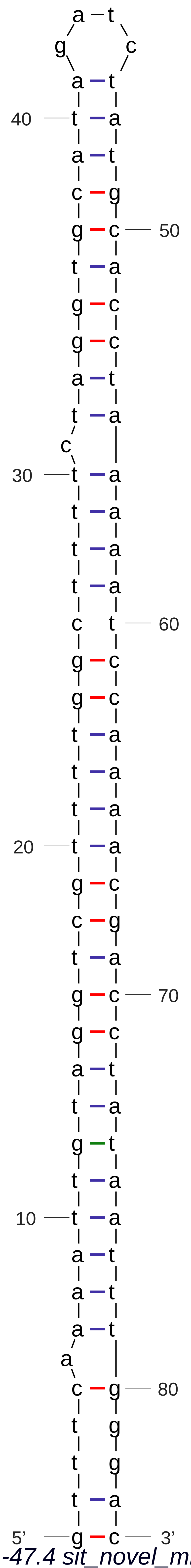

dG = -47.4 sit_novel_miR31

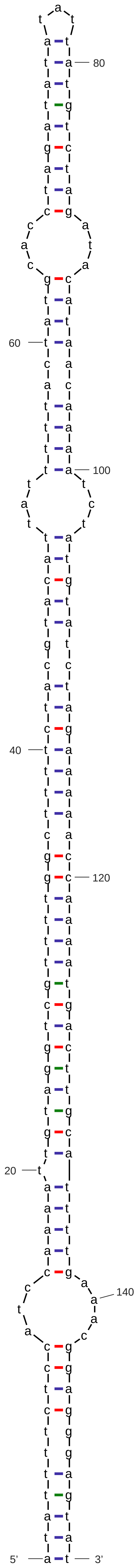

dG = -58.6 sit_novel_miR32

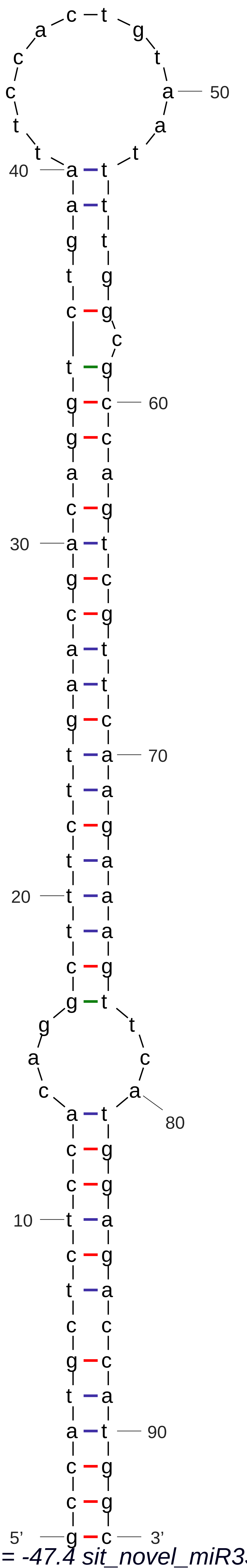

dG = -47.4 sit_novel_miR33

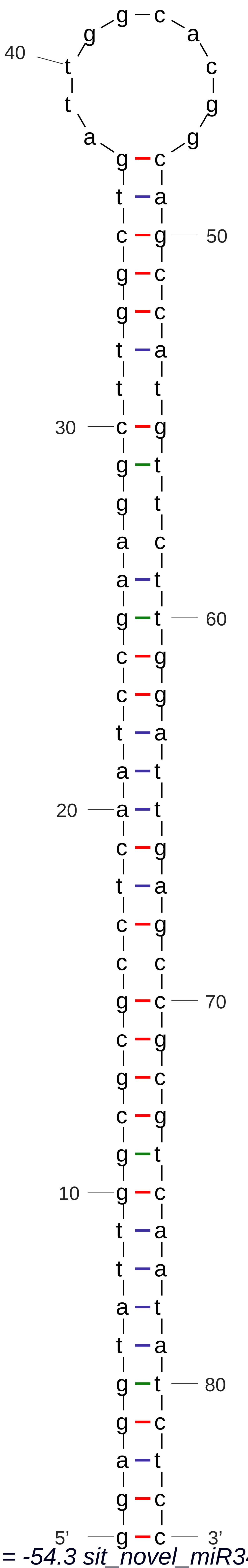

dG = -54.3 sit_novel_miR34

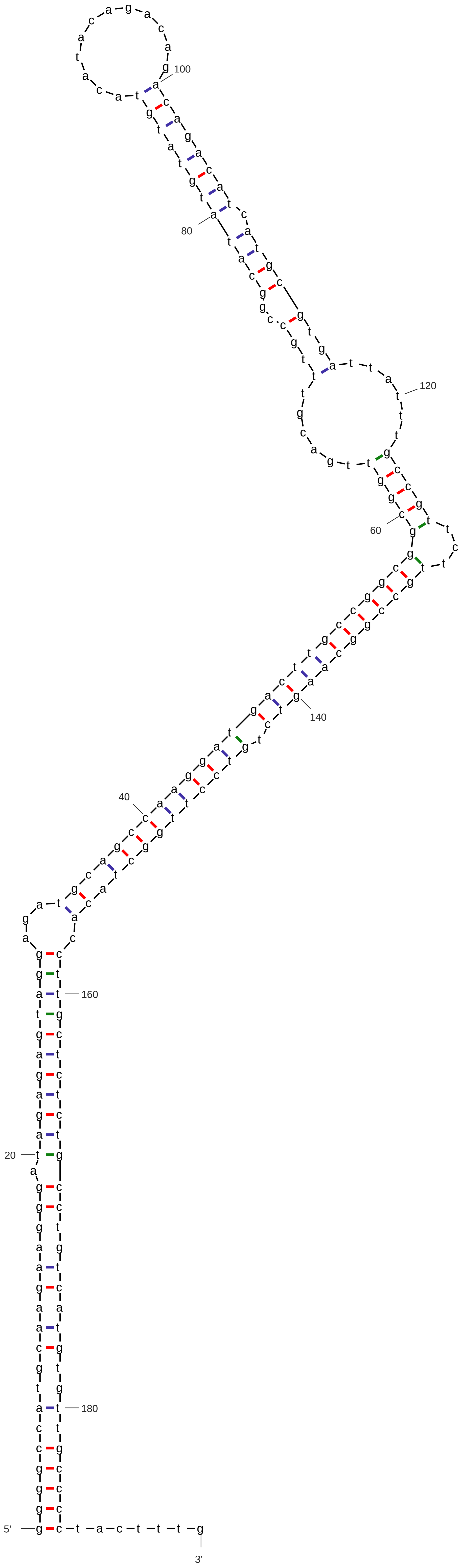

dG = -94.7 sit_novel_miR35

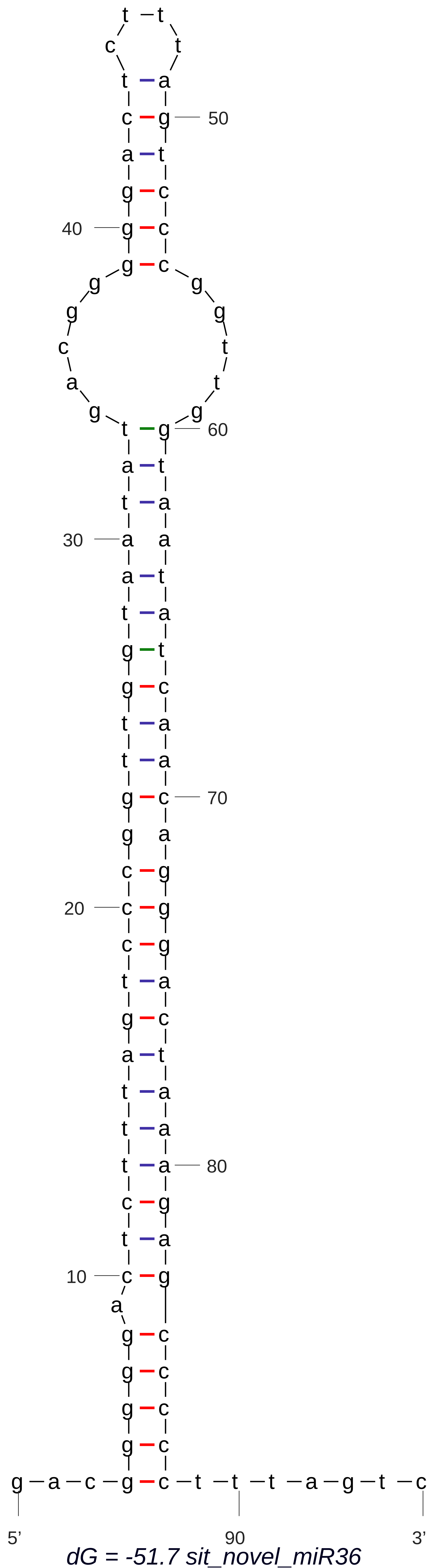

dG = -51.7 sit_novel_miR36

Output of sir_graph (©)
mfold_util 4.7

Created Tue Feb 23 05:51:30 2016

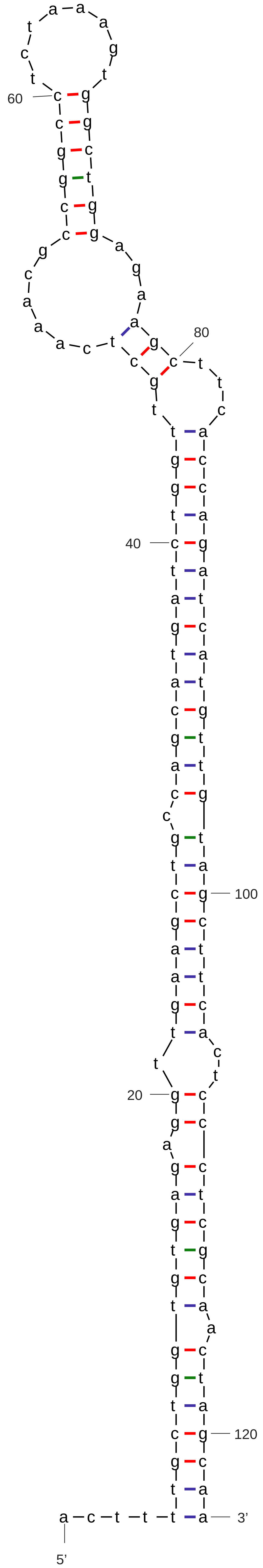

dG = -68.2 sit_novel_miR37

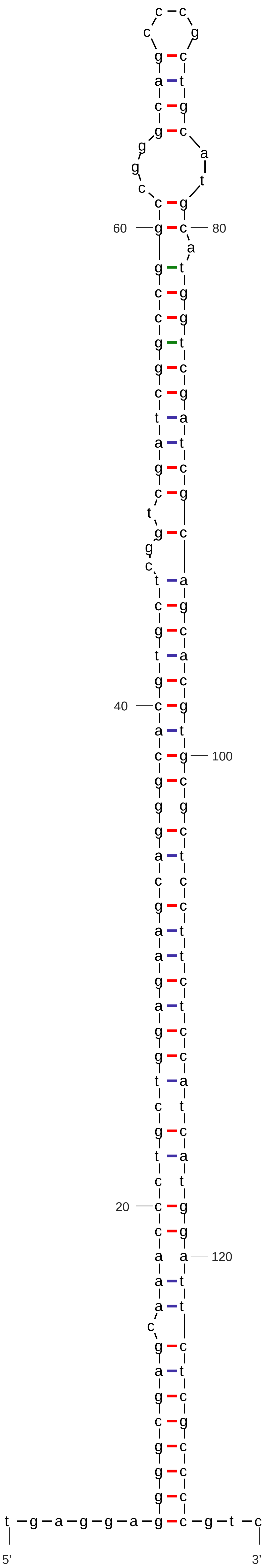

dG = -82 sit_novel_miR38

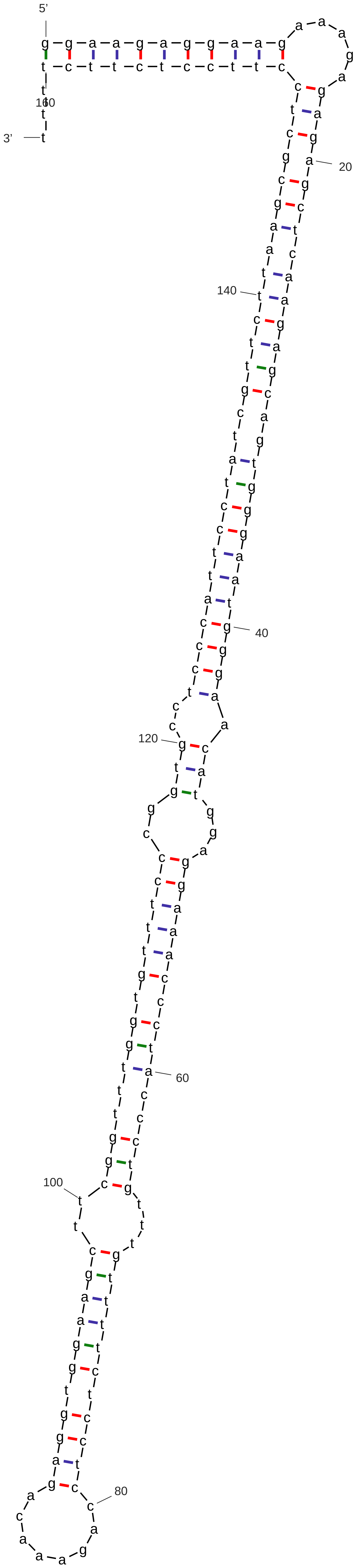

dG = -77.7 sit_novel_miR39

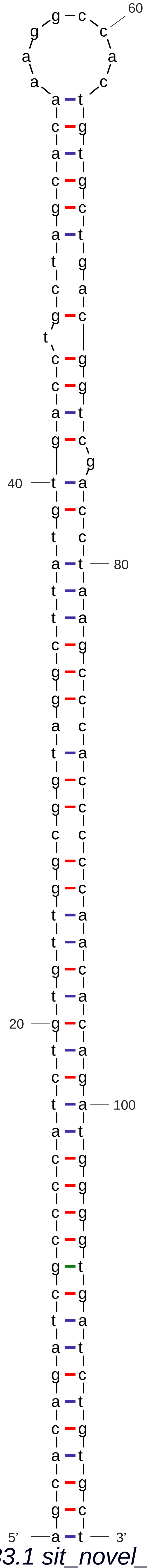

dG = -83.1 sit_novel_miR40

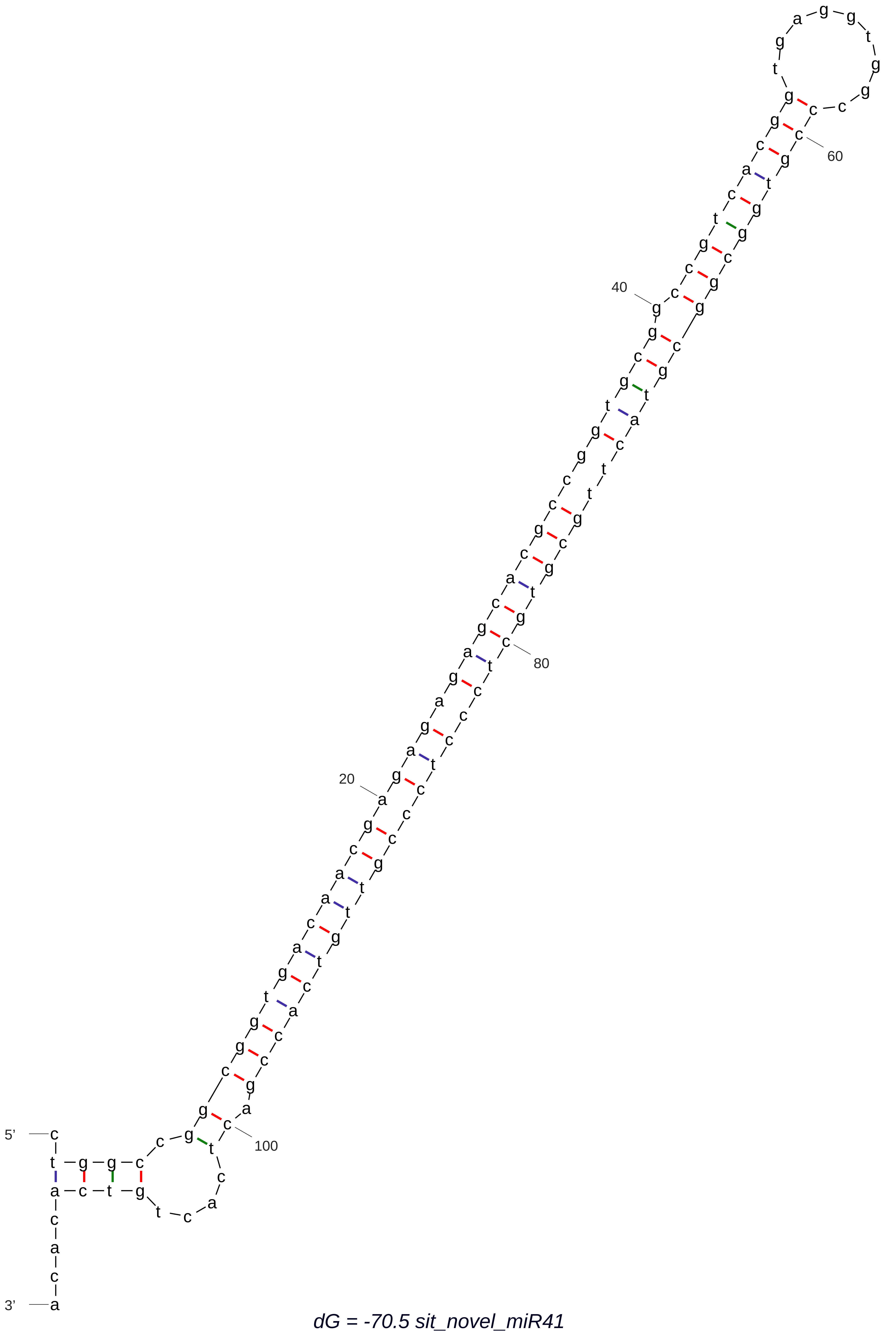

dG = -70.5 sit_novel_miR41

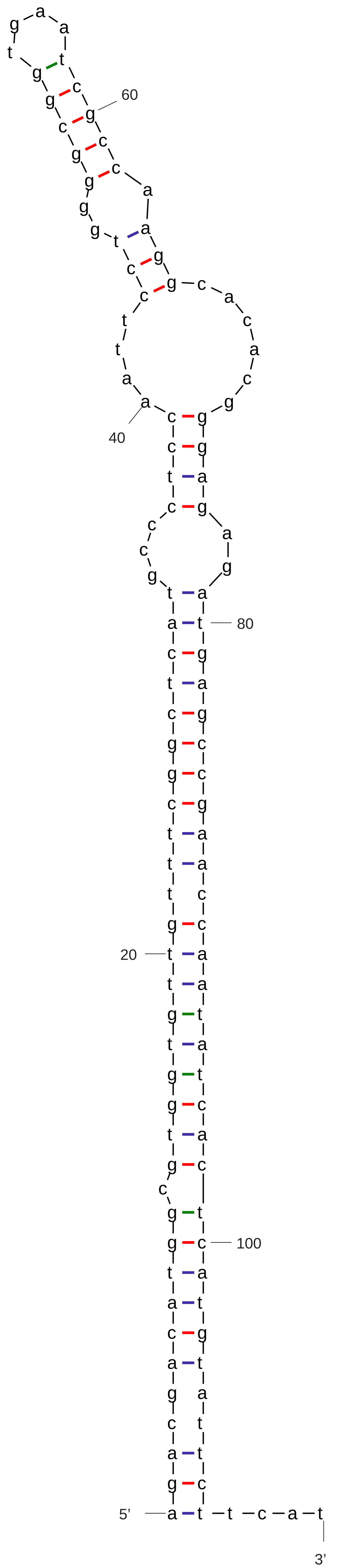

dG = -52.5 sit_novel_miR42

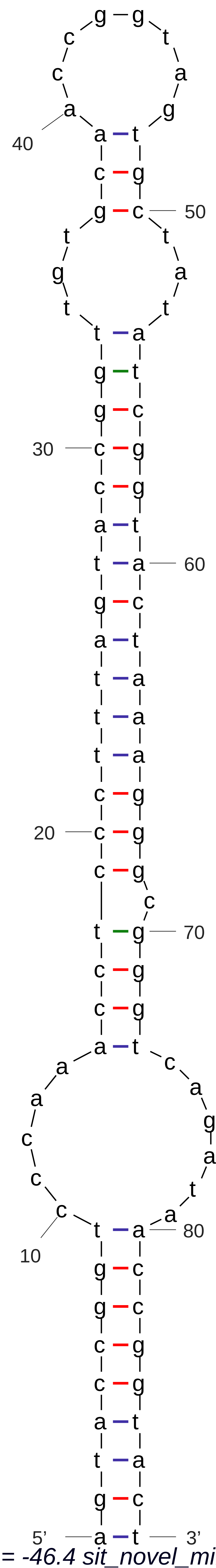

dG = -46.4 sit_novel_miR43

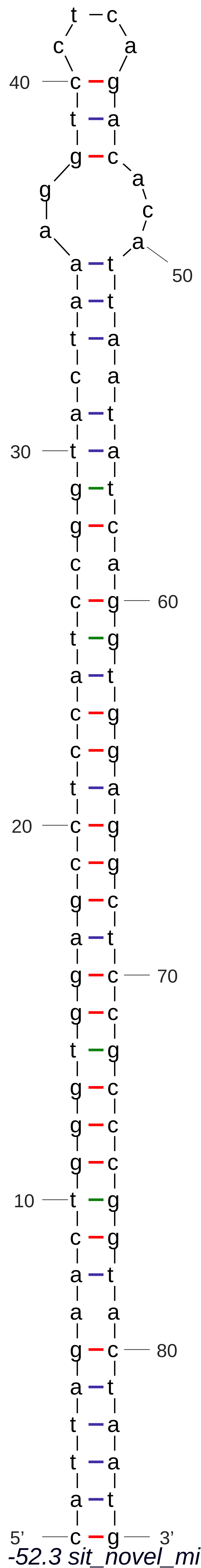

dG = -52.3 sit_novel_miR44

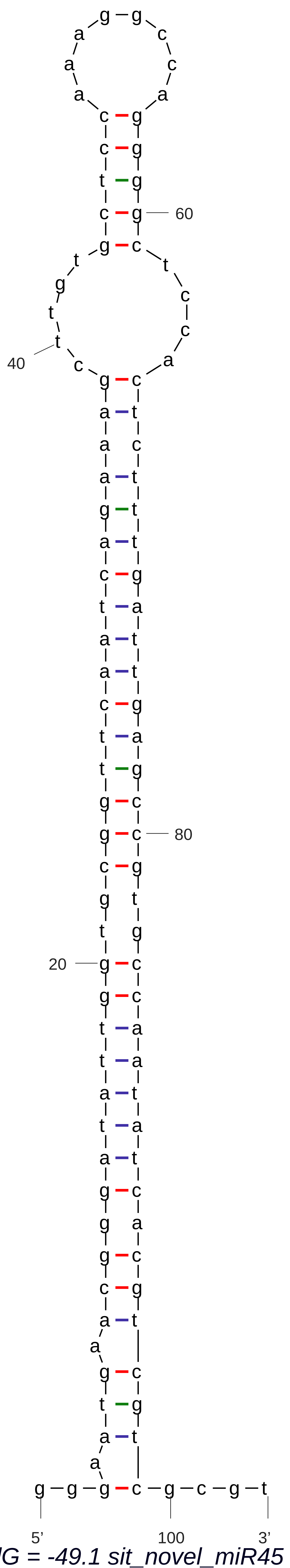

dG = -49.1 sit_novel_miR45

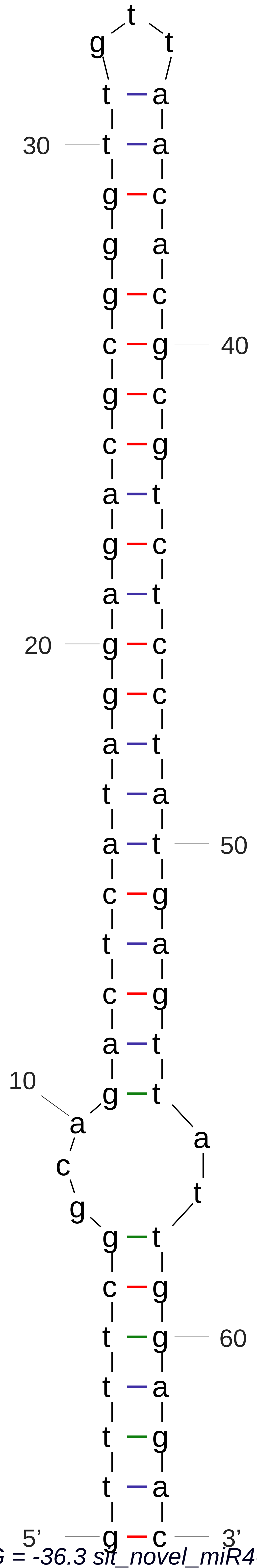

dG = -36.3 sit_novel_miR46

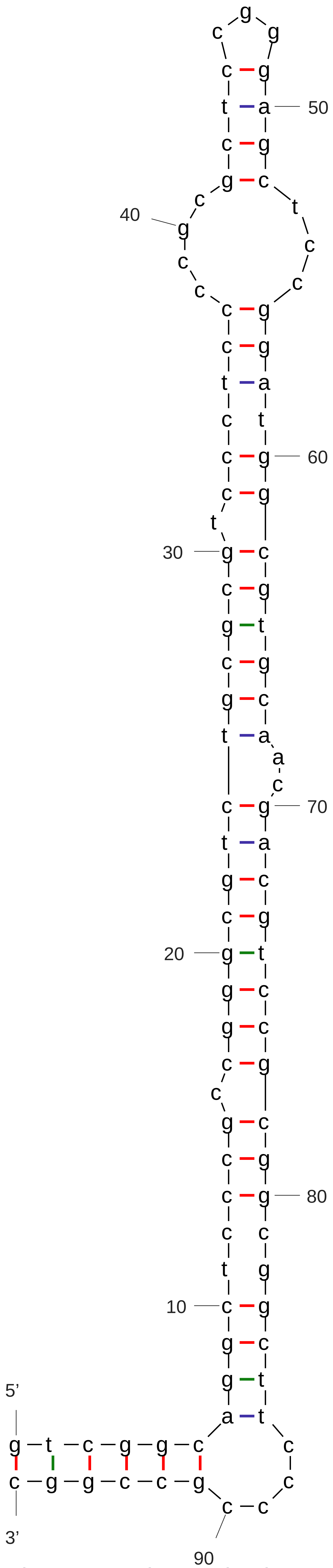

dG = -53.2 sit_novel_miR47

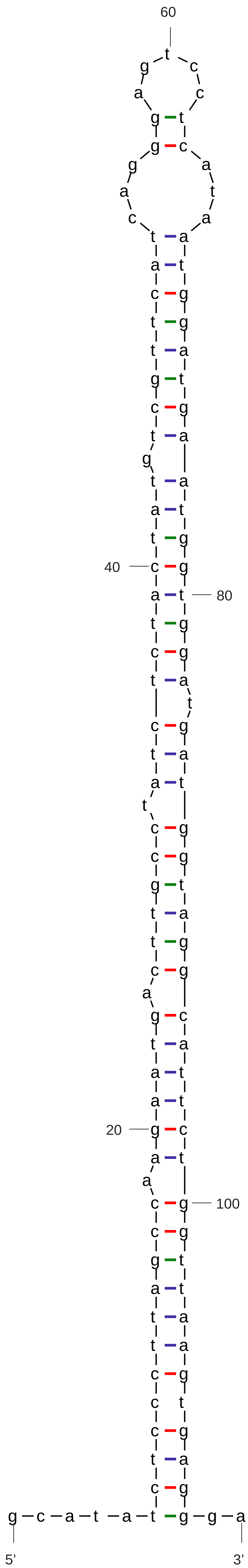

dG = -49.8 sit_novel_miR48

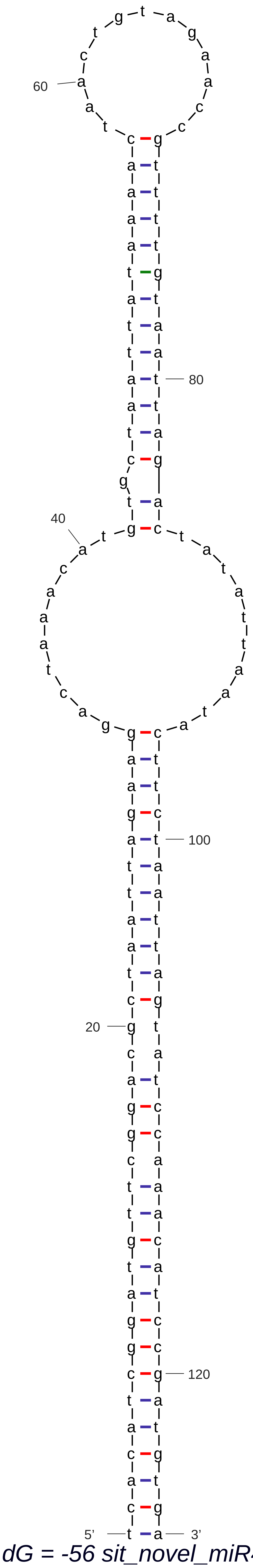

dG = -56 sit_novel_miR49

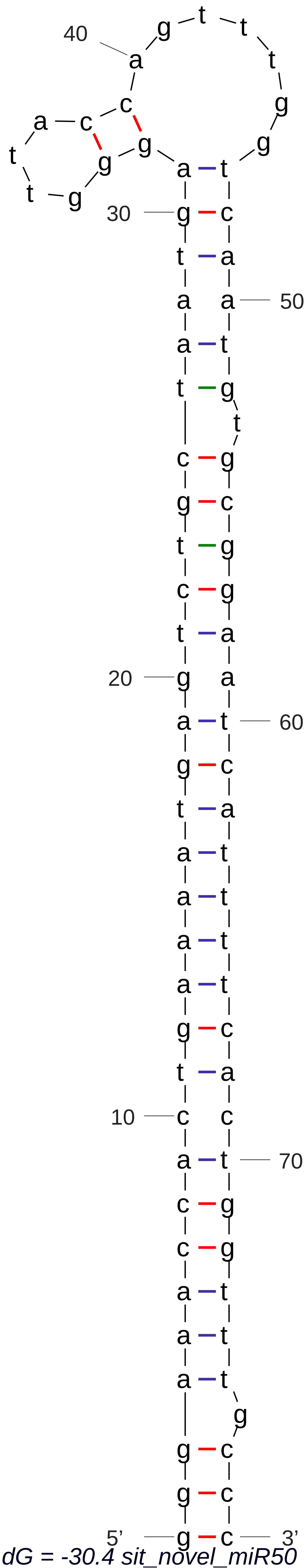

dG = -30.4 sit_novel_miR50

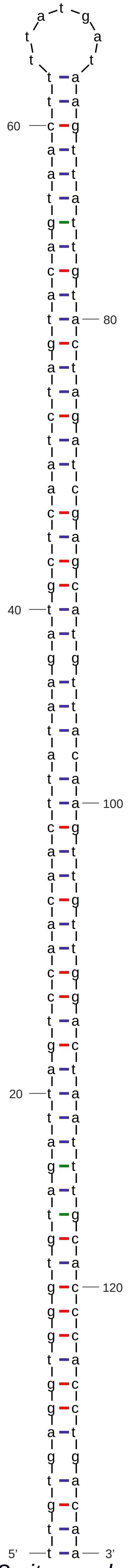

dG = -89.8 sit_novel_miR51-1

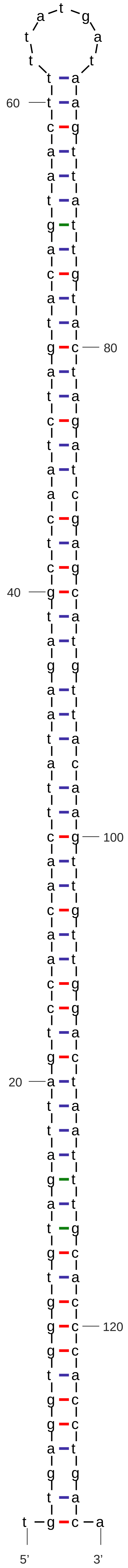

dG = -89 sit_novel_miR51-2

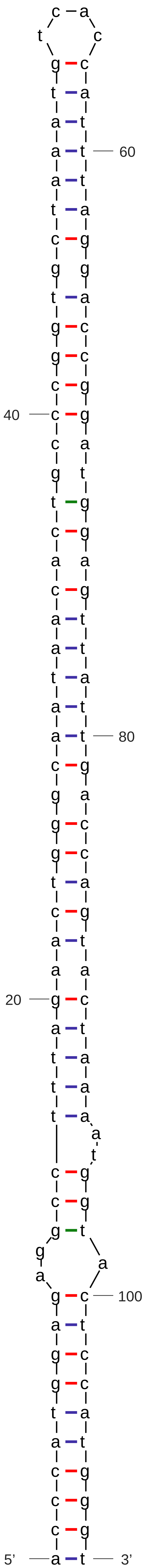

dG = -60.8 sit_novel_miR52

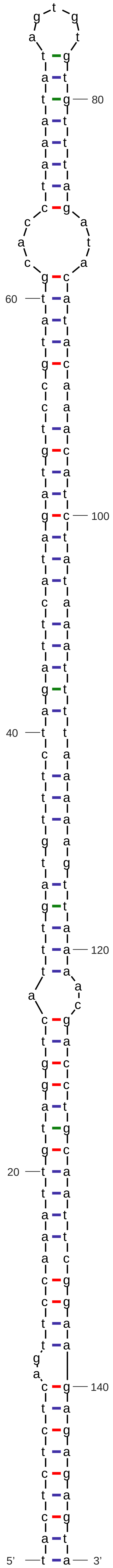

dG = -58.9 sit_novel_miR53

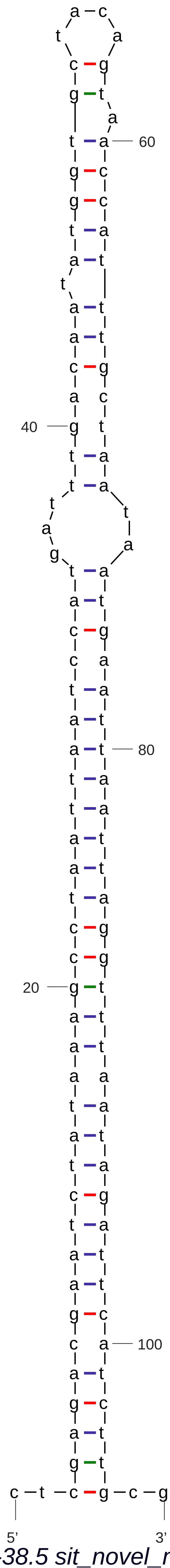

dG = -38.5 sit_novel_miR54

Output of sir_graph (©)
mfold_util 4.7

Created Tue Feb 23 05:57:32 2016

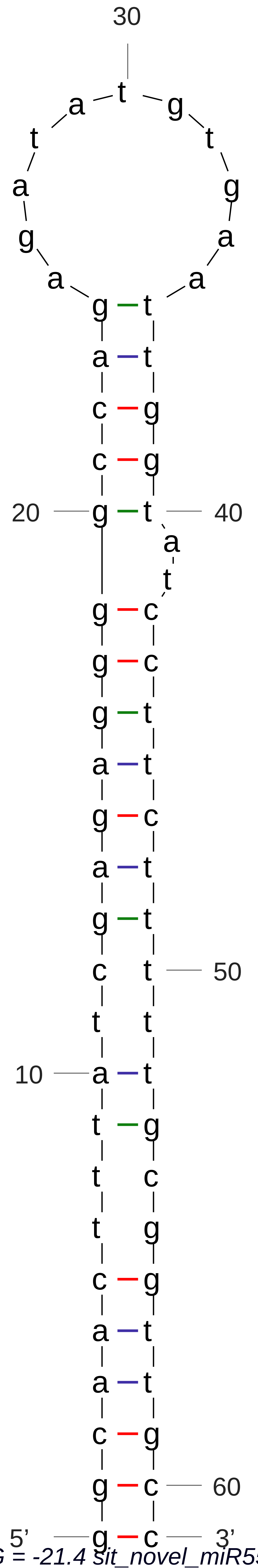

dG = -21.4 sit_novel_miR55

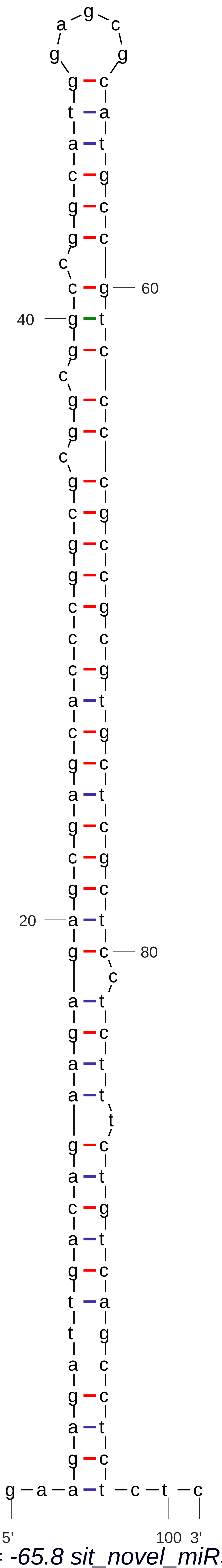

dG = -65.8 sit_novel_miR56

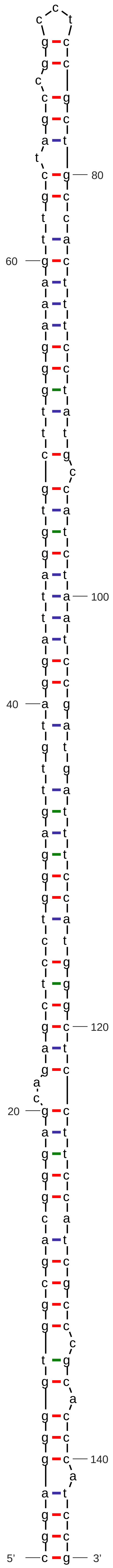

dG = -82.2 sit_novel_miR57-1

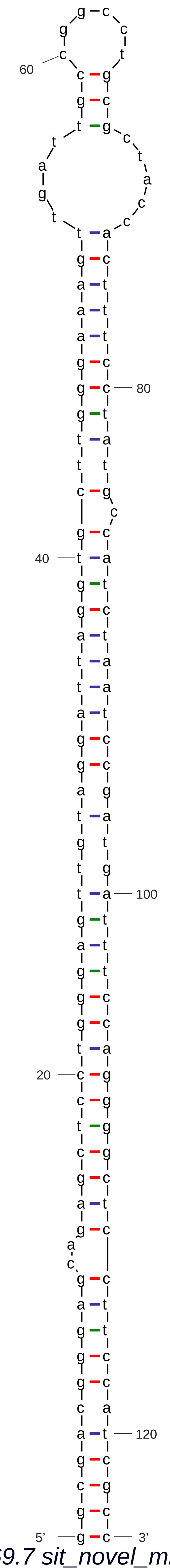

dG = -69.7 sit_novel_miR57-2

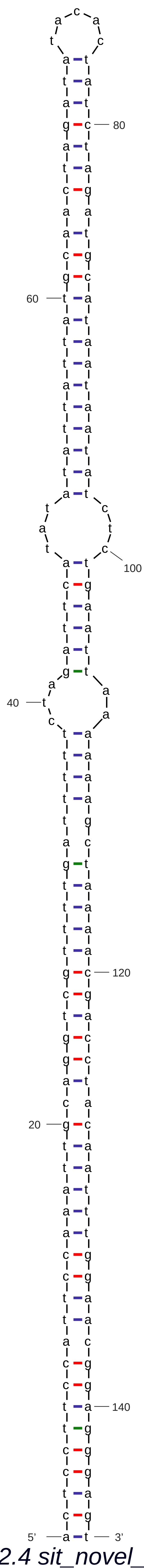

dG = -72.4 sit_novel_miR58

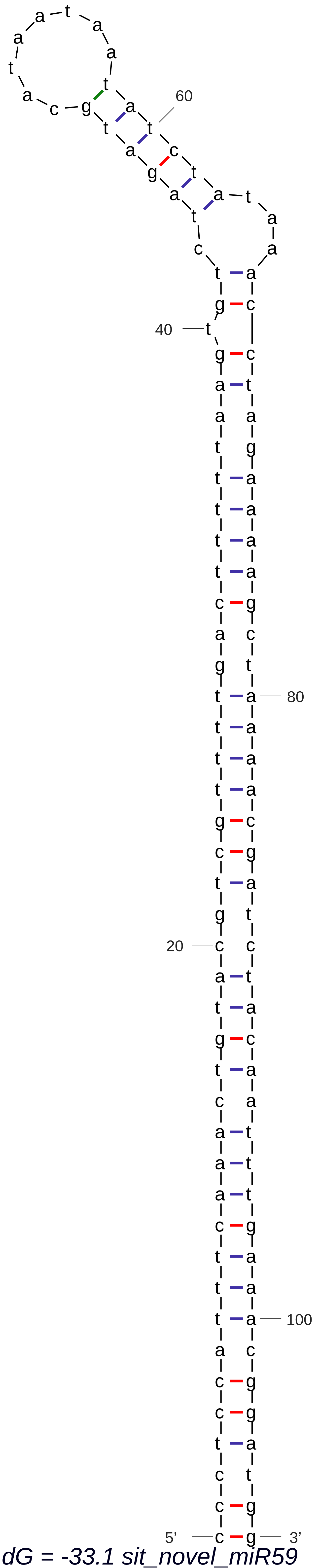

dG = -33.1 sit_novel_miR59

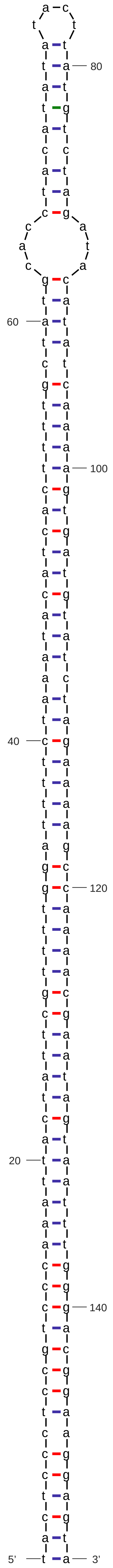

dG = -89.4 sit_novel_miR60-1

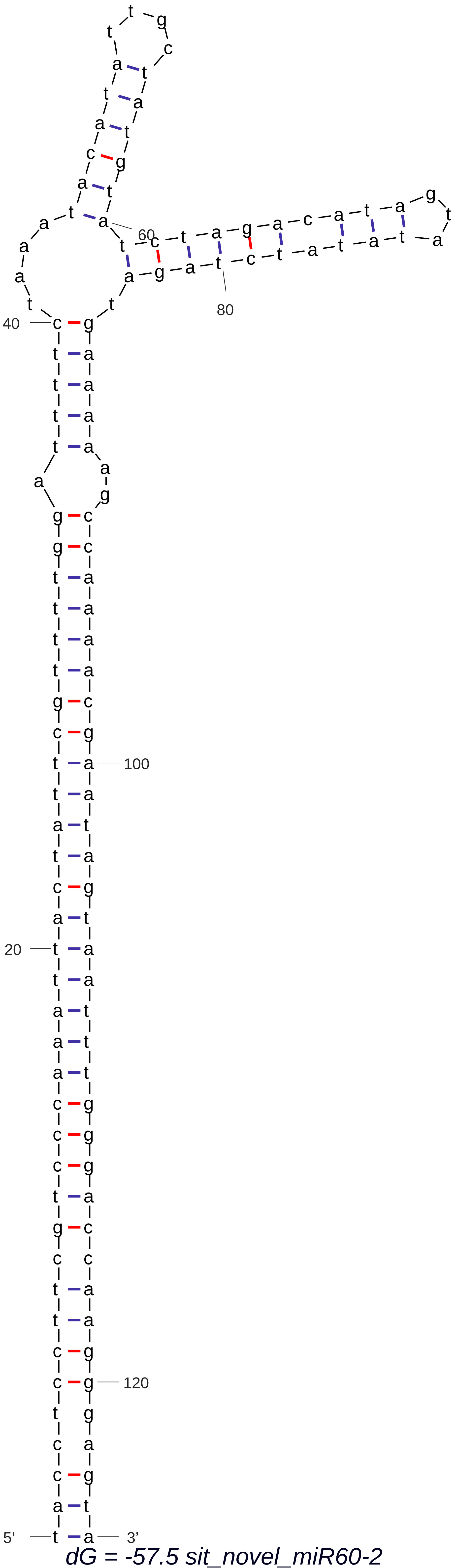

dG = -57.5 sit_novel_miR60-2

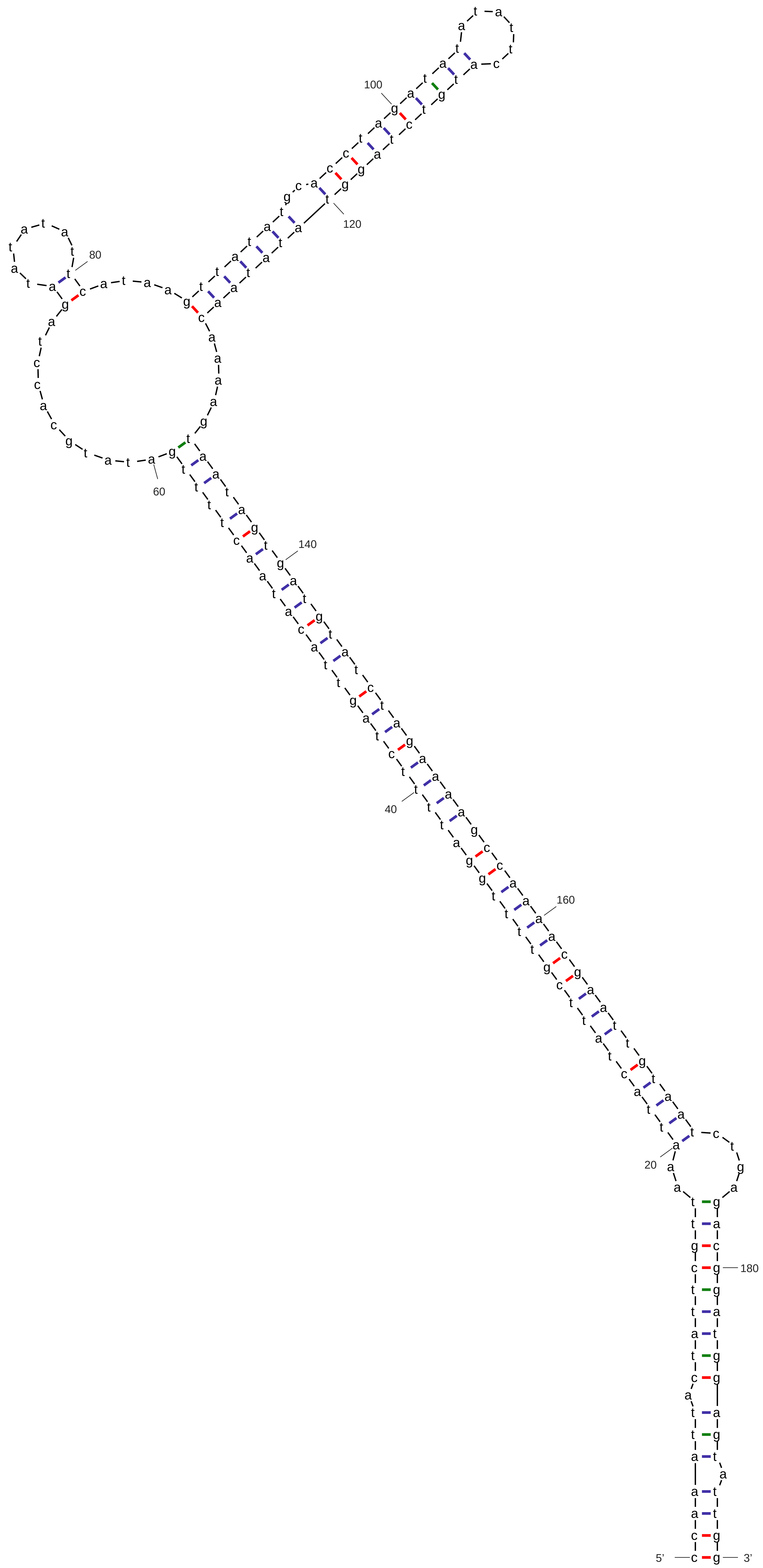

dG = -64.8 sit_novel_miR60-3

Output of sir_graph (©)
mfold_util 4.7

Created Tue Feb 23 06:00:02 2016

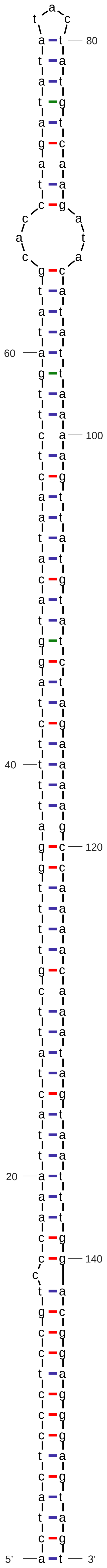

dG = -89.4 sit_novel_miR60-4

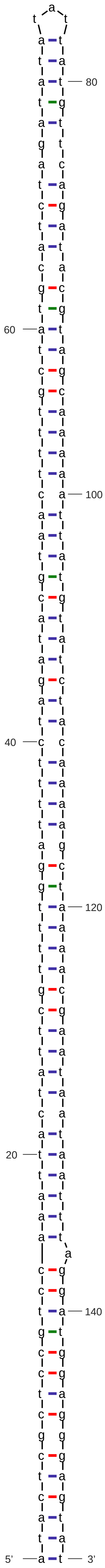

dG = -75.5 sit_novel_miR60-5

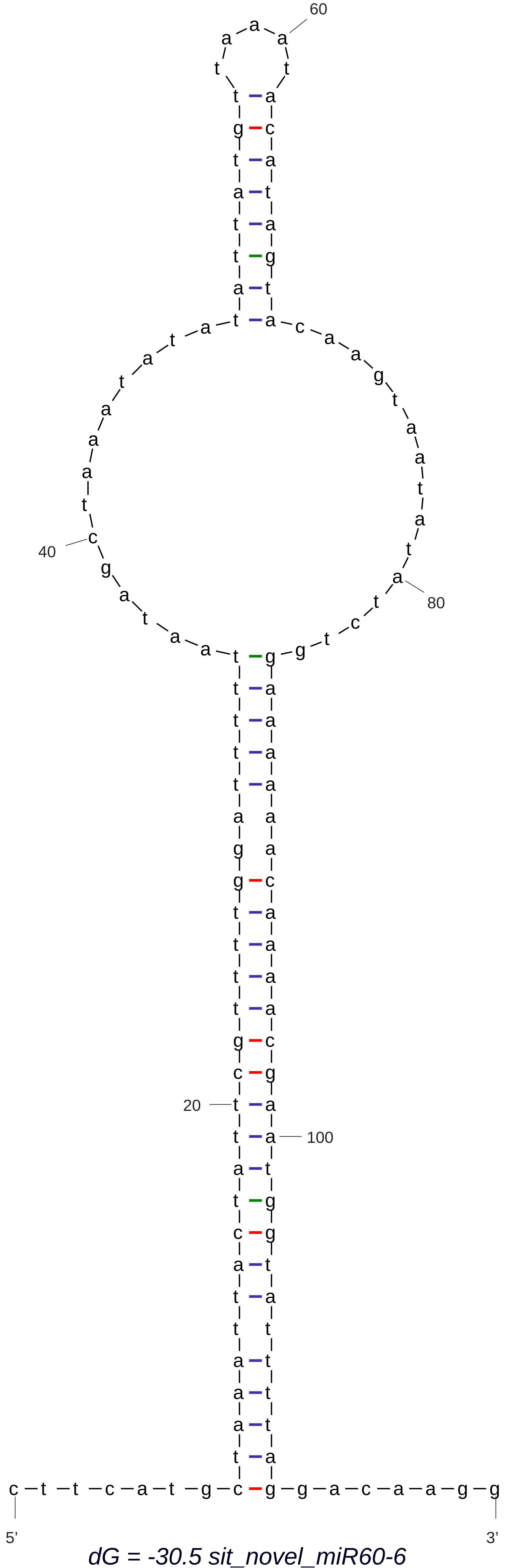

dG = -30.5 sit_novel_miR60-6

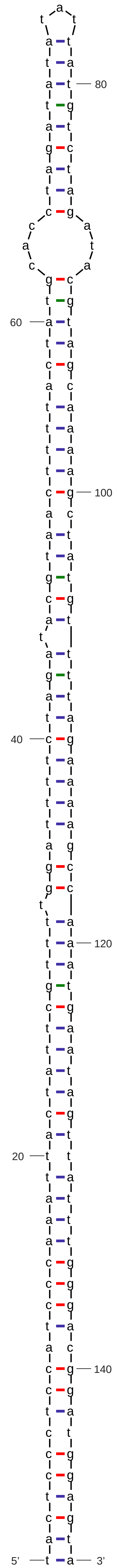

dG = -68.7 sit_novel_miR60-7

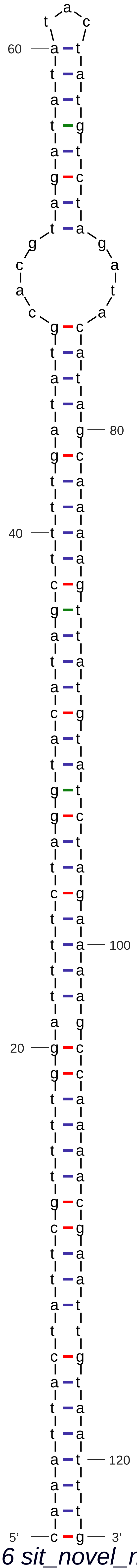

dG = -60.6 sit_novel_miR60-8

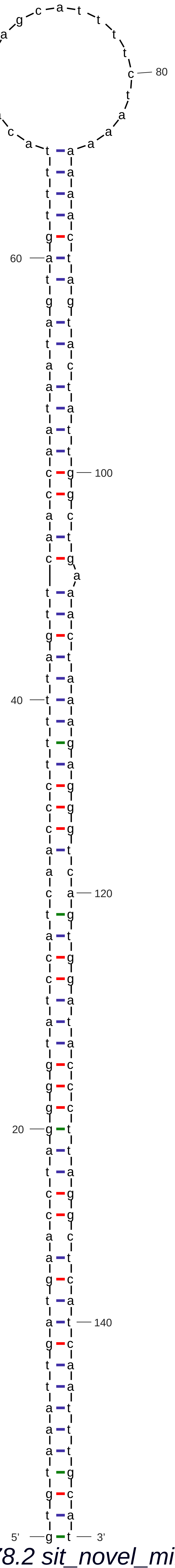

dG = -78.2 sit_novel_miR61

Output of sir_graph (©)
mfold_util 4.7

Created Tue Feb 23 06:02:23 2016

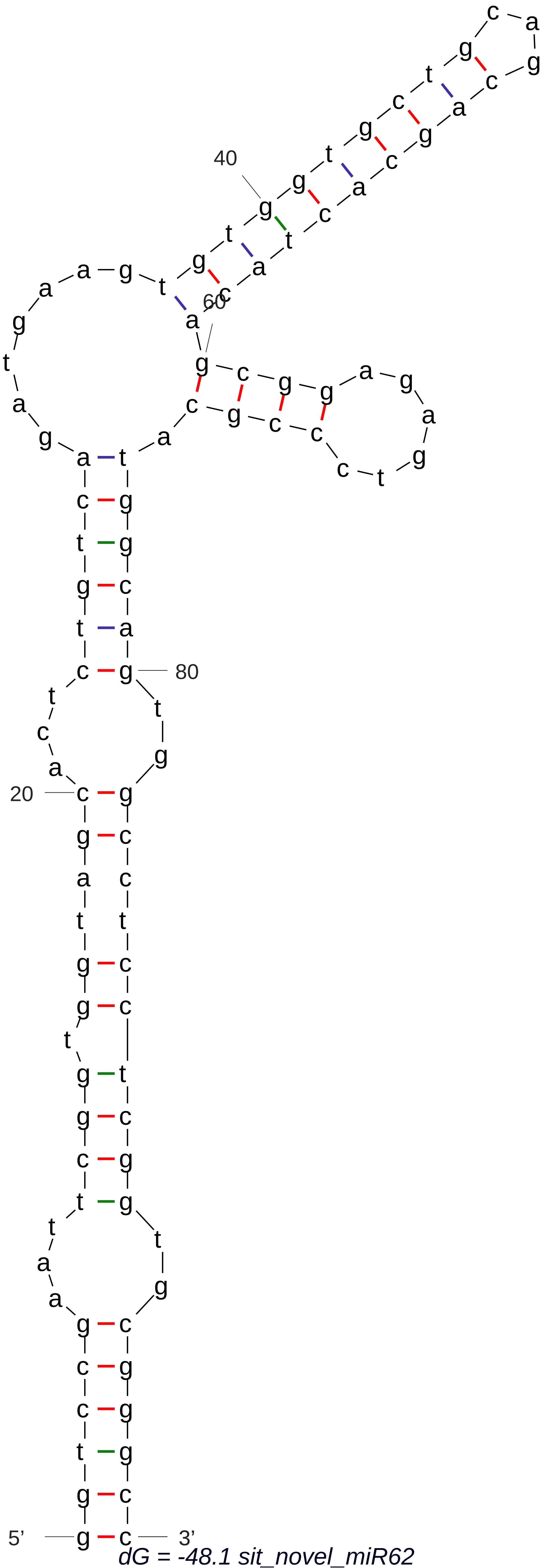

dG = -48.1 sit_novel_miR62

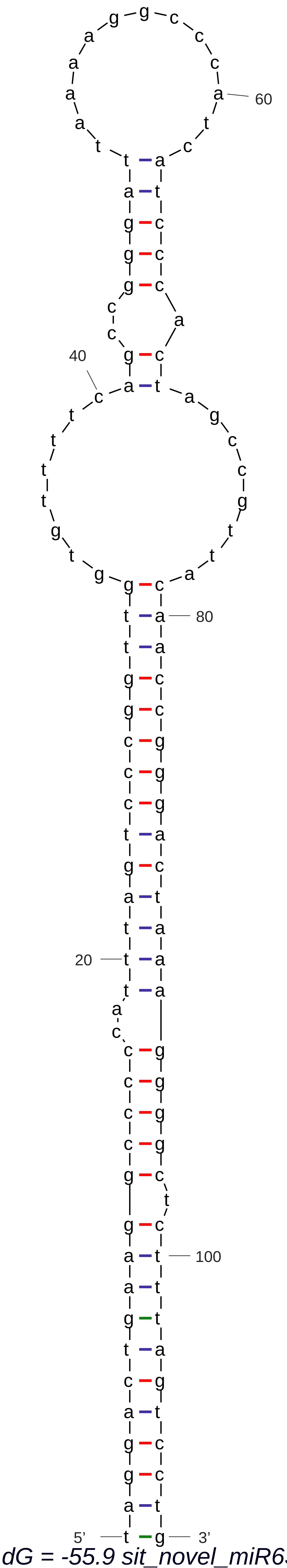

dG = -55.9 sit_novel_miR63
