# Supplementary material for: Combined small RNA and degradome sequencing to identify miRNAs and their targets in response to drought in foxtail millet
Source: BMC Genet. 2016 Apr 12;17:57. doi: 10.1186/s12863-016-0364-7 (PMC4828802; doi:10.1186/s12863-016-0364-7)
Supplement: Additional file 9: — Target plots of identified miRNA targets using degradome sequencing. (PDF 762 kb) [file 12863_2016_364_MOESM9_ESM.pdf]

Si005991m pacid=19704688 locus=Si005991m.g annot-version=v2.1

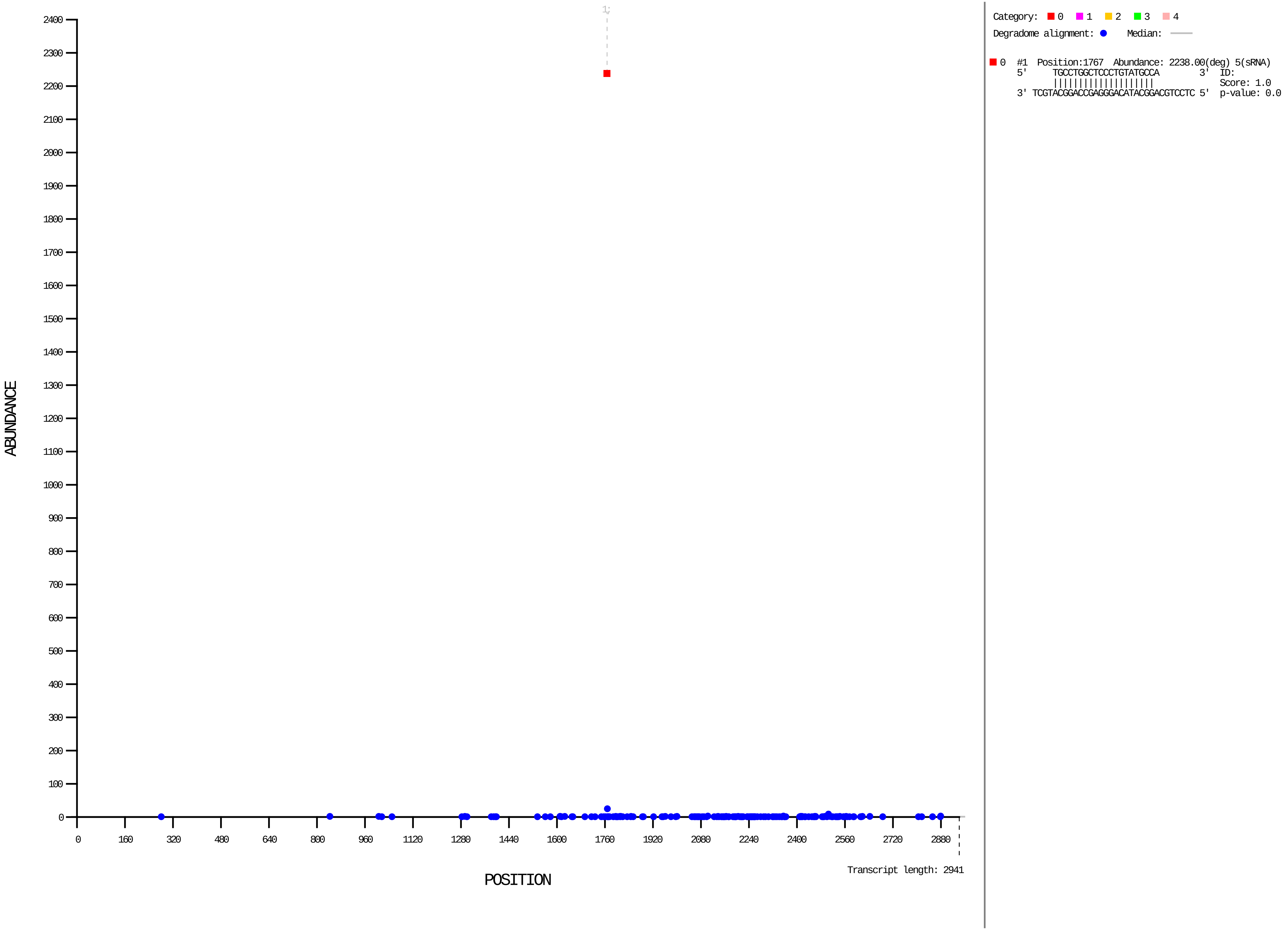

Si034525m pacid=19682948 locus=Si034525m.g annot-version=v2.1

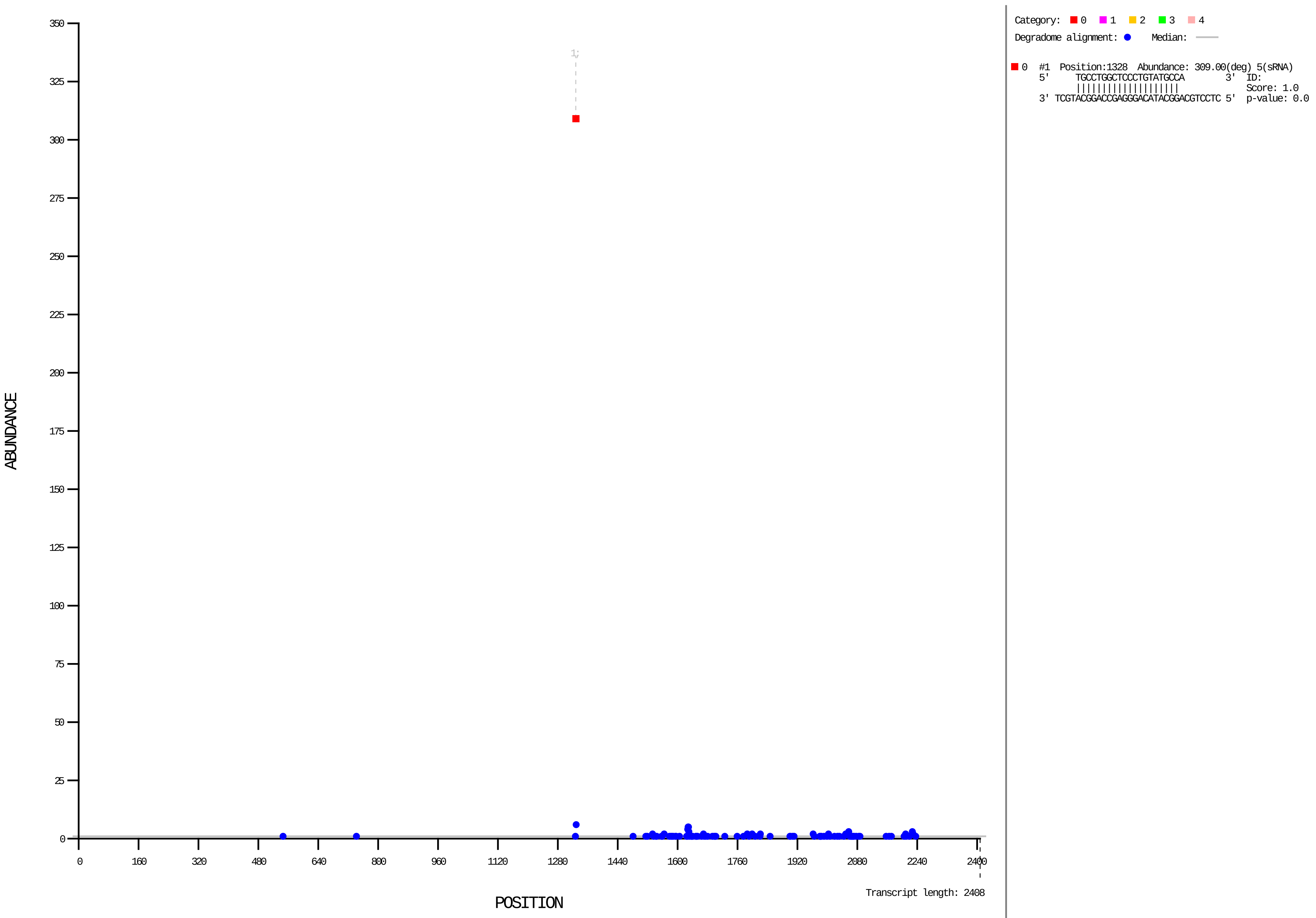

Si035794m pacid=19683976 locus=Si035794m.g annot-version=v2.1

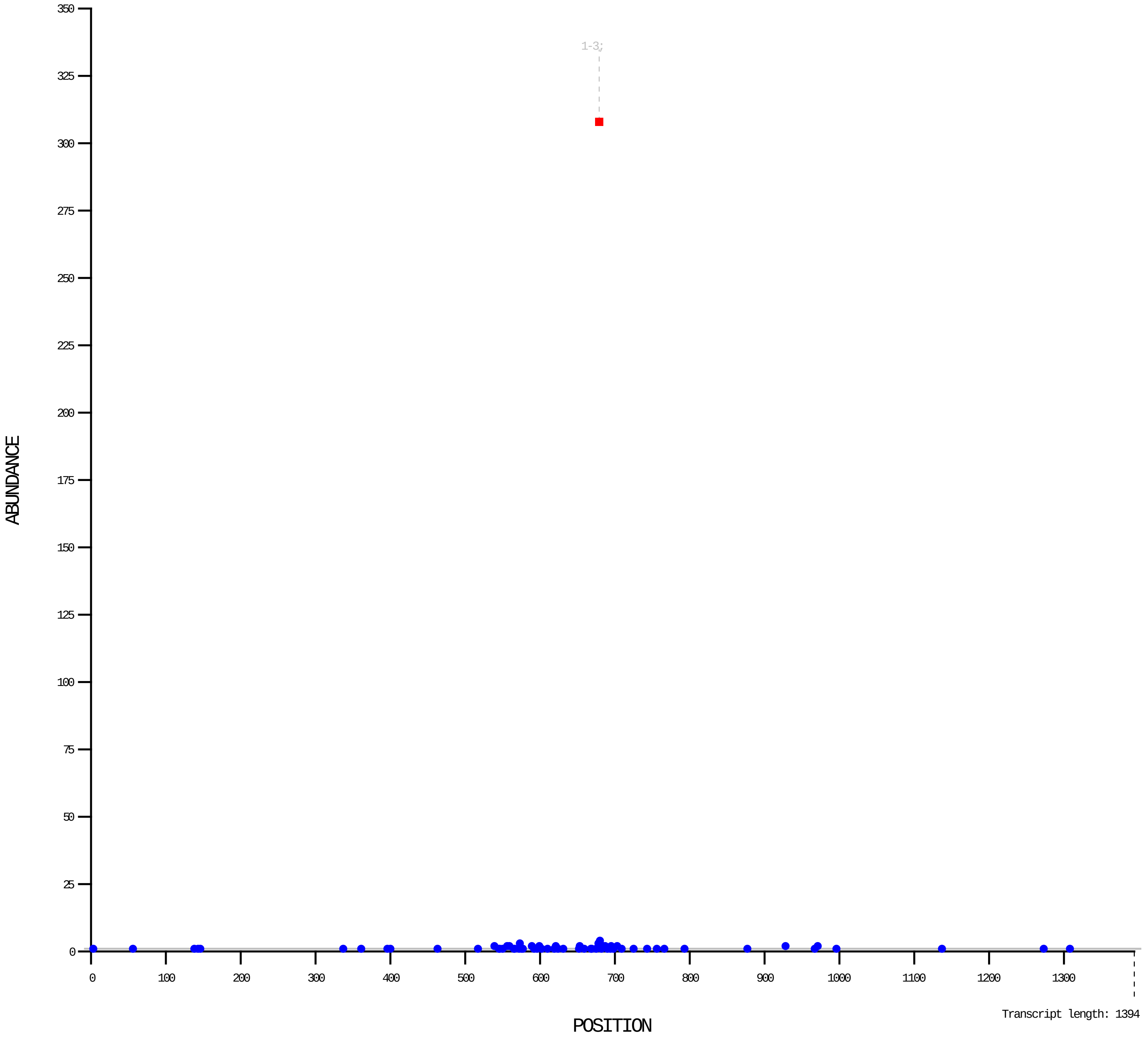

Category: 0 1 2 3 4
Degradome alignment: Median:

0 #1 Position:679 Abundance: 308.00(deg) 2(sRNA)
5' TTCCACA-GCTTTCTTGAACTG 3' ID:
Score: 2.0
3' CAAAAGGTGTCCGAAAGAACTTGCCACTGCCA 5' p-value: 0.0

0 #2 Position:679 Abundance: 308.00(deg) 1(sRNA)
5' TCCACAGGCTTTCTTGAACTG 3' ID:
Score: 1.0
3' CAAAAGGTGTCCGAAAGAACTTGCCACTGCCA 5' p-value: 0.0

0 #3 Position:679 Abundance: 308.00(deg) 1(sRNA)
5' TTCCACA-GCTTTCTTGAACTT 3' ID:
Score: 3.0
3' CAAAAGGTGTCCGAAAGAACTTGCCACTGCCA 5' p-value: 0.0

Si034251m pacid=19684049 locus=Si034251m.g annot-version=v2.1

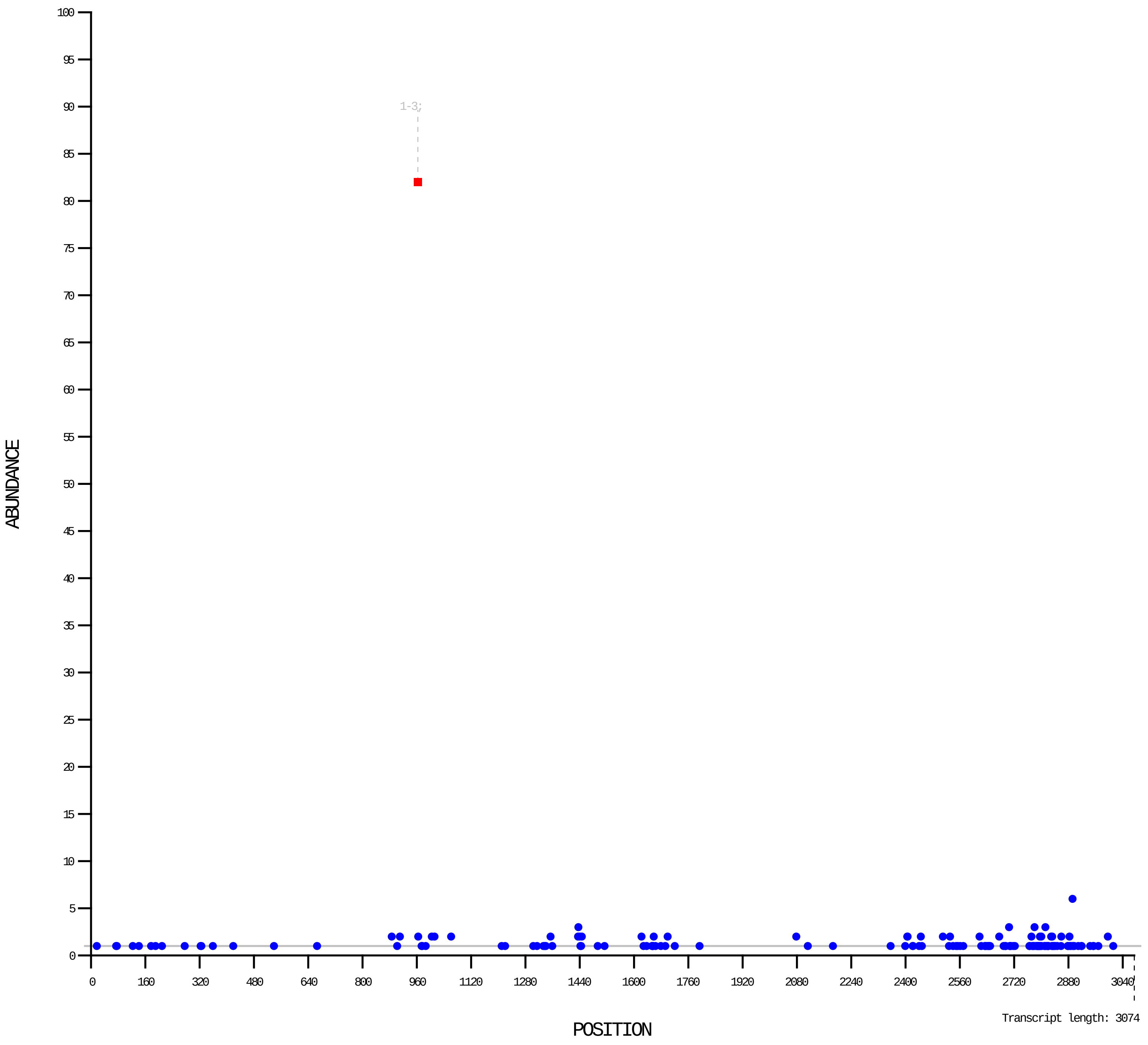

Category: 0 1 2 3 4
Degradome alignment: Median:

0 #1 Position:963 Abundance: 82.00(deg) 7(sRNA)
5' TCGGACCAGGCTTCATTCCCC 3' ID:
o|||||||||||||||| ||| Score: 2.5
3' CTTAGGCCTGGTCCGAAGTA-GGGTCCGTAGA 5' p-value: 0.0

0 #2 Position:963 Abundance: 82.00(deg) 2(sRNA)
5' TCGGACCAGGCTTCAATCCCT 3' ID:
o|||||||||||||||| |||| Score: 2.5
3' CTTAGGCCTGGTCCGAAGT-AGGGTCCGTAGA 5' p-value: 0.0

0 #3 Position:963 Abundance: 82.00(deg) 2(sRNA)
5' TCGGACCAGGCTTCATTCCTC 3' ID:
o||||||||||||||||o|| Score: 3.0
3' CTTAGGCCTGGTCCGAAGTAGGGTCCGTAGAC 5' p-value: 0.0

Si008818m pacid=19706159 locus=Si008818m.g annot-version=v2.1

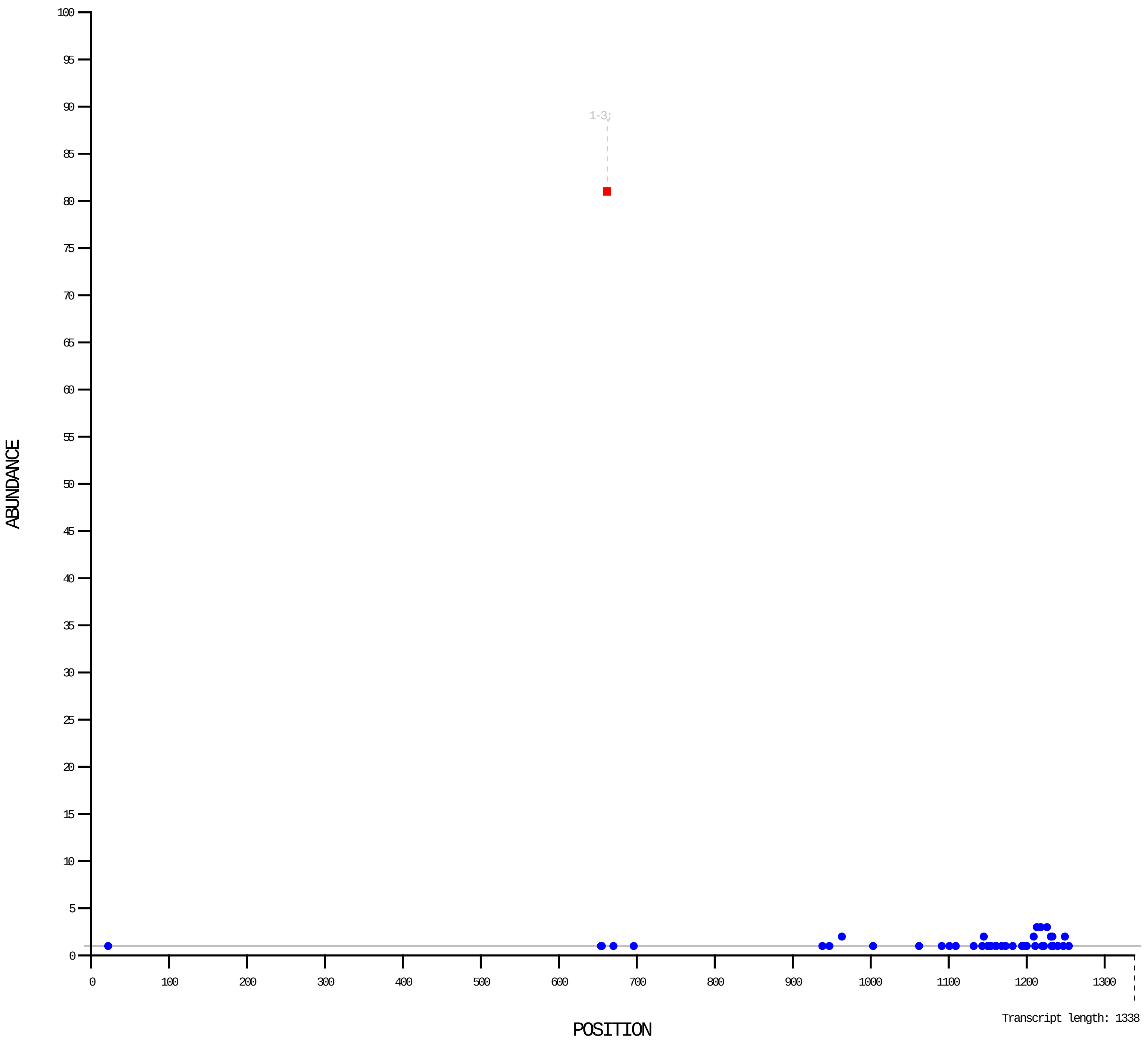

Category: 0 1 2 3 4
Degradome alignment: Median:

```
0  #1  Position:662  Abundance: 81.00(deg) 2(sRNA)
   5'    TTCCACA-GCTTTCTTGAACTG        3'  ID:
         ||||||| ||||||||||||| |           Score: 2.0
   3' CCTAAGGTGTCCGAAAGAACTTGCCAACGCCG 5'  p-value: 0.0

0  #2  Position:662  Abundance: 81.00(deg) 1(sRNA)
   5'     TCCACAGGCTTTCTTGAACTG        3'  ID:
          ||||||||||||||||||| |            Score: 1.0
   3' CCTAAGGTGTCCGAAAGAACTTGCCAACGCCG 5'  p-value: 0.0

0  #3  Position:662  Abundance: 81.00(deg) 1(sRNA)
   5'    TTCCACA-GCTTTCTTGAACTT        3'  ID:
         ||||||| ||||||||||||              Score: 3.0
   3' CCTAAGGTGTCCGAAAGAACTTGCCAACGCCG 5'  p-value: 0.0
```

Si035812m pacid=19681742 locus=Si035812m.g annot-version=v2.1

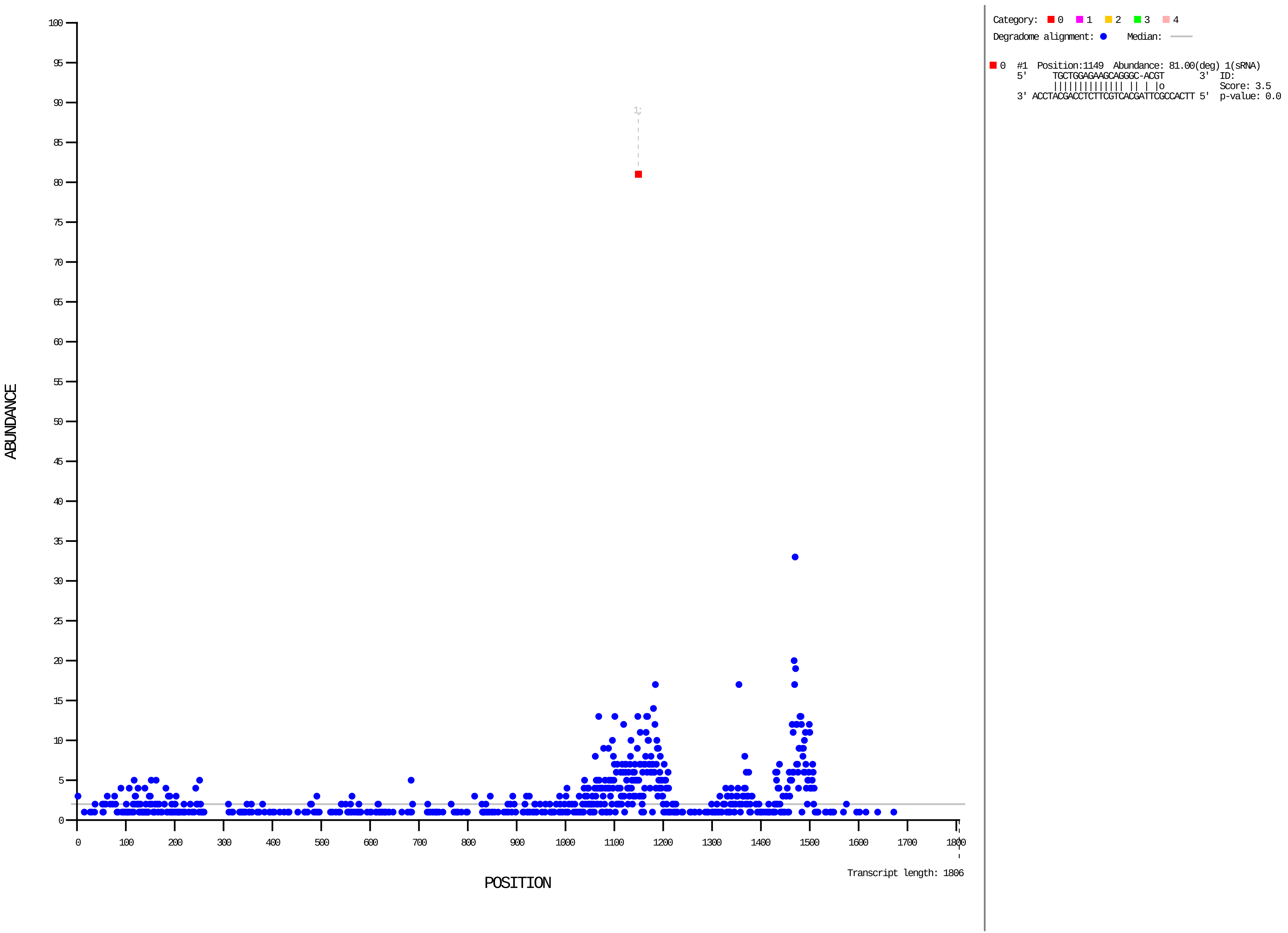

Si001804m pacid=19677692 locus=Si001804m.g annot-version=v2.1

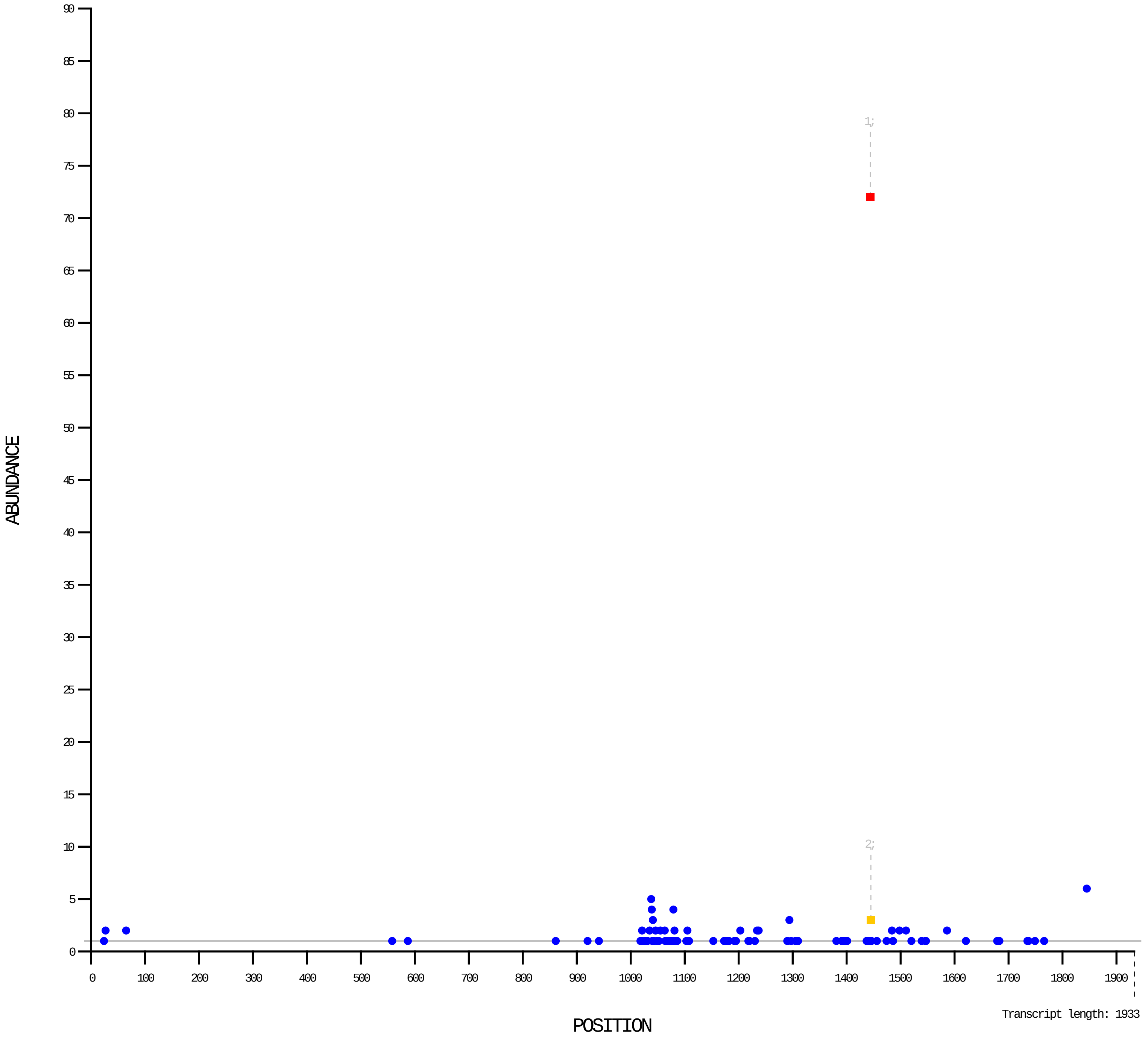

Category: 0 1 2 3 4
Degradome alignment: Median:

```
0  #1  Position:1444  Abundance: 72.00(deg) 8(sRNA)
   5'     TGACAGAAGAGAGTGAGCAC        3'  ID:
          ||||||||||||| ||||||            Score: 1.0
   3' ACAGACTGTCTTCTCTCTCTCGTGTCAACGGC 5'  p-value: 0.0

2  #2  Position:1445  Abundance: 3.00(deg) 1(sRNA)
   5'     TTGACAGAAGAGAGCGAGCAC       3'  ID:
          o||||||||||||| ||||||           Score: 1.5
   3' AACAGACTGTCTTCTCTCTCTCGTGTCAACGG 5'  p-value: 0.0
```

Si009523m pacid=19695695 locus=Si009523m.g annot-version=v2.1

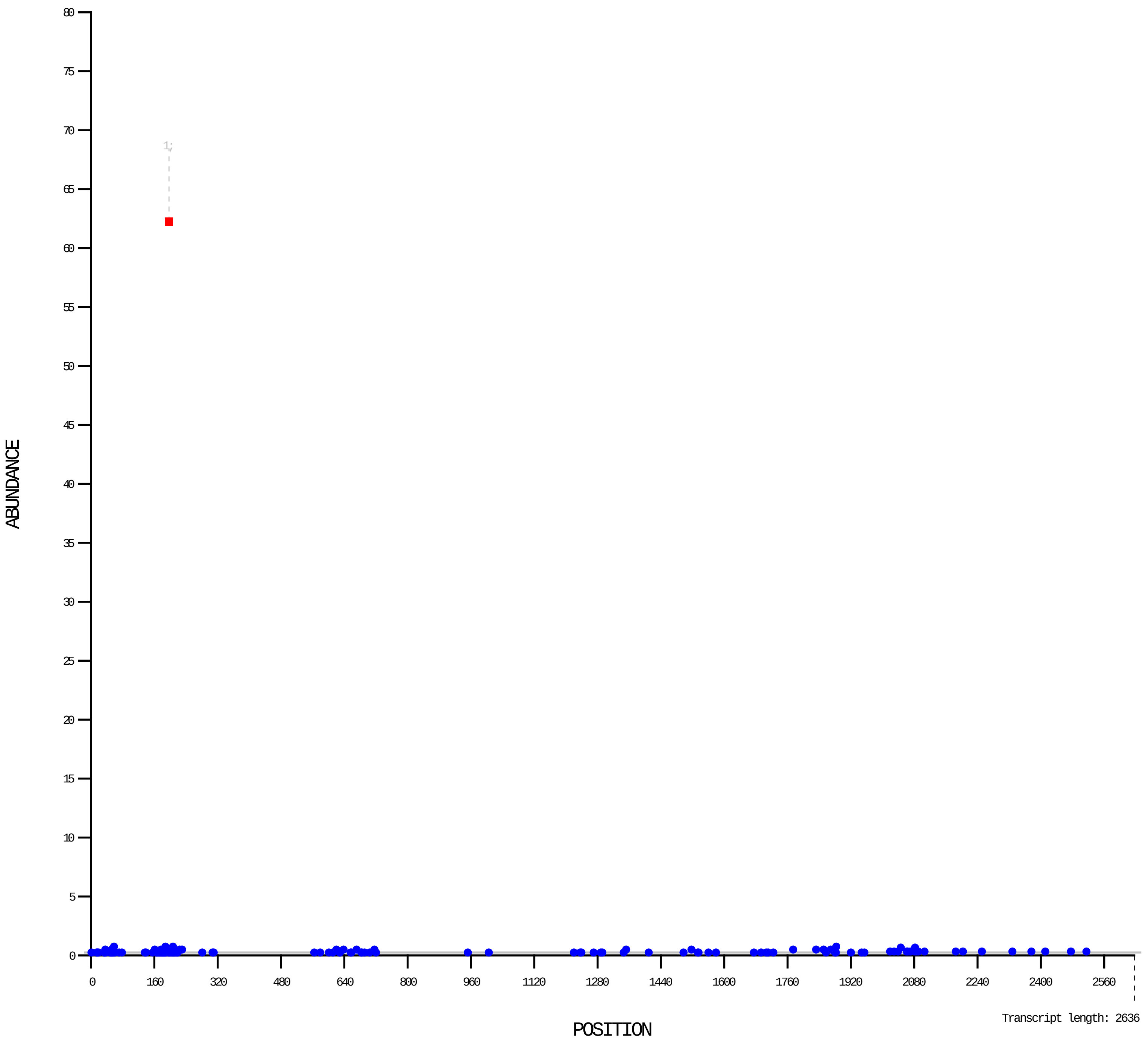

Category: 0 1 2 3 4

Degradome alignment: Median:

0 #1 Position:197 Abundance: 62.25(deg) 1(sRNA)

```
5'     TTAGATGACCATCAGCAAACA        3'  ID:
       |||||||| ||||||||||||            Score: 1.0
3'  TGTTAATCTACTTGTAGTCGTTTGTGTTTGTG 5'  p-value: 0.0
```

Si009851m pacid=19695698 locus=Si009523m.g annot-version=v2.1

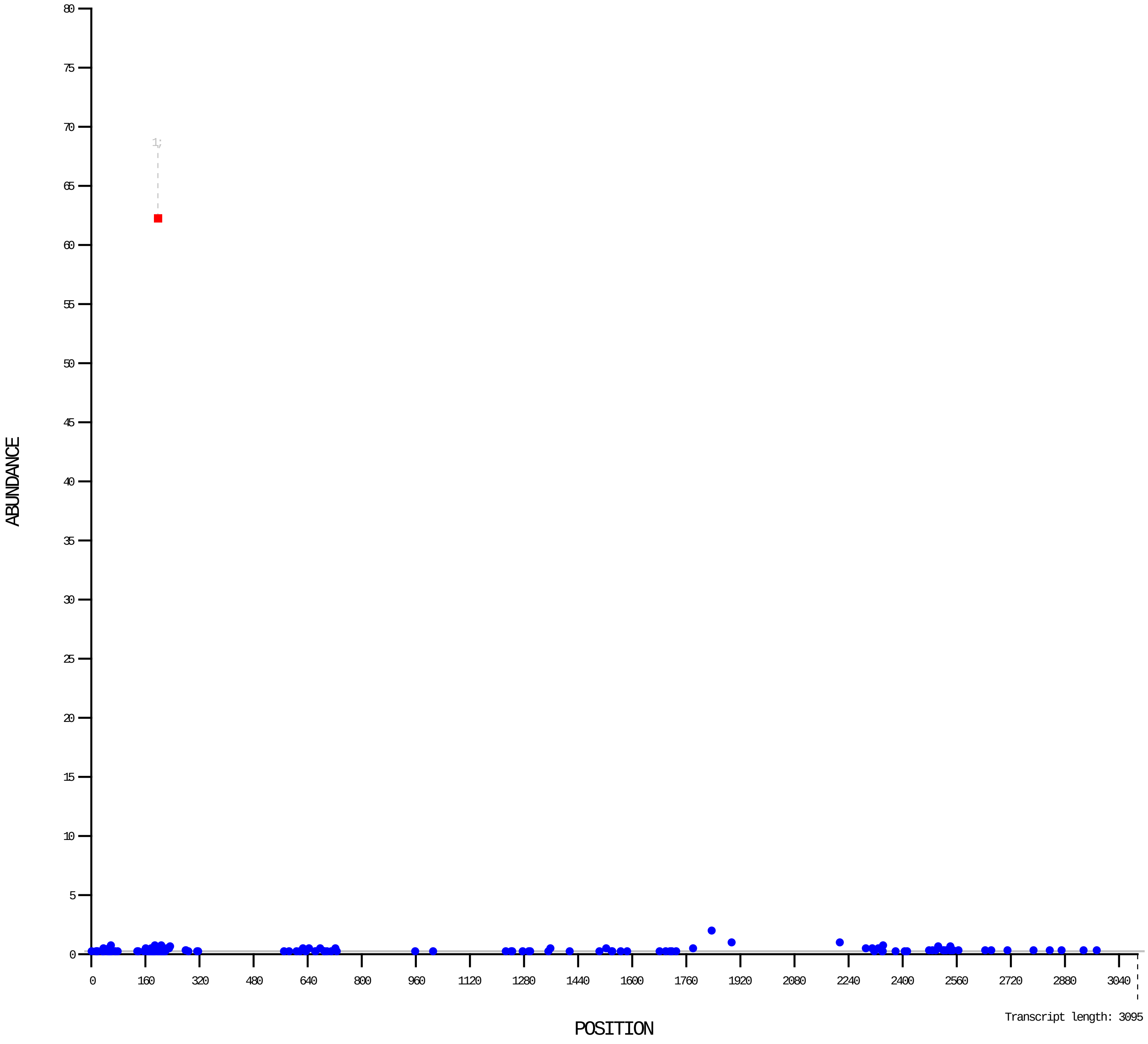

Category: 0 1 2 3 4
Degradome alignment: Median:

0 #1 Position:197 Abundance: 62.25(deg) 1(sRNA)
5' TTAGATGACCATCAGCAAACA 3' ID:
|||||||| ||||||||||||| Score: 1.0
3' TGTTAATCTACTTGTAGTCGTTTGTGTTTGTG 5' p-value: 0.0

Si009706m pacid=19695697 locus=Si009523m.g annot-version=v2.1

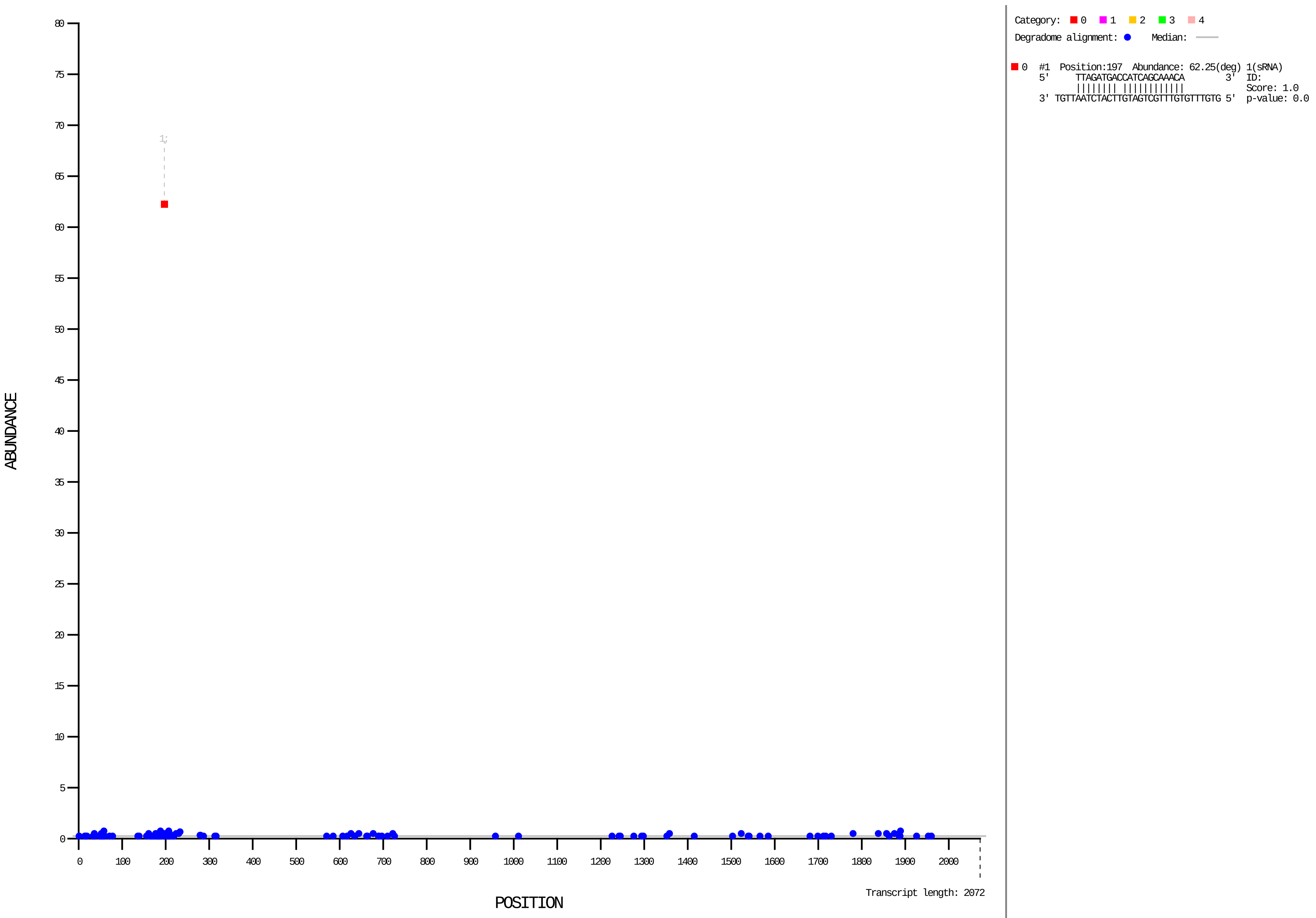

Si009522m pacid=19695696 locus=Si009523m.g annot-version=v2.1

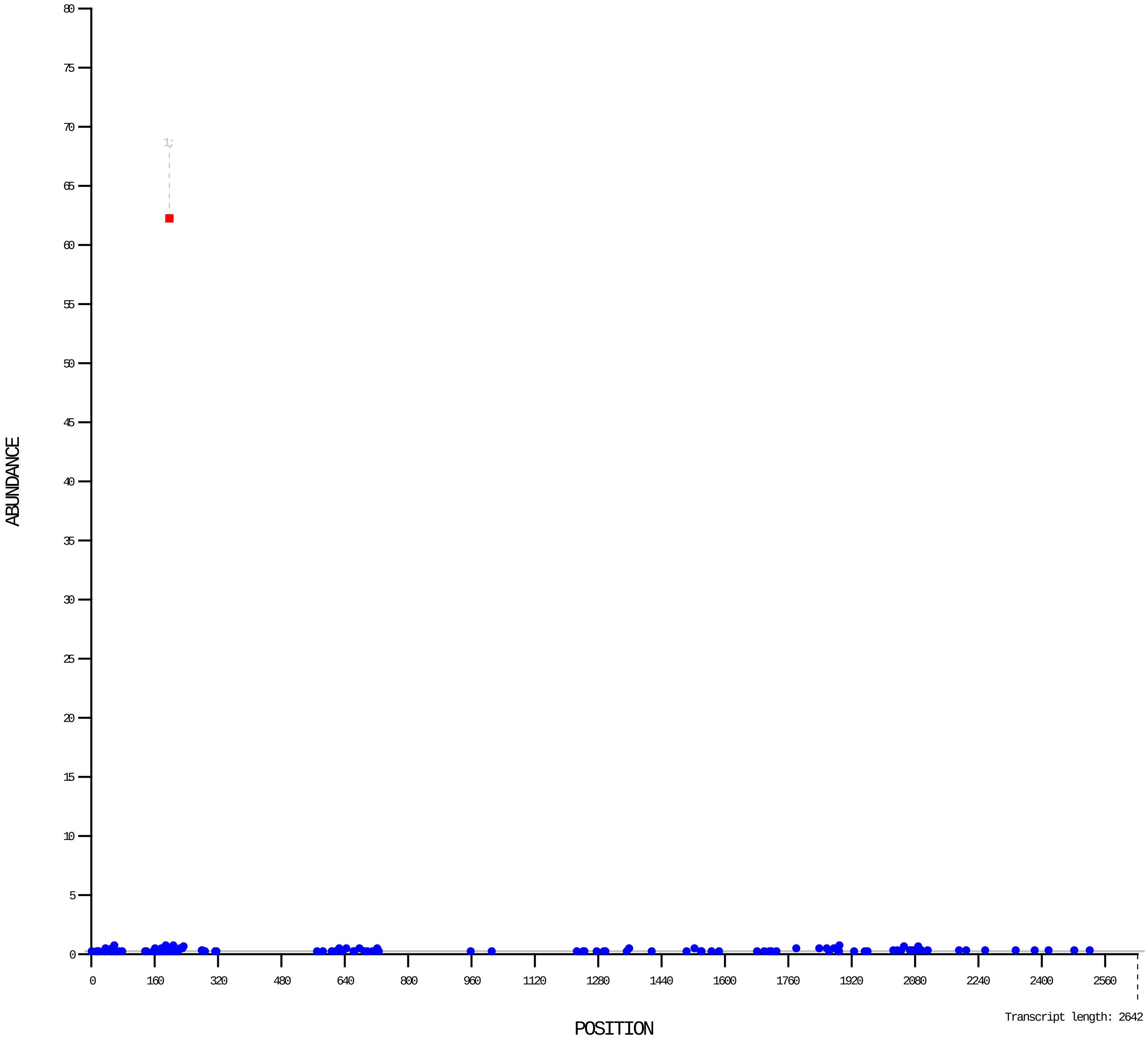

Category: 0 1 2 3 4

Degradome alignment: Median:

0 #1 Position:197 Abundance: 62.25(deg) 1(sRNA)

```
5'     TTAGATGACCATCAGCAAACA        3'  ID:
       |||||||| ||||||||||||            Score: 1.0
3' TGTTAATCTACTTGTAGTCGTTTGTGTTTGTG 5'  p-value: 0.0
```

Si016509m pacid=19686089 locus=Si016509m.g annot-version=v2.1

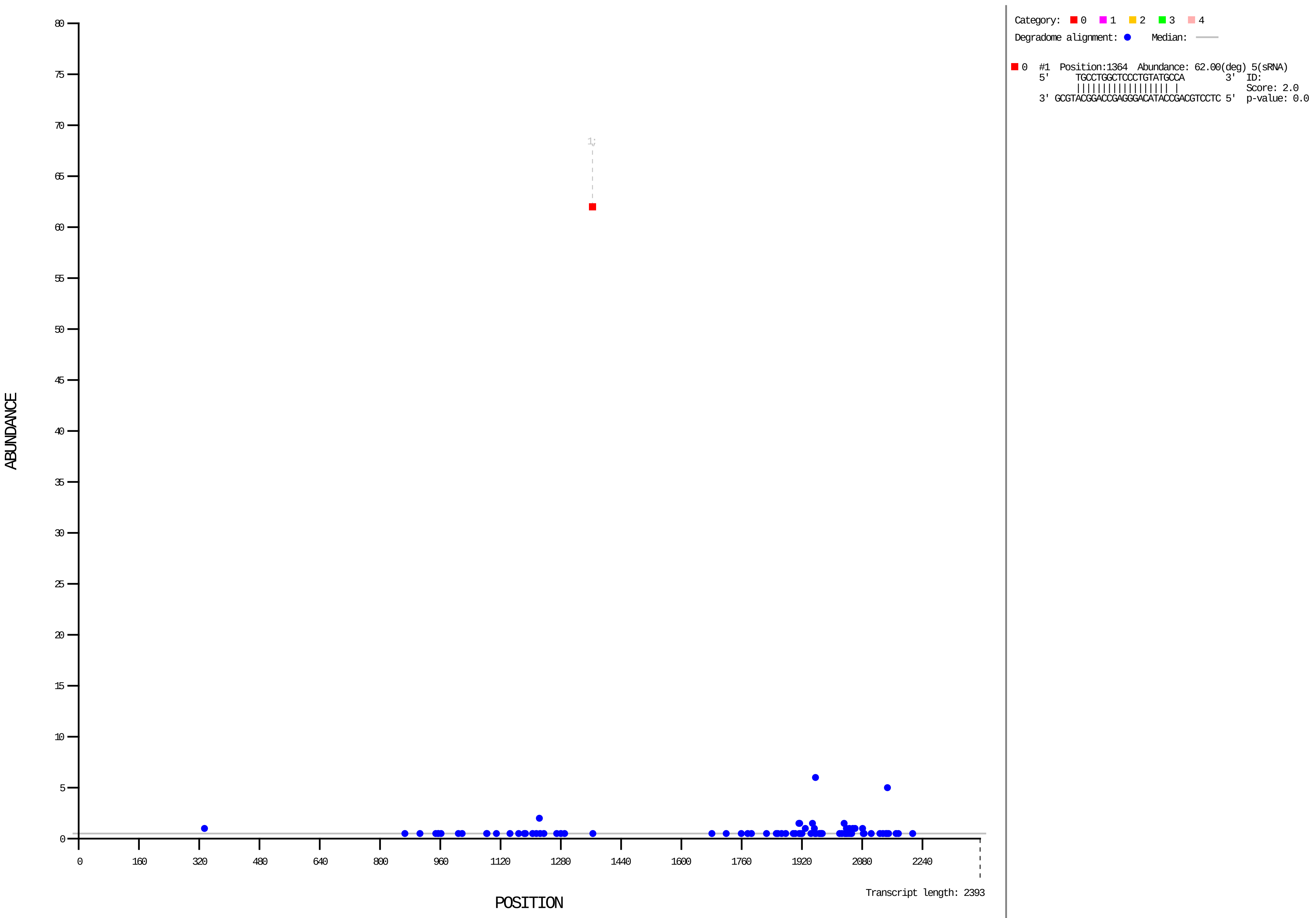

Si016559m pacid=19686090 locus=Si016509m.g annot-version=v2.1

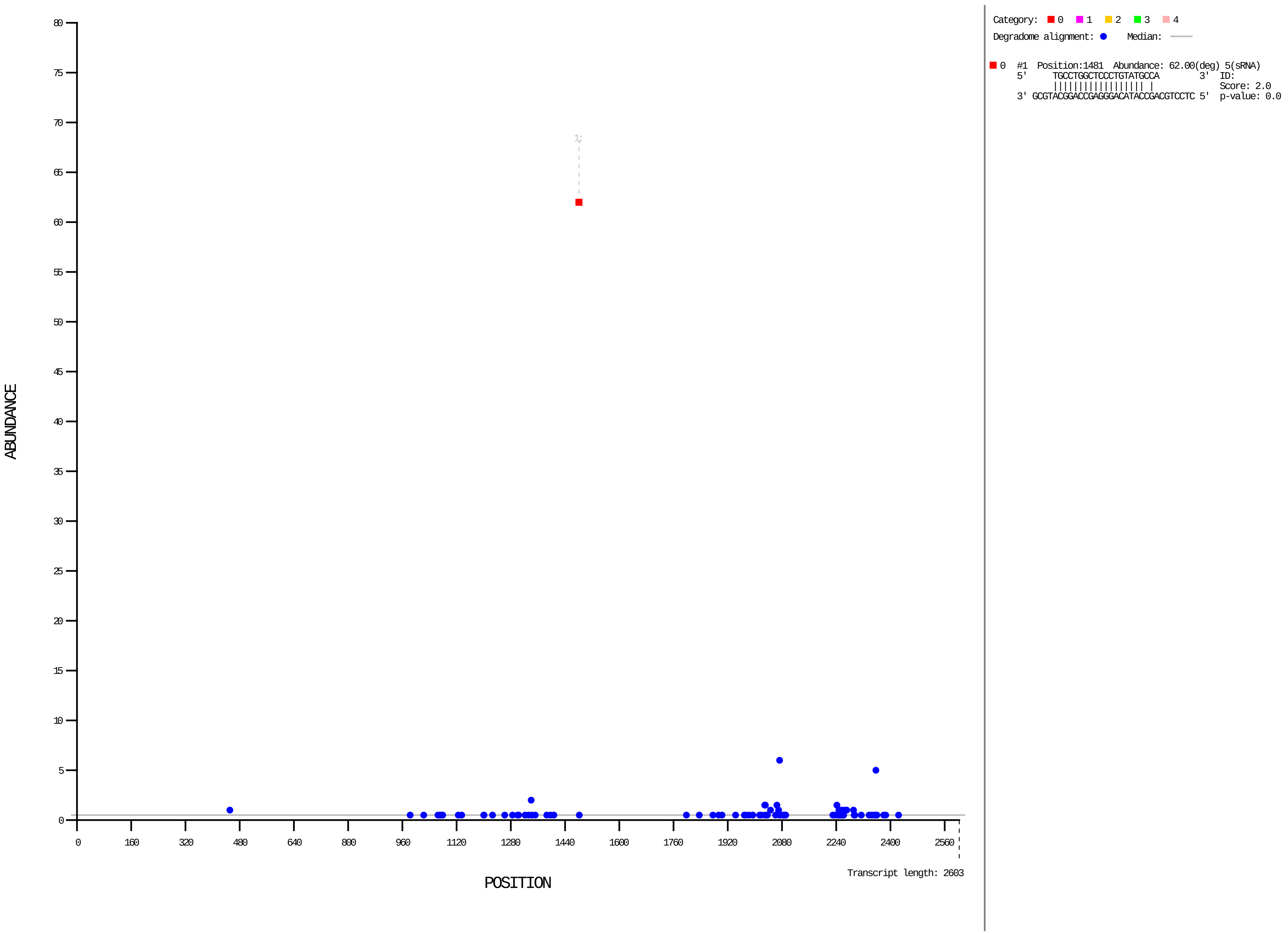

Si033853m pacid=19680293 locus=Si033853m.g annot-version=v2.1

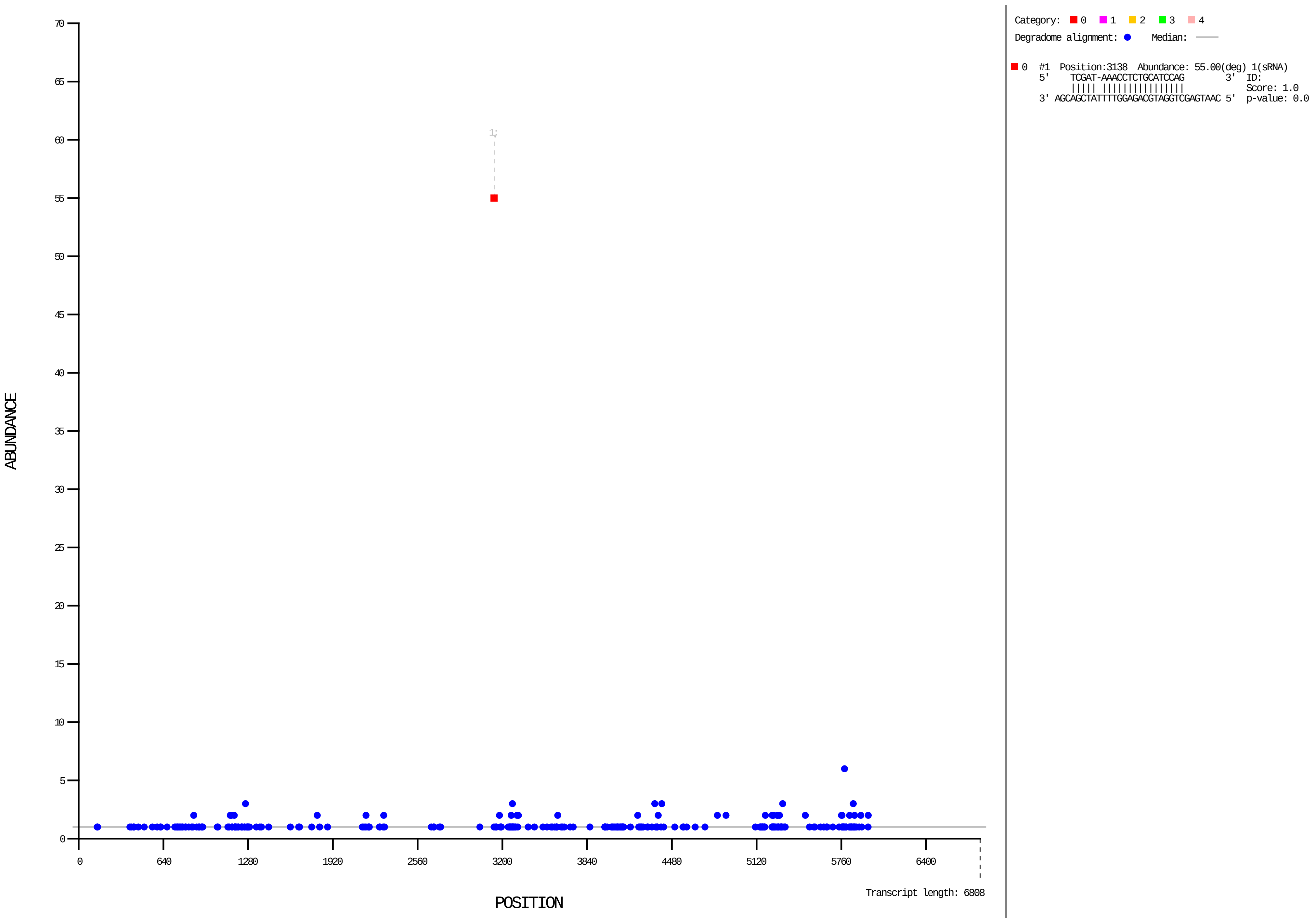

Si016508m pacid=19689224 locus=Si016508m.g annot-version=v2.1

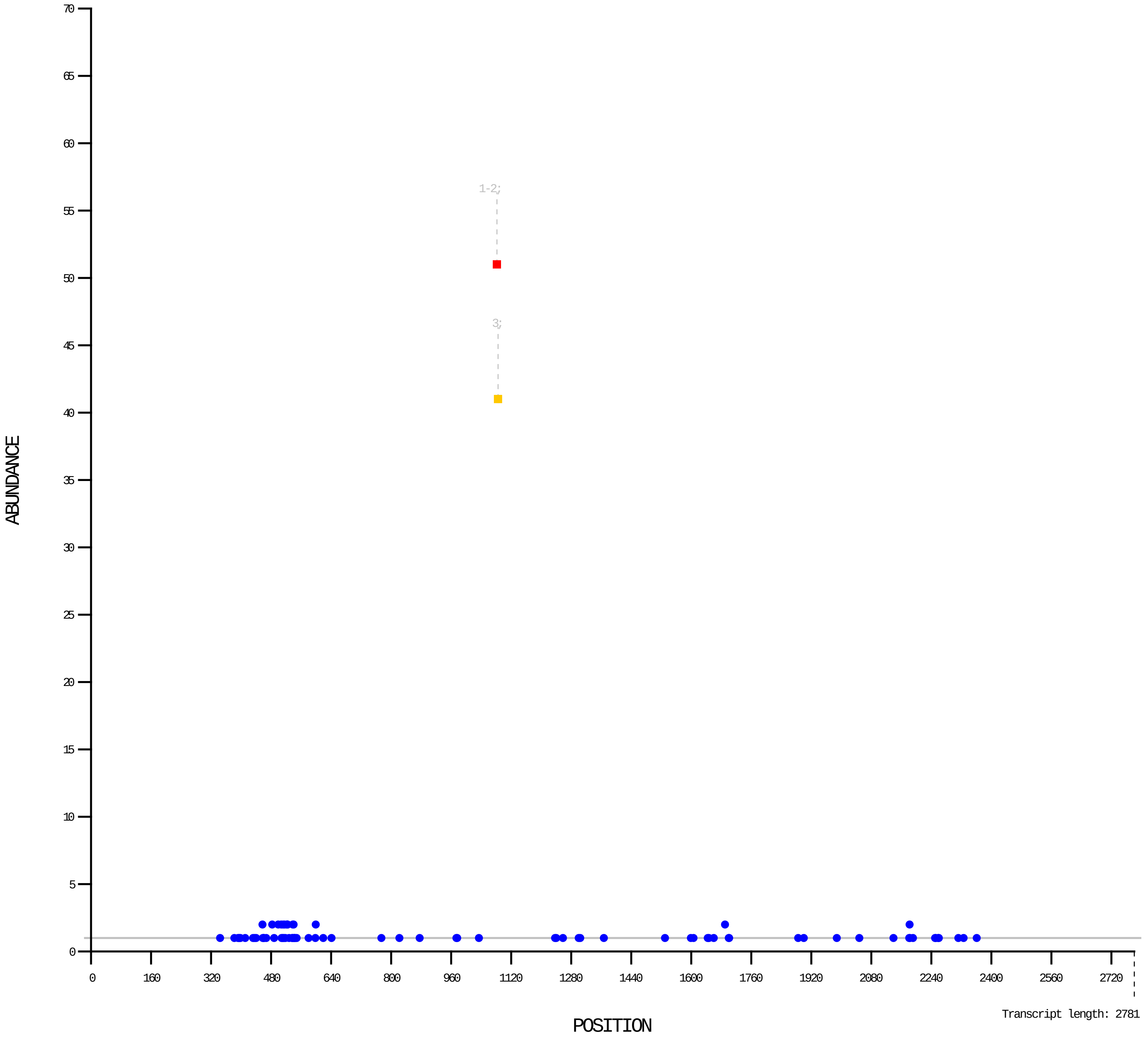

Category: 0 1 2 3 4
Degradome alignment: Median:

0 #1 Position:1082 Abundance: 51.00(deg) 1(sRNA)
5' TTGAGCCGCGCCAATATCTCT 3' ID:
|||||||||||||||||||| Score: 0.0
3' CACTAACTCGGCGCGGTTATAGAGAGCGCGCG 5' p-value: 0.0

0 #2 Position:1082 Abundance: 51.00(deg) 1(sRNA)
5' TTGAGCCGCGTCAATATCTCC 3' ID:
||||||||||o||||||||| Score: 1.5
3' CACTAACTCGGCGCGGTTATAGAGAGCGCGCG 5' p-value: 0.0

2 #3 Position:1085 Abundance: 41.00(deg) 5(sRNA)
5' TGATTGAGCCGTGCCAATATC 3' ID:
|||||||||||o||||||||| Score: 0.5
3' GACCACTAACTCGGCGCGGTTATAGAGAGCGC 5' p-value: 0.0

Si035458m pacid=19679373 locus=Si035458m.g annot-version=v2.1

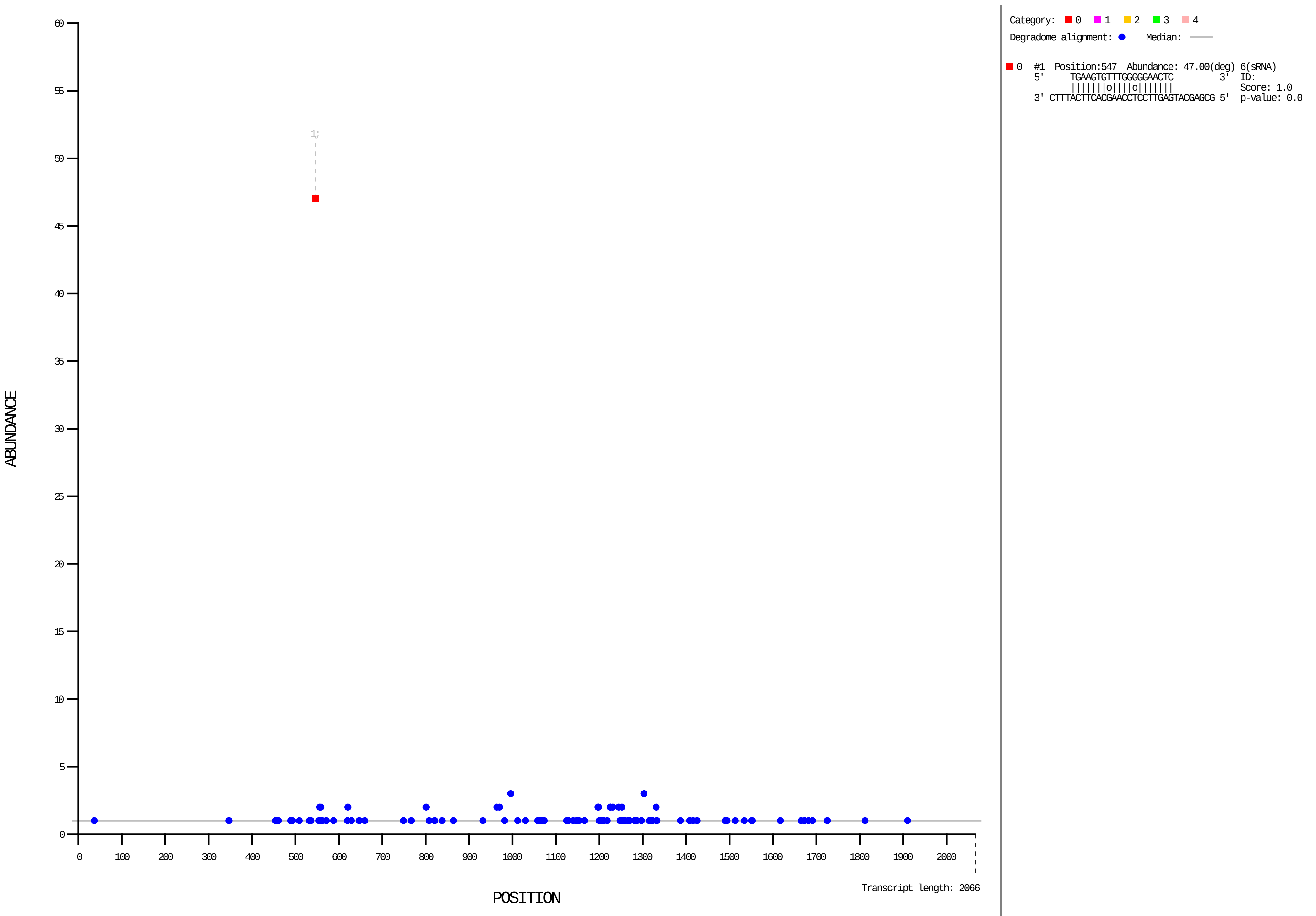

Si006975m pacid=19704071 locus=Si006975m.g annot-version=v2.1

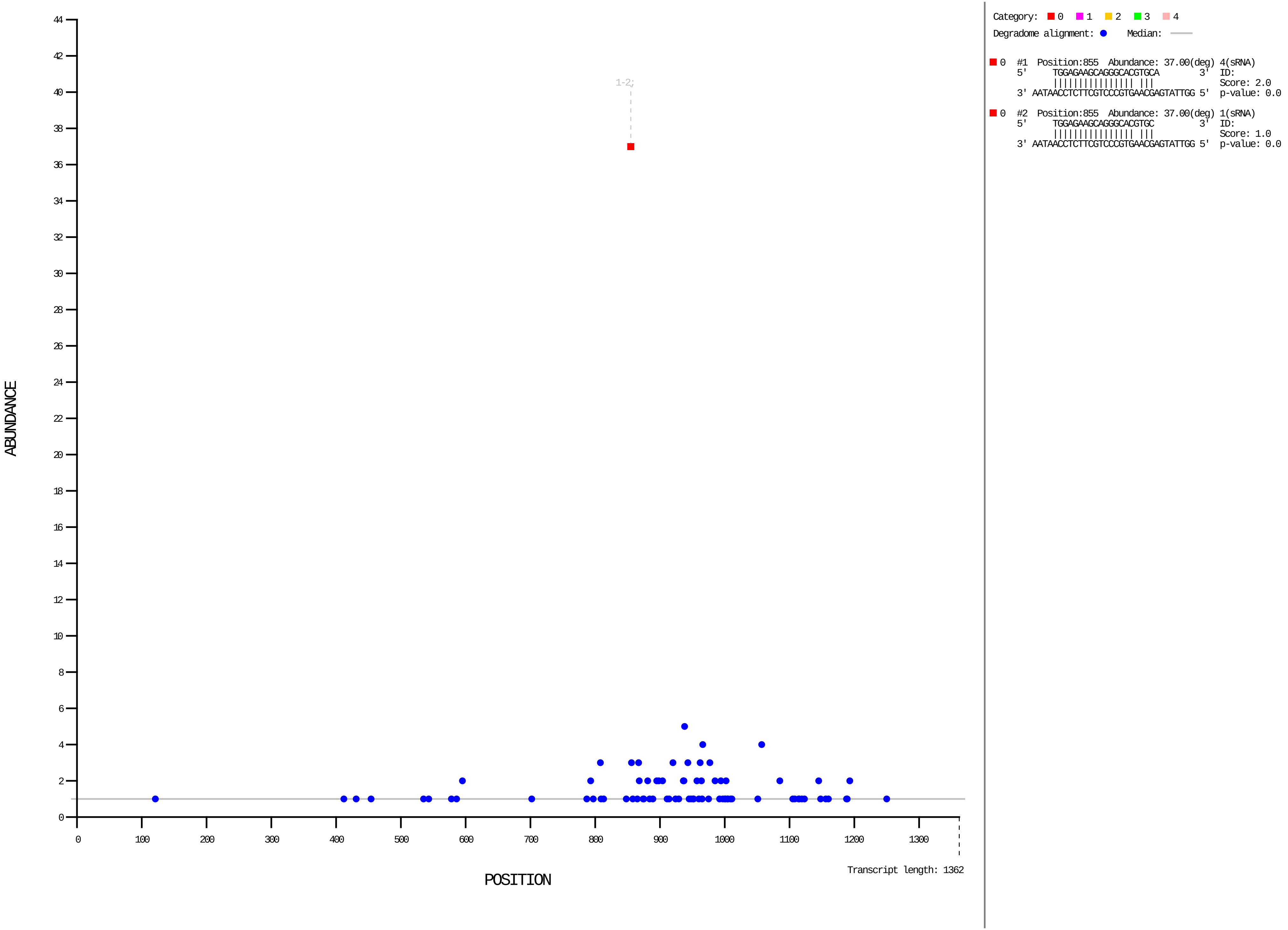

Si034228m pacid=19683970 locus=Si034228m.g annot-version=v2.1

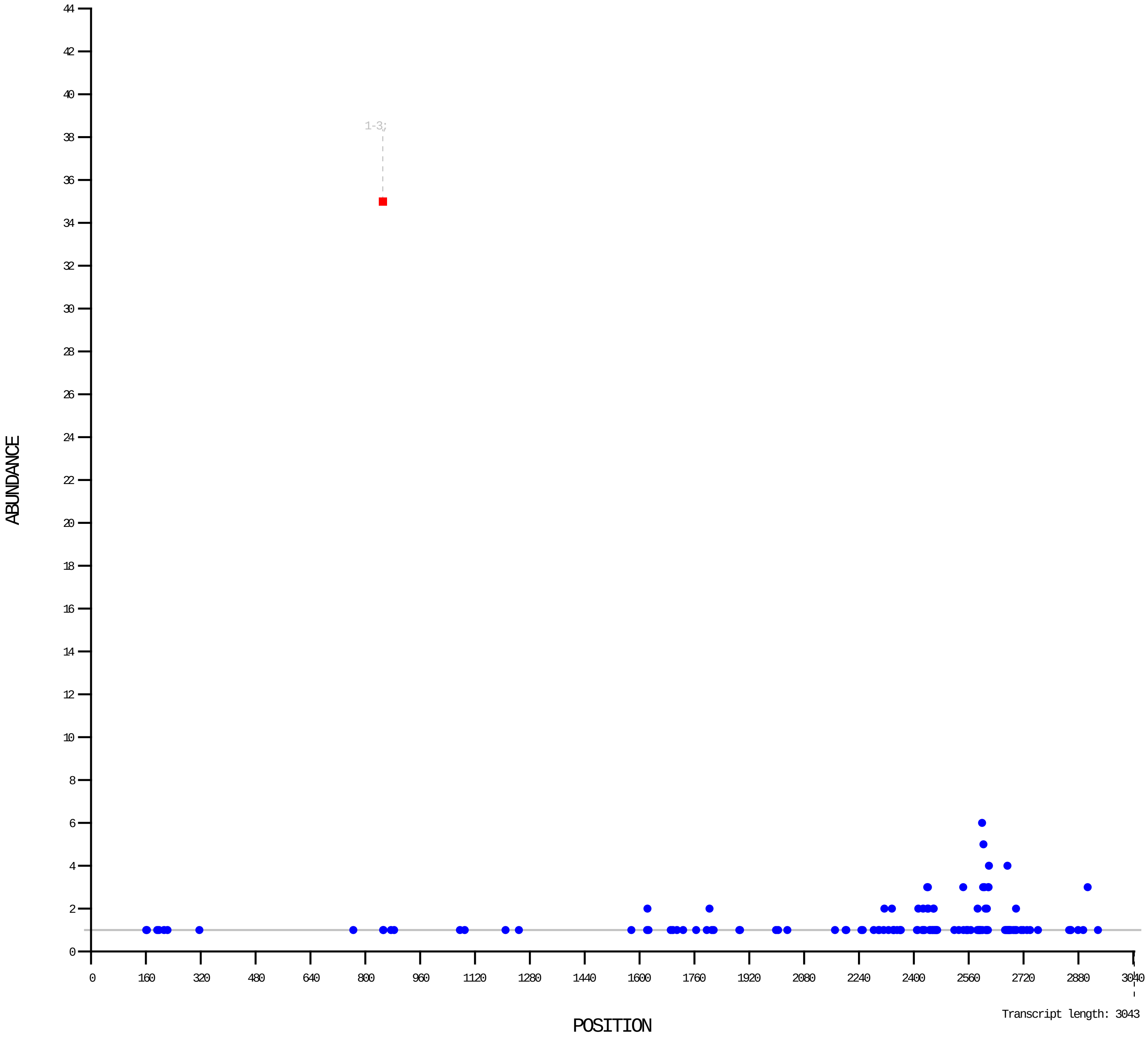


```
0  #1  Position:851  Abundance: 35.00(deg) 7(sRNA)
   5'     TCGGACCAGGCTTCATTCCCC        3'  ID:
          o||||||||||||||| |||             Score: 2.5
   3' CTTAGGCCTGGTCCGAAGTA-GGGTTGGTAAA 5'  p-value: 0.0

0  #2  Position:851  Abundance: 35.00(deg) 2(sRNA)
   5'     TCGGACCAGGCTTCAATCCCT        3'  ID:
          o|||||||||||||| ||||             Score: 2.5
   3' CTTAGGCCTGGTCCGAAGT-AGGGTTGGTAAA 5'  p-value: 0.0

0  #3  Position:851  Abundance: 35.00(deg) 2(sRNA)
   5'     TCGGACCAGGCTTCATTCCTC        3'  ID:
          o|||||||||||||||o||              Score: 3.0
   3' CTTAGGCCTGGTCCGAAGTAGGGTTGGTAAAC 5'  p-value: 0.0
```

Si000907m pacid=19678185 locus=Si000907m.g annot-version=v2.1

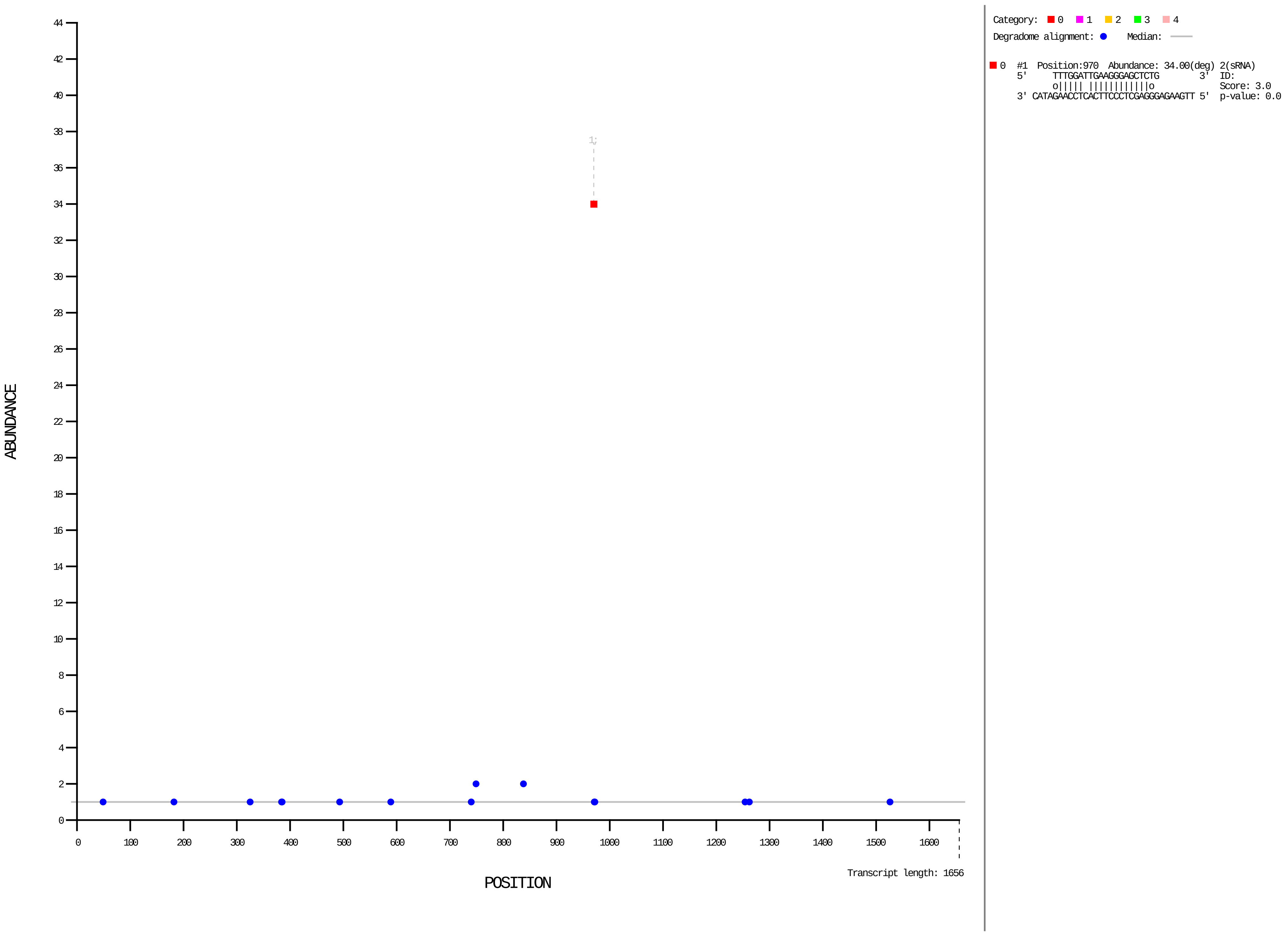

Si034254m pacid=19681172 locus=Si034254m.g annot-version=v2.1

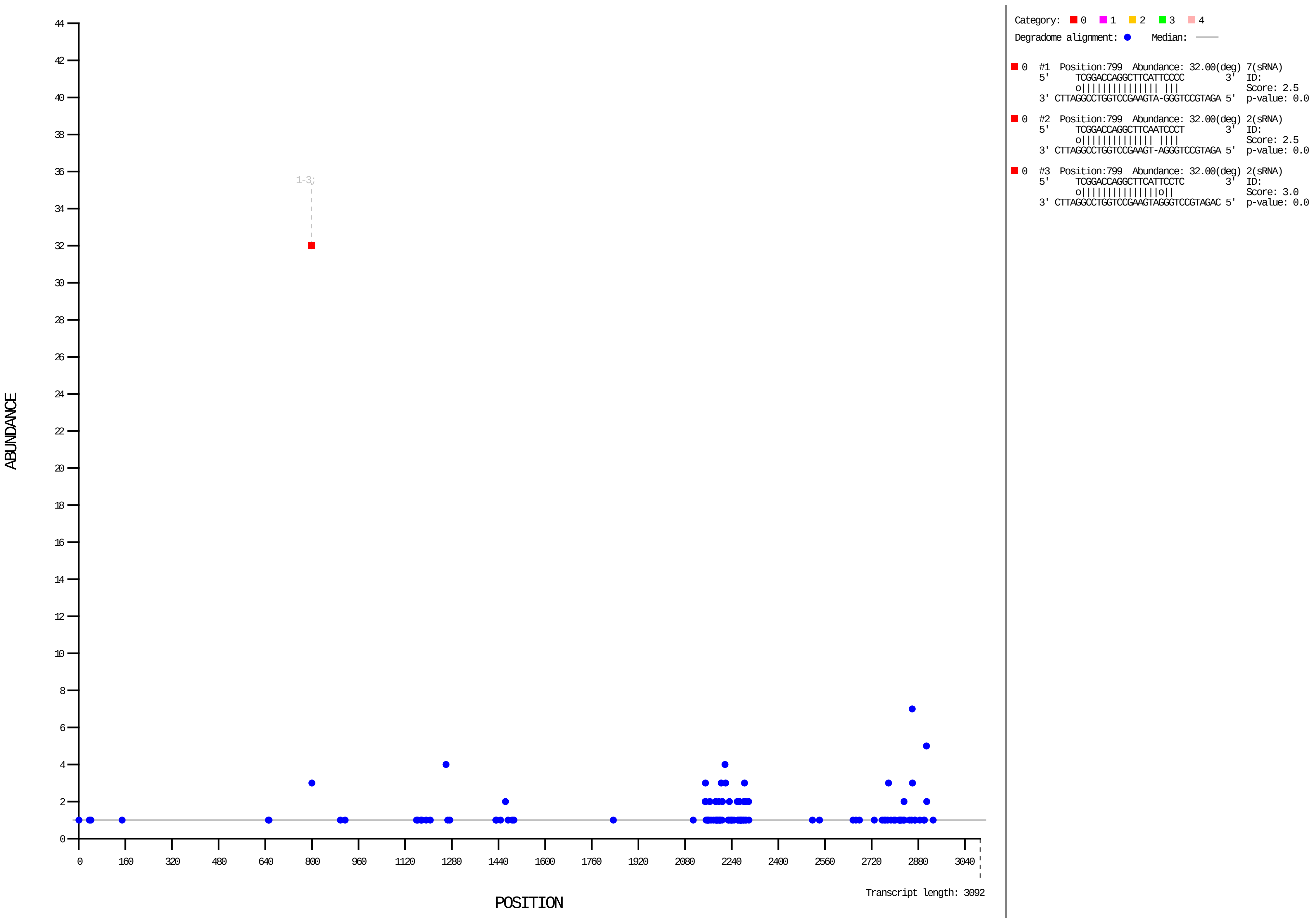

Si022747m pacid=19701137 locus=Si022747m.g annot-version=v2.1

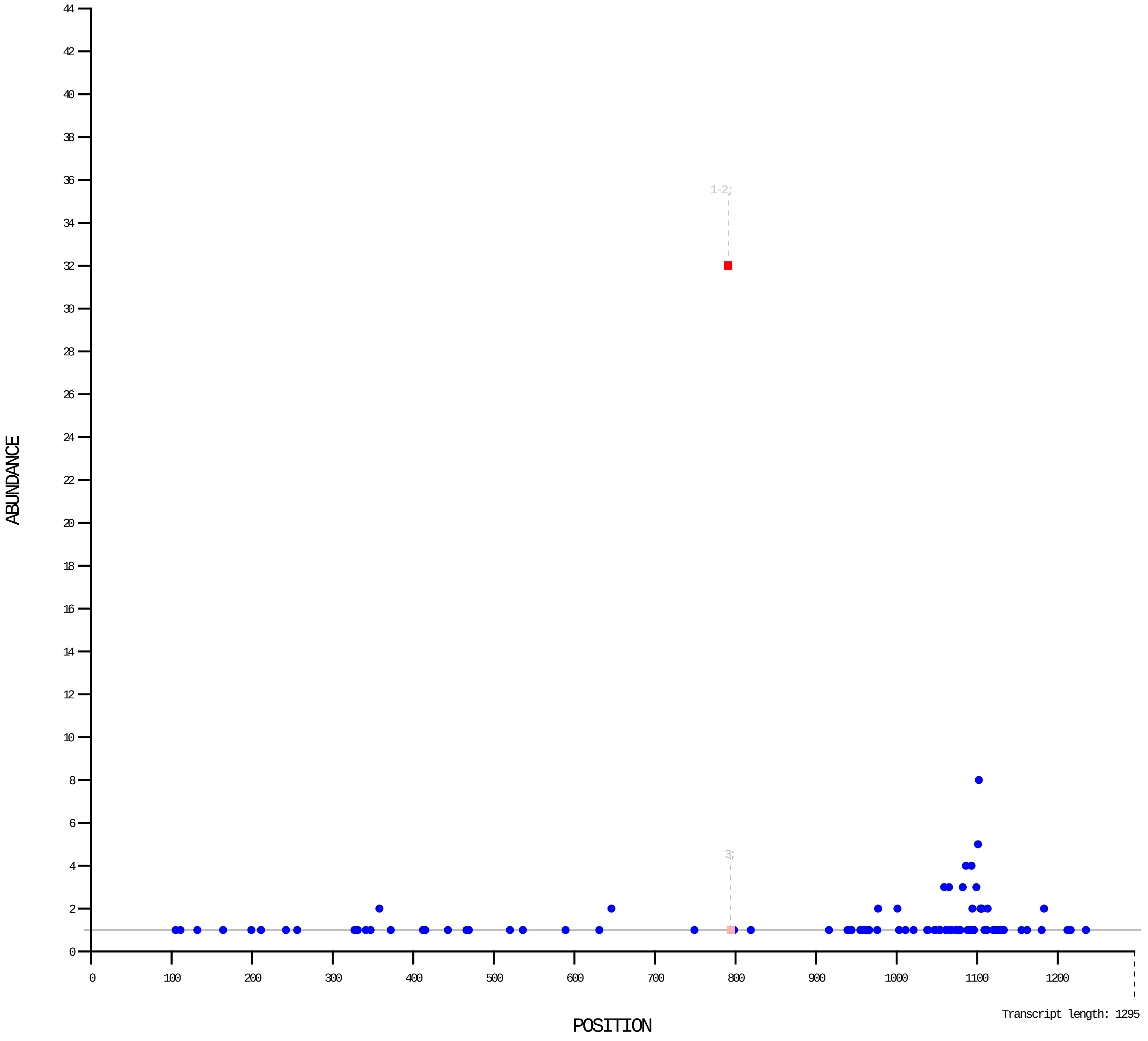


```
Category: 0  1  2  3  4
Degradome alignment:    Median:

0  #1  Position:791  Abundance: 32.00(deg) 4(sRNA)
   5'     TGGAGAAGCAGGGCACGTGCA        3'  ID:
          ||||||||||||||||| |||            Score: 2.0
   3' TTCGACCTCTTCGTCCCGTGGACGAGCTAACG 5'  p-value: 0.0

0  #2  Position:791  Abundance: 32.00(deg) 1(sRNA)
   5'     TGGAGAAGCAGGGCACGTGC         3'  ID:
          ||||||||||||||||| |||            Score: 1.0
   3' TTCGACCTCTTCGTCCCGTGGACGAGCTAACG 5'  p-value: 0.0

4  #3  Position:794  Abundance: 1.00(deg) 1(sRNA)
   5'     TGCTGGAGAAGCAGGGCACGT        3'  ID:
           |||||||||||||||||| |            Score: 2.0
   3' AACTTCGACCTCTTCGTCCCGTGGACGAGCTA 5'  p-value: 0.01
```

Si006258m pacid=19703625 locus=Si006258m.g annot-version=v2.1

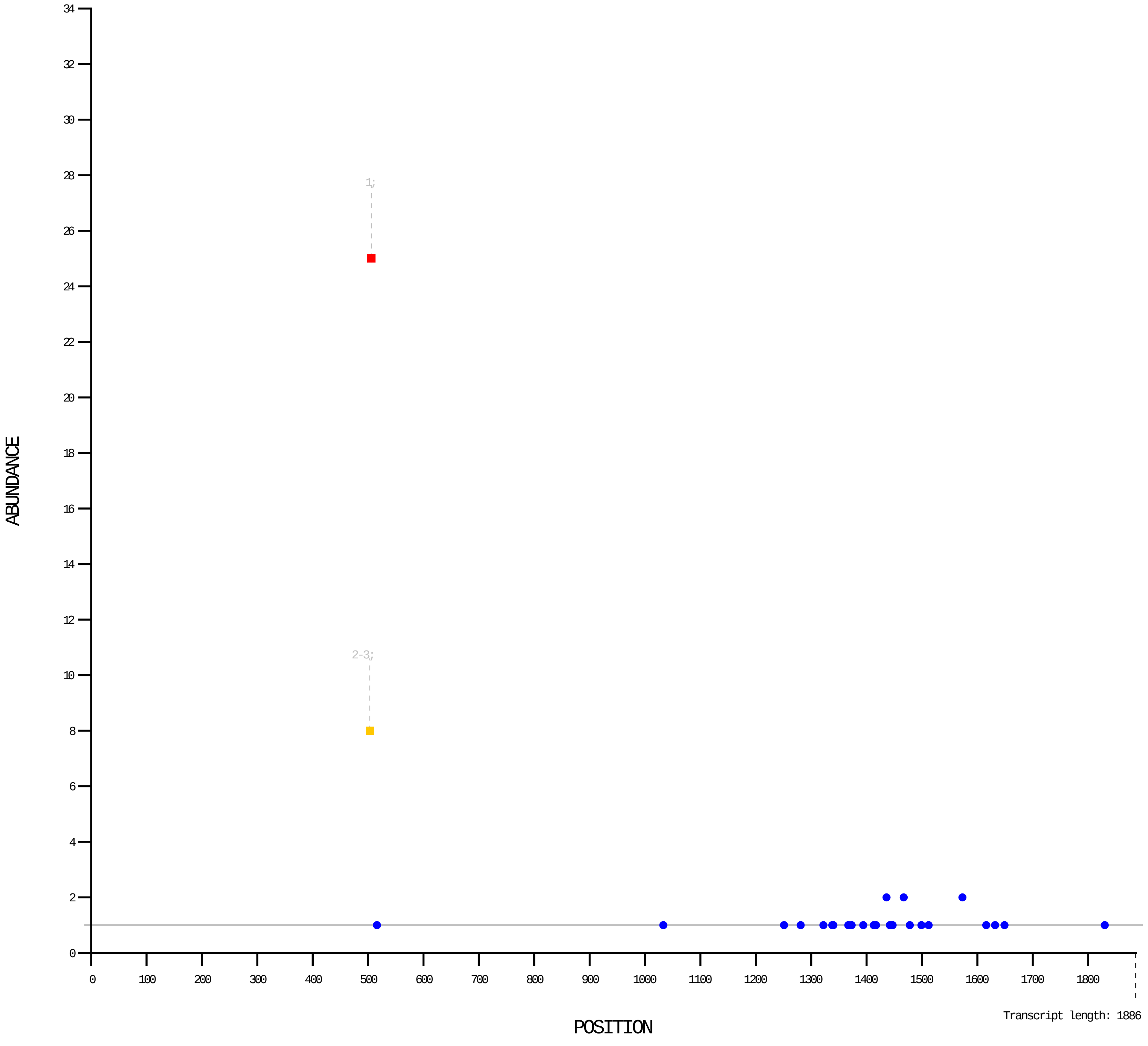

Category: 0 1 2 3 4
Degradome alignment: ● Median: —

```
0  #1  Position:506  Abundance: 25.00(deg) 5(sRNA)
   5'     TGATTGAGCCGTGCCAATATC         3'  ID:
          |||||||||||o|||||||||             Score: 0.5
   3' GGCCACTAACTCGGCGCGGTTATAGAGCGCGC 5'  p-value: 0.0

2  #2  Position:503  Abundance: 8.00(deg) 1(sRNA)
   5'     TTGAGCCGCGCCAATATCTCT         3'  ID:
          |||||||||||||||||||||             Score: 1.0
   3' CACTAACTCGGCGCGGTTATAGAGCGCGCGCG 5'  p-value: 0.0

2  #3  Position:503  Abundance: 8.00(deg) 1(sRNA)
   5'     TTGAGCCGCGTCAATATCTCC         3'  ID:
          ||||||||||o||||||||||             Score: 1.5
   3' CACTAACTCGGCGCGGTTATAGAGCGCGCGCG 5'  p-value: 0.01
```

Si021562m pacid=19701213 locus=Si021562m.g annot-version=v2.1

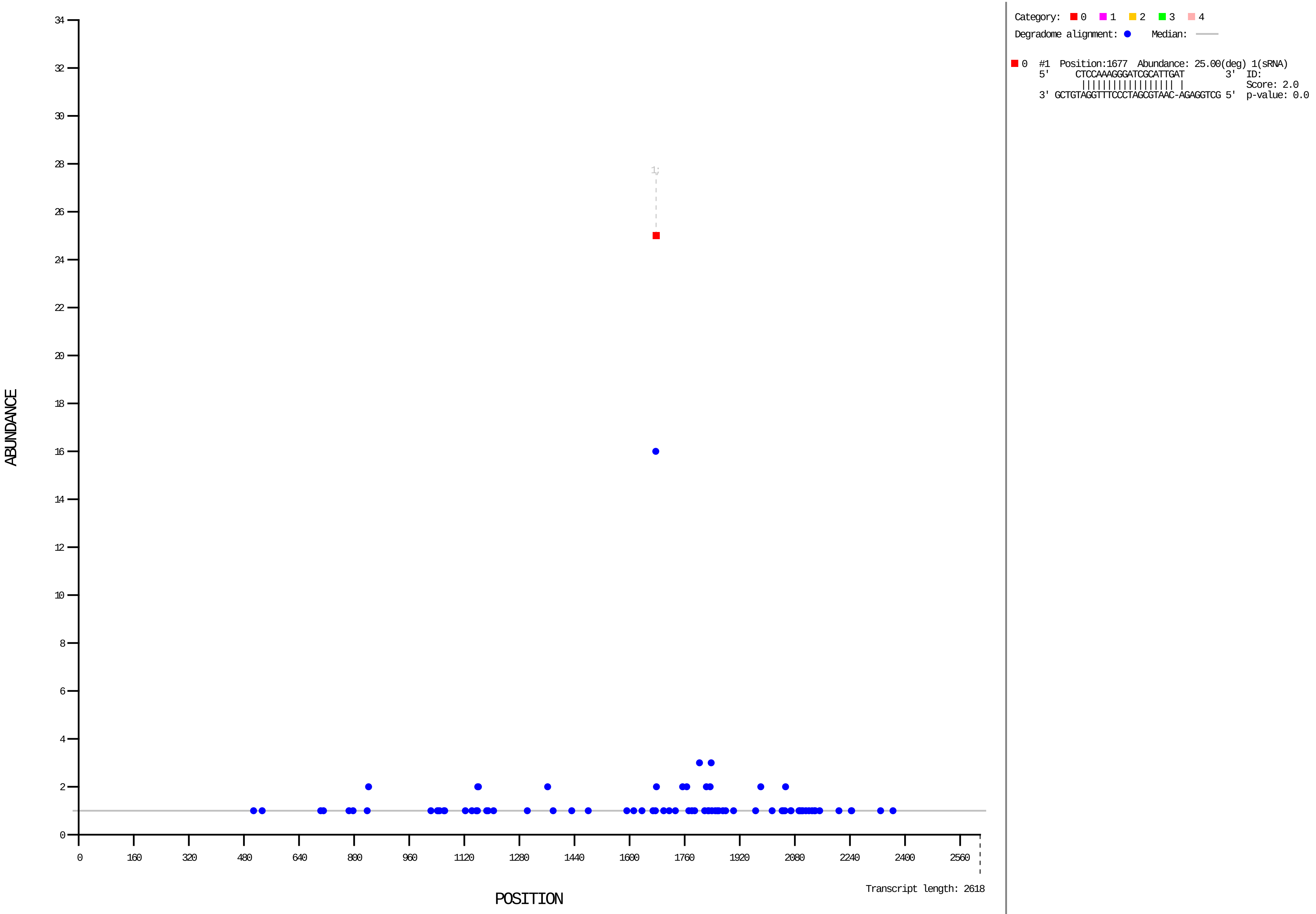

Si034822m pacid=19680013 locus=Si034822m.g annot-version=v2.1

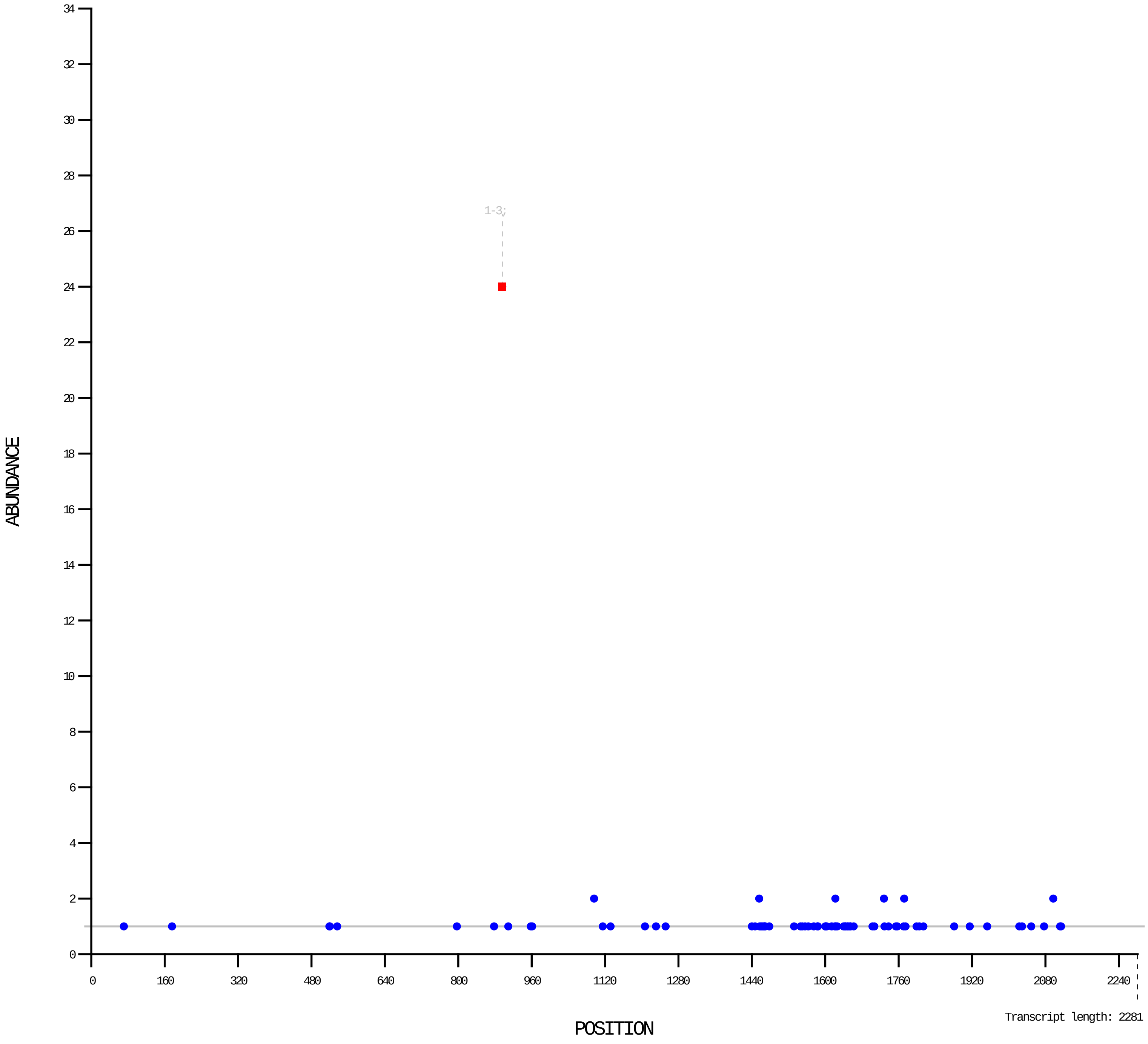

Category: 0 1 2 3 4
Degradome alignment: Median:

0 #1 Position:896 Abundance: 24.00(deg) 2(sRNA)
```
5'    TTCCACA-GCTTTCTTGAACTG        3'  ID:
      ||||||| |||||||||||| o            Score: 2.5
3' CGGAAGGTGTACGAAAGAACTTGCTACCGCAG 5'  p-value: 0.0
```

0 #2 Position:896 Abundance: 24.00(deg) 1(sRNA)
```
5'     TCCACAGGCTTTCTTGAACTG        3'  ID:
       |||||| |||||||||||| |            Score: 2.0
3' CGGAAGGTGTACGAAAGAACTTG-CTACCGCA 5'  p-value: 0.0
```

0 #3 Position:896 Abundance: 24.00(deg) 1(sRNA)
```
5'    TTCCACA-GCTTTCTTGAACTT        3'  ID:
      ||||||| ||||||||||||              Score: 3.0
3' CGGAAGGTGTACGAAAGAACTTGCTACCGCAG 5'  p-value: 0.01
```

Si009541m pacid=19694259 locus=Si009541m.g annot-version=v2.1

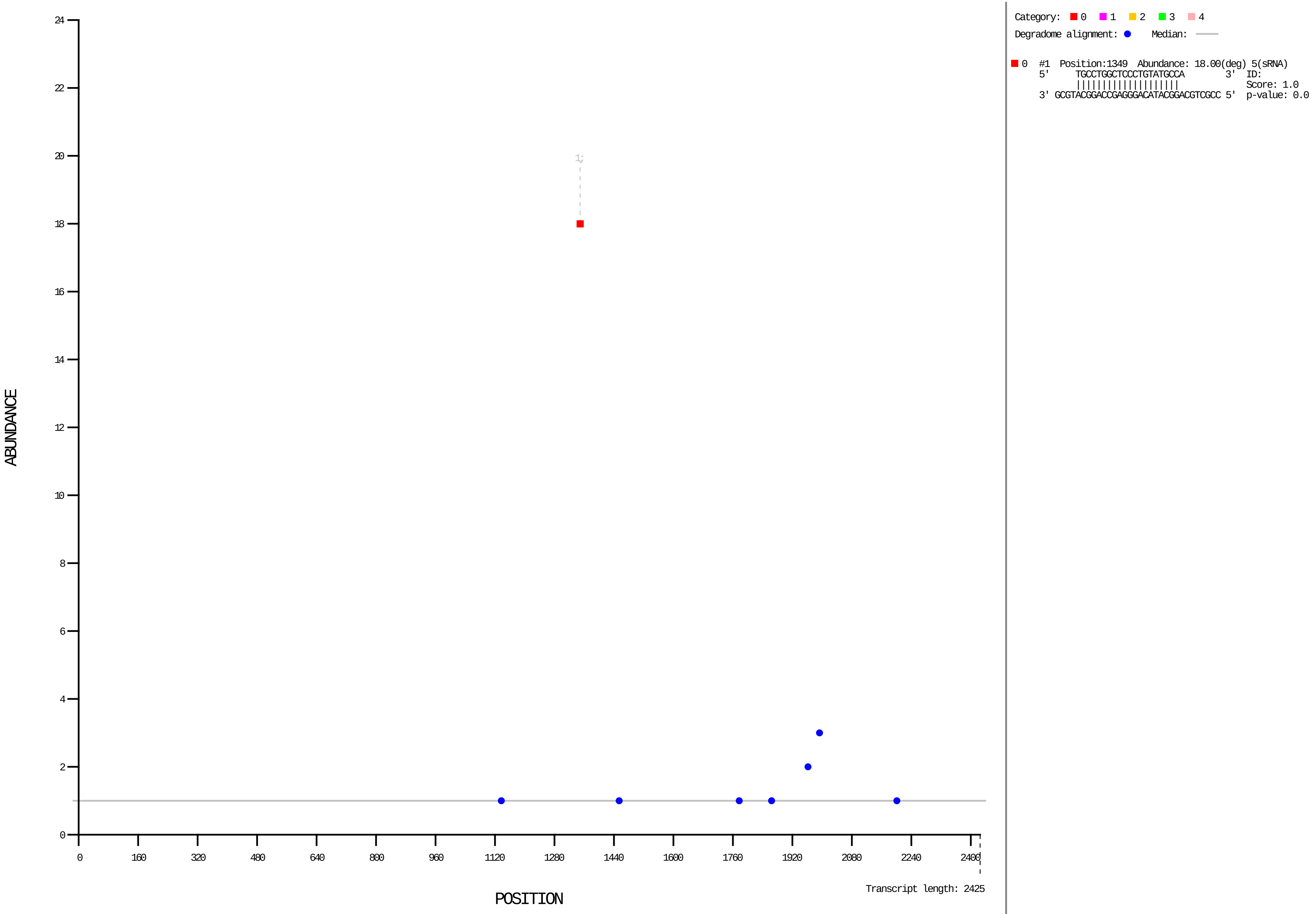

Si011853m pacid=19694602 locus=Si011853m.g annot-version=v2.1

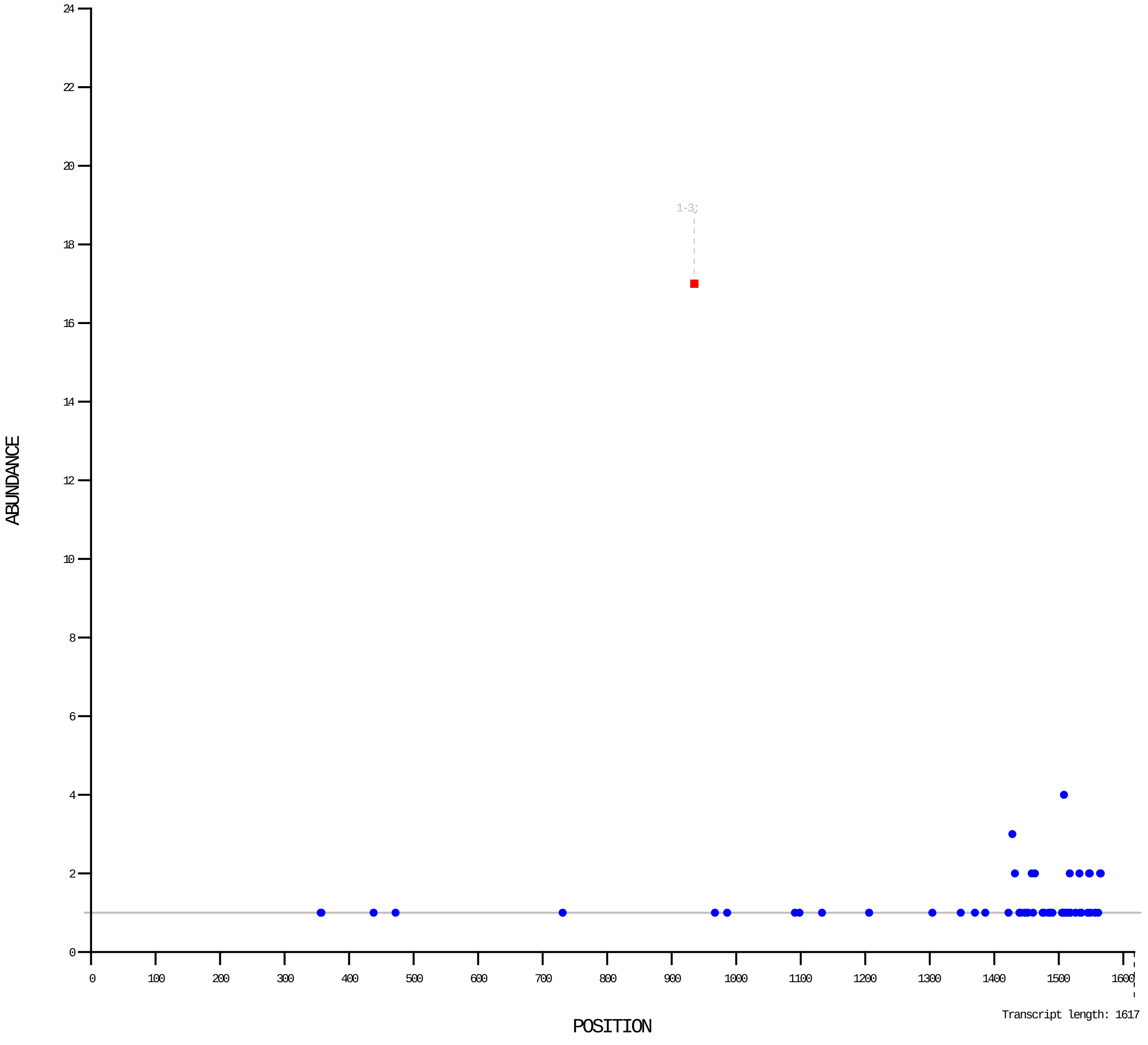


```
Category: 0  1  2  3  4
Degradome alignment:      Median:

0   #1  Position:935  Abundance: 17.00(deg) 2(sRNA)
    5'    TTCCACA-GCTTTCTTGAACTG        3'  ID:
          ||||||| ||||||||||||| |           Score: 2.0
    3' GCGAAGGTGTCCGAAAGAACTTGCCAACGCCG 5'  p-value: 0.0

0   #2  Position:935  Abundance: 17.00(deg) 1(sRNA)
    5'     TCCACAGGCTTTCTTGAACTG        3'  ID:
           ||||||||||||||||||||| |          Score: 1.0
    3' GCGAAGGTGTCCGAAAGAACTTGCCAACGCCG 5'  p-value: 0.0

0   #3  Position:935  Abundance: 17.00(deg) 1(sRNA)
    5'    TTCCACA-GCTTTCTTGAACTT        3'  ID:
          ||||||| |||||||||||||             Score: 3.0
    3' GCGAAGGTGTCCGAAAGAACTTGCCAACGCCG 5'  p-value: 0.0
```

Si001465m pacid=19676000 locus=Si001465m.g annot-version=v2.1

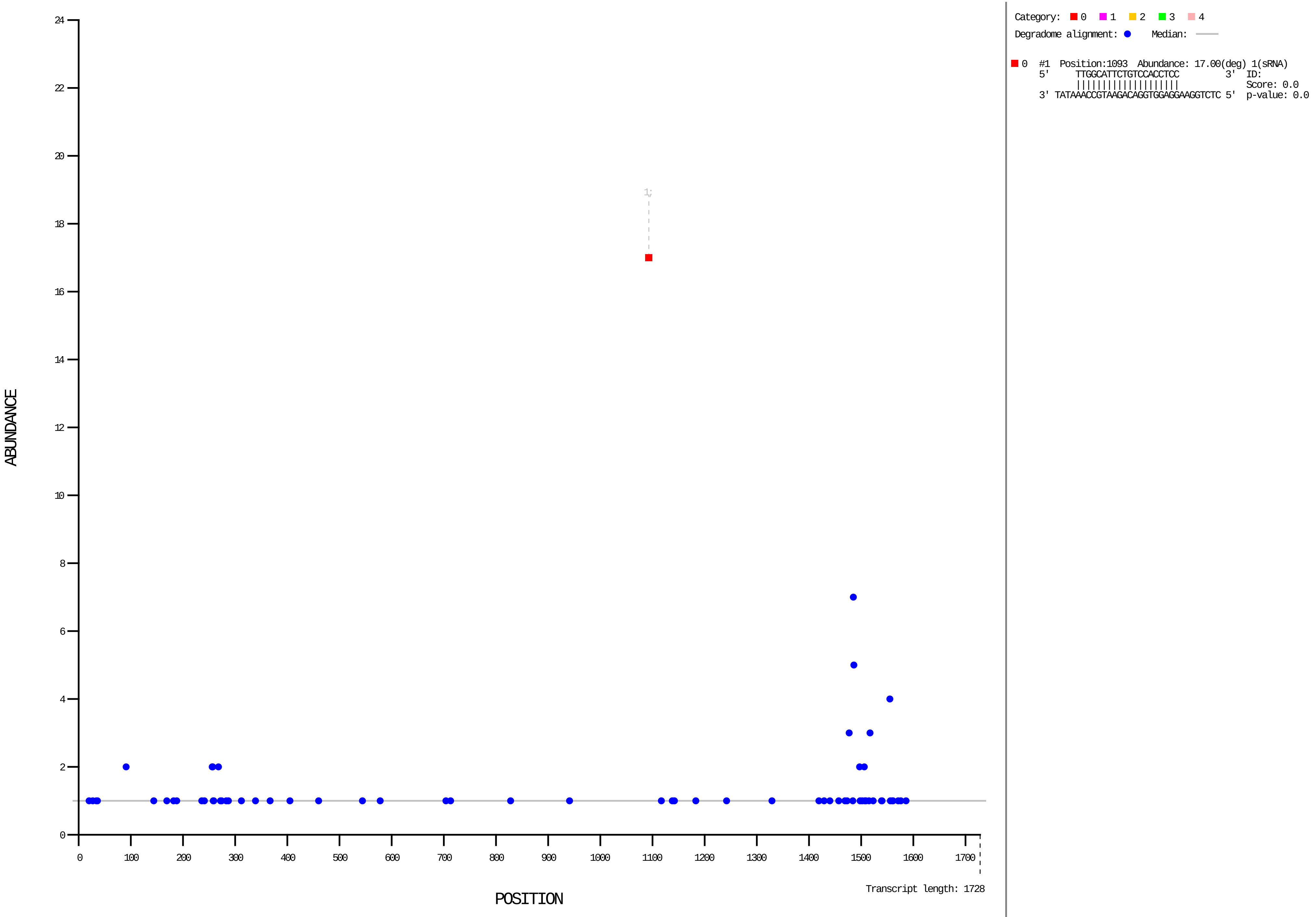

Si021833m pacid=19701328 locus=Si021833m.g annot-version=v2.1

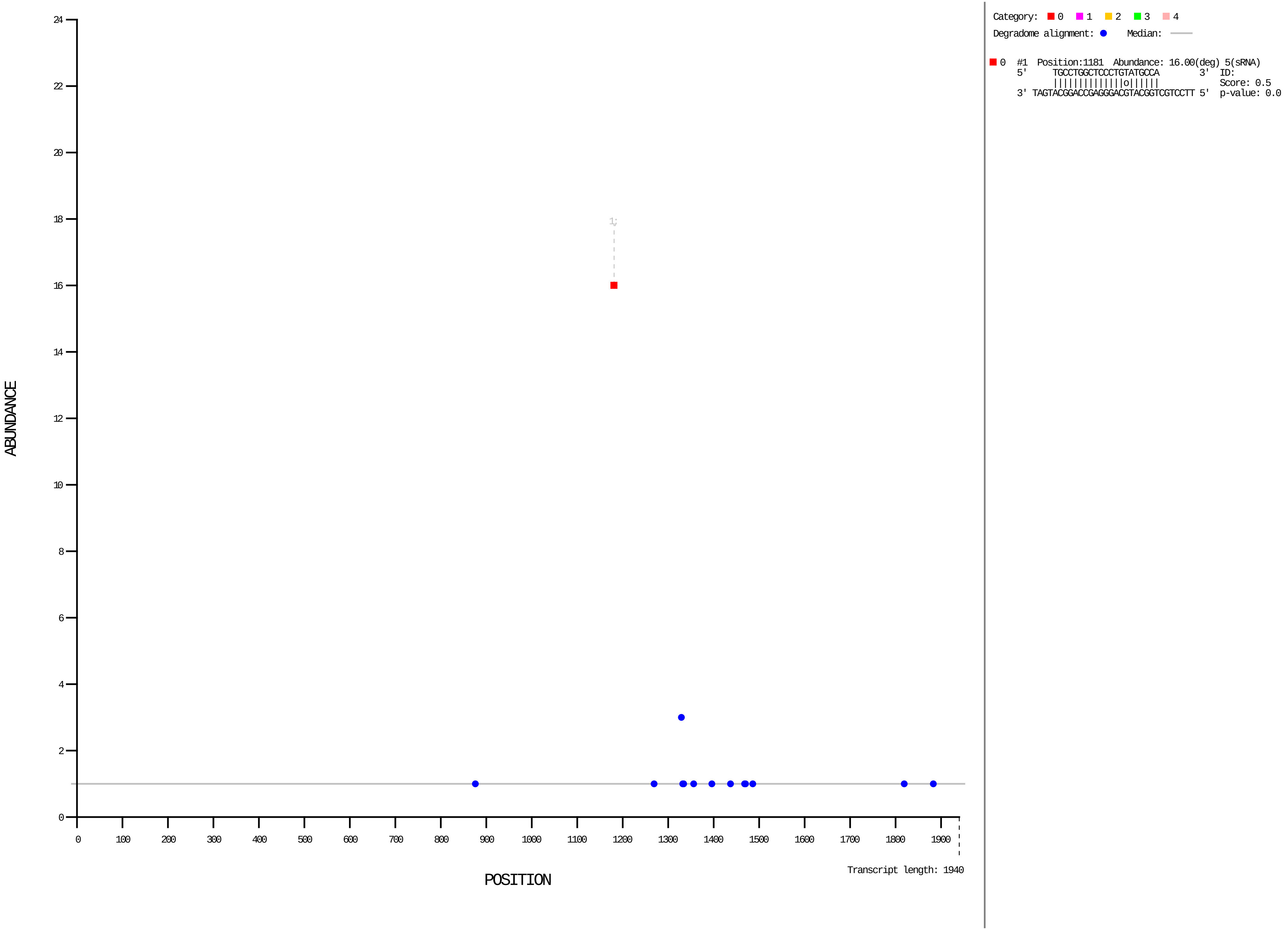

Si039098m pacid=19683057 locus=Si039098m.g annot-version=v2.1

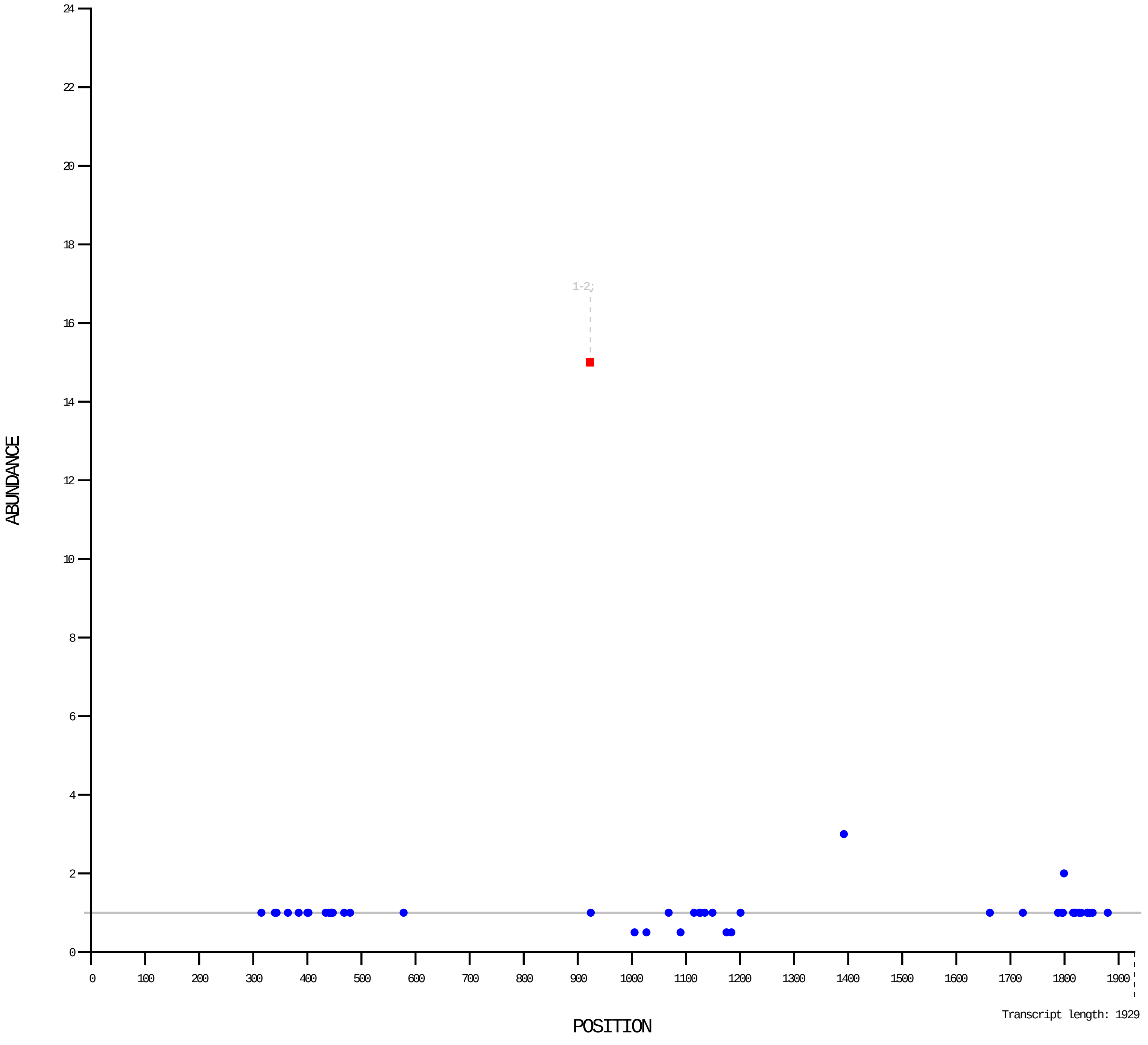

Category: 0 1 2 3 4
Degradome alignment: Median:

```
0  #1  Position:923  Abundance: 15.00(deg) 1(sRNA)
   5'     TTGAGCCGCGCCAATATCTCT         3'  ID:
          |||||||||||||||||||||             Score: 1.0
   3' TATTAACTCGGCGCGGTTATAGAGCGCCCGCG 5'  p-value: 0.0

0  #2  Position:923  Abundance: 15.00(deg) 1(sRNA)
   5'     TTGAGCCGCGTCAATATCTCC         3'  ID:
          ||||||||||o||||||||||             Score: 1.5
   3' TATTAACTCGGCGCGGTTATAGAGCGCCCGCG 5'  p-value: 0.0
```

Si029871m pacid=19712002 locus=Si029871m.g annot-version=v2.1

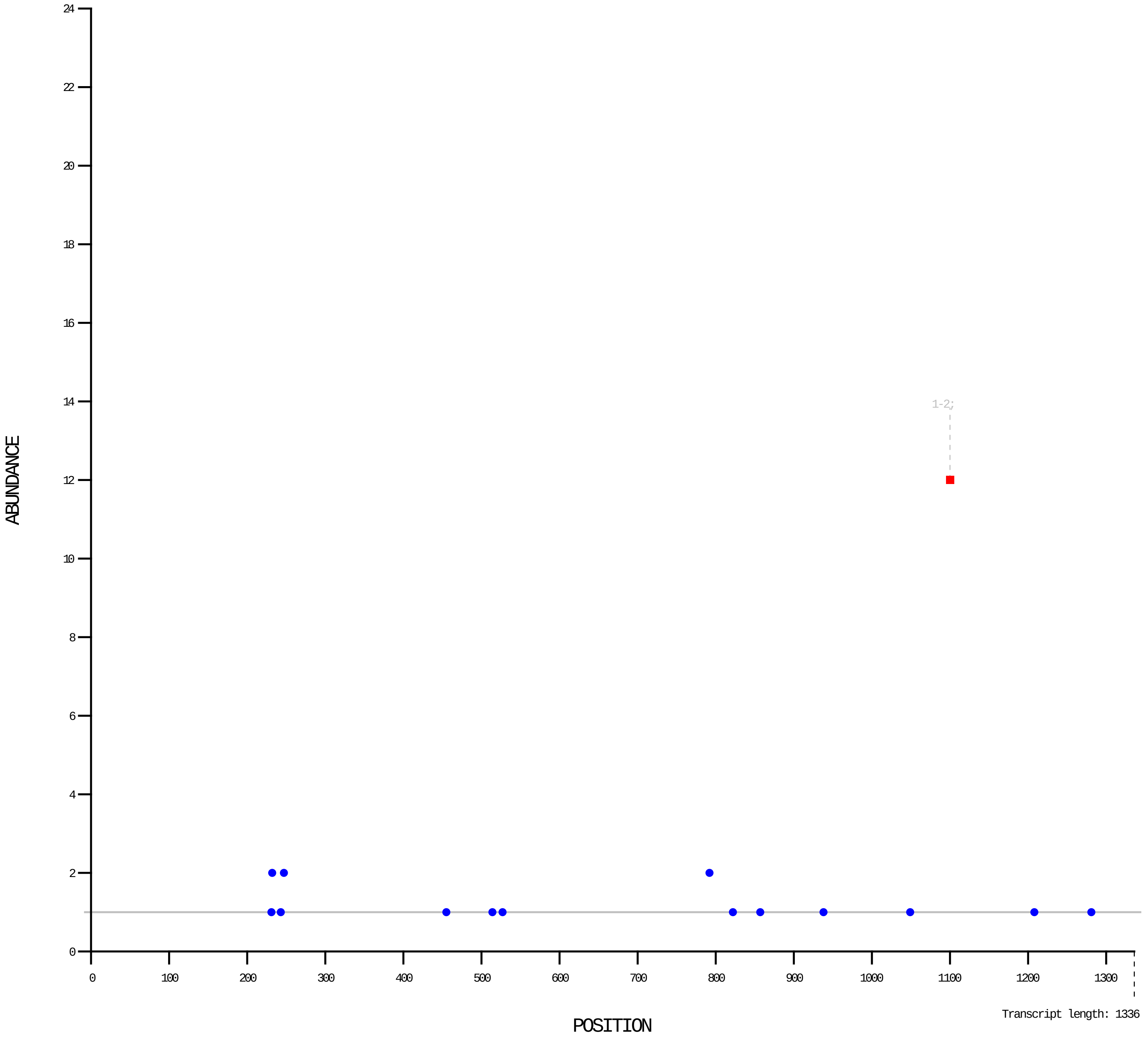


```
Category: 0 1 2 3 4
Degradome alignment:     Median:

0  #1  Position:1100  Abundance: 12.00(deg) 1(sRNA)
   5'     TTGGACTGAAGGGTGCTCCCT        3'  ID:
          |||||||||||||| |o||||            Score: 1.5
   3' GCTTAACCTGACTTCCCA-GGGGGATGACAAC 5'  p-value: 0.0

0  #2  Position:1100  Abundance: 12.00(deg) 1(sRNA)
   5'     TTGGATTGAAGGGAGCTCC          3'  ID:
          |||||o|||||||  |o||              Score: 3.0
   3' GCTTAACCTGACTTCCCAGGGGGATGACAACT 5'  p-value: 0.03
```

Si017749m pacid=19689830 locus=Si017749m.g annot-version=v2.1

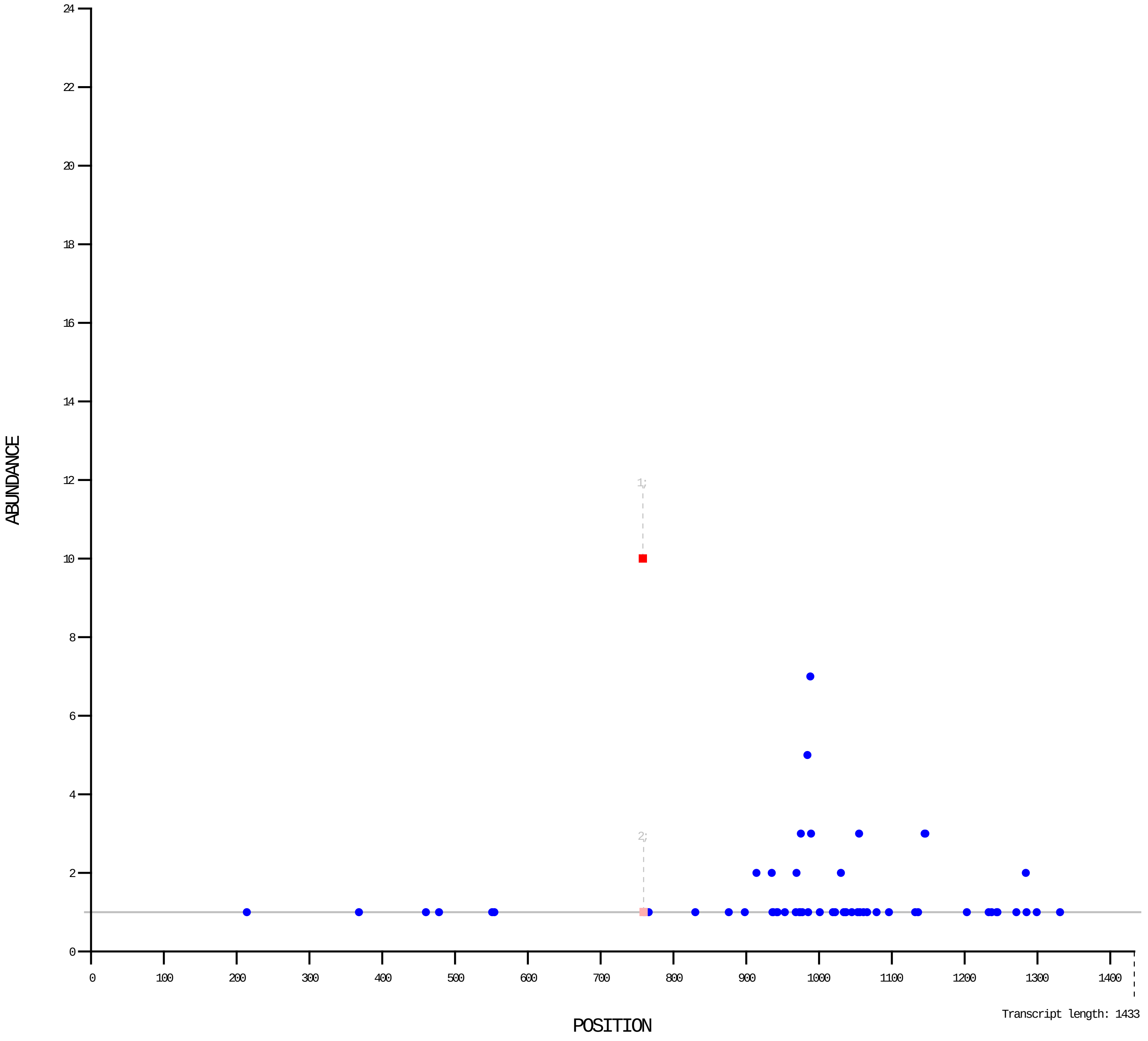

Category: 0 1 2 3 4
Degradome alignment: Median:

```
0   #1  Position:758  Abundance: 10.00(deg) 8(sRNA)
    5'     TGACAGAAGAGAGTGAGCAC         3'  ID:
           ||||||||||||| ||||||             Score: 1.0
    3' GTCGACTGTCTTCTCTCTCTCGTGCGACCTCT 5'  p-value: 0.0

4   #2  Position:759  Abundance: 1.00(deg) 1(sRNA)
    5'     TTGACAGAAGAGAGCGAGCAC        3'  ID:
           o||||||||||||| ||||||            Score: 1.5
    3' GGTCGACTGTCTTCTCTCTCTCGTGCGACCTC 5'  p-value: 0.0
```

Si010553m pacid=19694091 locus=Si010553m.g annot-version=v2.1

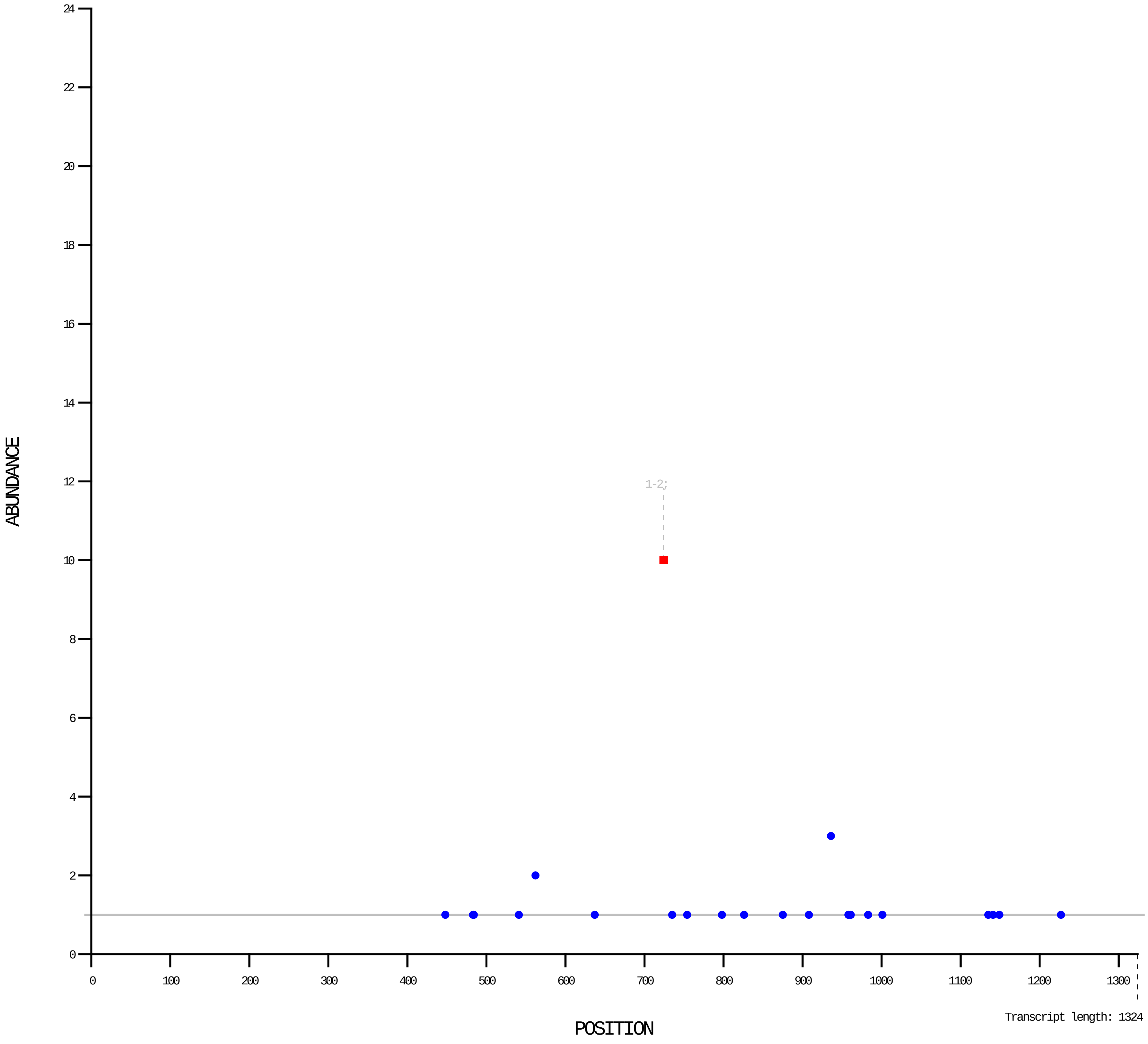

Category: 0 1 2 3 4
Degradome alignment: Median:

0 #1 Position:724 Abundance: 10.00(deg) 4(sRNA)
5' TGGAGAAGCAGGGCACGTGCA 3' ID:
|||||||||||| |||||| Score: 3.0
3' CGCAACCTCTTCGTCCAGTGCACACGGCGGCG 5' p-value: 0.0

0 #2 Position:724 Abundance: 10.00(deg) 1(sRNA)
5' TGGAGAAGCAGGGCACGTGC 3' ID:
|||||||||||| |||||| Score: 2.0
3' CGCAACCTCTTCGTCCAGTGCACACGGCGGCG 5' p-value: 0.0

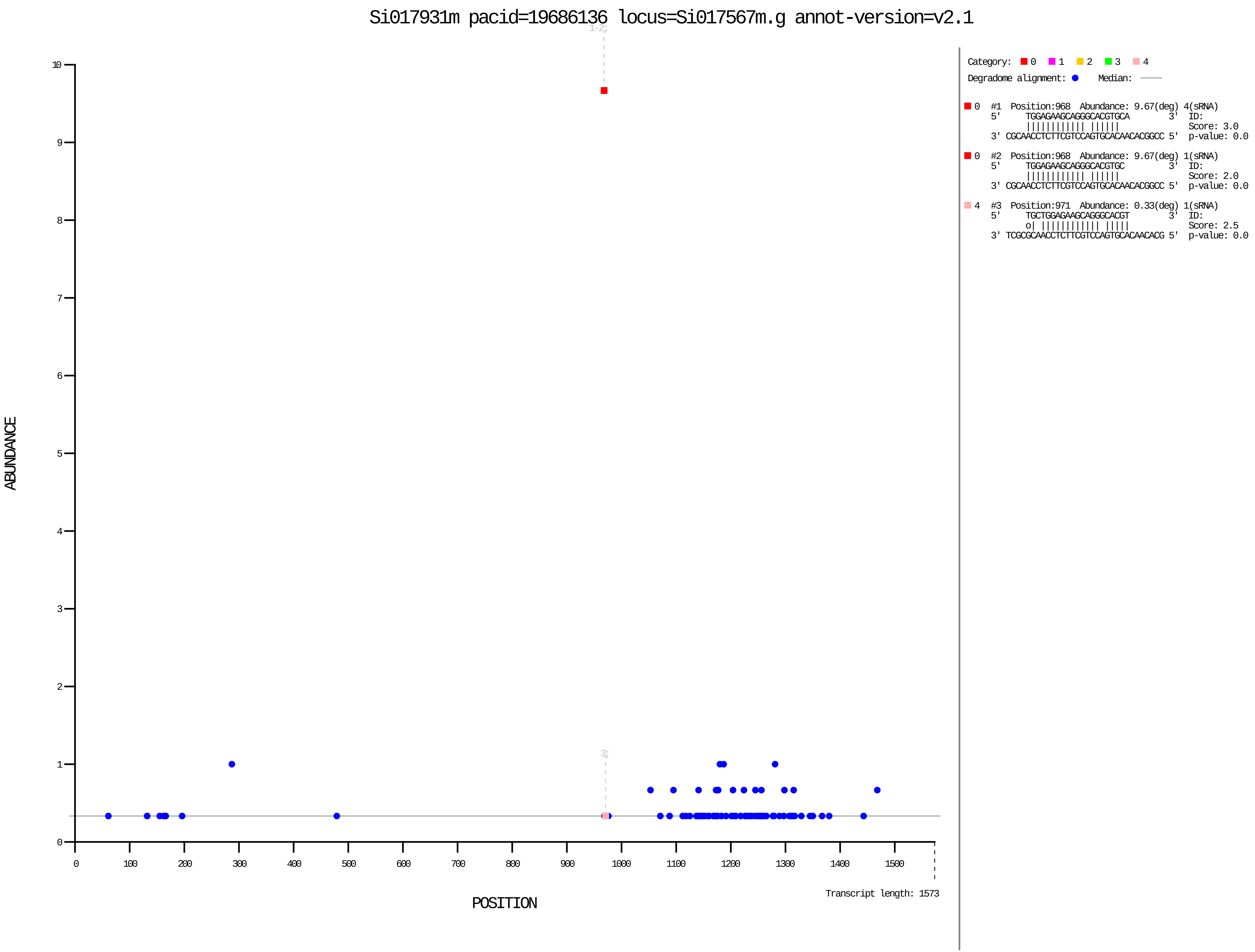

Si017931m pacid=19686136 locus=Si017567m.g annot-version=v2.1
ABUNDANCE
POSITION
Transcript length: 1573
Category: 0 1 2 3 4
Degradome alignment:
Median:
0 #1 Position:968 Abundance: 9.67(deg) 4(sRNA)
5' TGGAGAAGCAGGGCACGTGCA 3' ID:
Score: 3.0
3' CGCAACCTCTTCGTCCAGTGCACAACACGGCC 5' p-value: 0.0
0 #2 Position:968 Abundance: 9.67(deg) 1(sRNA)
5' TGGAGAAGCAGGGCACGTGC 3' ID:
Score: 2.0
3' CGCAACCTCTTCGTCCAGTGCACAACACGGCC 5' p-value: 0.0
4 #3 Position:971 Abundance: 0.33(deg) 1(sRNA)
5' TGCTGGAGAAGCAGGGCACGT 3' ID:
Score: 2.5
3' TCGCGCAACCTCTTCGTCCAGTGCACAACACG 5' p-value: 0.0

Si017570m pacid=19686135 locus=Si017567m.g annot-version=v2.1

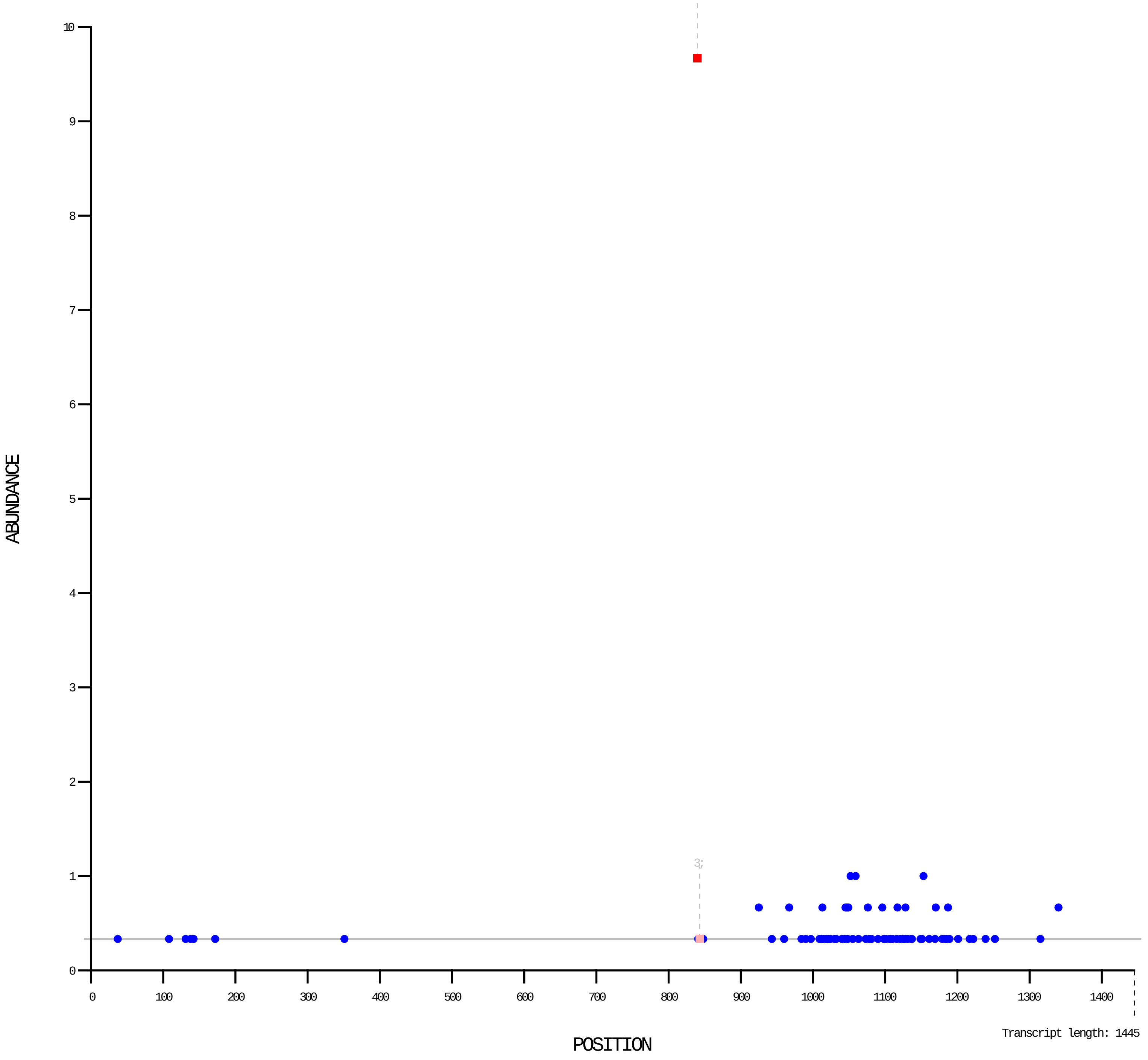

Category: 0 1 2 3 4
Degradome alignment: Median:

0 #1 Position:840 Abundance: 9.67(deg) 4(sRNA)
```
5'      TGGAGAAGCAGGGCACGTGCA        3'  ID:
        |||||||||||| ||||||              Score: 3.0
3' CGCAACCTCTTCGTCCAGTGCACAACACGGCC 5'  p-value: 0.0
```

0 #2 Position:840 Abundance: 9.67(deg) 1(sRNA)
```
5'      TGGAGAAGCAGGGCACGTGC         3'  ID:
        |||||||||||| ||||||              Score: 2.0
3' CGCAACCTCTTCGTCCAGTGCACAACACGGCC 5'  p-value: 0.0
```

4 #3 Position:843 Abundance: 0.33(deg) 1(sRNA)
```
5'      TGCTGGAGAAGCAGGGCACGT        3'  ID:
        o| |||||||||||| |||||            Score: 2.5
3' TCGCGCAACCTCTTCGTCCAGTGCACAACACG 5'  p-value: 0.05
```

Si017567m pacid=19686134 locus=Si017567m.g annot-version=v2.1

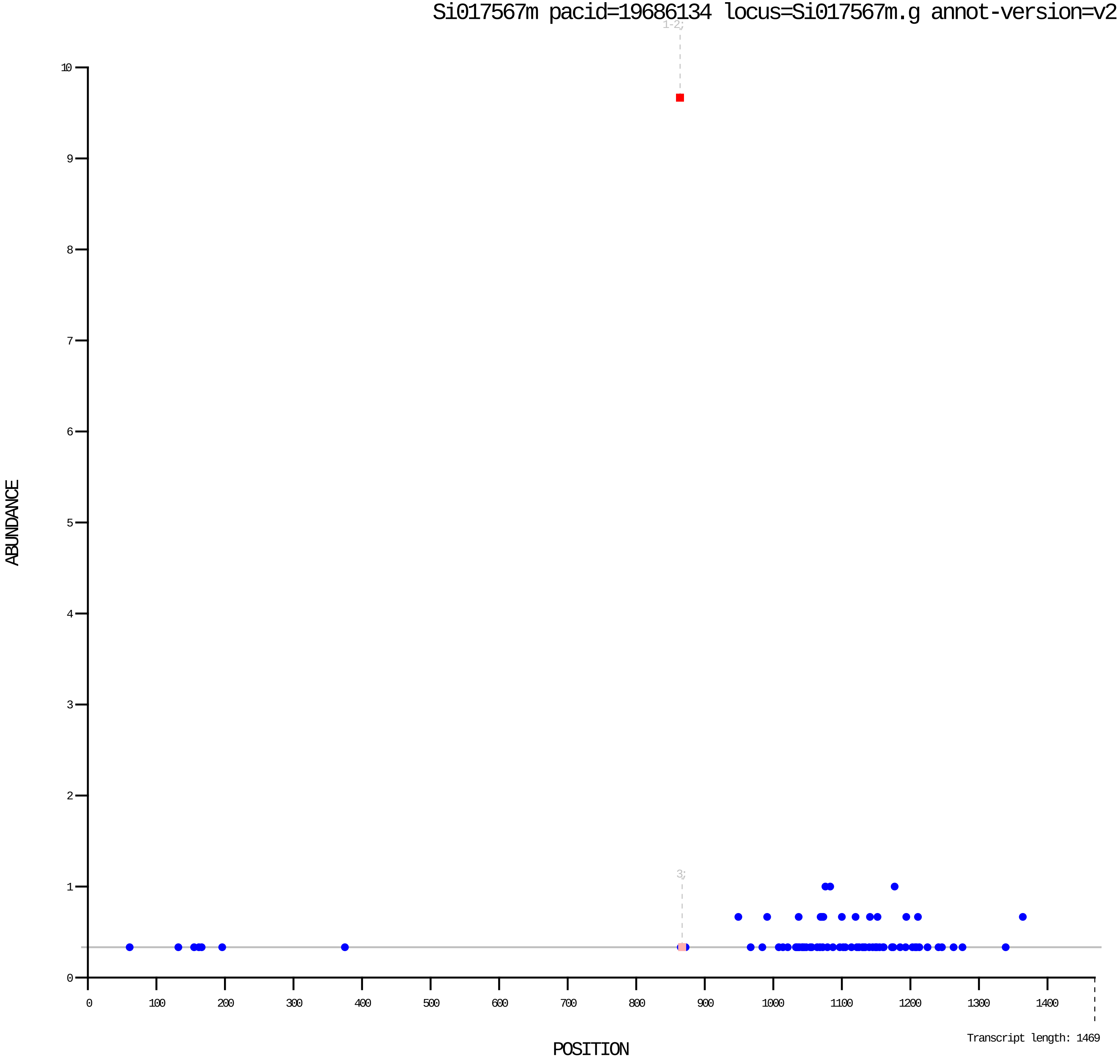

0 #1 Position:864 Abundance: 9.67(deg) 4(sRNA)
5' TGGAGAAGCAGGGCACGTGCA 3' ID:
|||||||||||| |||||| Score: 3.0
3' CGCAACCTCTTCGTCCAGTGCACAACACGGCC 5' p-value: 0.0

0 #2 Position:864 Abundance: 9.67(deg) 1(sRNA)
5' TGGAGAAGCAGGGCACGTGC 3' ID:
|||||||||||| |||||| Score: 2.0
3' CGCAACCTCTTCGTCCAGTGCACAACACGGCC 5' p-value: 0.0

4 #3 Position:867 Abundance: 0.33(deg) 1(sRNA)
5' TGCTGGAGAAGCAGGGCACGT 3' ID:
o| |||||||||||| ||||| Score: 2.5
3' TCGCGCAACCTCTTCGTCCAGTGCACAACACG 5' p-value: 0.02

Si026656m pacid=19707882 locus=Si026656m.g annot-version=v2.1

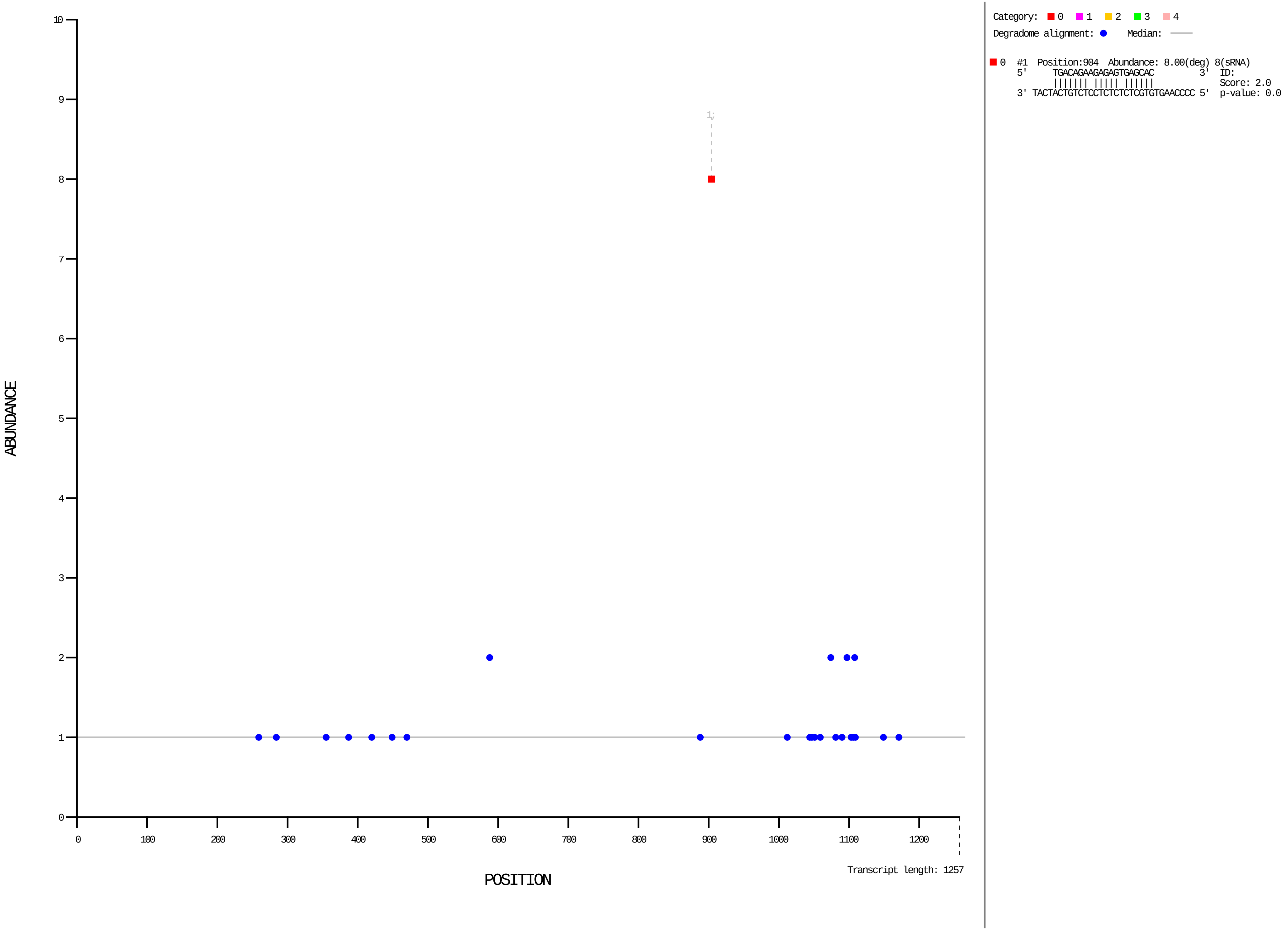

Si030892m pacid=19710830 locus=Si030892m.g annot-version=v2.1

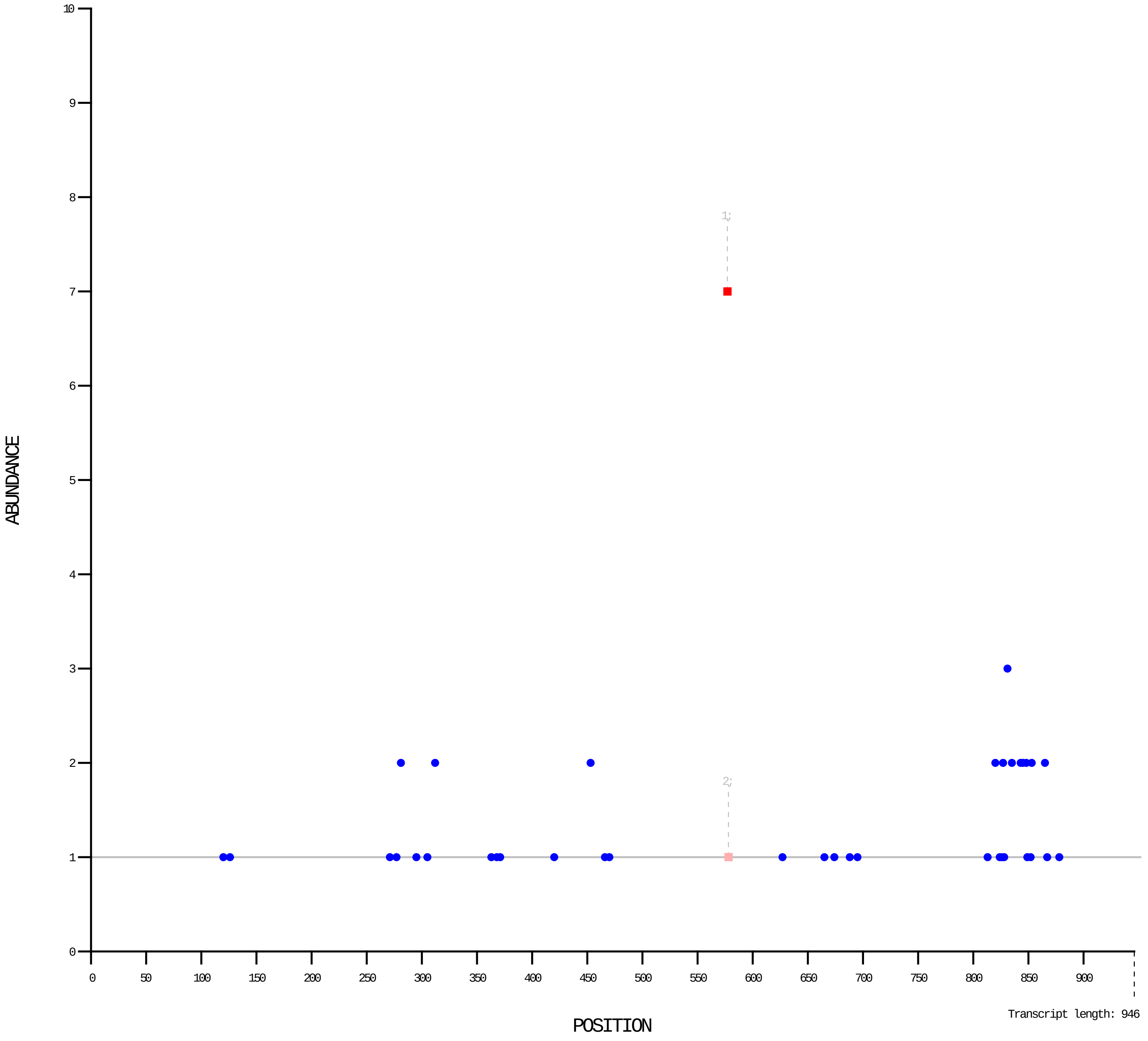

Category: 0 1 2 3 4
Degradome alignment: Median:

```
0  #1  Position:577  Abundance: 7.00(deg) 8(sRNA)
   5'     TGACAGAAGAGAGTGAGCAC        3'  ID:
          ||||||||||||| ||||||            Score: 1.0
   3' CCCTACTGTCTTCTCTCTCTCGTGTTAGGCTC 5'  p-value: 0.0

4  #2  Position:578  Abundance: 1.00(deg) 1(sRNA)
   5'     TTGACAGAAGAGAGCGAGCAC       3'  ID:
           ||||||||||||| ||||||           Score: 2.0
   3' CCCCTACTGTCTTCTCTCTCTCGTGTTAGGCT 5'  p-value: 0.01
```

Si023059m pacid=19701353 locus=Si023059m.g annot-version=v2.1

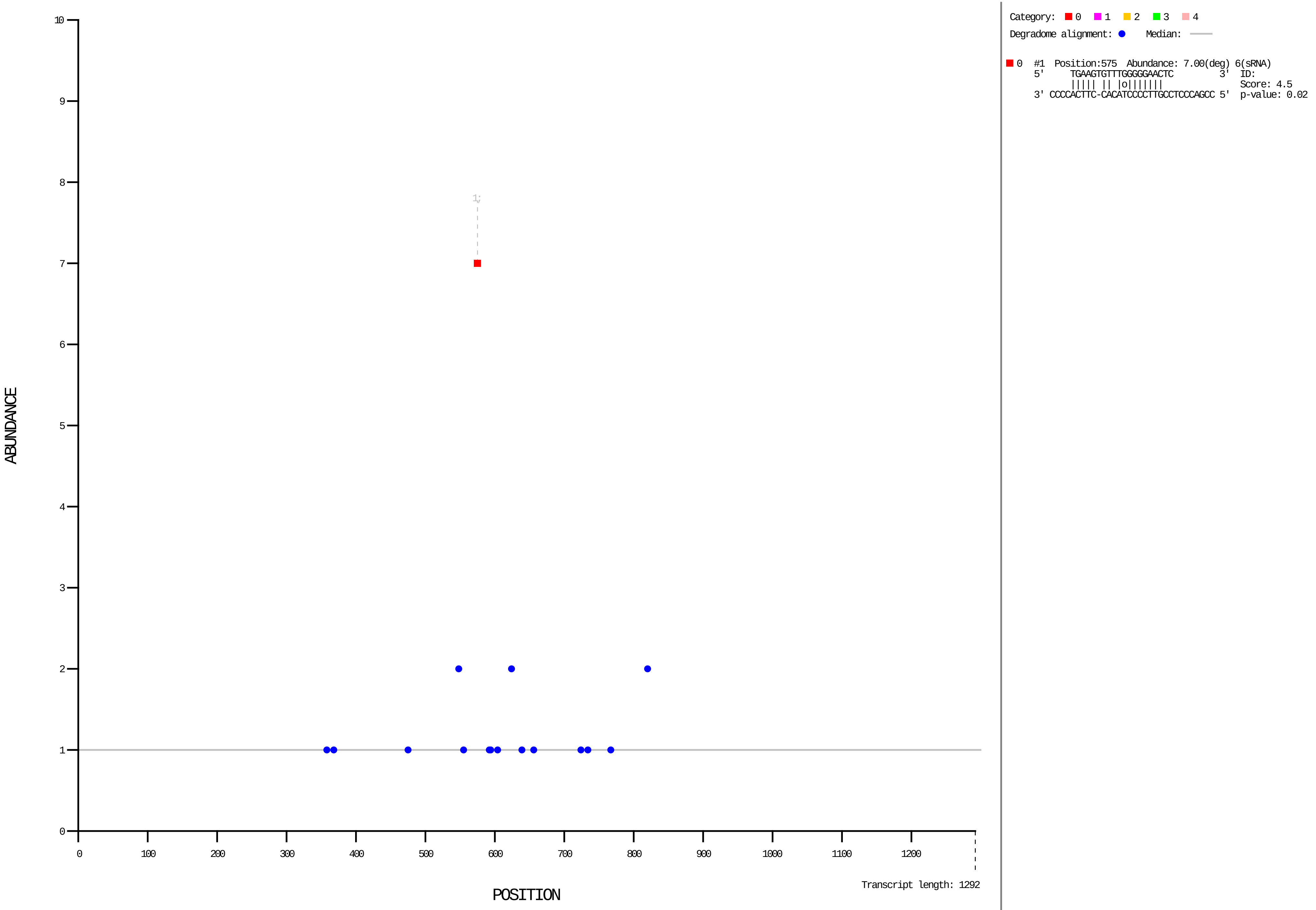

Si026680m pacid=19707179 locus=Si026680m.g annot-version=v2.1

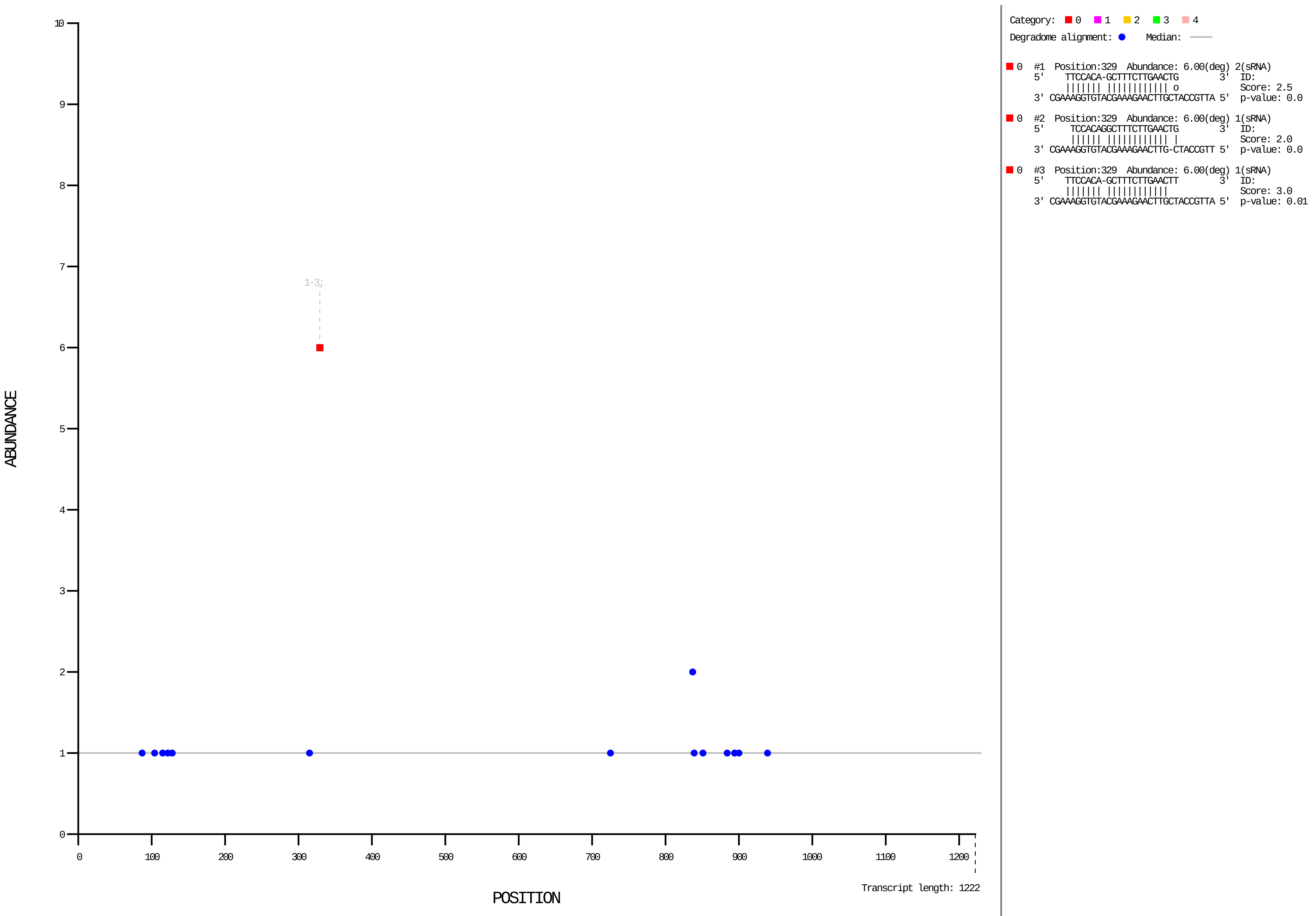

Si034587m pacid=19685698 locus=Si034587m.g annot-version=v2.1

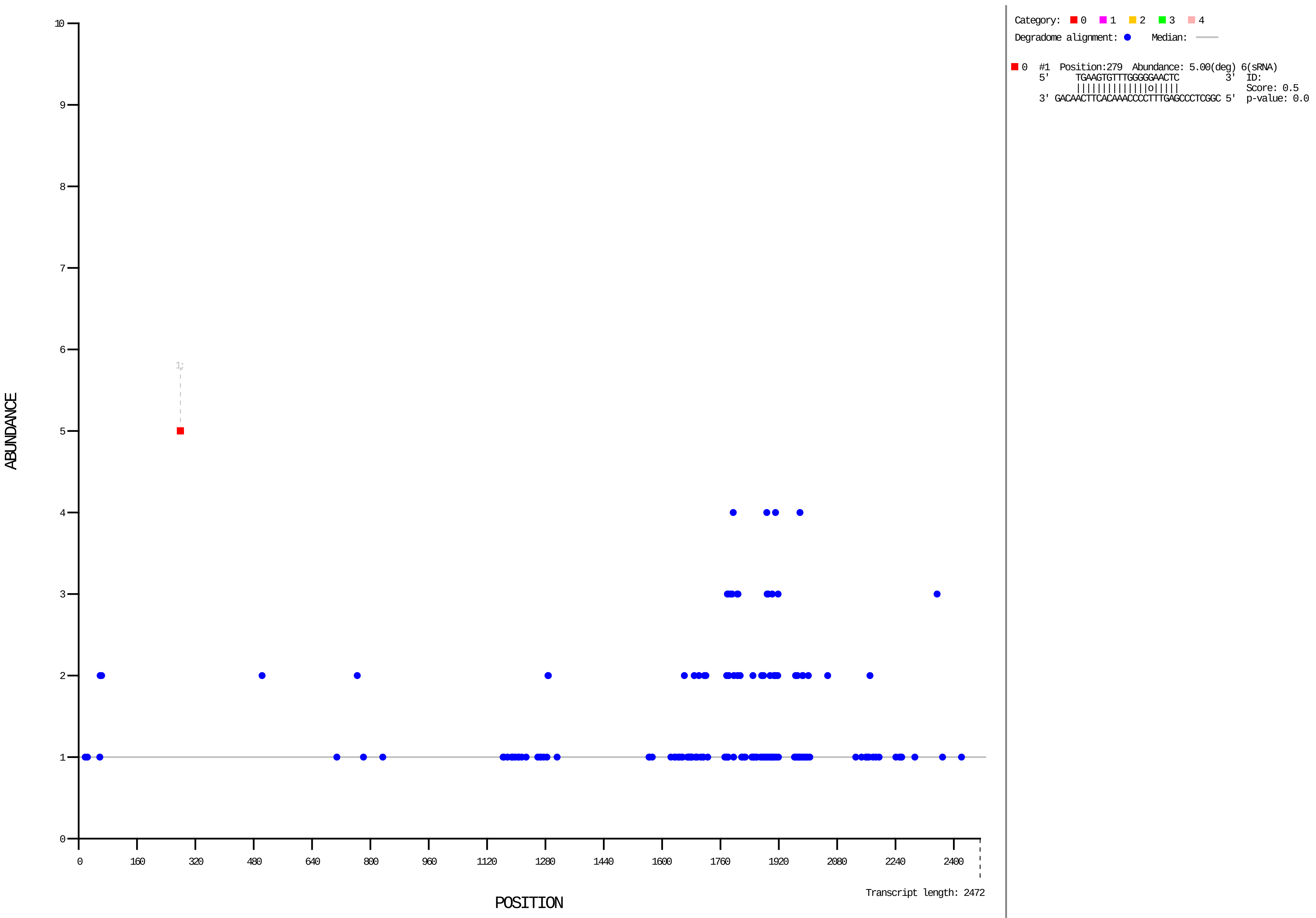

Si018249m pacid=19687960 locus=Si018249m.g annot-version=v2.1

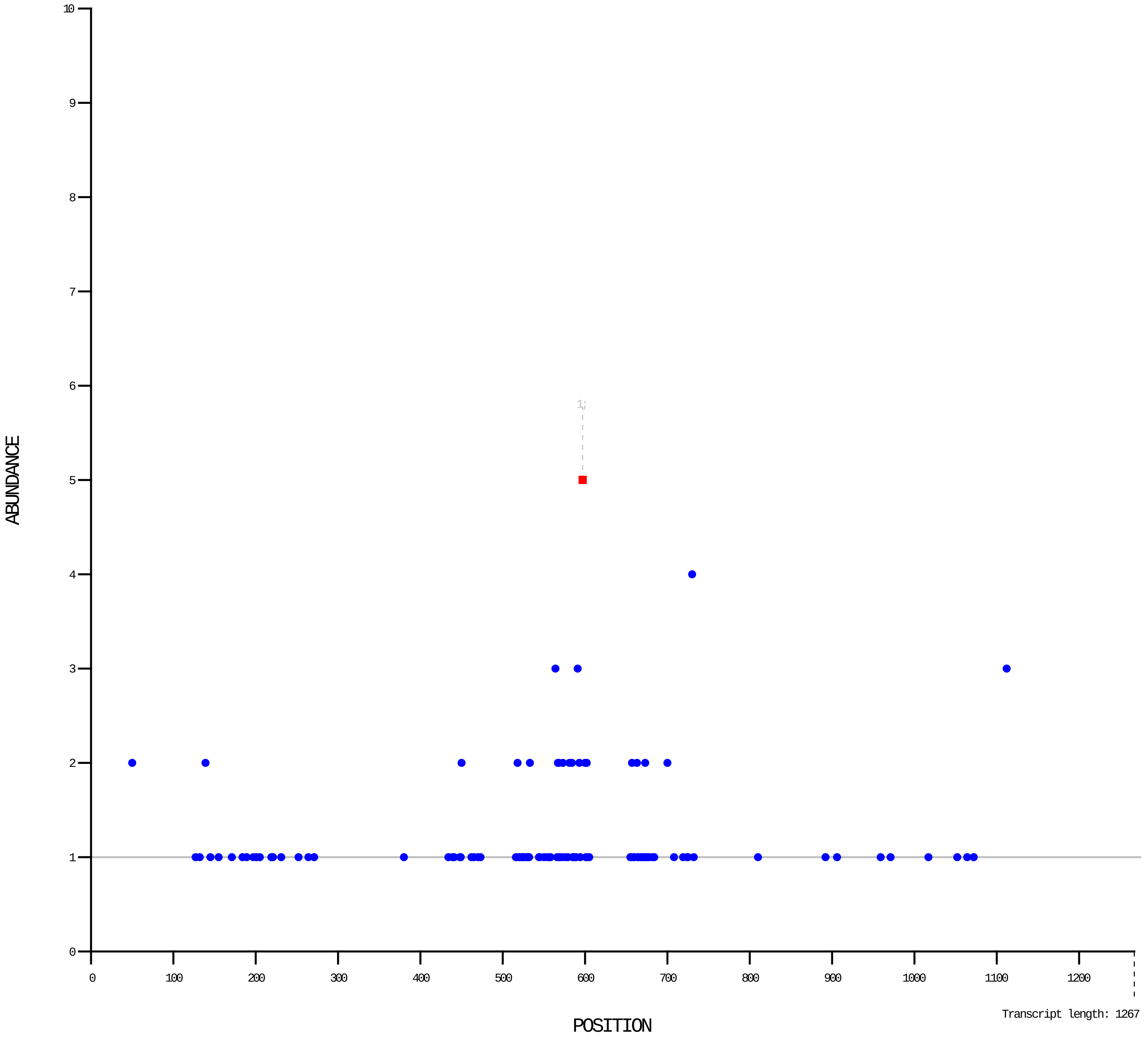

Category: 0 1 2 3 4

Degradome alignment: Median:

```
0  #1  Position:597  Abundance: 5.00(deg) 1(sRNA)
   5'     GCAGCACCATCAAGATTCACA        3'  ID:
          ||||| |||||||o|| || |            Score: 3.5
   3' AACCCGTCGAGGTAGTTTTA-GTTTTAGTGGT 5'  p-value: 0.0
```

Si013870m pacid=19691313 locus=Si013870m.g annot-version=v2.1

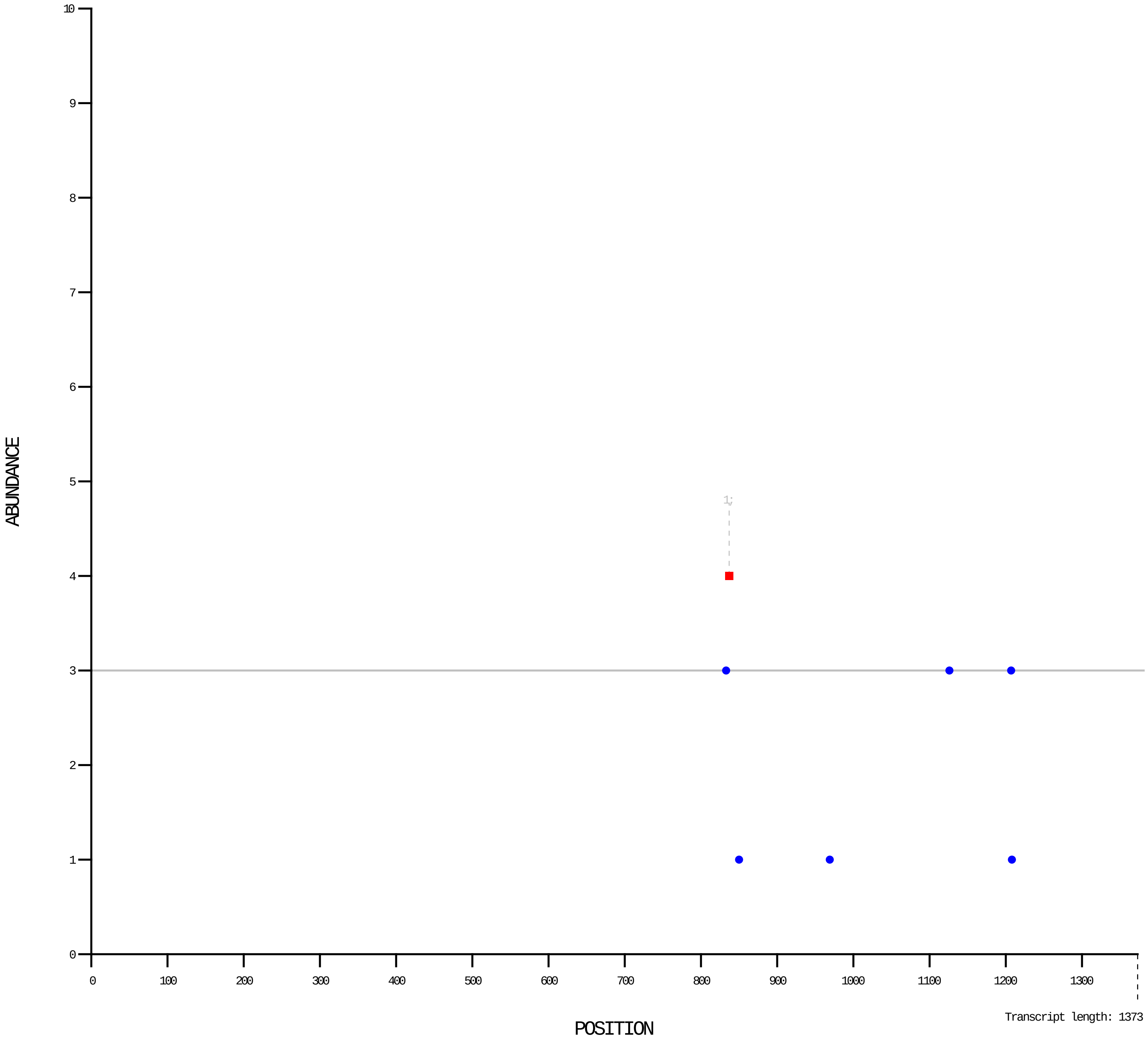

Category: 0 1 2 3 4
Degradome alignment: Median:

0 #1 Position:837 Abundance: 4.00(deg) 8(sRNA)
5' TGACAGAAGAGAGTGAGCAC 3' ID:
||||||||||||| |||||| Score: 1.0
3' CTCAACTGTCTTCTCTCTCTCGTGTCGATCTC 5' p-value: 0.0

Si021157m pacid=19697803 locus=Si021157m.g annot-version=v2.1

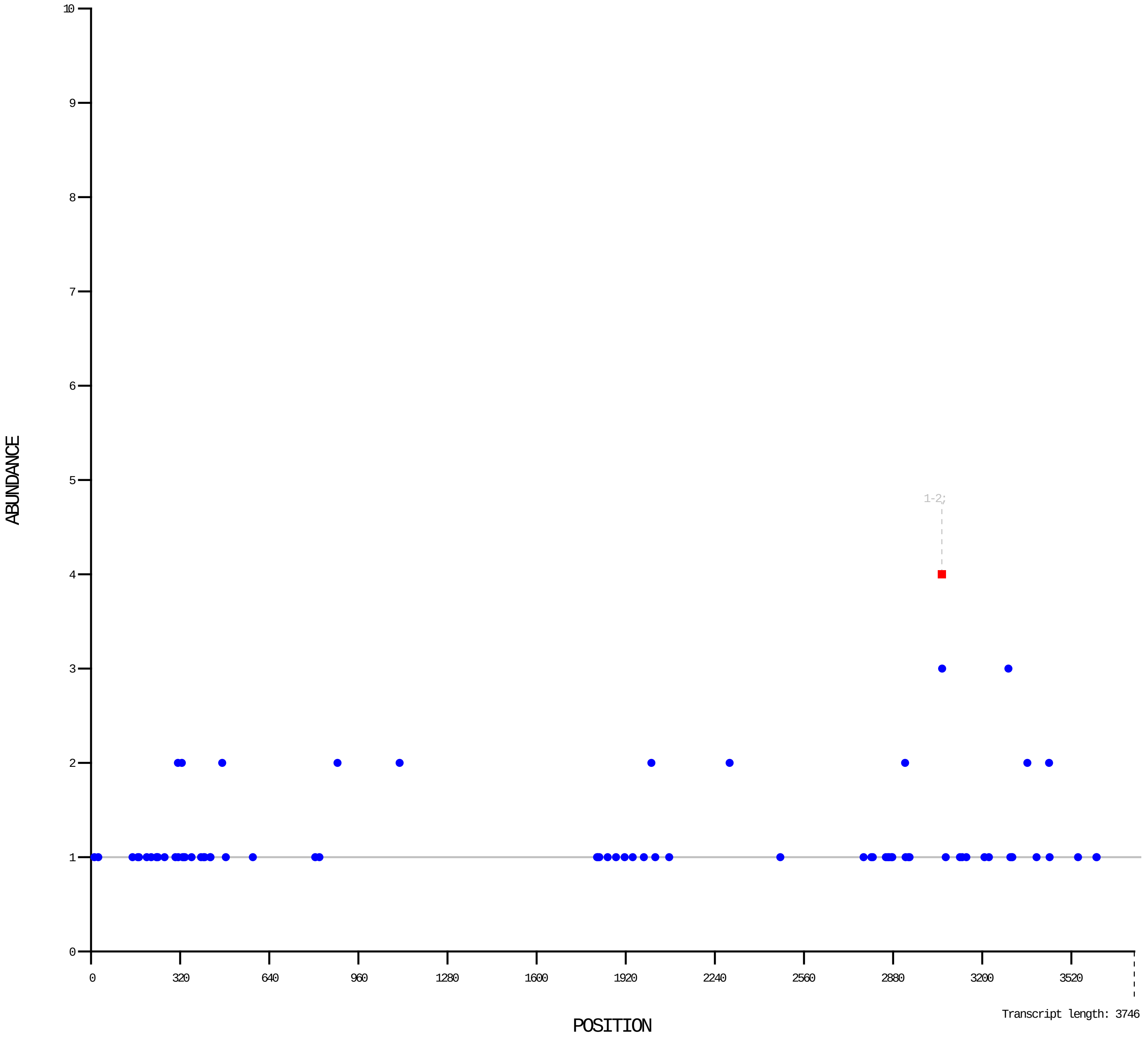

Category: 0 1 2 3 4
Degradome alignment: Median:

0 #1 Position:3055 Abundance: 4.00(deg) 6(sRNA)
5' TGAAGCTGCCAGCATGATCTGA 3' ID:
||||||||||| |||||| |
Score: 4.0
3' CTTATGTTCGACGGTCGGACTAGAATGTCCCA 5' p-value: 0.01

0 #2 Position:3055 Abundance: 4.00(deg) 2(sRNA)
5' TGAAGCTGCCAGCATGATCTA 3' ID:
||||||||||| ||||||
Score: 4.0
3' CTTATGTTCGACGGTCGGACTAGAATGTCCCA 5' p-value: 0.0

Si021179m pacid=19700076 locus=Si021179m.g annot-version=v2.1

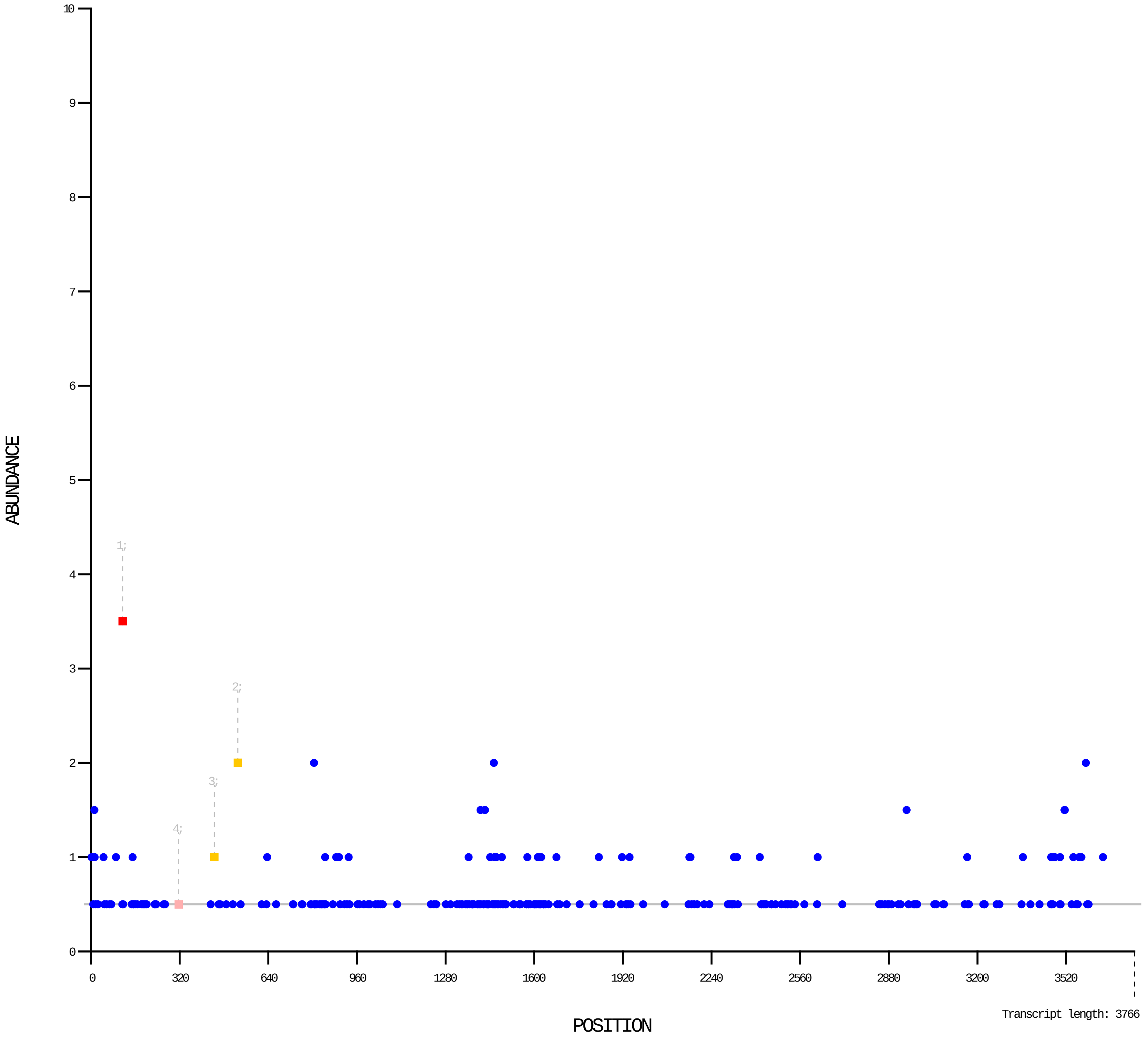

Category: 0 1 2 3 4

Degradome alignment: ● Median: —

```
0  #1  Position:114  Abundance: 3.50(deg) 1(sRNA)
   5'     TGCCAAAGGAGAGTTGCCCTG        3'  ID:
          |||||||||||| |||||||o            Score: 1.5
   3' ACAAACGGTTTCCTCTAAACGGGATTTTGTTC 5'  p-value: 0.0

2  #2  Position:530  Abundance: 2.00(deg) 1(sRNA)
   5'     TGCCAAAGGAGAGTTGCCCTG        3'  ID:
          ||||||||||||o||o|||||            Score: 1.0
   3' AATTACGGTTTCCTCTTAATGGGACTATATCG 5'  p-value: 0.0

2  #3  Position:445  Abundance: 1.00(deg) 1(sRNA)
   5'     TGCCAAAGGAGAGTTGCCCTG        3'  ID:
          |||||||||||| |||||| o            Score: 2.5
   3' AAAAACGGTTTCCTCTAAACGGGTTTCTCGTC 5'  p-value: 0.02

4  #4  Position:316  Abundance: 0.50(deg) 1(sRNA)
   5'     TGCCAAAGGAGAGTTGCCCTG        3'  ID:
          |||||||||||| |||||| |            Score: 2.0
   3' CATTACGGTTTCCTCTAAACGGGTCGTTTAGT 5'  p-value: 0.0
```

Si021181m pacid=19700077 locus=Si021179m.g annot-version=v2.1

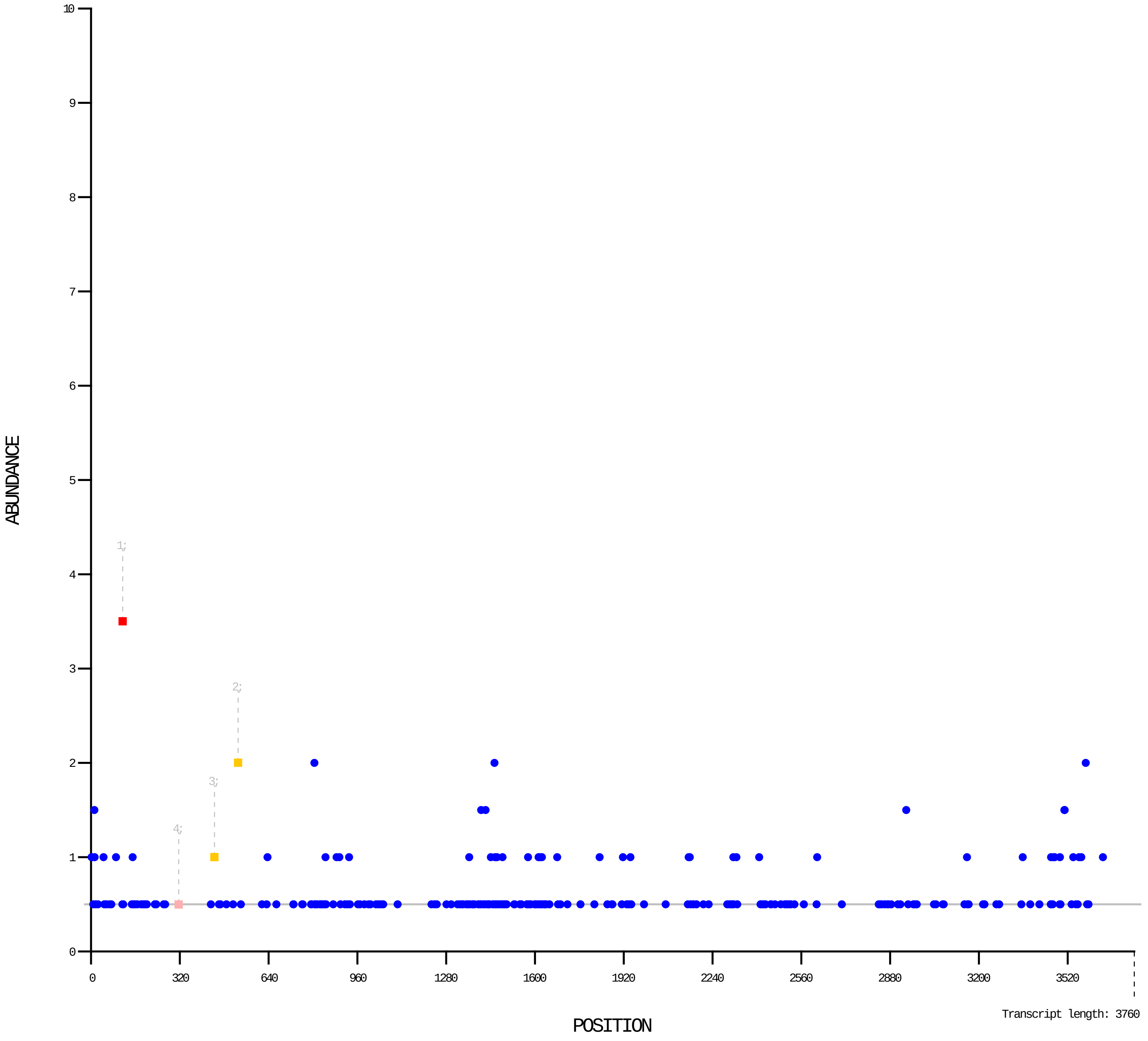


```
Category: 0 1 2 3 4
Degradome alignment:     Median:

0  #1  Position:114  Abundance: 3.50(deg) 1(sRNA)
   5'     TGCCAAAGGAGAGTTGCCCTG        3'  ID:
          |||||||||||| |||||||o            Score: 1.5
   3' ACAAACGGTTTCCTCTAAACGGGATTTTGTTC 5'  p-value: 0.0

2  #2  Position:530  Abundance: 2.00(deg) 1(sRNA)
   5'     TGCCAAAGGAGAGTTGCCCTG        3'  ID:
          ||||||||||||o||o|||||            Score: 1.0
   3' AATTACGGTTTCCTCTTAATGGGACTATATCG 5'  p-value: 0.0

2  #3  Position:445  Abundance: 1.00(deg) 1(sRNA)
   5'     TGCCAAAGGAGAGTTGCCCTG        3'  ID:
          |||||||||||| |||||| o            Score: 2.5
   3' AAAAACGGTTTCCTCTAAACGGGTTTCTCGTC 5'  p-value: 0.0

4  #4  Position:316  Abundance: 0.50(deg) 1(sRNA)
   5'     TGCCAAAGGAGAGTTGCCCTG        3'  ID:
          |||||||||||| |||||| |            Score: 2.0
   3' CATTACGGTTTCCTCTAAACGGGTCGTTTAGT 5'  p-value: 0.0
```

Si013747m pacid=19692046 locus=Si013747m.g annot-version=v2.1

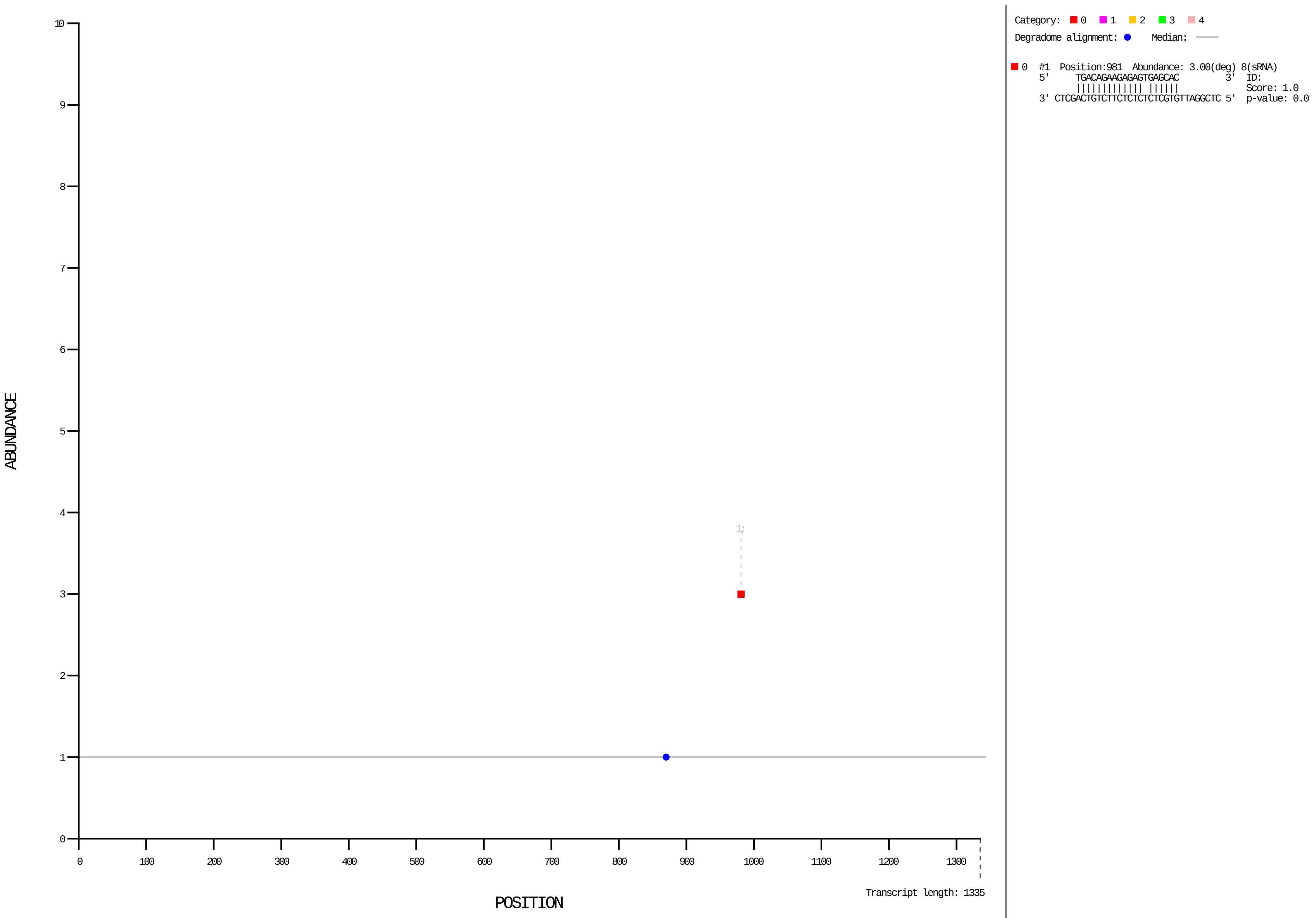

Si017883m pacid=19686225 locus=Si017883m.g annot-version=v2.1

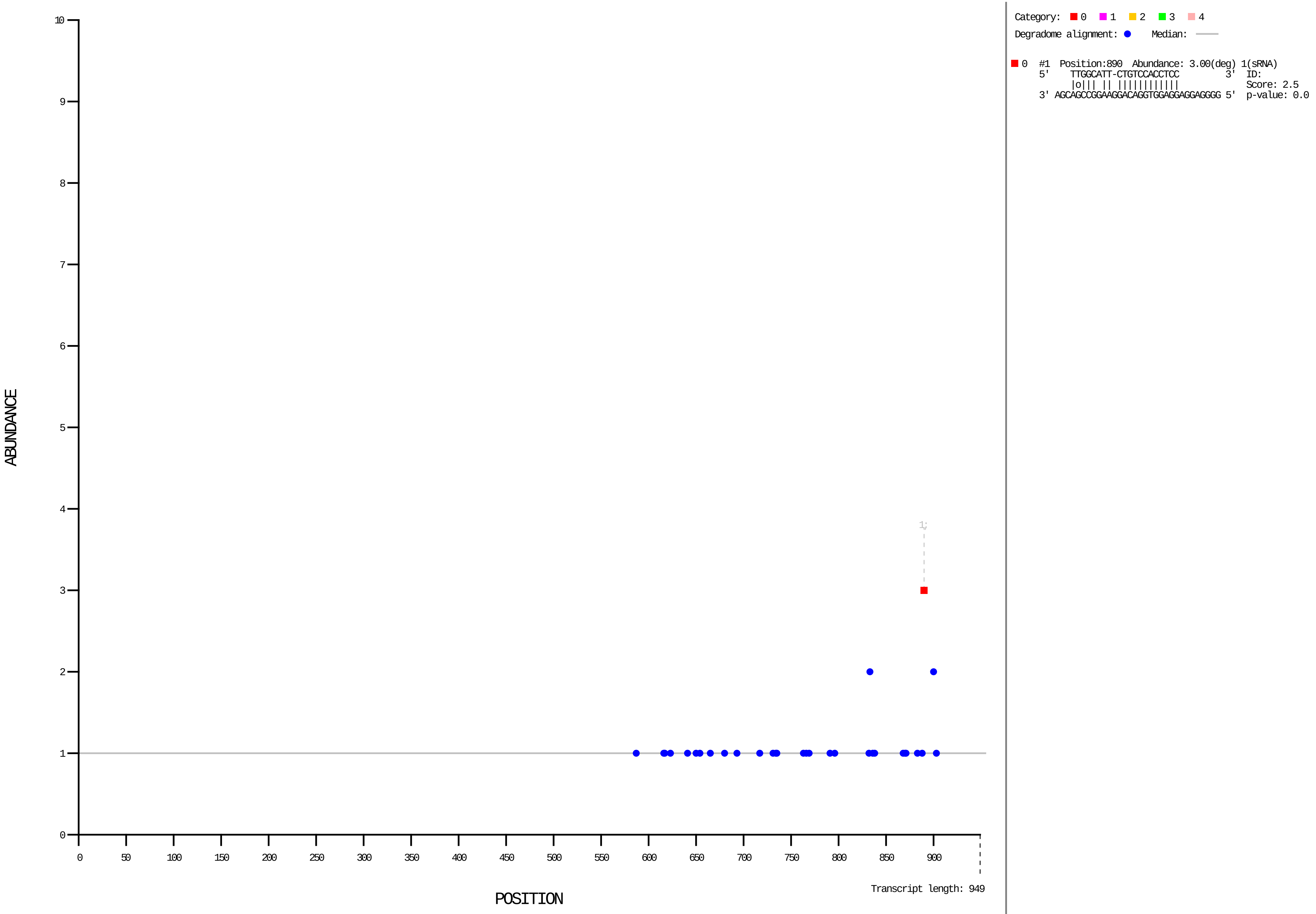

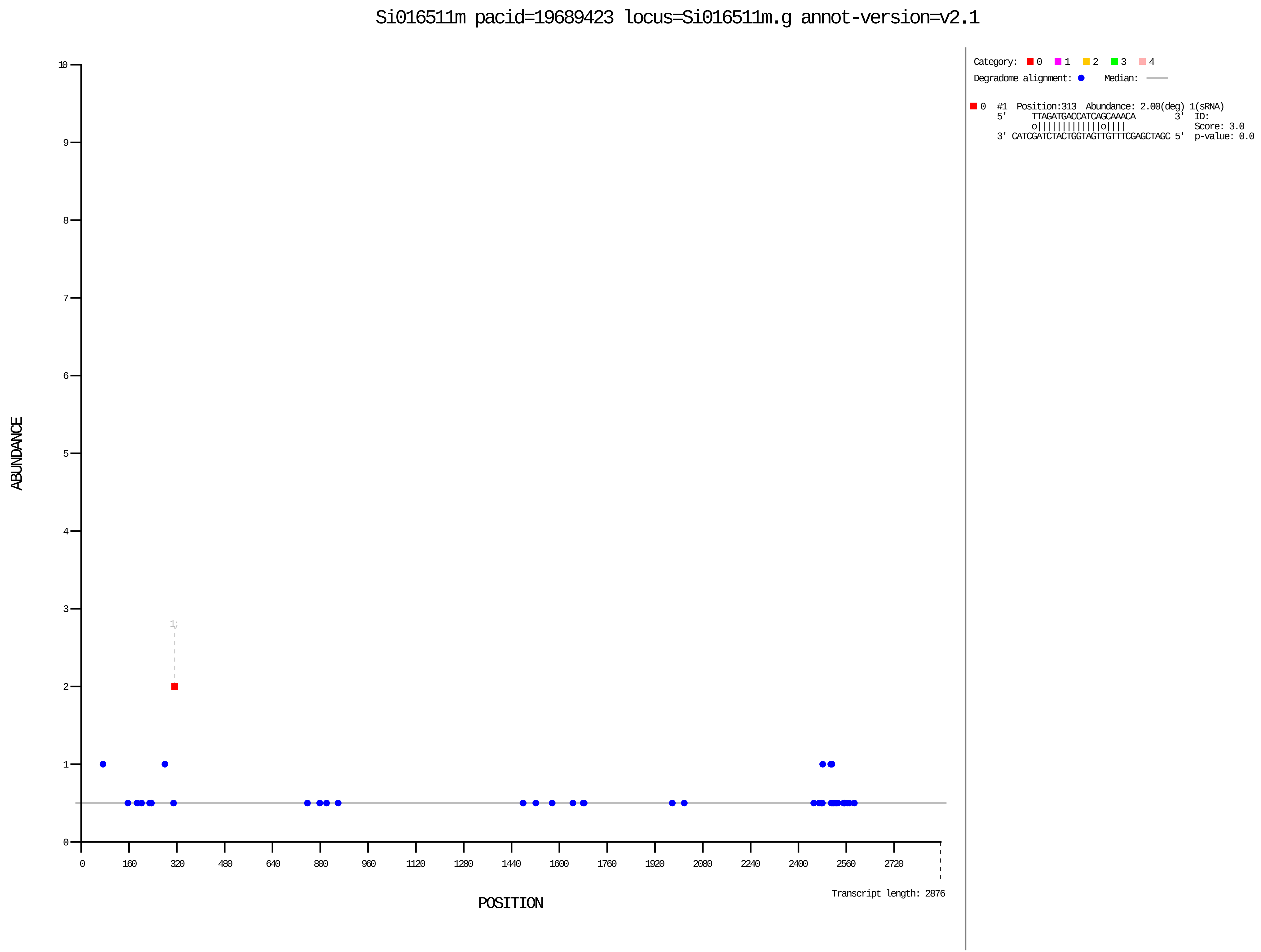
Si016511m pacid=19689423 locus=Si016511m.g annot-version=v2.1
Category: 0 1 2 3 4
Degradome alignment:
Median:
0 #1 Position:313 Abundance: 2.00(deg) 1(sRNA)
5' TTAGATGACCATCAGCAAACA 3' ID:
o|||||||||||||o||||
Score: 3.0
3' CATCGATCTACTGGTAGTTGTTTCGAGCTAGC 5' p-value: 0.0
ABUNDANCE
POSITION
Transcript length: 2876

Si016510m pacid=19689424 locus=Si016511m.g annot-version=v2.1

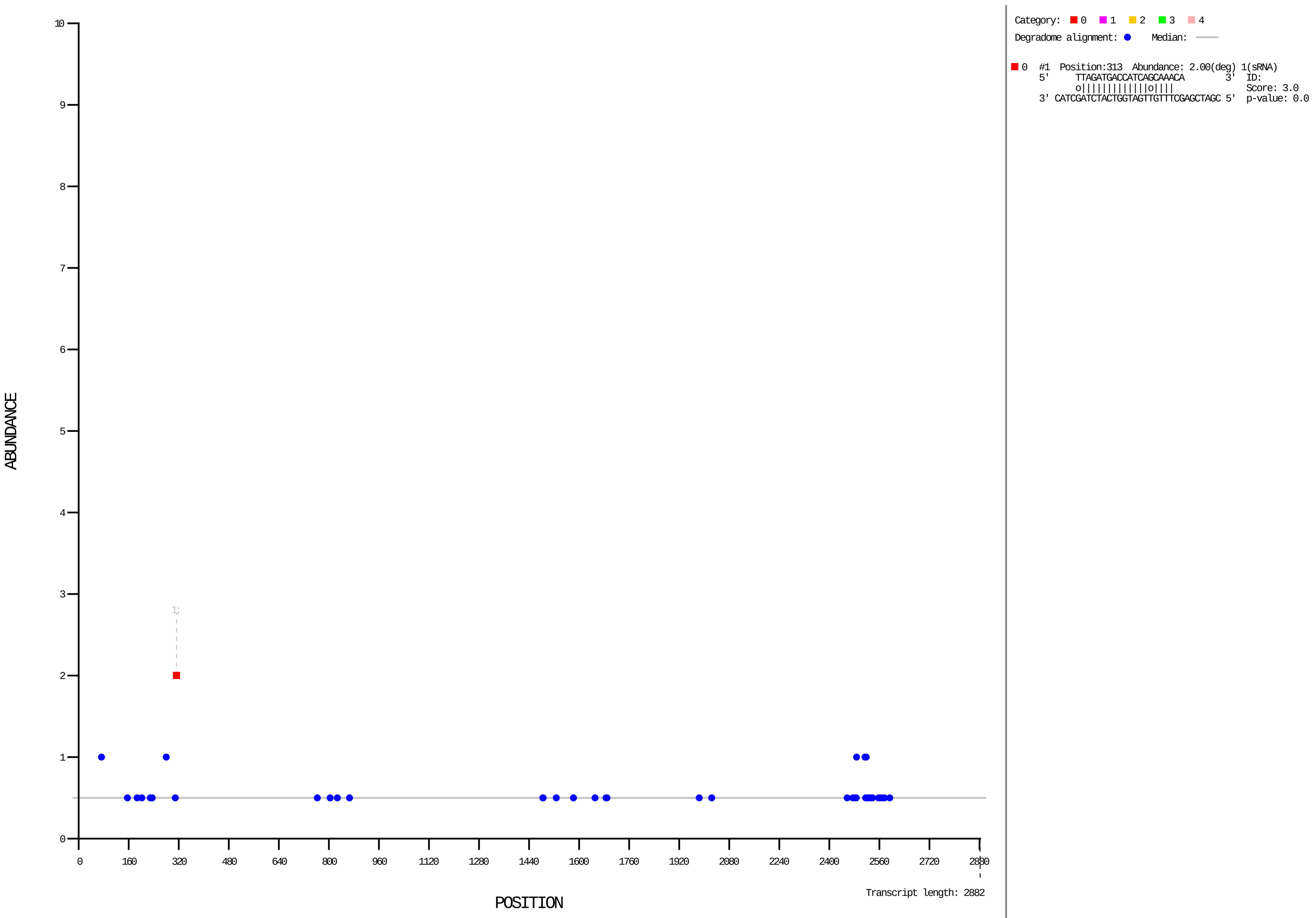

Si006472m pacid=19705891 locus=Si006472m.g annot-version=v2.1

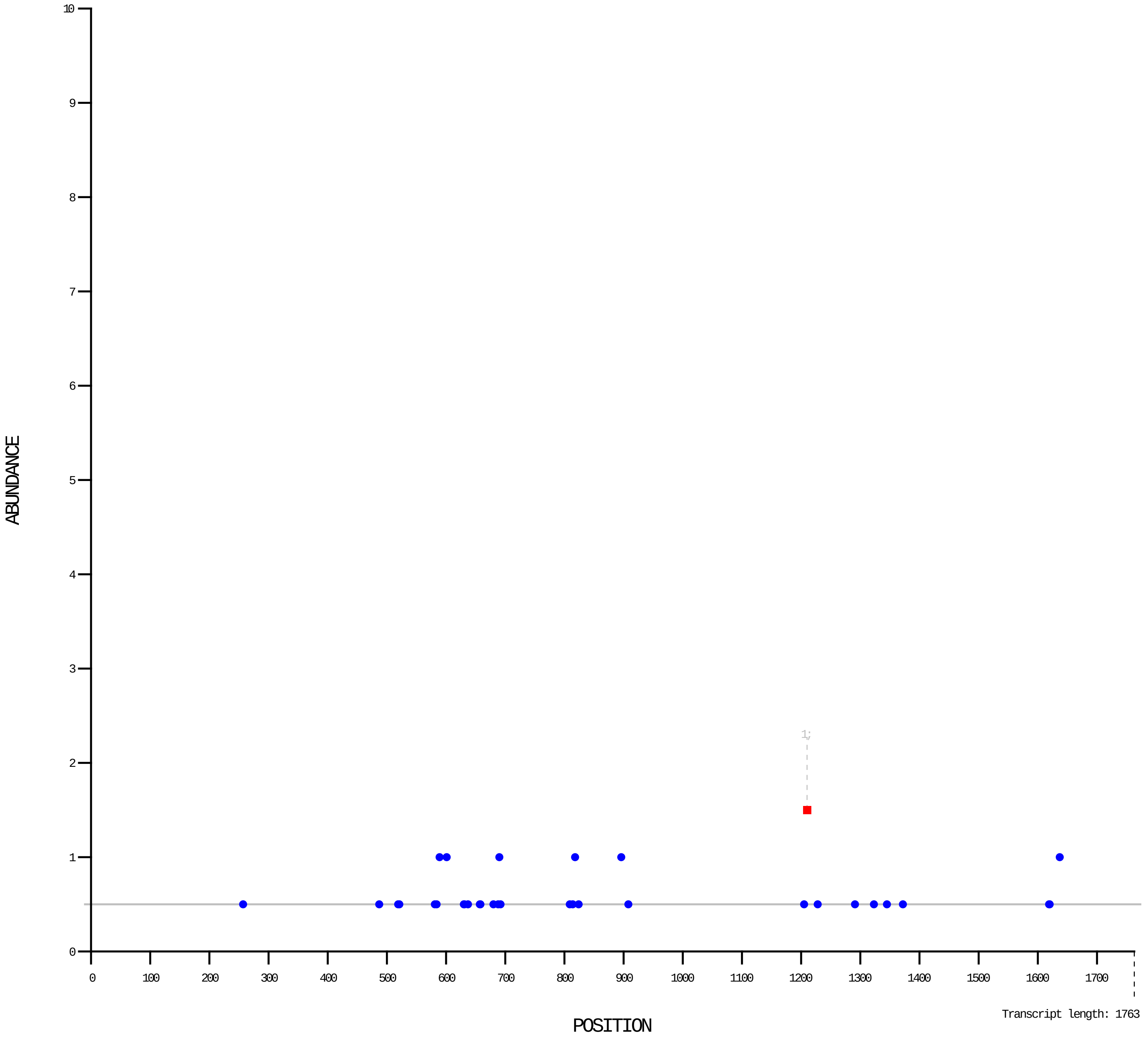

Category: 0 1 2 3 4
Degradome alignment: Median:

0 #1 Position:1210 Abundance: 1.50(deg) 8(sRNA)
5' TGACAGAAGAGAGTGAGCAC 3' ID:
|||||||||||| ||||||| Score: 1.0
3' GCAGACTGTCTTCTCTCTCTCGTGCGGGTTCA 5' p-value: 0.0

Si006471m pacid=19705892 locus=Si006472m.g annot-version=v2.1

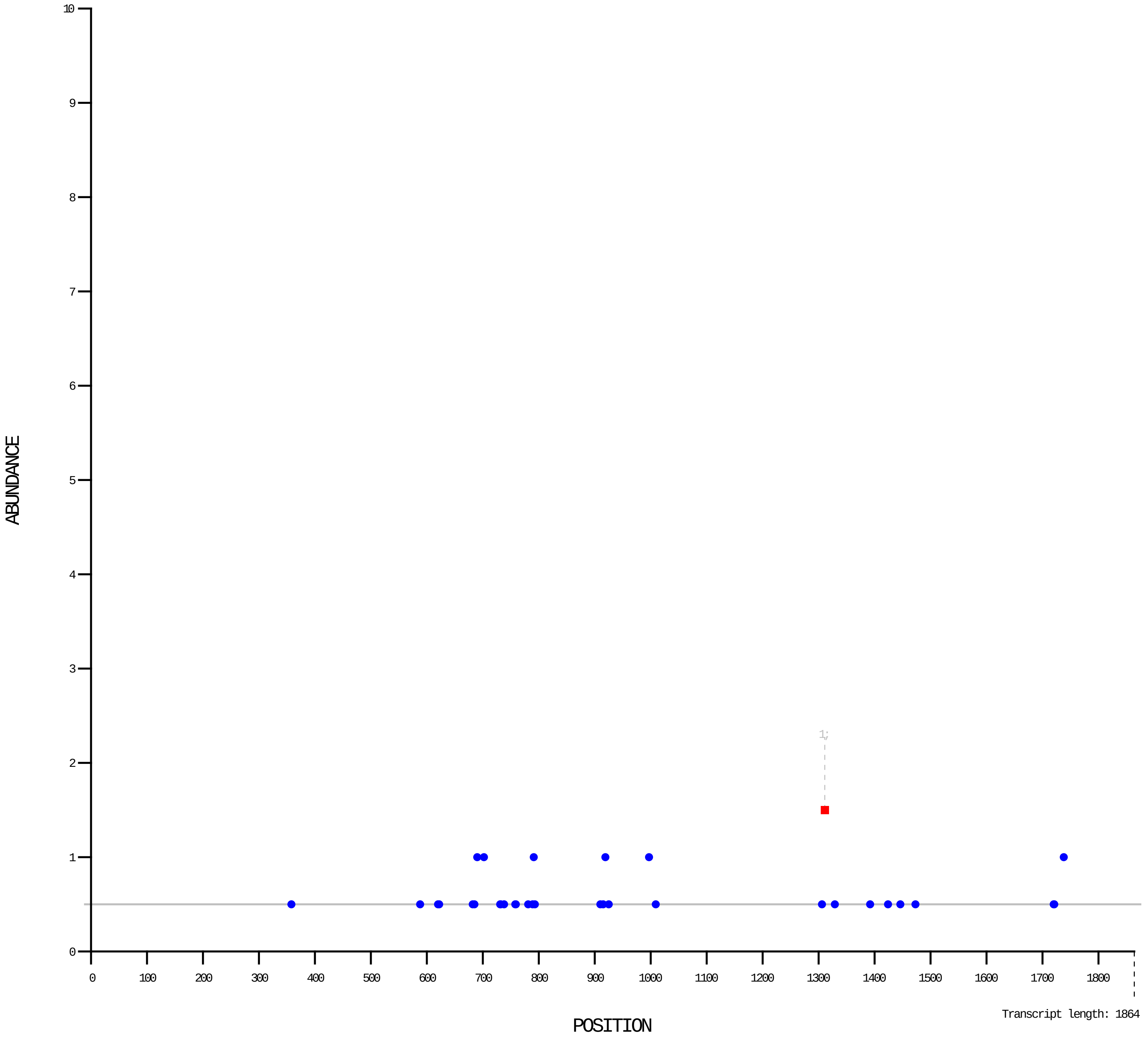

Category: 0 1 2 3 4
Degradome alignment: Median:

0 #1 Position:1311 Abundance: 1.50(deg) 8(sRNA)
```
5'     TGACAGAAGAGAGTGAGCAC          3'  ID:
       |||||||||||| ||||||               Score: 1.0
3' GCAGACTGTCTTCTCTCTCTCGTGCGGGTTCA 5'  p-value: 0.0
```

Si002023m pacid=19675870 locus=Si002023m.g annot-version=v2.1

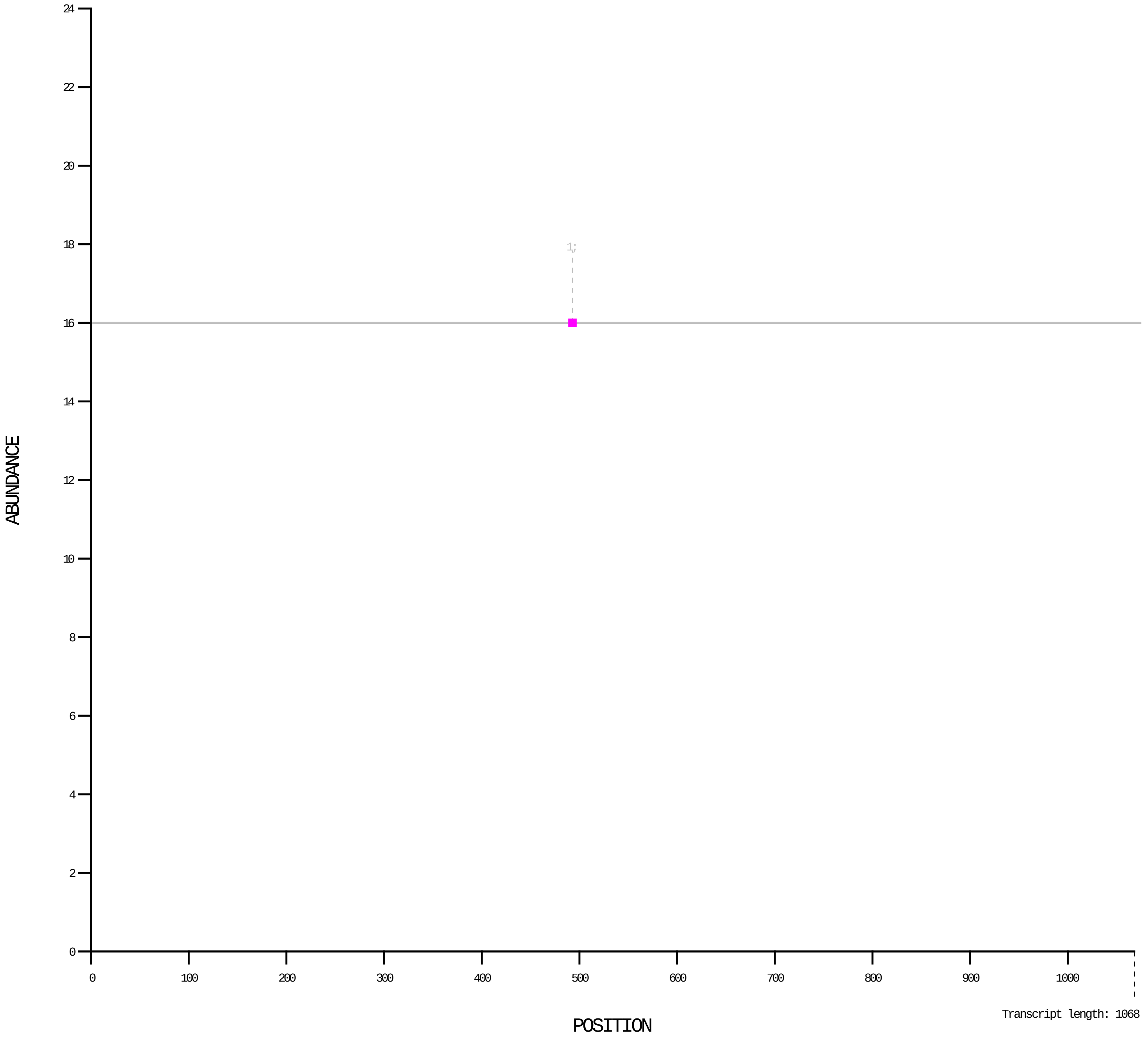

Category: 0 1 2 3 4

Degradome alignment: Median:

```
1  #1  Position:493  Abundance: 16.00(deg) 2(sRNA)
   5'     TTTGGATTGAAGGGAGCTCTG        3'  ID:
          ||||| ||||||||| ||||o|           Score: 2.5
   3' ATGTAAACCAAACTTCCCCCGAGGCAACCCAC 5'  p-value: 0.0
```

Si021201m pacid=19701825 locus=Si021201m.g annot-version=v2.1

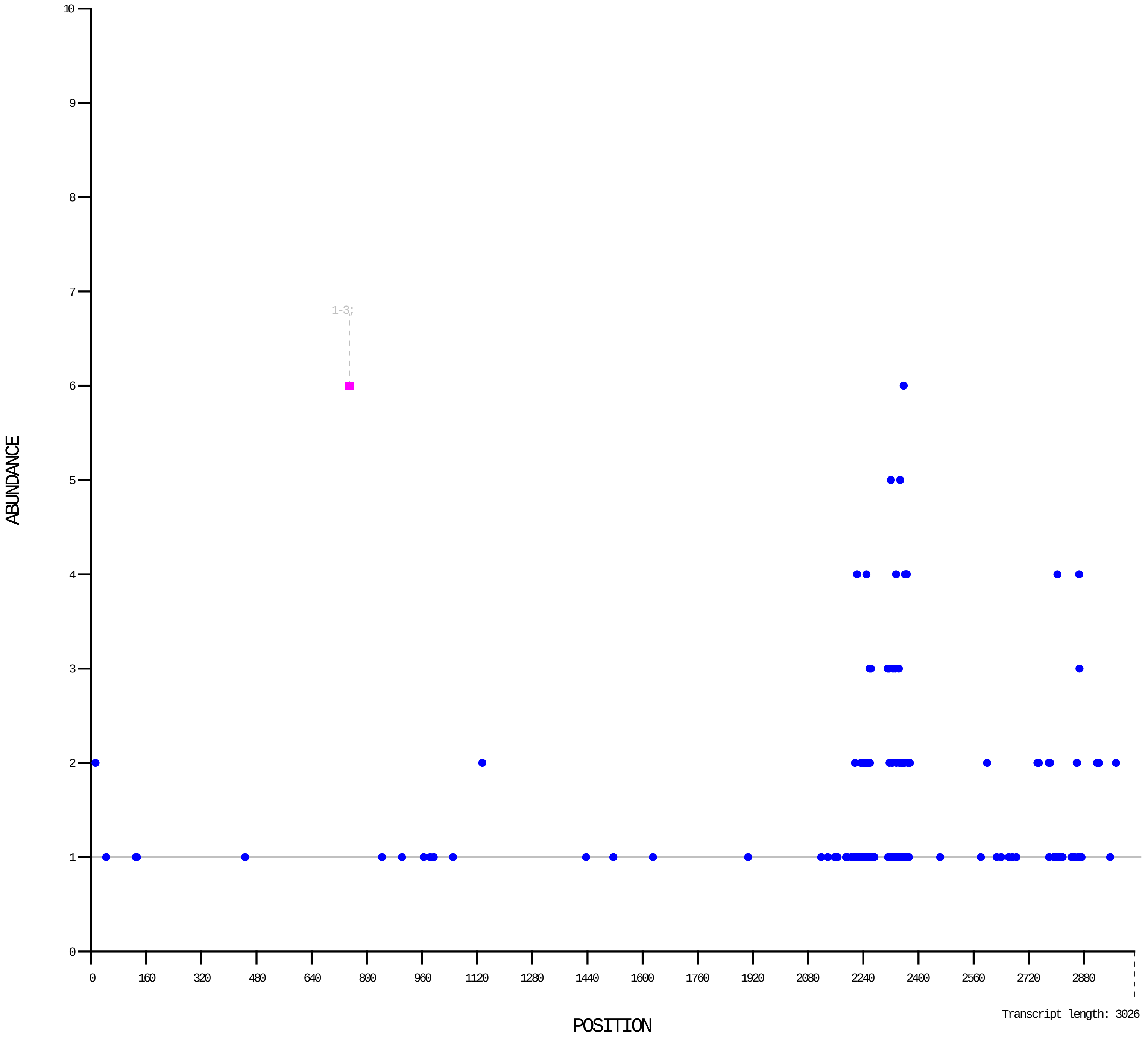


```
Category: 0 1 2 3 4
Degradome alignment:     Median:

1   #1  Position:750  Abundance: 6.00(deg) 7(sRNA)
    5'     TCGGACCAGGCTTCATTCCCC        3'  ID:
           o|||||||||||||||| |||            Score: 2.5
    3' CTTAGGCCTGGTCCGAAGTA-GGGTTGGTAAA 5'  p-value: 0.01

1   #2  Position:750  Abundance: 6.00(deg) 2(sRNA)
    5'     TCGGACCAGGCTTCAATCCCT        3'  ID:
           o||||||||||||||| ||||            Score: 2.5
    3' CTTAGGCCTGGTCCGAAGT-AGGGTTGGTAAA 5'  p-value: 0.0

1   #3  Position:750  Abundance: 6.00(deg) 2(sRNA)
    5'     TCGGACCAGGCTTCATTCCTC        3'  ID:
           o|||||||||||||||o||              Score: 3.0
    3' CTTAGGCCTGGTCCGAAGTAGGGTTGGTAAAC 5'  p-value: 0.01
```

Si039629m pacid=19681059 locus=Si039629m.g annot-version=v2.1

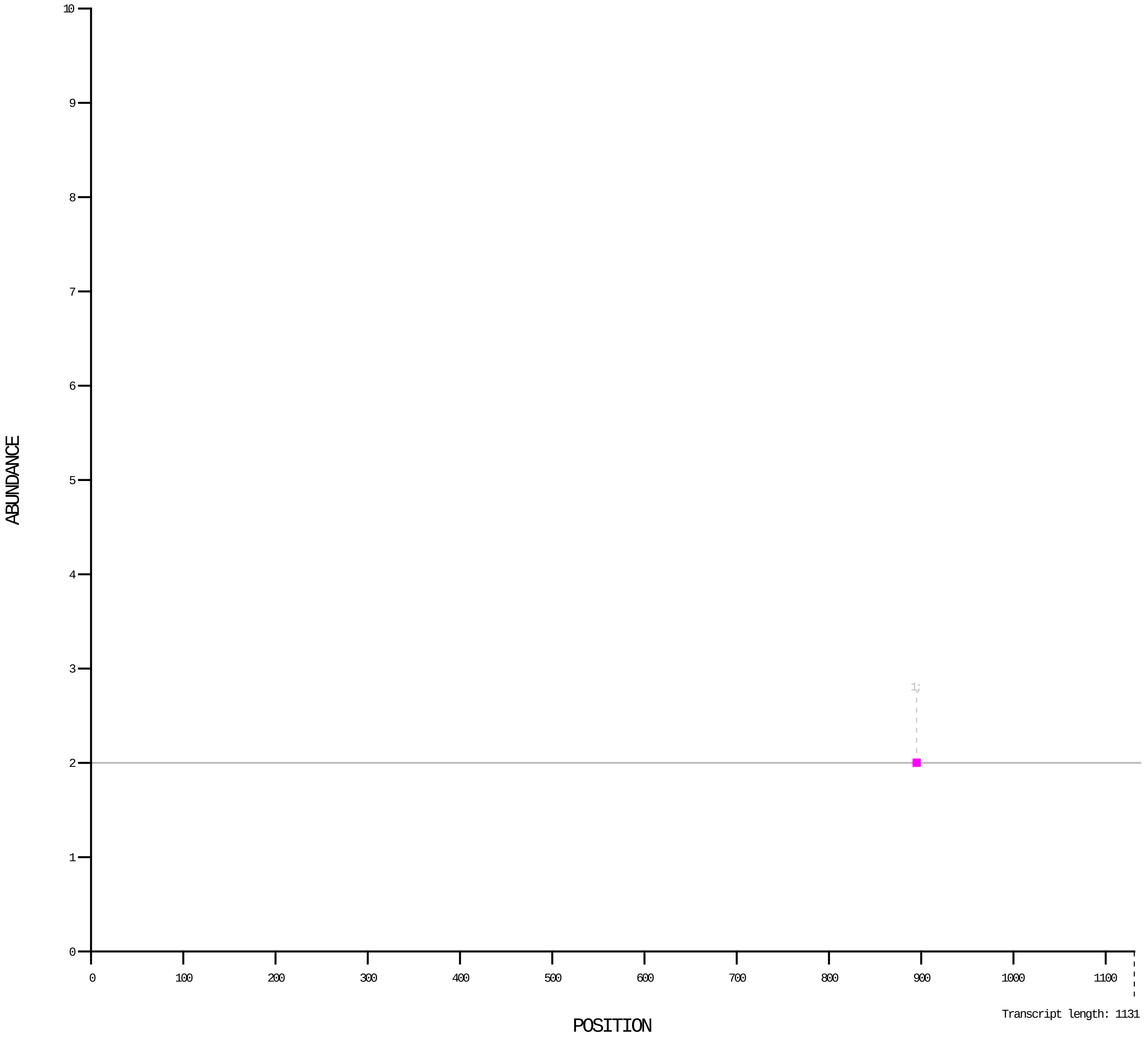

Category: 0 1 2 3 4
Degradome alignment: Median:

1 #1 Position:895 Abundance: 2.00(deg) 2(sRNA)

```
5'     TTTGGATTGAAGGGAGCTCTG        3'  ID:
          |||| ||||||||||||||oo         Score: 3.0
3' GGCTTAACCAAACTTCCCTCGAGGTGAGACCC 5'  p-value: 0.0
```

Si010741m pacid=19694328 locus=Si010741m.g annot-version=v2.1

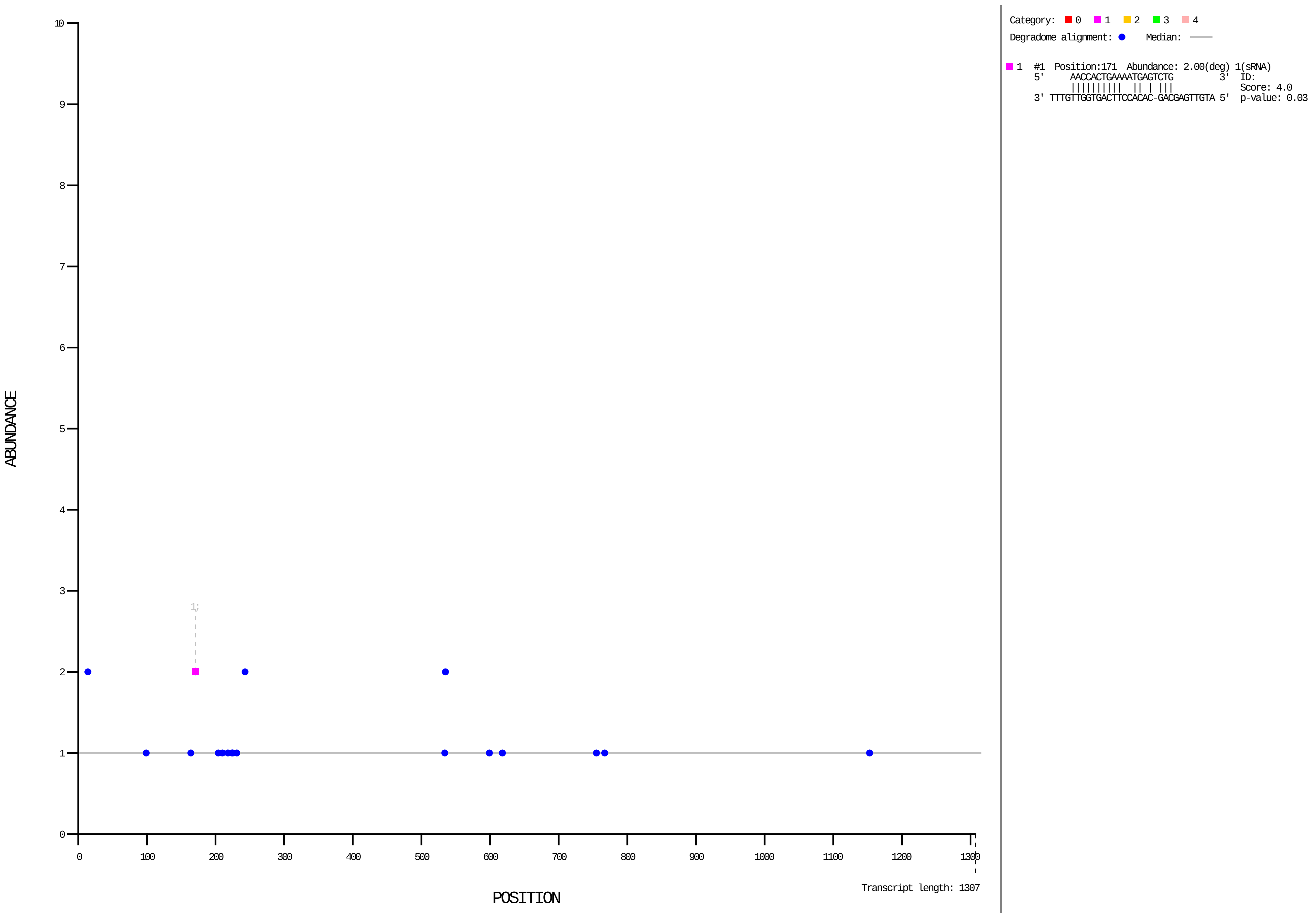

Si034686m pacid=19679389 locus=Si034686m.g annot-version=v2.1

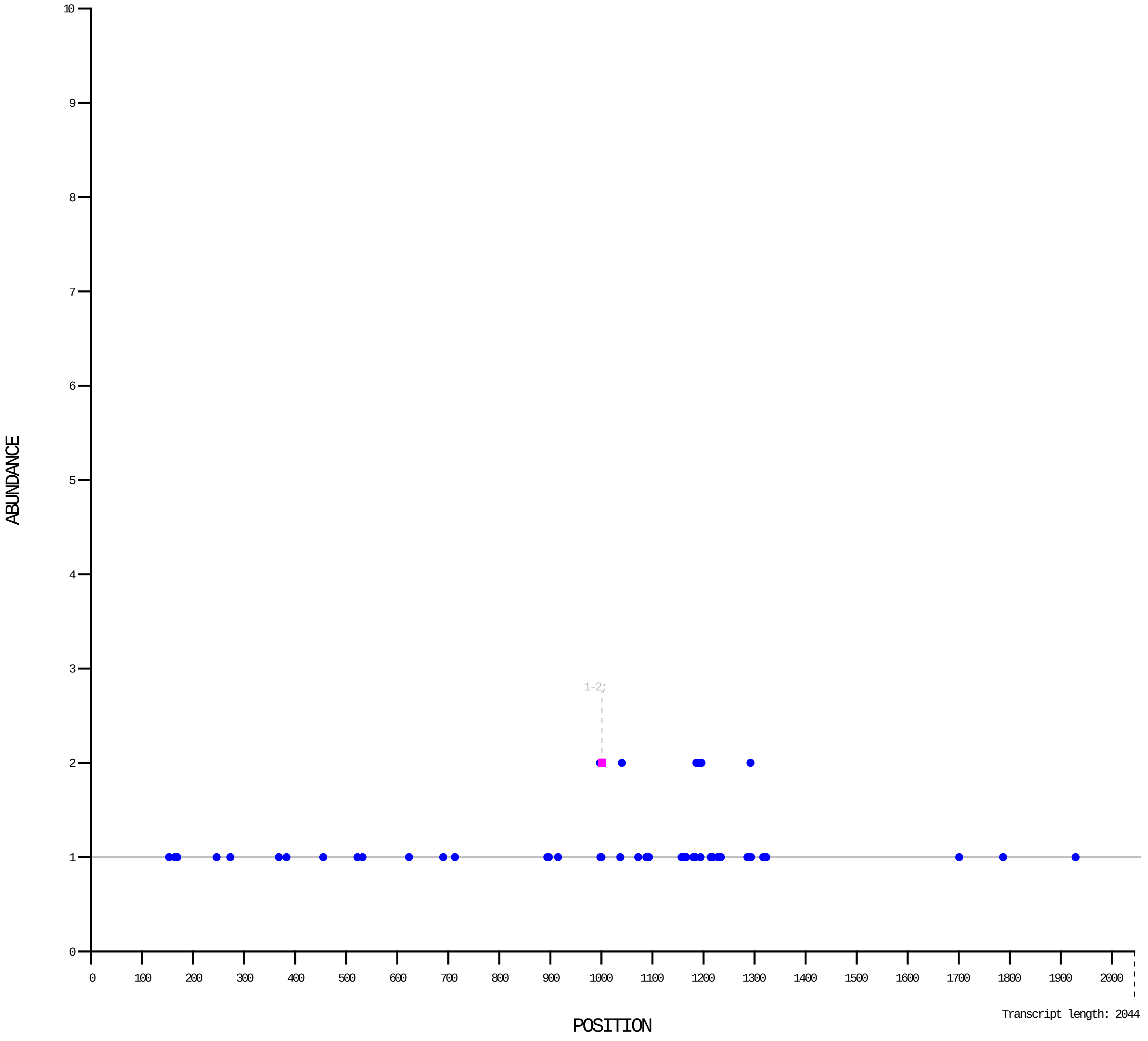

Category: 0 1 2 3 4
Degradome alignment: Median:

```
1   #1  Position:1001  Abundance: 2.00(deg) 1(sRNA)
    5'     TTGAGCCGCGTCAATATCTCC        3'  ID:
           ||||||||||||| o |||||            Score: 2.5
    3' CCGGAACTCGGCGCAGTCGGAGAGGTGGAGGA 5'  p-value: 0.0

1   #2  Position:1001  Abundance: 2.00(deg) 1(sRNA)
    5'     TTGAGCCGCGCCAATATCTCT        3'  ID:
           |||||||||| || o ||||o            Score: 4.0
    3' CCGGAACTCGGCGCAGTCGGAGAGGTGGAGGA 5'  p-value: 0.0
```

Si014342m pacid=19692803 locus=Si014342m.g annot-version=v2.1

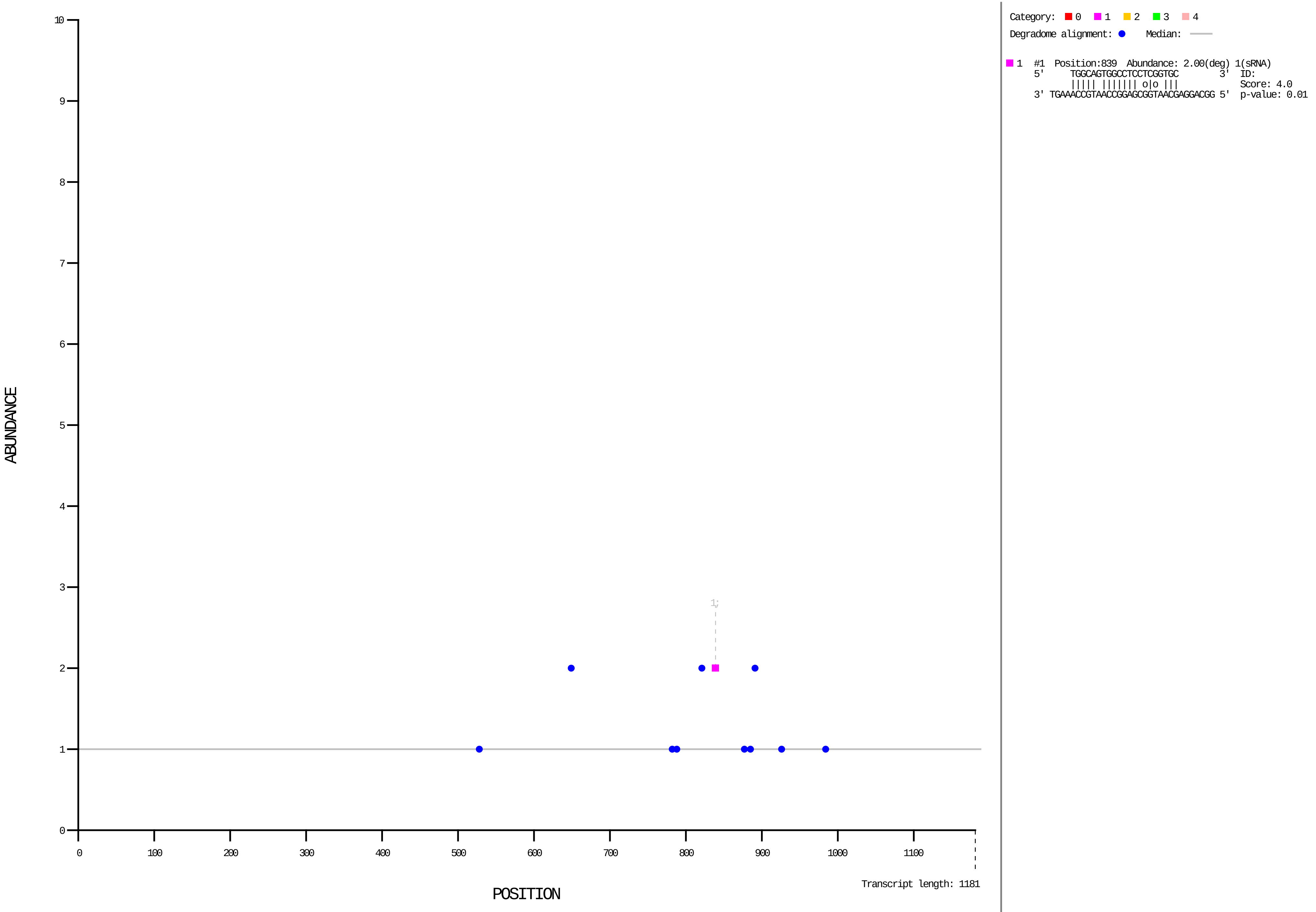

Si011264m pacid=19697080 locus=Si011264m.g annot-version=v2.1

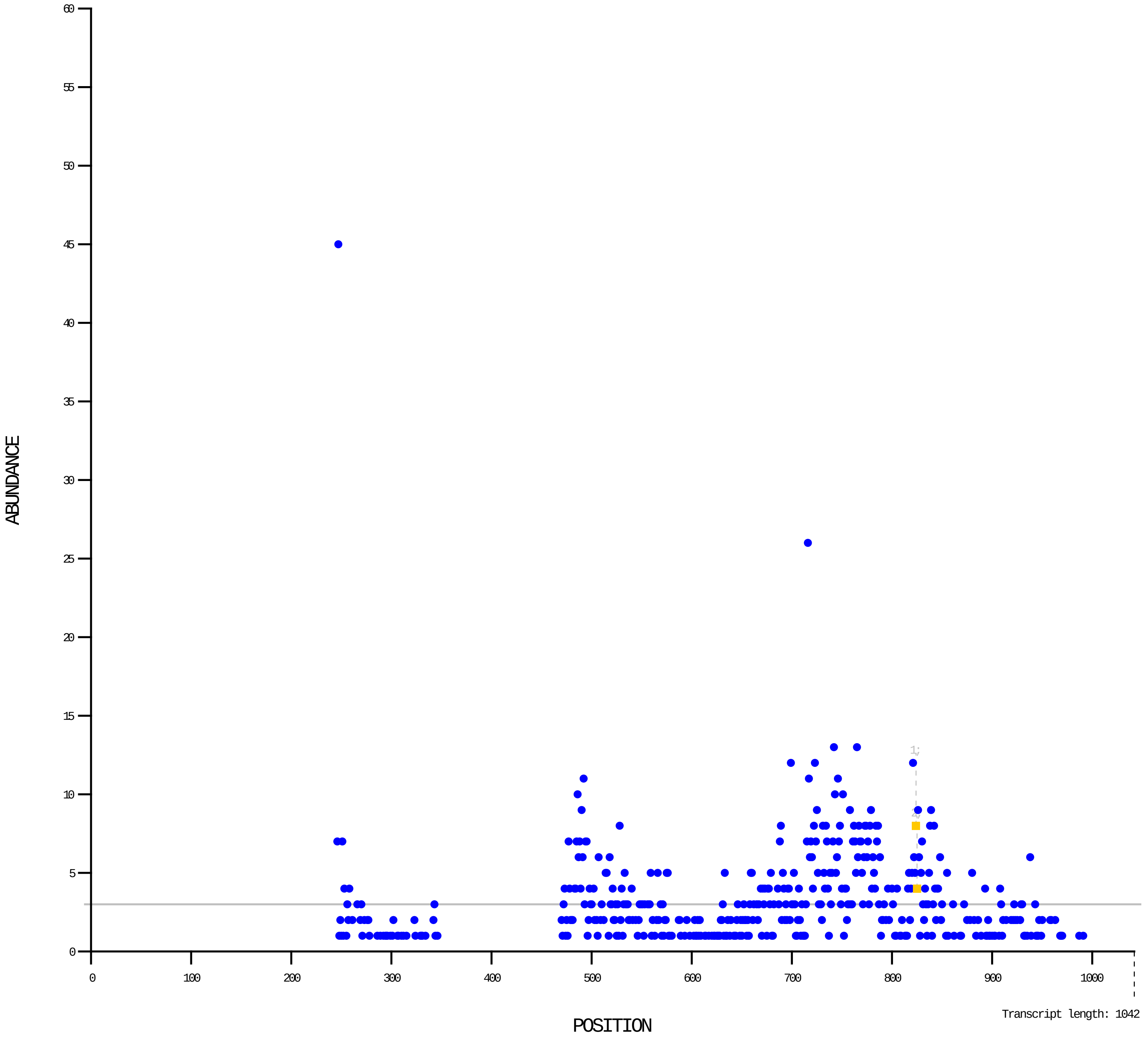

Category: 0 1 2 3 4
Degradome alignment: Median:

2 #1 Position:824 Abundance: 8.00(deg) 1(sRNA)
5' TATAAAGCC-TAATTAATCCAT 3' ID:
|||| |o|| |||||||o|||| Score: 3.0
3' TCAATATATTGGAATTAATTGGGTACGTACCT 5' p-value: 0.01

2 #2 Position:825 Abundance: 4.00(deg) 1(sRNA)
5' TATAAAGCCT-AATTAATCCAT 3' ID:
|||| |o||| ||||||o|||| Score: 3.0
3' ATCAATATATTGGAATTAATTGGGTACGTACC 5' p-value: 0.0

Si031154m pacid=19710244 locus=Si031154m.g annot-version=v2.1

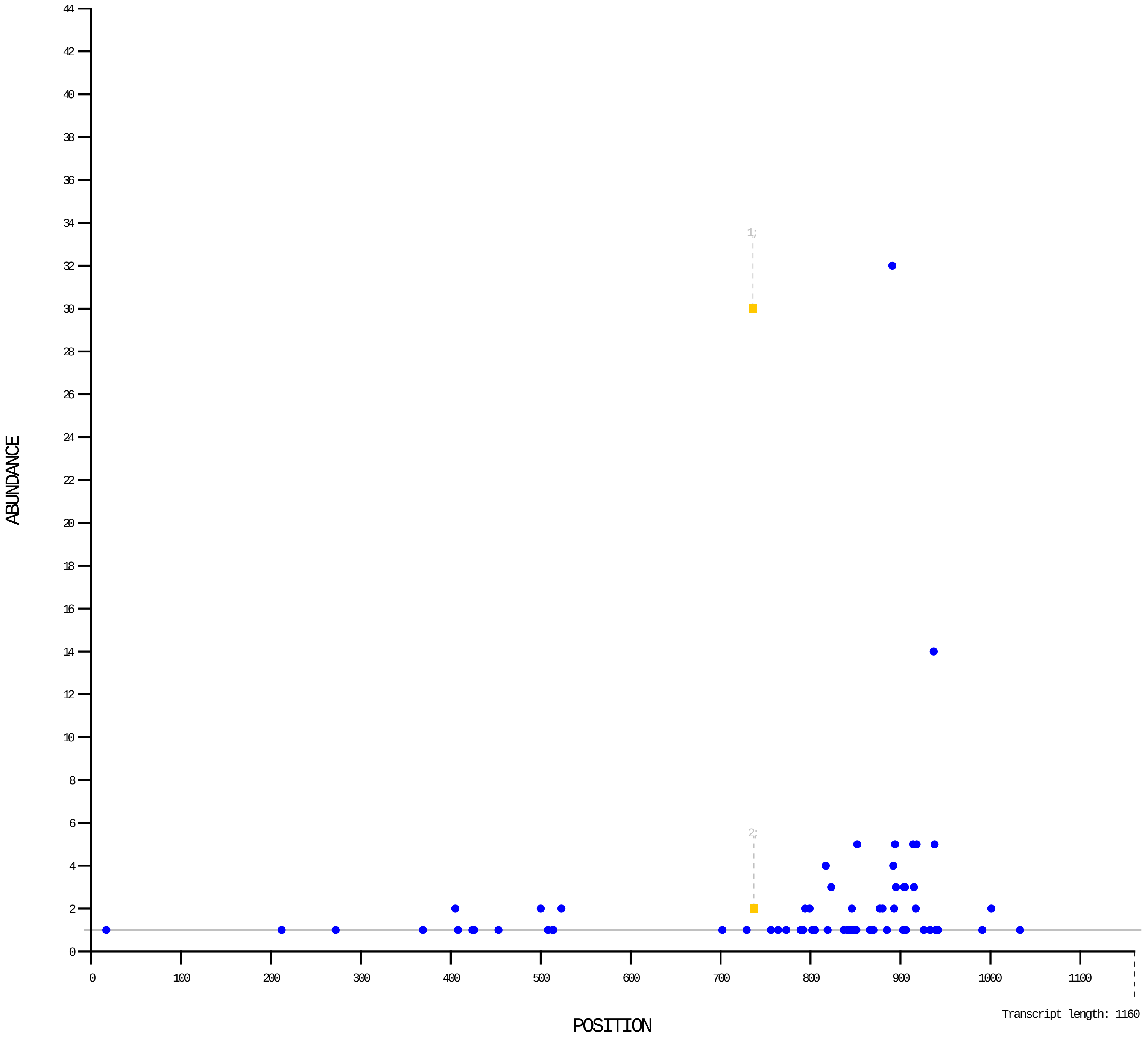

Category: 0 1 2 3 4
Degradome alignment: Median:

2 #1 Position:736 Abundance: 30.00(deg) 8(sRNA)
5' TGACAGAAGAGAGTGAGCAC 3' ID:
||||||||||||| ||||| Score: 2.0
3' ACGTACTGTCTTCTCTCTCTCGTACGAATCTA 5' p-value: 0.0

2 #2 Position:737 Abundance: 2.00(deg) 1(sRNA)
5' TTGACAGAAGAGAGCGAGCAC 3' ID:
||||||||||||| ||||| Score: 3.0
3' GACGTACTGTCTTCTCTCTCTCGTACGAATCT 5' p-value: 0.02

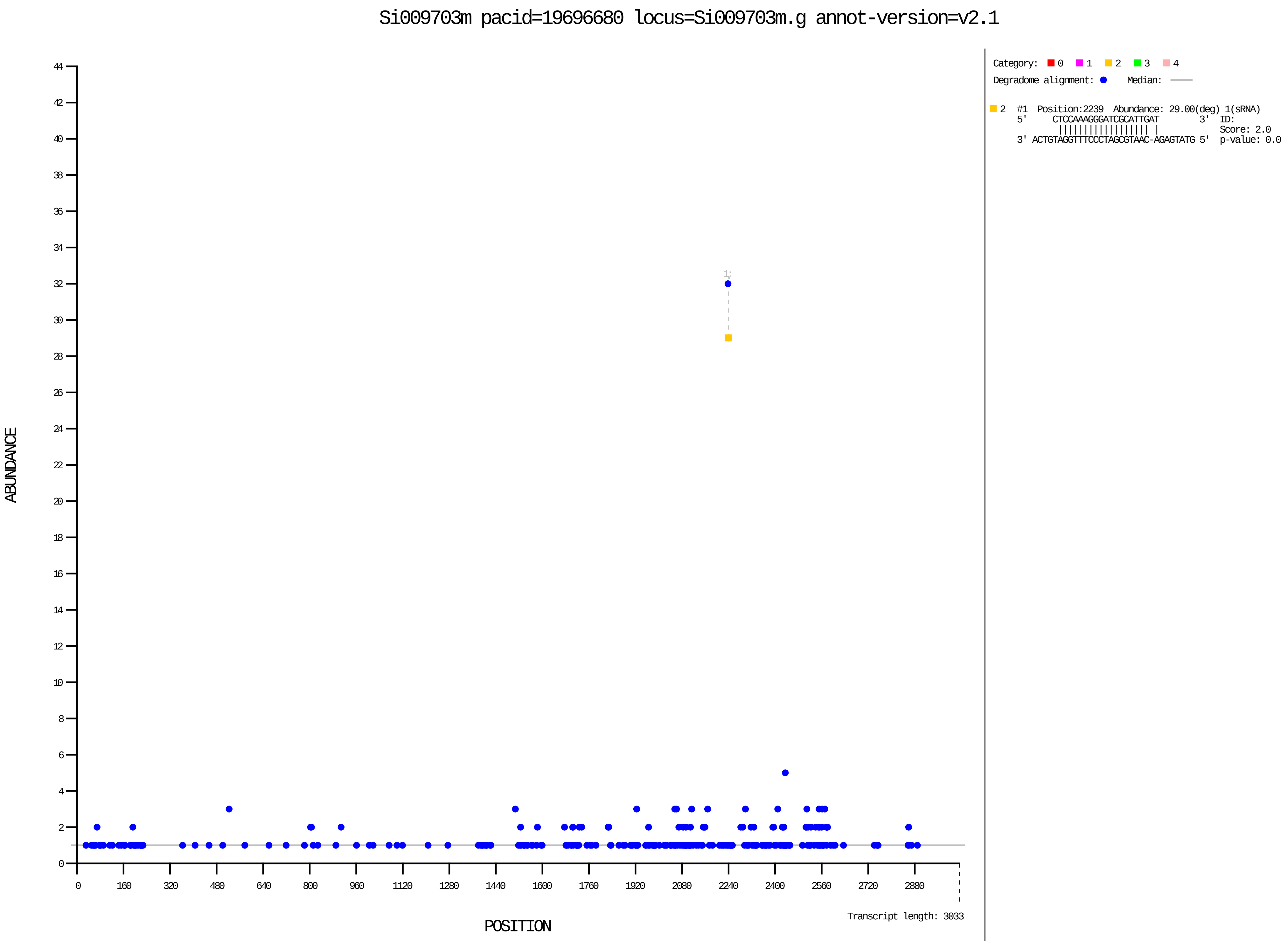

Si009703m pacid=19696680 locus=Si009703m.g annot-version=v2.1
ABUNDANCE
POSITION
Transcript length: 3033
Category: 0 1 2 3 4
Degradome alignment:
Median:
2 #1 Position:2239 Abundance: 29.00(deg) 1(sRNA)
5' CTCCAAAGGGATCGCATTGAT 3' ID:
Score: 2.0
3' ACTGTAGGTTTCCCTAGCGTAAC-AGAGTATG 5' p-value: 0.0

Si011317m pacid=19695406 locus=Si011260m.g annot-version=v2.1

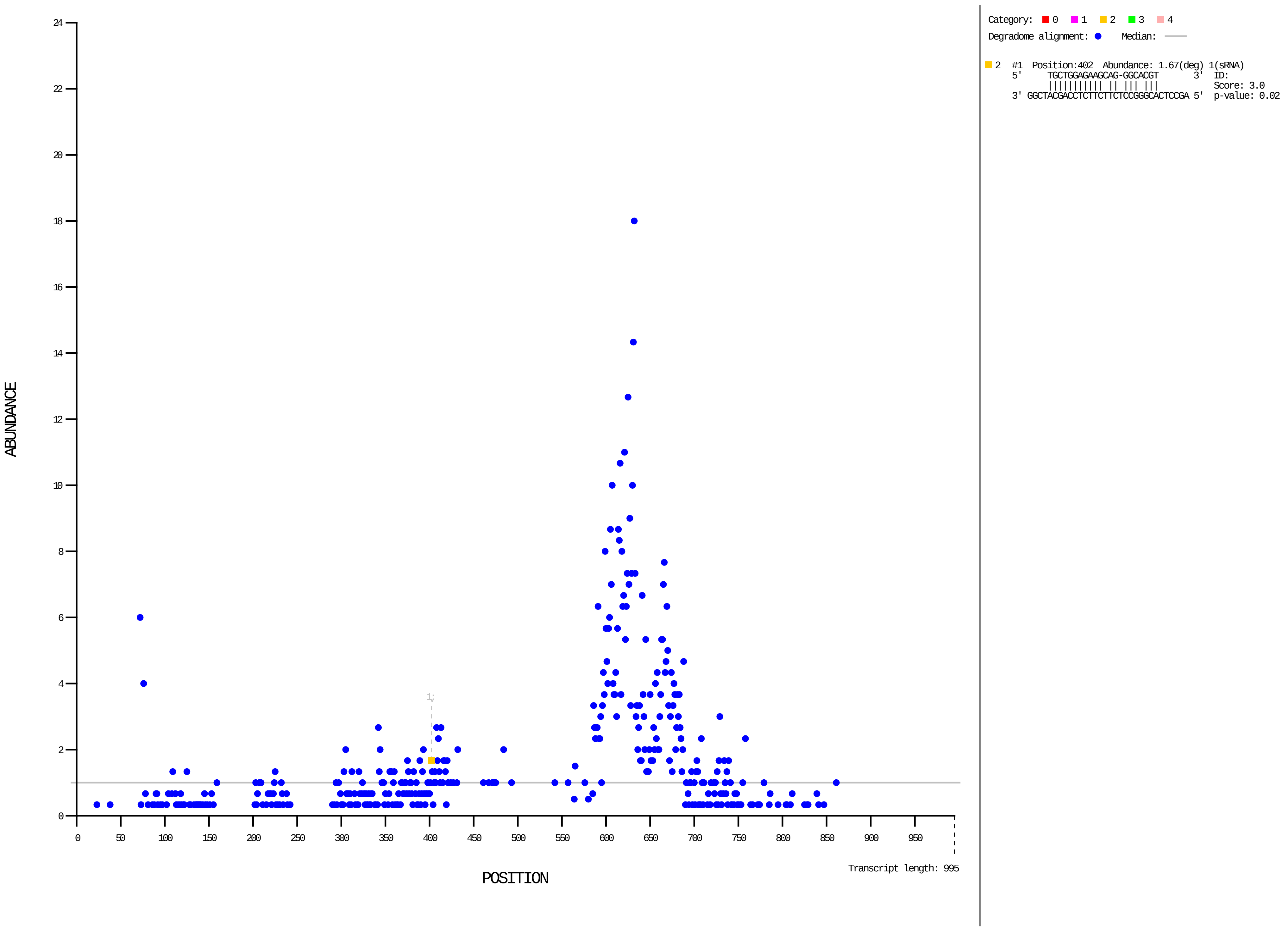

Si010282m pacid=19696127 locus=Si010282m.g annot-version=v2.1

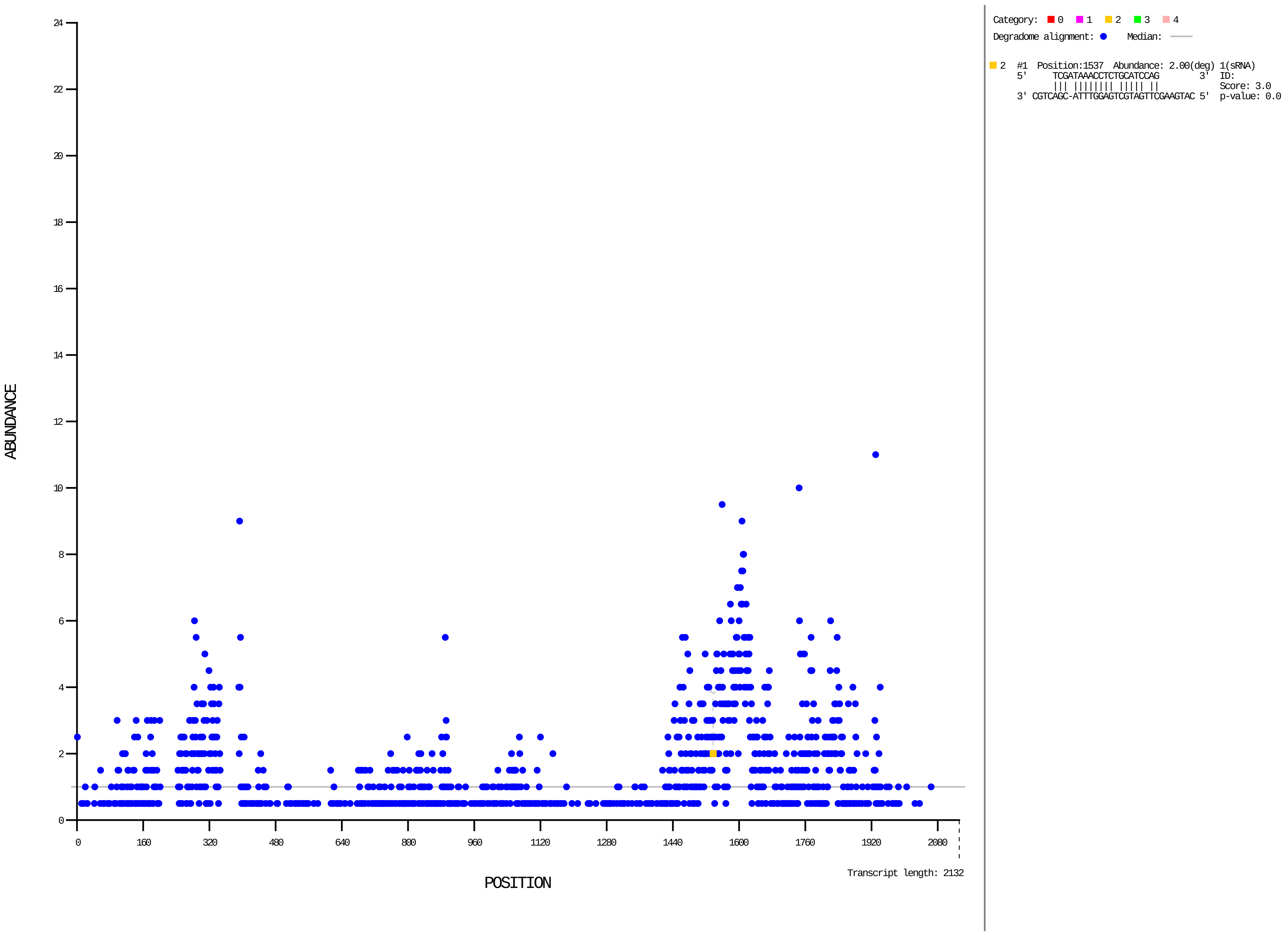

Si010280m pacid=19696128 locus=Si010282m.g annot-version=v2.1

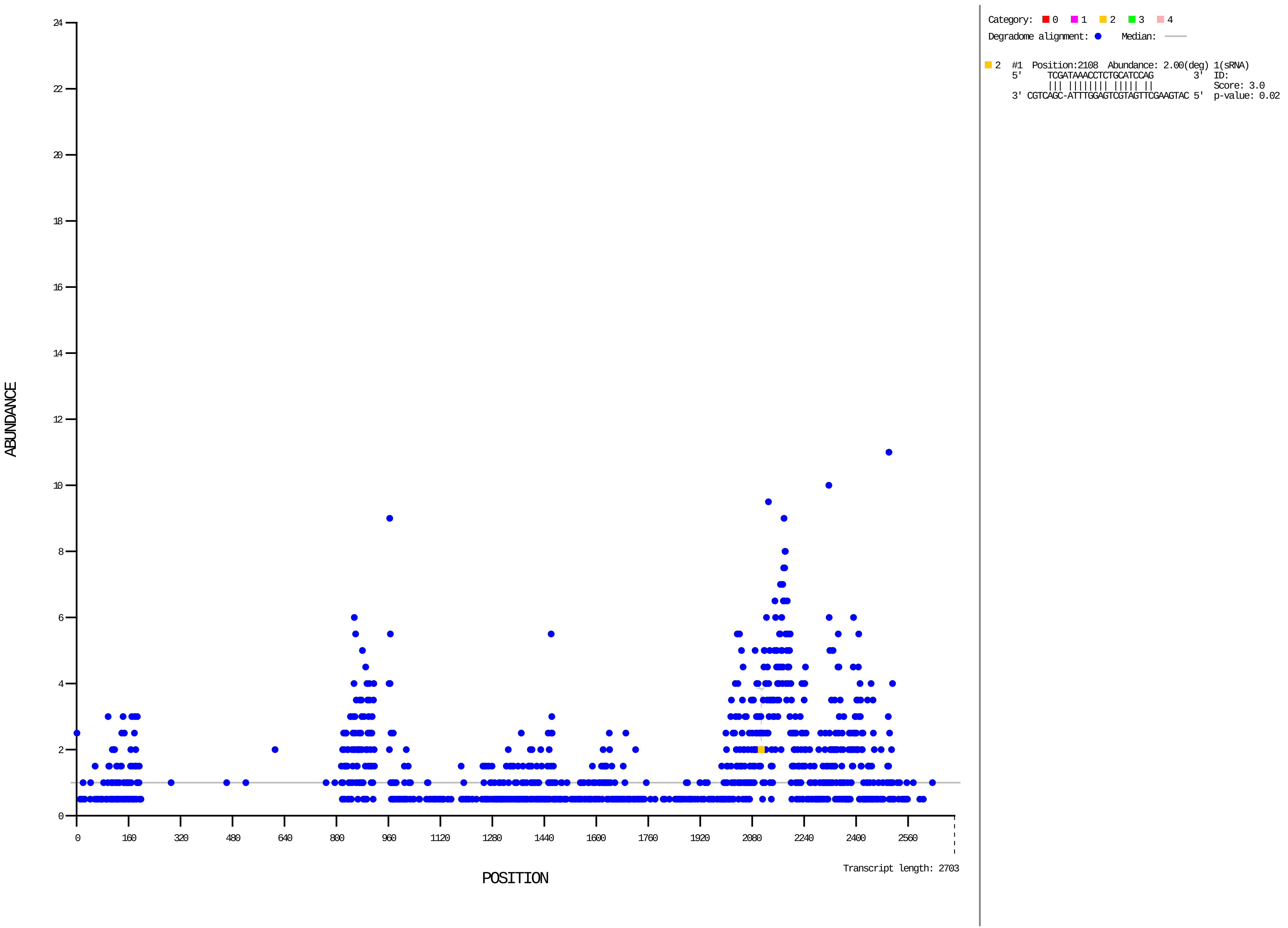

Si002017m pacid=19676299 locus=Si002017m.g annot-version=v2.1

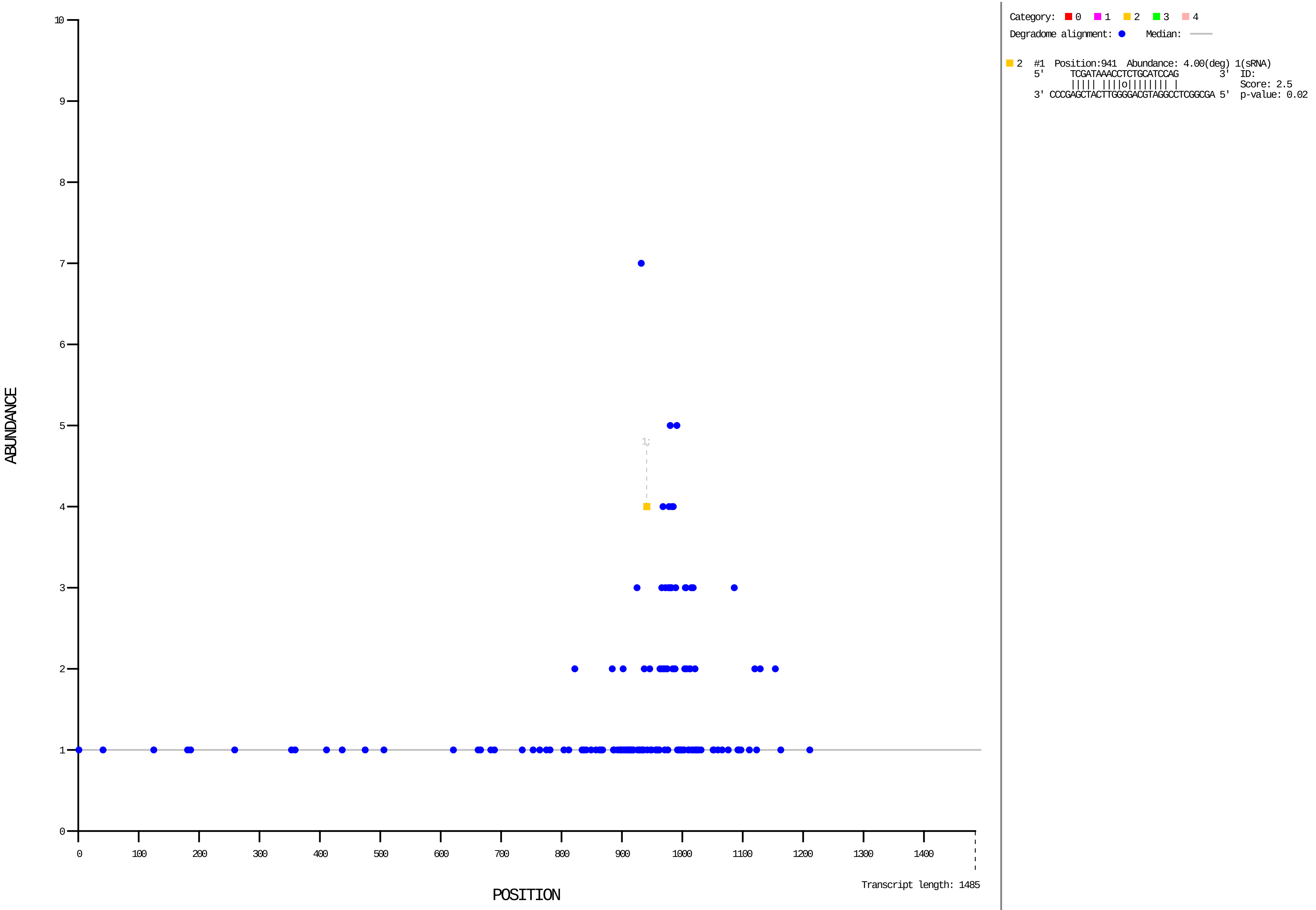

Si030725m pacid=19712780 locus=Si030725m.g annot-version=v2.1

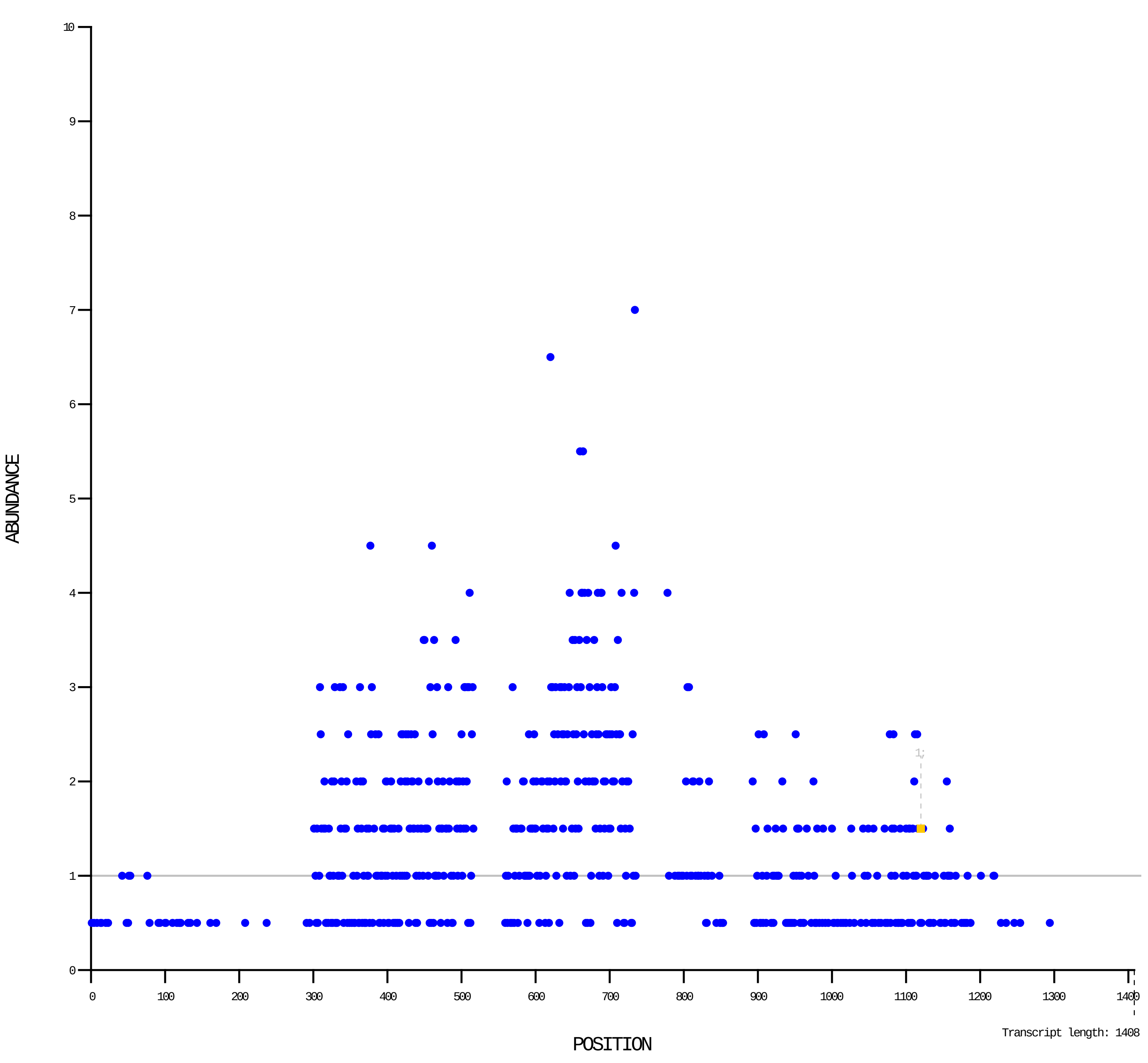

Category: 0 1 2 3 4

Degradome alignment: Median:

2 #1 Position:1120 Abundance: 1.50(deg) 1(sRNA)

```
5'     GTATGGAAGAACTGCTGCGCCA       3'  ID:
       |||o|| ||||| |||o|o|||           Score: 3.5
3' GAAACATGCCGTCTTG-CGATGTGGTGTGCTG 5'  p-value: 0.02
```

Si030969m pacid=19712781 locus=Si030725m.g annot-version=v2.1

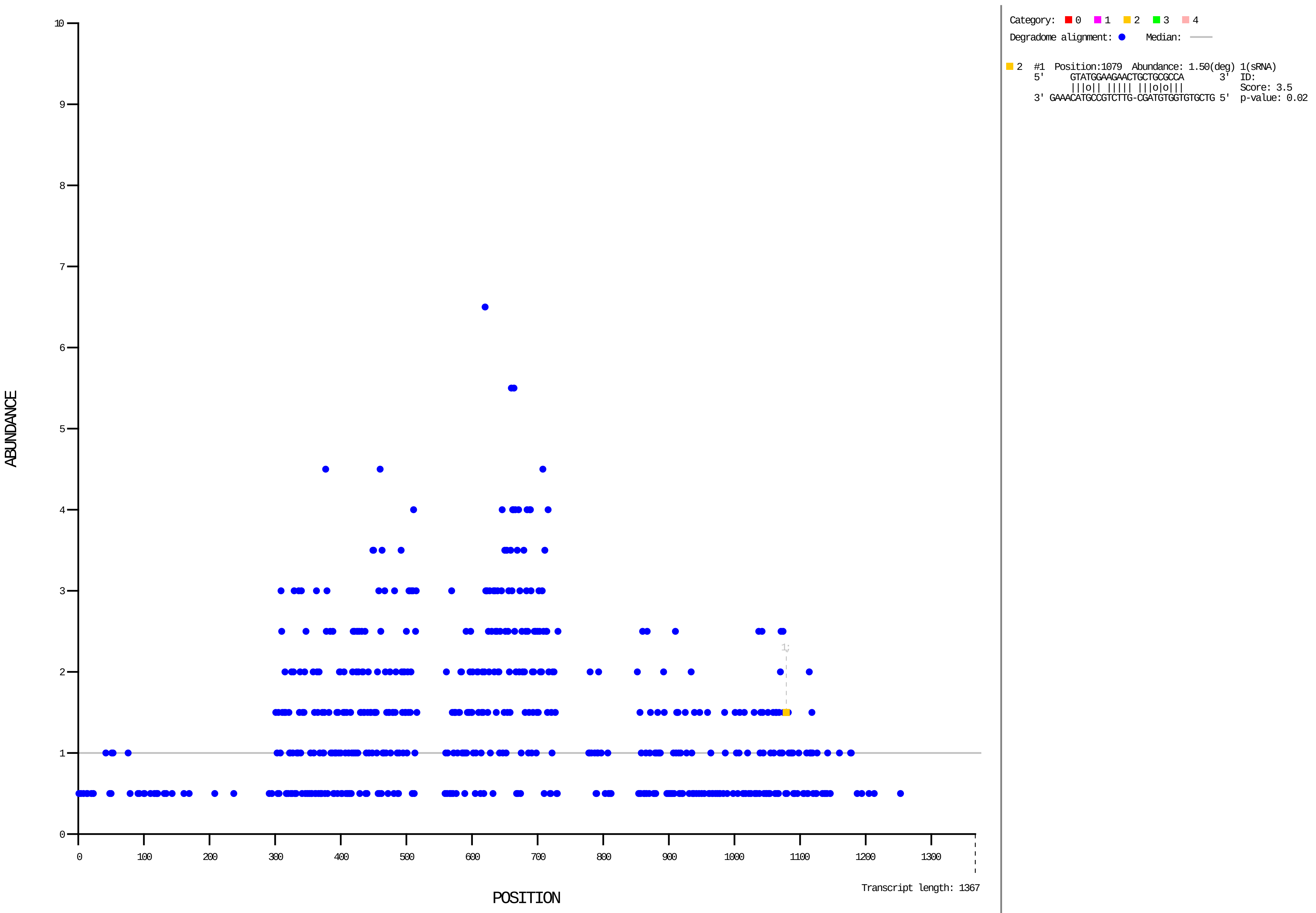

Si013308m pacid=19690928 locus=Si013308m.g annot-version=v2.1

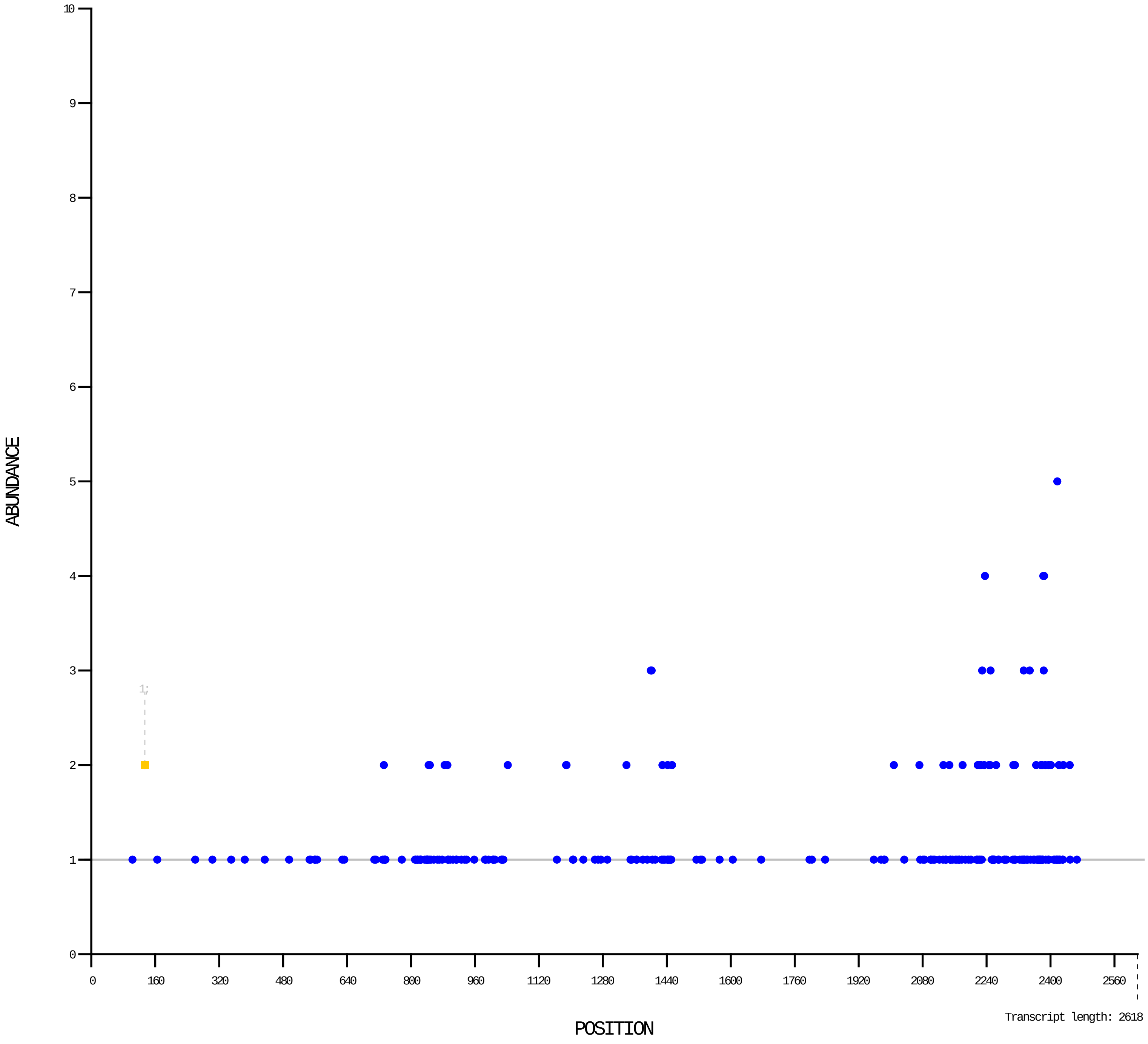

Category: 0 1 2 3 4

Degradome alignment: Median:

2 #1 Position:134 Abundance: 2.00(deg) 8(sRNA)

```
5'    TGACAGAAGAGAGTGAGCAC         3'  ID:
      ||||||| |||||o|| |||             Score: 2.5
3' CTCCACTGTCTCCTCTCGCTGGTGCGGGCAGC 5'  p-value: 0.02
```

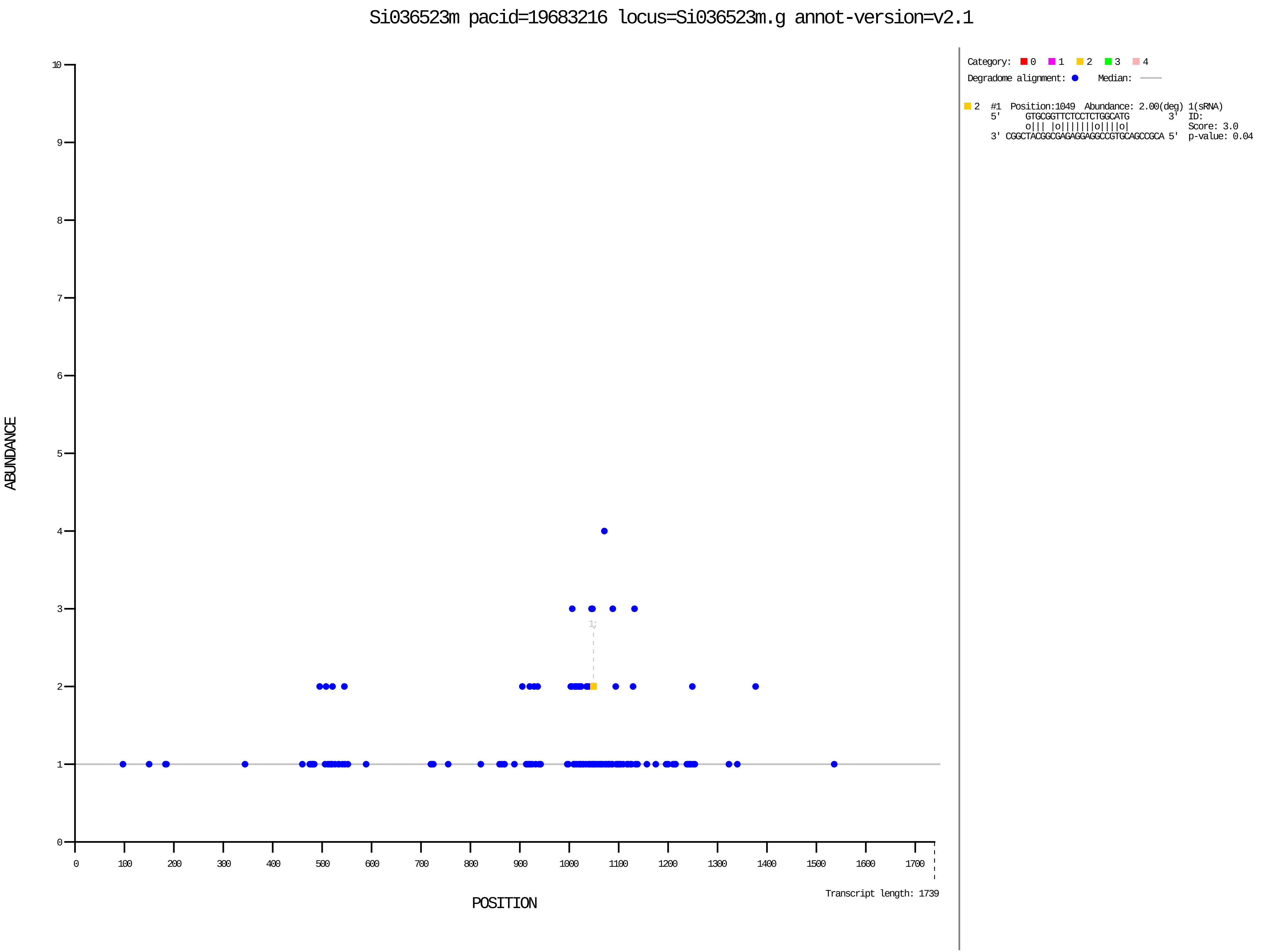
Si036523m pacid=19683216 locus=Si036523m.g annot-version=v2.1
ABUNDANCE
POSITION
Transcript length: 1739
Category: 0 1 2 3 4
Degradome alignment:
Median:
2 #1 Position:1049 Abundance: 2.00(deg) 1(sRNA)
5' GTGCGGTTCTCCTCTGGCATG 3' ID:
o||| |o|||||||o||||o|
Score: 3.0
3' CGGCTACGGCGAGAGGAGGCCGTGCAGCCGCA 5' p-value: 0.04

Si029211m pacid=19711641 locus=Si029211m.g annot-version=v2.1

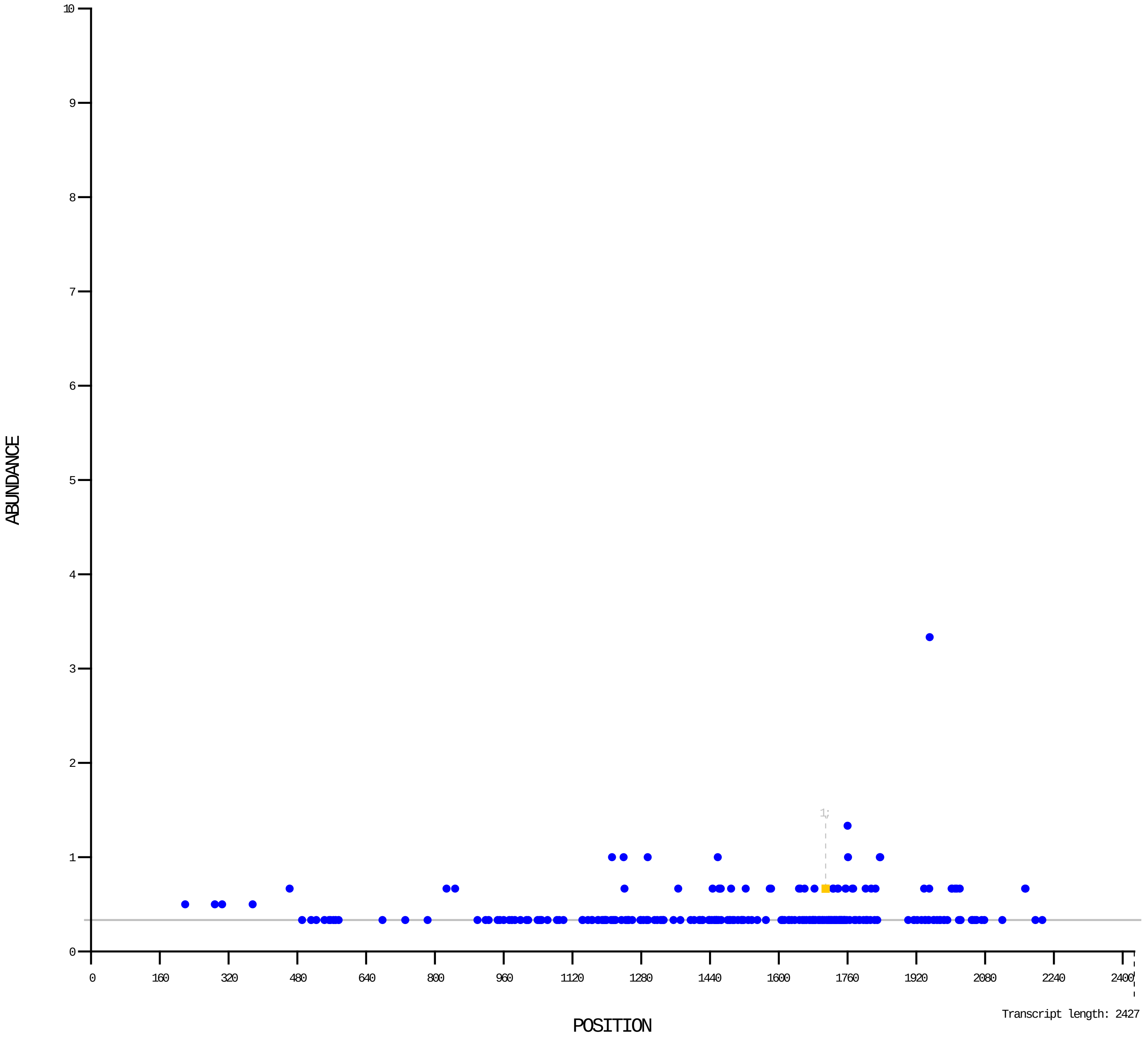

Category: 0 1 2 3 4
Degradome alignment: Median:

2 #1 Position:1709 Abundance: 0.67(deg) 1(sRNA)
5' GTATGGAAGAACTGCTGCGCCA 3' ID:
||||||||o|||||| | || | Score: 3.5
3' CTTCCATACCTTTTTGACGCC-CGATATCCGG 5' p-value: 0.02

Si029214m pacid=19711642 locus=Si029211m.g annot-version=v2.1

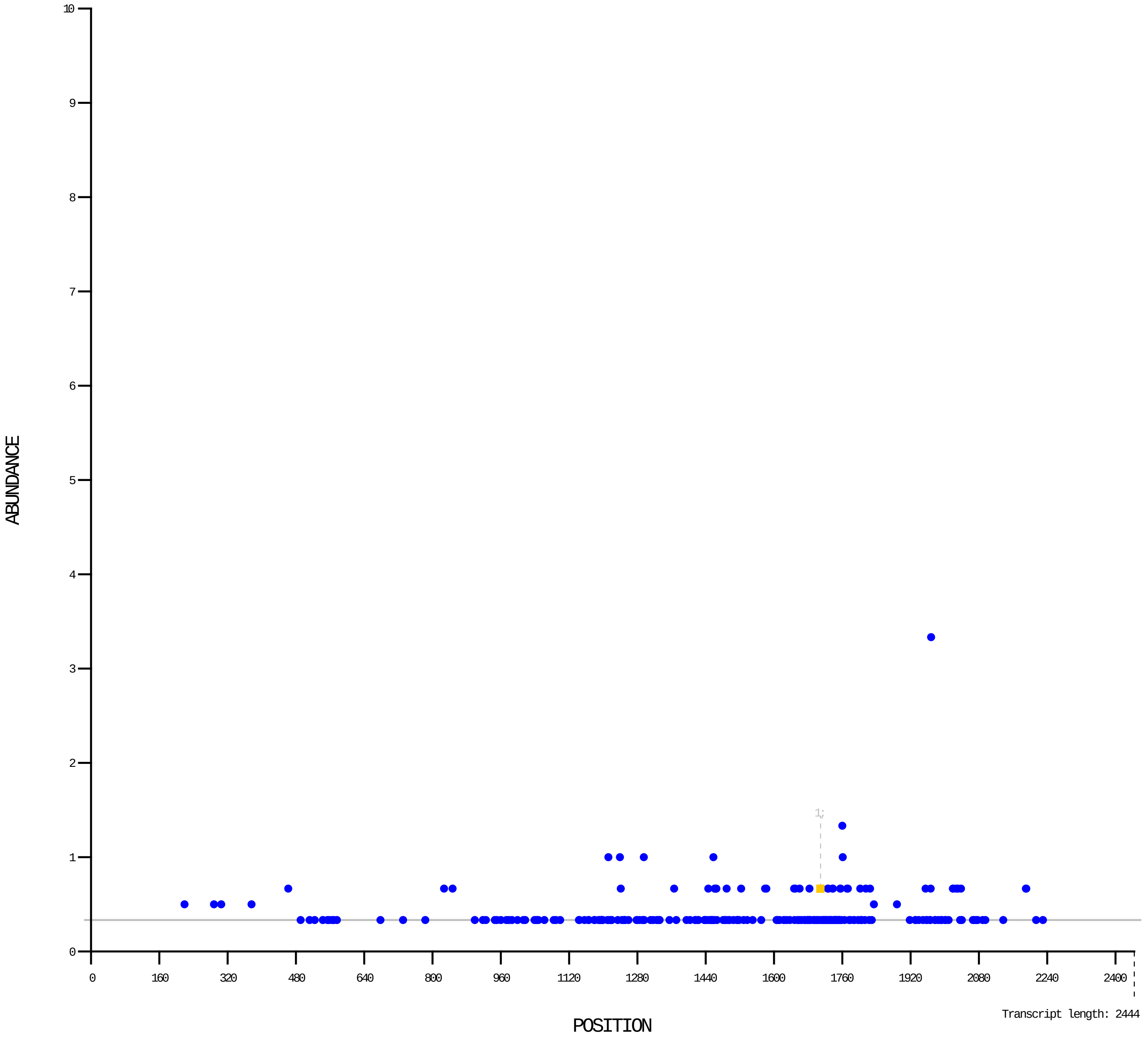

Category: 0 1 2 3 4

Degradome alignment: Median:

```
2  #1  Position:1709  Abundance: 0.67(deg) 1(sRNA)
   5'     GTATGGAAGAACTGCTGCGCCA       3'  ID:
          ||||||||o|||||| | || |           Score: 3.5
   3' CTTCCATACCTTTTTGACGCC-CGATATCCGG 5'  p-value: 0.04
```

Si029581m pacid=19711643 locus=Si029211m.g annot-version=v2.1

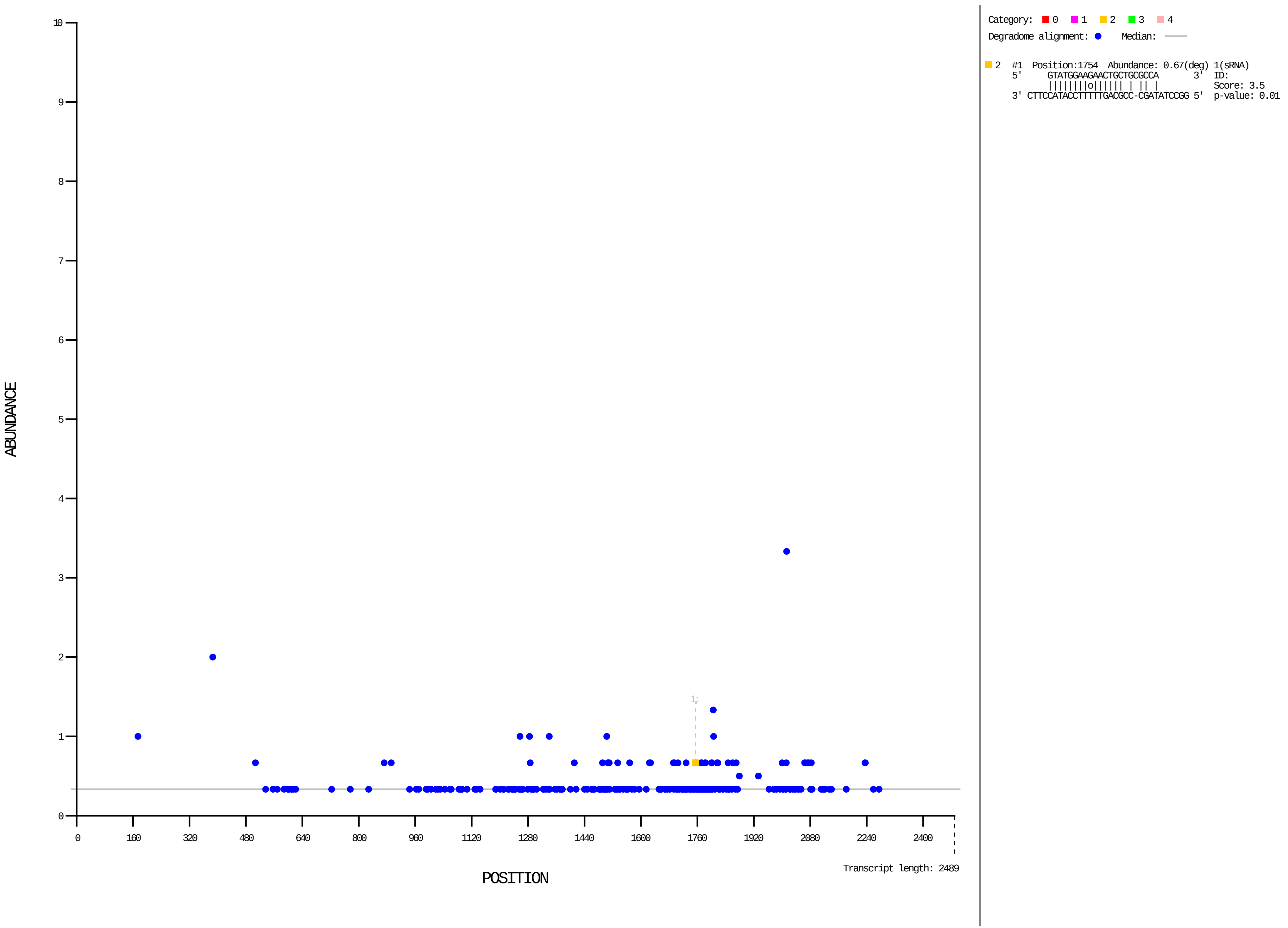

Si030195m pacid=19713916 locus=Si030195m.g annot-version=v2.1

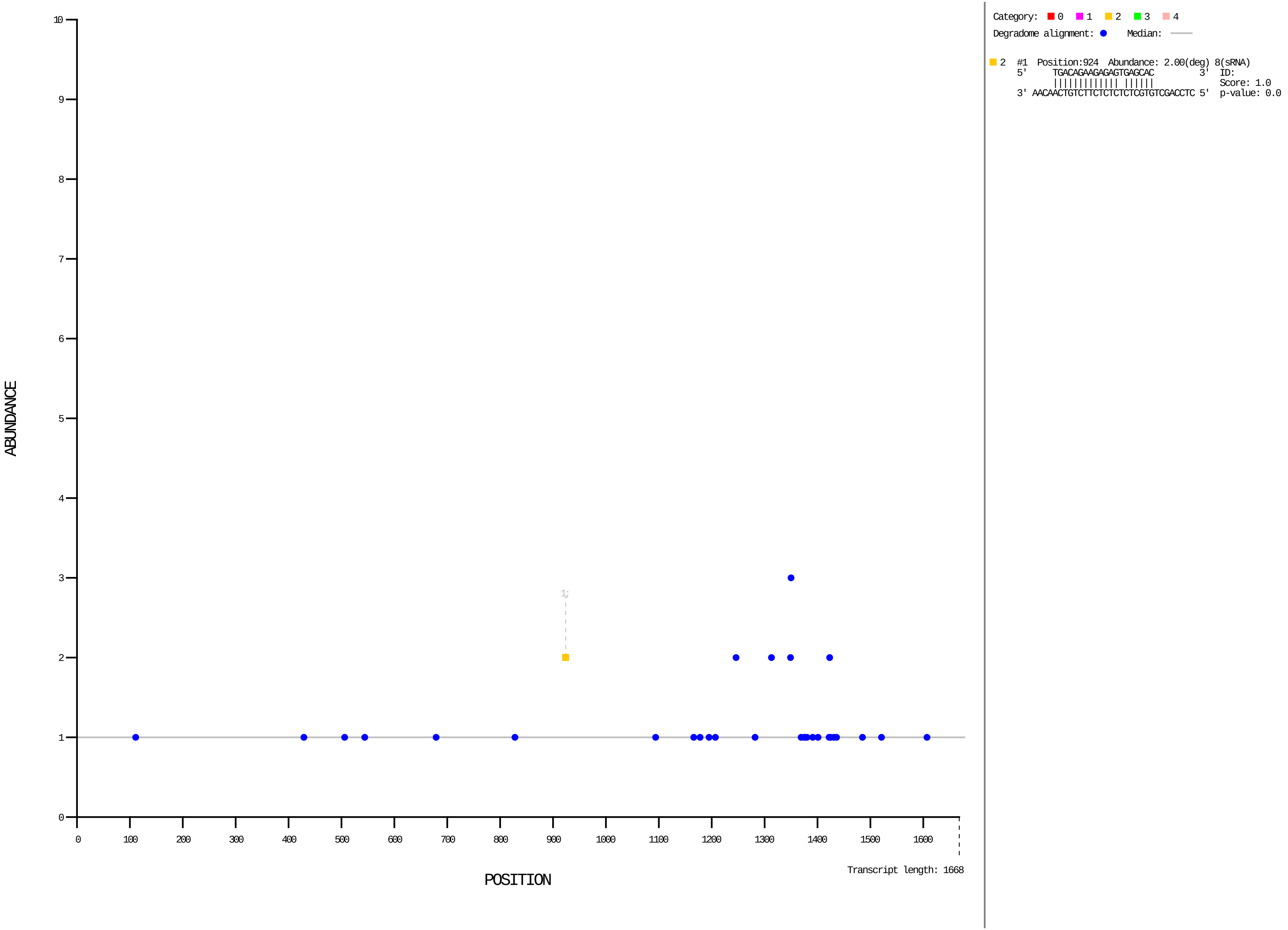

Si003343m pacid=19674388 locus=Si003343m.g annot-version=v2.1

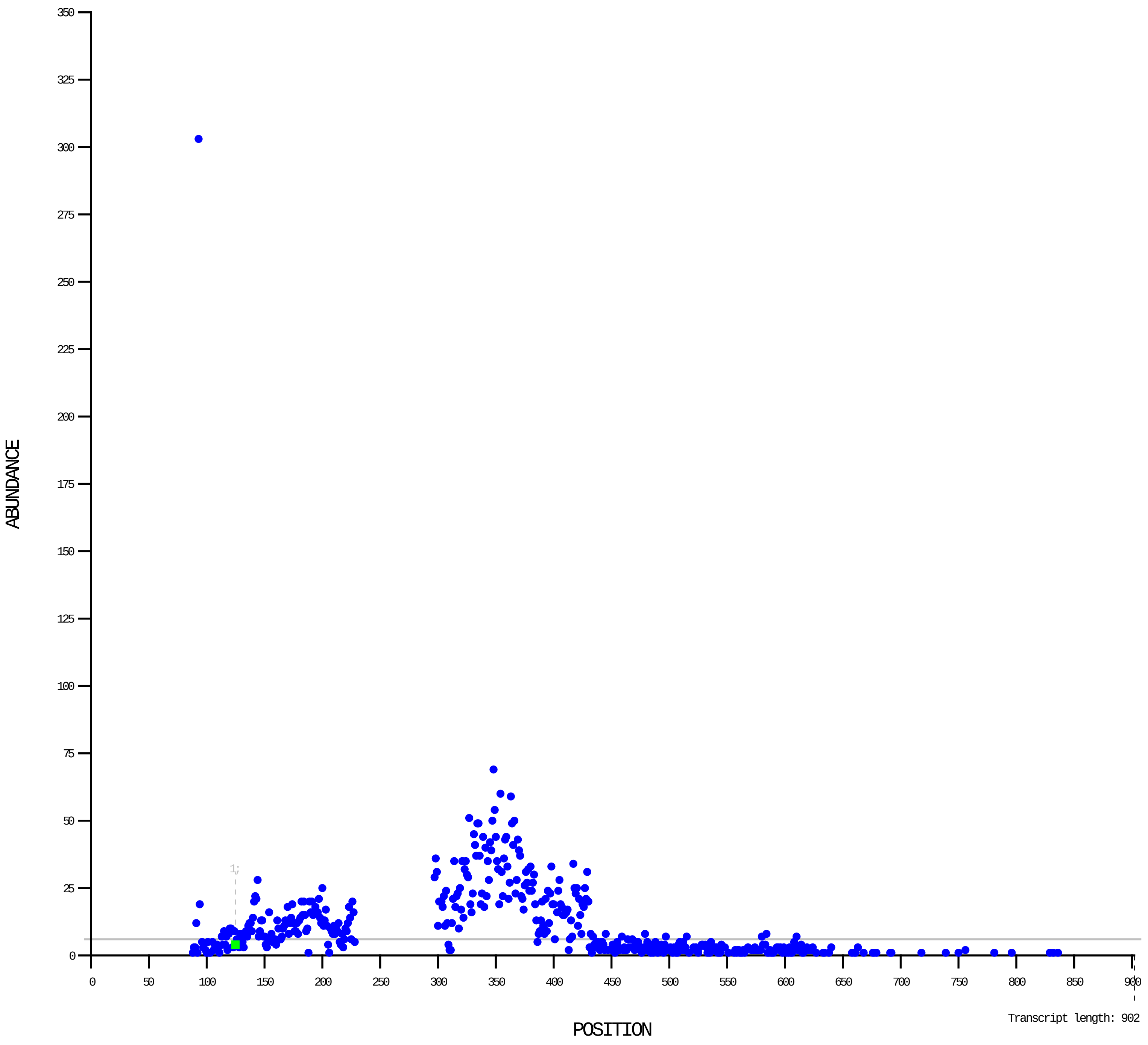

Category: 0 1 2 3 4

Degradome alignment: Median:

```
3  #1  Position:125  Abundance: 4.00(deg) 1(sRNA)
   5'     AGATCATGTTGTAGCTTCACT        3'  ID:
           |||| |||||| |||| |||            Score: 4.0
   3' AGACACTAG-ACAACACCGAAATGAACTTCTT 5'  p-value: 0.03
```

Si021433m pacid=19702410 locus=Si021433m.g annot-version=v2.1

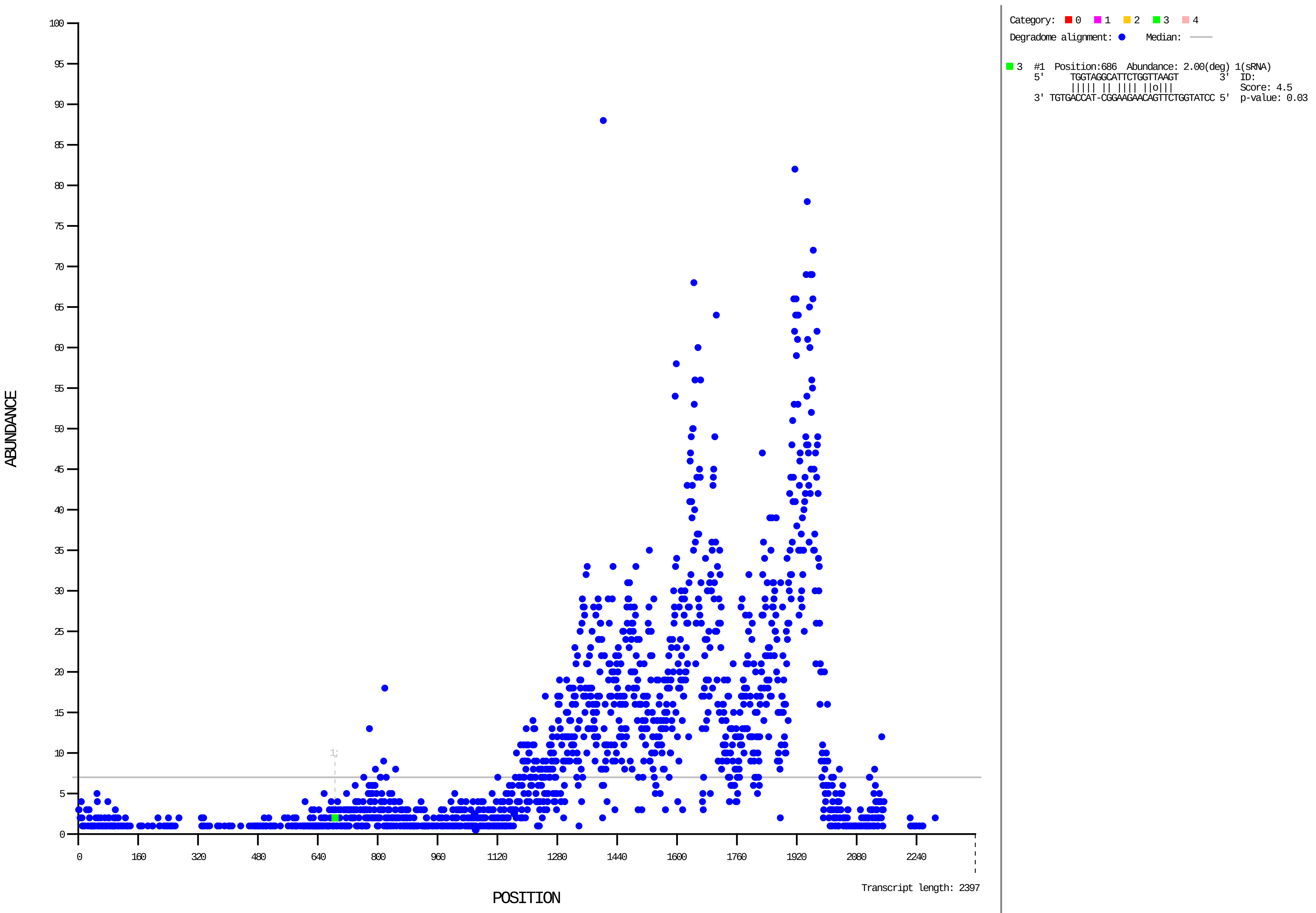

Si011260m pacid=19695405 locus=Si011260m.g annot-version=v2.1

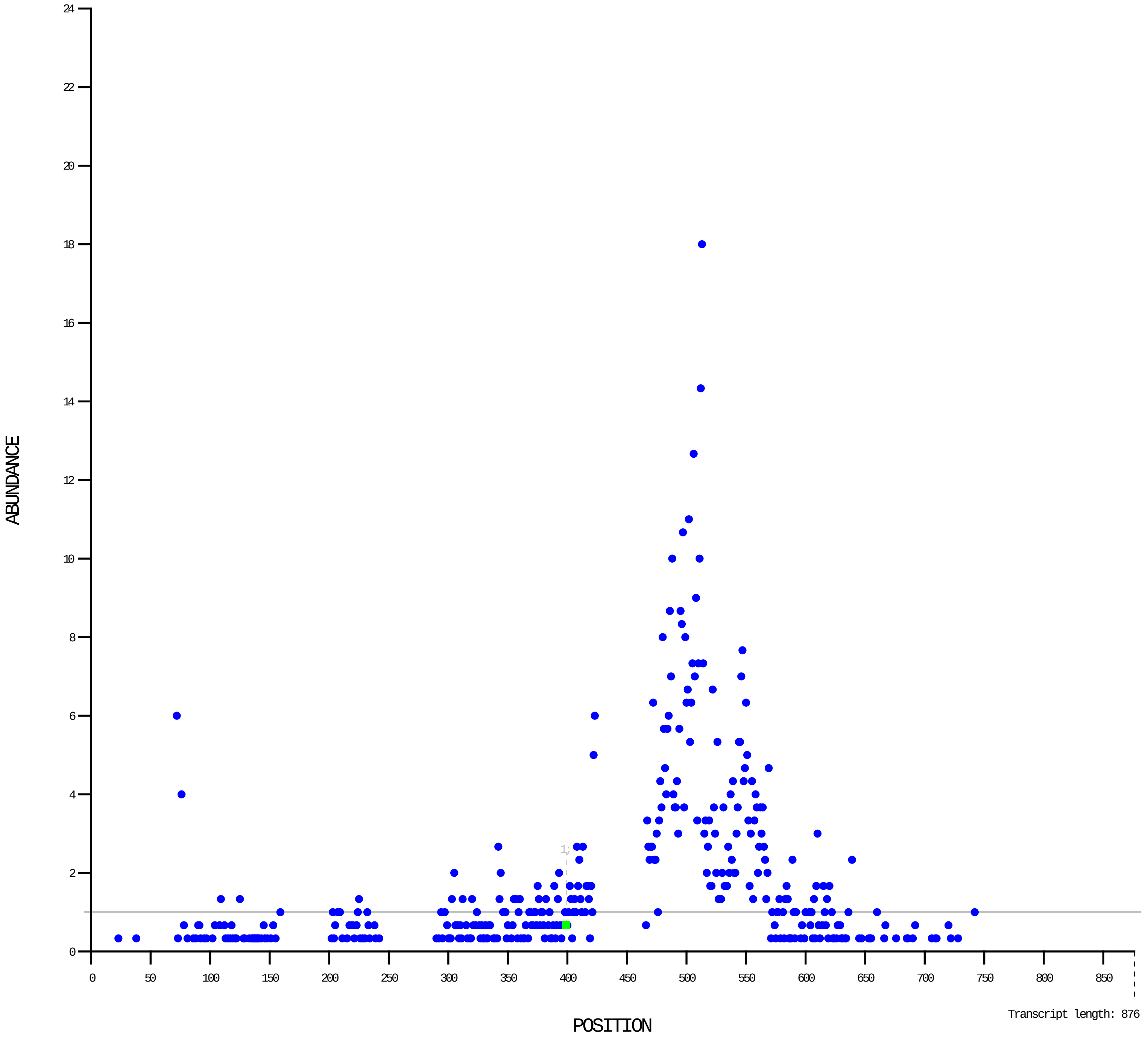


```
Category: 0 1 2 3 4
Degradome alignment:    Median:

3  #1  Position:399  Abundance: 0.67(deg) 1(sRNA)
   5'     TGGAGAAGCAG-GGCACGTGC         3'  ID:
          ||||||||| || ||| ||||              Score: 4.0
   3' TACGACCTCTTCTTCTCCGGGCACTCCGACAA 5'  p-value: 0.03
```

Si011389m pacid=19695407 locus=Si011260m.g annot-version=v2.1

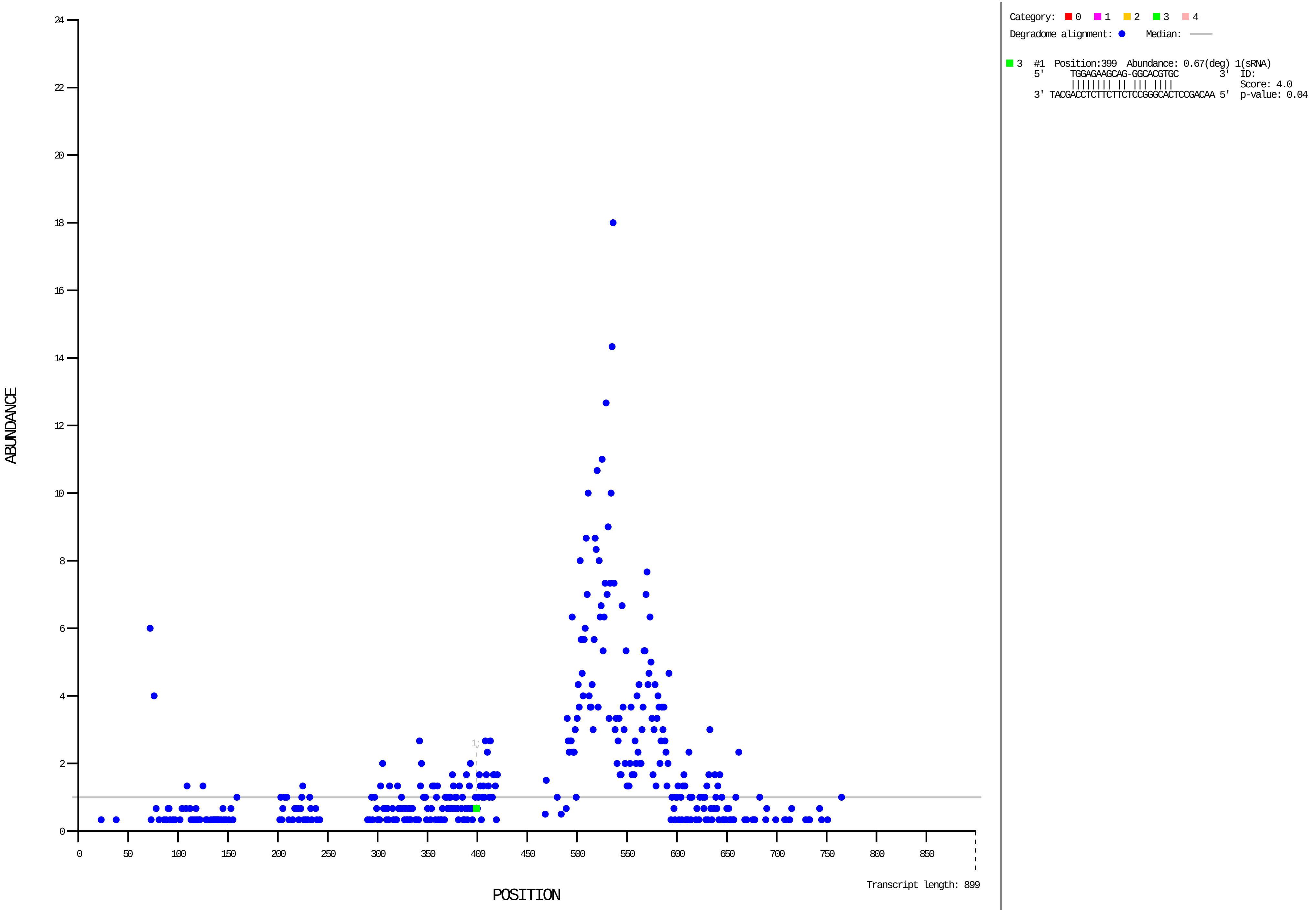

Si023679m pacid=19701413 locus=Si023662m.g annot-version=v2.1

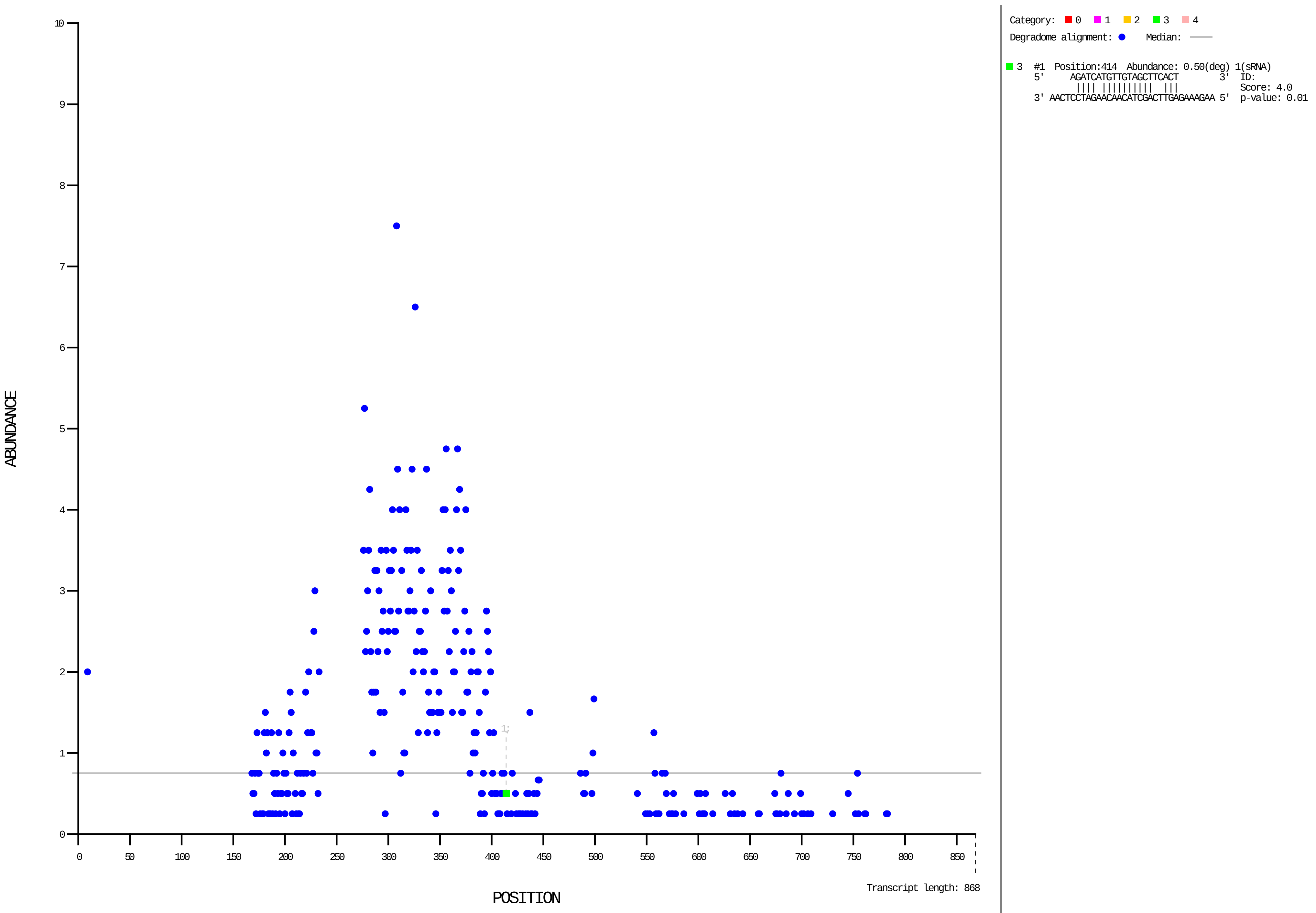

Si023678m pacid=19701412 locus=Si023662m.g annot-version=v2.1

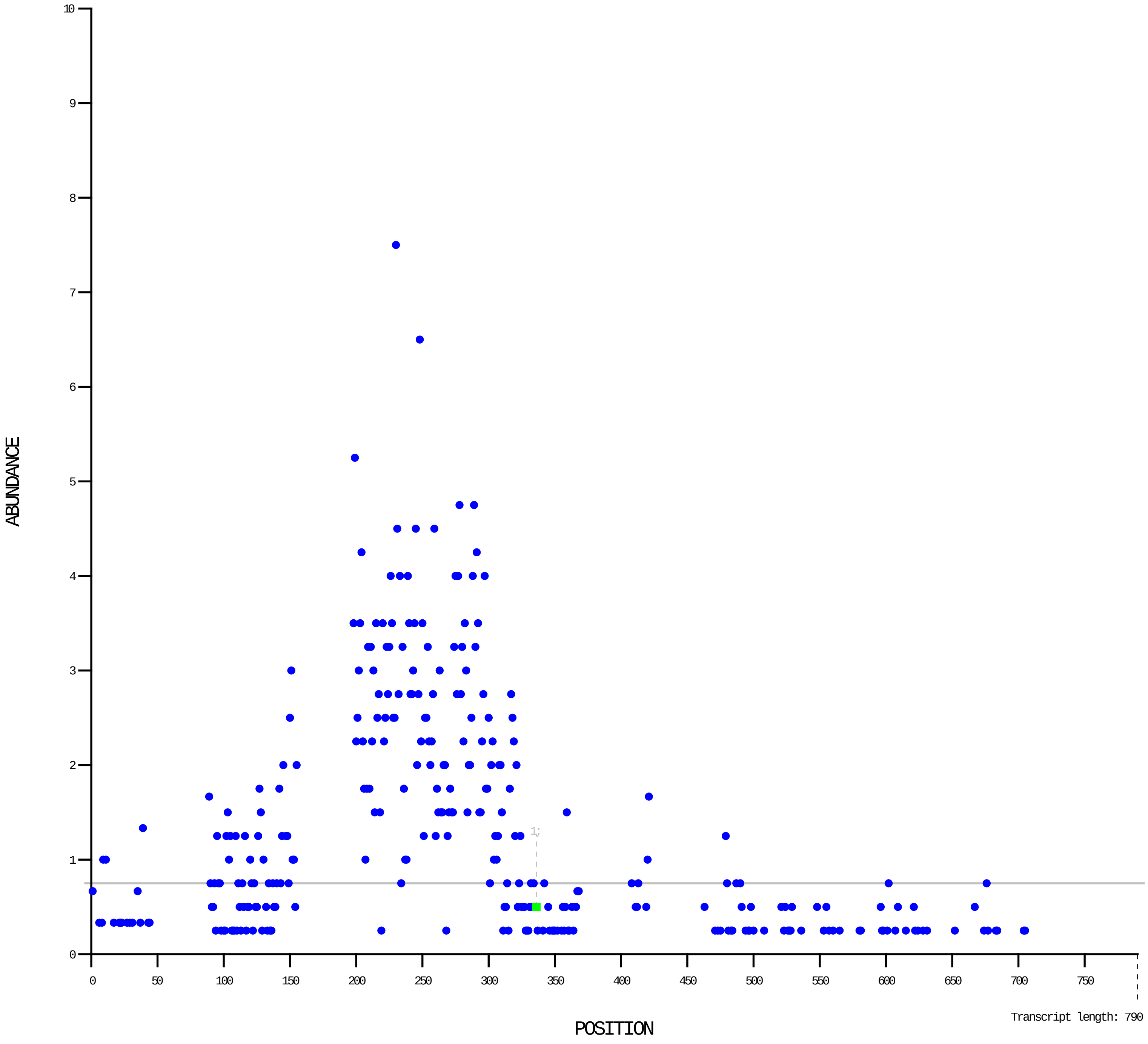

Category: 0 1 2 3 4
Degradome alignment: Median:

3 #1 Position:336 Abundance: 0.50(deg) 1(sRNA)

```
5'     AGATCATGTTGTAGCTTCACT        3'  ID:
         |||| ||||||||||  |||           Score: 4.0
3' AACTCCTAGAACAACATCGACTTGAGAAAGAA 5'  p-value: 0.03
```

Si023662m pacid=19701411 locus=Si023662m.g annot-version=v2.1

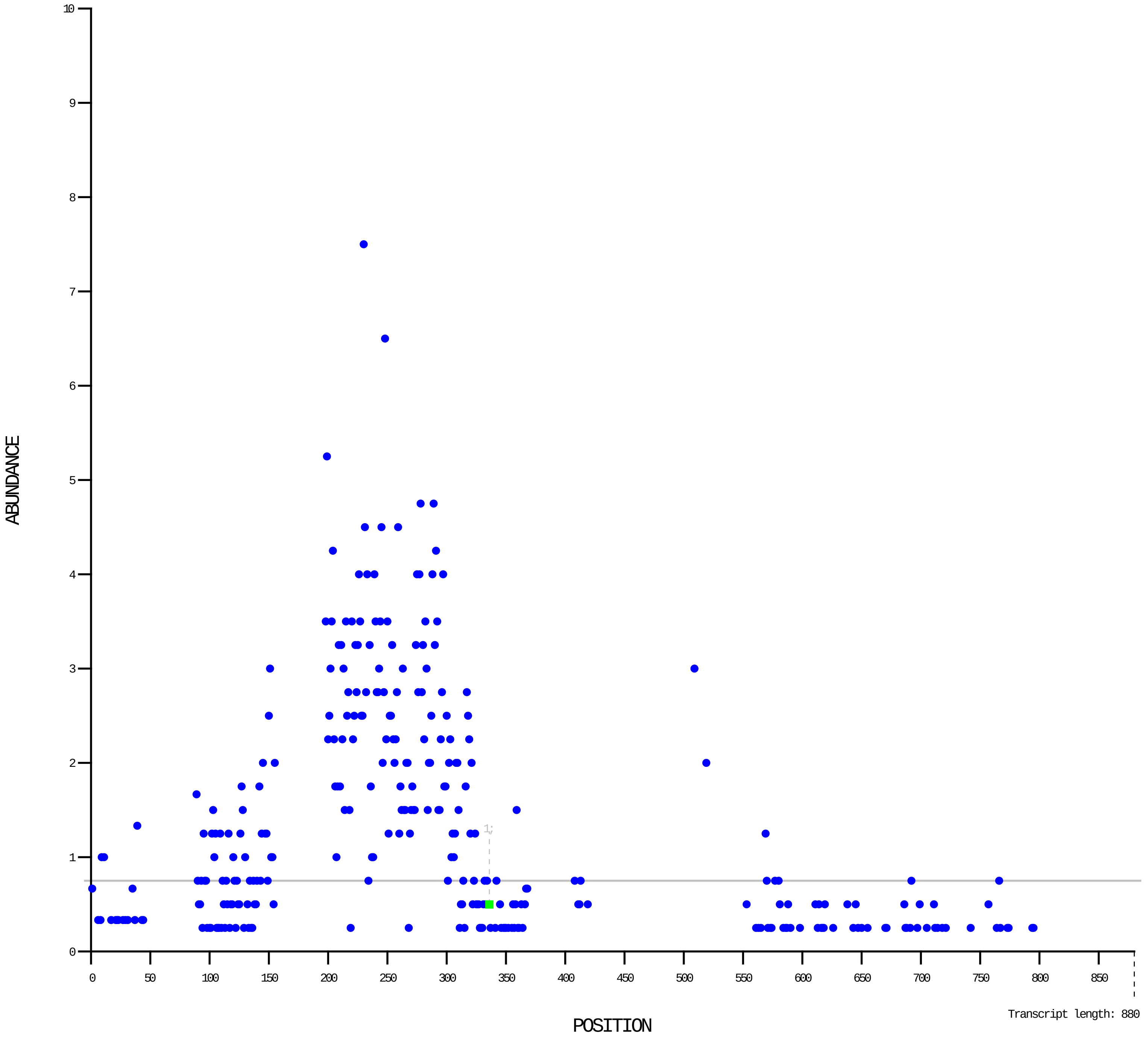

Category: 0 1 2 3 4

Degradome alignment: ● Median:

3 #1 Position:336 Abundance: 0.50(deg) 1(sRNA)

```
5'     AGATCATGTTGTAGCTTCACT        3'  ID:
         |||| |||||||||||  |||          Score: 4.0
3' AACTCCTAGAACAACATCGACTTGAGAAAGAA 5'  p-value: 0.02
```

Si023821m pacid=19701414 locus=Si023662m.g annot-version=v2.1

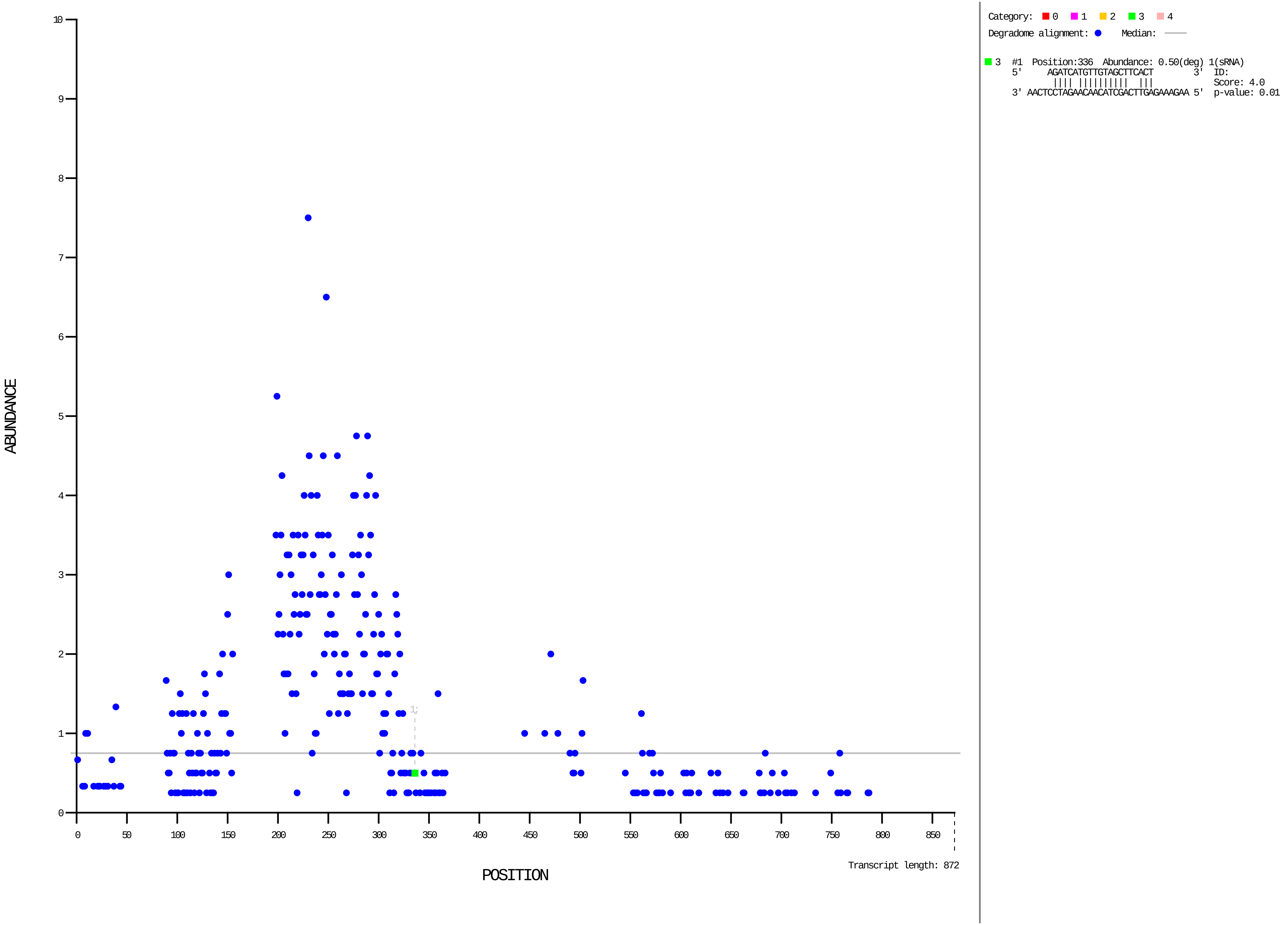

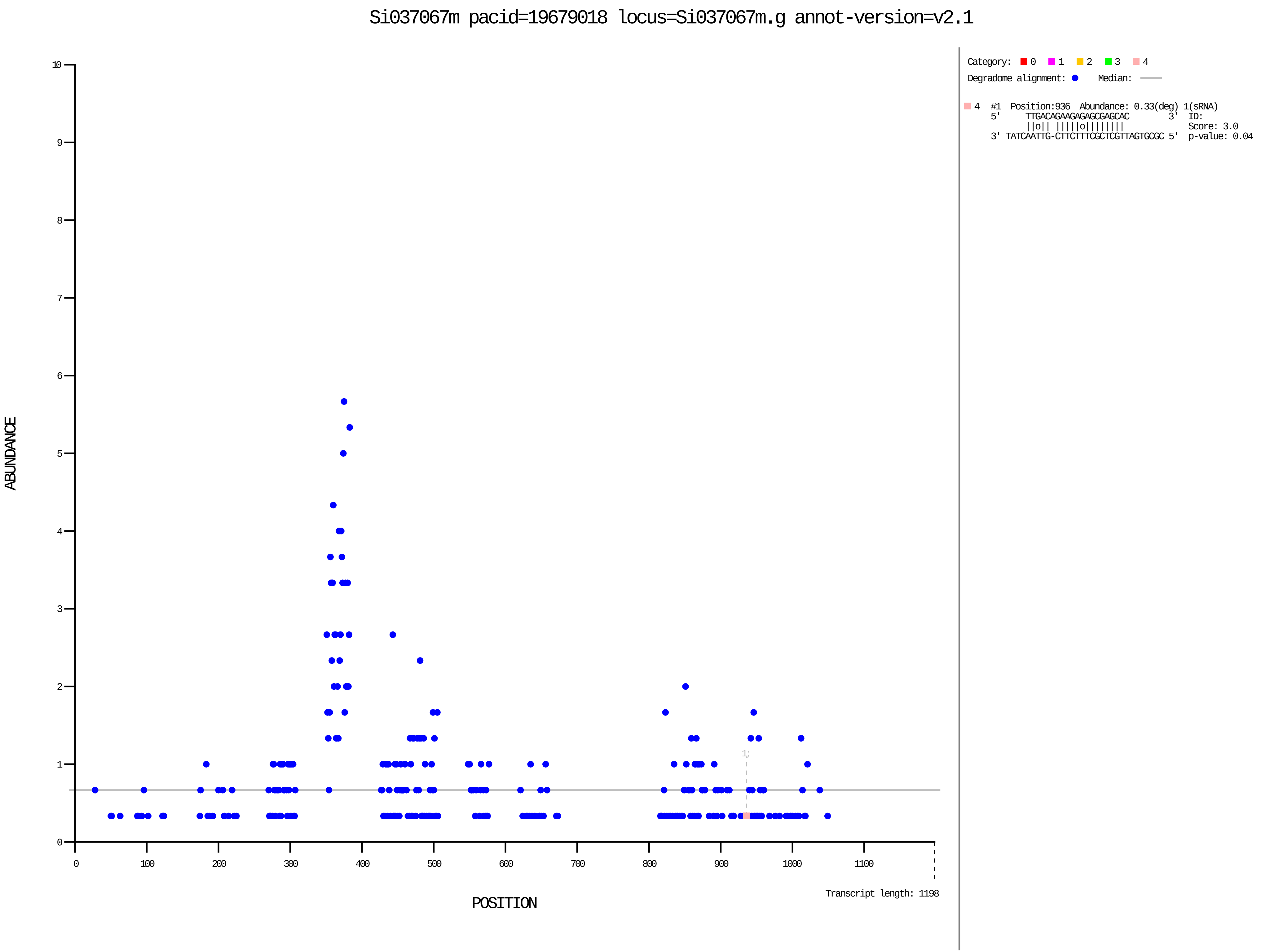

Si037067m pacid=19679018 locus=Si037067m.g annot-version=v2.1
ABUNDANCE
POSITION
Transcript length: 1198
Category: 0 1 2 3 4
Degradome alignment:
Median:
4 #1 Position:936 Abundance: 0.33(deg) 1(sRNA)
5' TTGACAGAAGAGAGCGAGCAC 3' ID:
||o|| |||||o||||||||
3' TATCAATTG-CTTCTTTCGCTCGTTAGTGCGC 5'
Score: 3.0
p-value: 0.04

Si006517m pacid=19705913 locus=Si006517m.g annot-version=v2.1

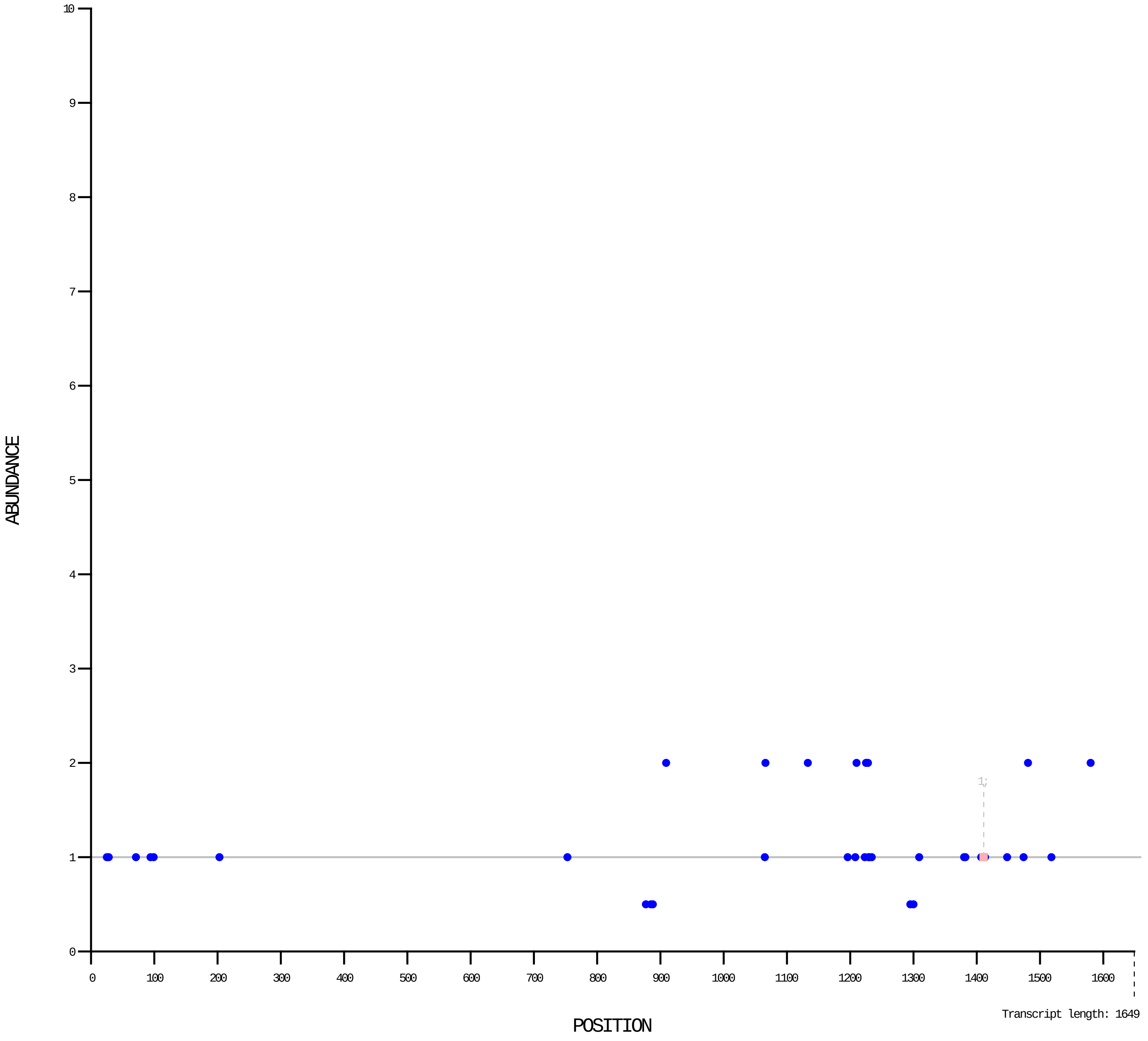


```
Category: 0 1 2 3 4
Degradome alignment:     Median:

4  #1  Position:1411  Abundance: 1.00(deg) 1(sRNA)
   5'     TTGAGCCGCGCCAATATCTCT          3'  ID:
          o ||| |||||||||||||o||             Score: 3.0
   3' TGCCGCCTC-GCGCGGTTATAGGGATCAACTG 5'  p-value: 0.03
```

Si000283m pacid=19673633 locus=Si000283m.g annot-version=v2.1

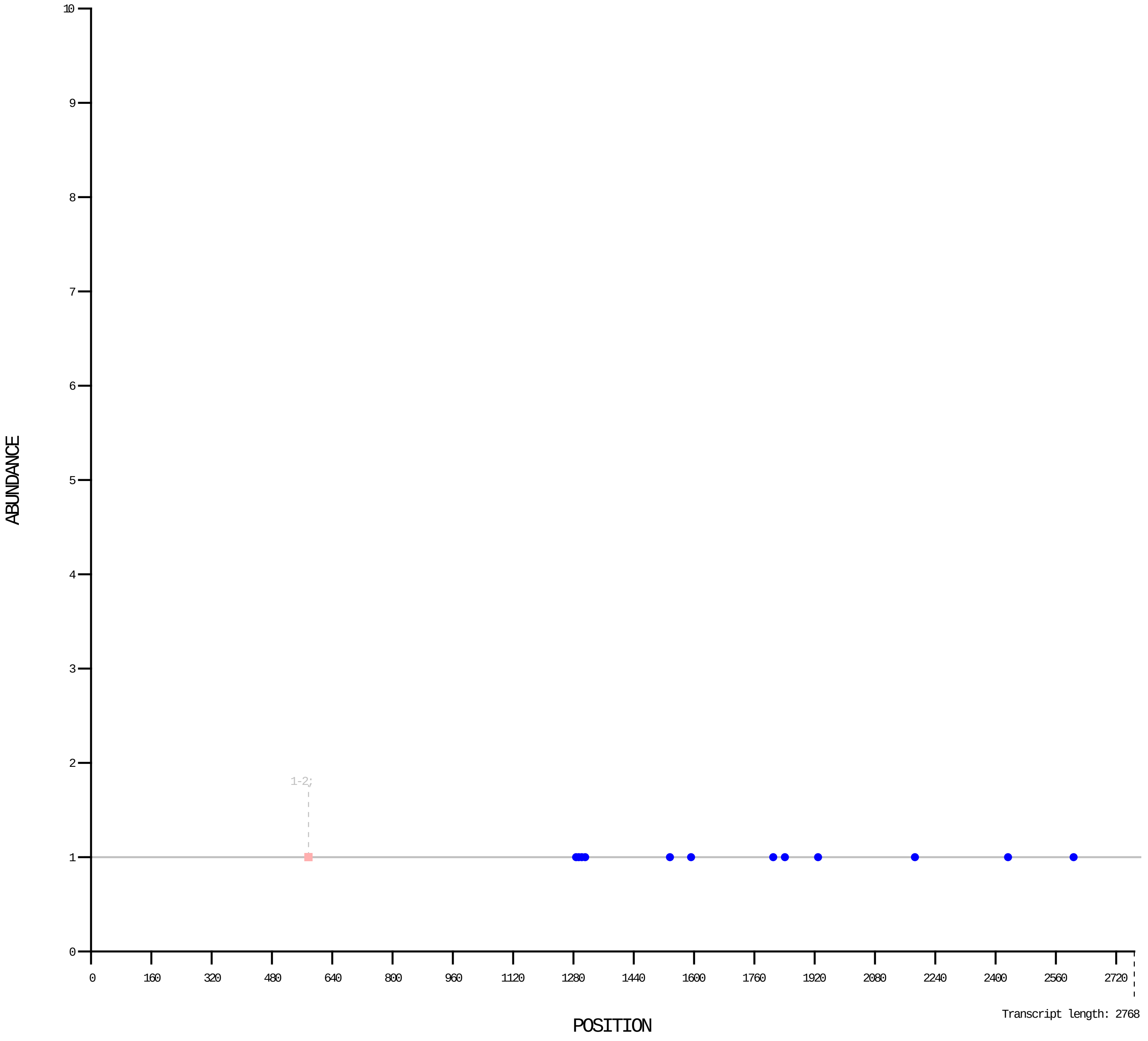

Category: 0 1 2 3 4

Degradome alignment: Median:

4 #1 Position:577 Abundance: 1.00(deg) 7(sRNA)
5' TCGGACCAGGCTTCATTCCCC 3' ID:
o|||||||||||||| ||| Score: 2.5
3' CTTAGGCCTGGTCCGAAGTA-GGGTCCGTAGA 5' p-value: 0.04

4 #2 Position:577 Abundance: 1.00(deg) 2(sRNA)
5' TCGGACCAGGCTTCAATCCCT 3' ID:
o||||||||||||| |||| Score: 2.5
3' CTTAGGCCTGGTCCGAAGT-AGGGTCCGTAGA 5' p-value: 0.03
