# Supplementary material for: Combined small RNA and degradome sequencing to identify miRNAs and their targets in response to drought in foxtail millet
Source: BMC Genet. 2016 Apr 12;17:57. doi: 10.1186/s12863-016-0364-7 (PMC4828802; doi:10.1186/s12863-016-0364-7)
Supplement: Additional file 10: — Targets of novel miRNAs identified by degradome sequencing. (DOC 57 kb) [file 12863_2016_364_MOESM10_ESM.doc]

**Additional file 10.** Targets of novel miRNAs identified by degradome sequencing

| **Family** | **Target gene** | **Cleave position** | **Category** | **Alignment score** | **Target gene annotation** |
| --- | --- | --- | --- | --- | --- |
| sit_novel_miR10 | Si029581m | 2 | 1754 | 3.5 | hydroxyproline-rich glycoprotein family protein |
|  | Si030725m | 2 | 1120 | 3.5 | THYLAKOID FORMATION1 |
|  | Si030969m | 2 | 1079 | 3.5 | THYLAKOID FORMATION1 |
|  | Si029214m | 2 | 1709 | 3.5 | hydroxyproline-rich glycoprotein family protein |
|  | Si029211m | 2 | 1709 | 3.5 | hydroxyproline-rich glycoprotein family protein |
| sit_novel_miR21 | Si036523m | 2 | 1049 | 3 | Tetratricopeptide repeat (TPR)-like superfamily protein |
| sit_novel_miR3 | Si021181m | 0 | 114 | 1.5 | ubiquitin conjugating enzyme protein |
|  | Si021179m | 0 | 114 | 1.5 | ubiquitin conjugating enzyme protein |
| sit_novel_miR37 | Si023821m | 3 | 336 | 4 | Translation initiation factor SUI1 family protein |
|  | Si023662m | 3 | 336 | 4 | Translation initiation factor SUI1 family protein |
|  | Si023678m | 3 | 336 | 4 | Translation initiation factor SUI1 family protein |
|  | Si023679m | 3 | 414 | 4 | Translation initiation factor SUI1 family protein |
|  | Si003343m | 3 | 125 | 4 | calvin cycle protein |
| sit_novel_miR38 | Si011389m | 2 | 402 | 3 | membrane protein |
|  | Si017567m | 4 | 867 | 2.5 | NAC domain containing protein |
|  | Si011260m | 2 | 402 | 3 | membrane protein |
|  | Si035812m | 0 | 1149 | 3.5 | cycling DOF factor |
|  | Si017570m | 4 | 843 | 2.5 | NAC domain containing protein |
|  | Si022747m | 4 | 794 | 2 | NAC domain containing protein |
|  | Si017931m | 4 | 971 | 2.5 | NAC domain containing protein |
|  | Si011317m | 2 | 402 | 3 | membrane protein |
| sit_novel_miR48 | Si021433m | 3 | 686 | 4.5 | heat shock protein |
| sit_novel_miR50 | Si010741m | 1 | 171 | 4 | Protein of unknown function |
| sit_novel_miR54 | Si011264m | 2 | 825 | 3 | albumin superfamily protein |
| sit_novel_miR62 | Si014342m | 1 | 839 | 4 | Protein of unknown function |
|  | Si011165m | 1 | 527 | 4.5 | Disease resistance-responsive (dirigent-like protein) family protein |
